# Zirconocene-Catalyzed Dimerization of α-Olefins: DFT Modeling of the Zr-Al Binuclear Reaction Mechanism

**DOI:** 10.3390/molecules24193565

**Published:** 2019-10-02

**Authors:** Ilya Nifant’ev, Alexander Vinogradov, Alexey Vinogradov, Stanislav Karchevsky, Pavel Ivchenko

**Affiliations:** 1 A.V. Topchiev Institute of Petrochemical Synthesis RAS, 29 Leninsky Pr., 119991 Moscow, Russia; amvvin@mail.ru (A.V.); vinasora@gmail.com (A.V.); phpasha1@yandex.ru (P.I.); 2 Chemistry Department, M.V. Lomonosov Moscow State University, 1 Leninskie Gory Str., Building 3, 119991 Moscow, Russia; 3 Joint-Stock Company “Institute of Petroleum Refining and Petrochemistry”, 12 Iniciativnaya Str., Ufa, 450065 Republic of Bashkortostan, Russia; st_karchevsky@mail.ru

**Keywords:** density functional theory, dimerization of α-olefins, polymerization, single-site catalysts, zirconocene

## Abstract

Zirconocene-mediated selective dimerization of α-olefins usually occurs when precatalyst (η^5^-C_5_H_5_)_2_ZrCl_2_ is activated by minimal excess of methylalumoxane (MAO). In this paper, we present the results of density functional theory (DFT) simulation of the initiation, propagation, and termination stages of dimerization and oligomerization of propylene within the framework of Zr-Al binuclear mechanism at M-06x/DGDZVP level of theory. The results of the analysis of the reaction profiles allow to explain experimental facts such as oligomerization of α-olefins at high MAO/(η^5^-C_5_H_5_)_2_ZrCl_2_ ratios and increase of the selectivity of dimerization in the presence of R_2_AlCl. The results of DFT simulations confirm the crucial role of the presence of chloride in the selectivity of dimerization. The molecular hydrogen was found in silico and proven experimentally as an effective agent that increases the rate and selectivity of dimerization.

## 1. Introduction

**Scheme 1:**
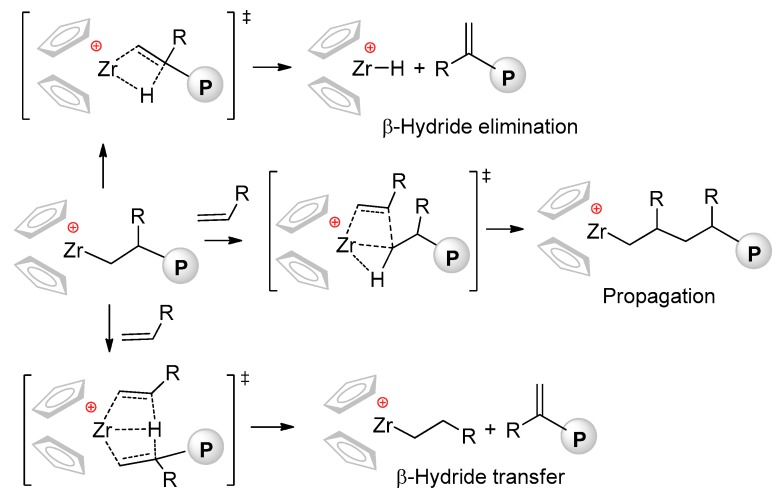
Traditional cationic mechanism of zirconocene-catalyzed polymerization of α-olefins.

Despite half-century of the fruitful study of zirconocene-catalyzed polymerization of α-olefins, some aspects of the reaction mechanism are unclear. One of the clouds obscuring the clear sky of Cosse-Arlman cationic coordination-insertion mechanism combined with few termination event pathways [1,2,3,4,5,6,7] (Scheme 1) is a highly selective dimerization of α-olefins with a formation of methylenealkanes [8,9,10,11,12,13].

**Scheme 2:**
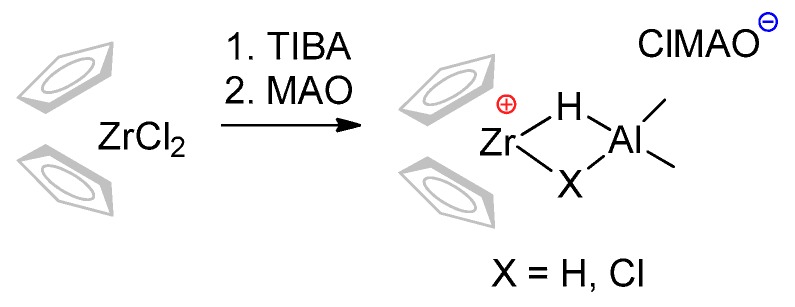
Cationic Zr-Al catalytic species proposed to explain the reactivity of zirconocenes under low MAO/Zr ratios [13,28].

Highly selective dimerization of α-olefins was detected when (η^5^-C_5_H_5_)_2_ZrCl_2_ [8,9,10] or Z(η^5^-C_5_H_4_)_2_ZrCl_2_ (Z-bridge between Cp rings) [13] have been activated by minimal excess of methylalumoxane (MAO). In the first publication of Bergman [9], the 1:1 Al/Zr ratio was used. In our experiments, we successfully applied two-stage activation of zirconocene dichlorides by the reaction with triisobutylaluminium (TIBA) and 5–10 equivalents of MAO [13]. Given that the reaction of LZrCl_2_ with TIBA results in the formation of Zr-Al hydride complexes [14,15,16,17], we proposed that the addition of minimal amounts of MAO results in the formation of cationic Zr-Al hydride species that catalyze dimerization of α-olefins. Relatively stable di-Zr complexes [18] could hardly be seen as prototype of catalytic species. Zr-Al complexes can vary in a number of coordinated Al atoms [15,19,20,21,22,23,24,25,26,27]. To explain experimental facts, we recently proposed a binuclear Zr-Al model (Scheme 2) [13,28] involving the catalytic species that are closely related to discrete ionic pairs containing Zr-(μ-Me)_2_AlMe_2_ fragments formed at the initial stage of zirconocene dichloride activation by MAO [29,30,31,32,33,34,35]. In References [13,28] we assumed that Zr-(μ-Cl)-Al species maintain selective dimerization, whereas Zr-(μ-H)-Al complexes catalyze the formation of higher oligomers.

In the present paper, we report the results of DFT modeling of initiation, propagation, and termination stages of α-olefin dimerization and oligomerization catalyzed by cationic (η^5^-C_5_H_5_)_2_Zr-Al species derived from R_2_AlX (R = Me, ^i^Bu; X = H, Cl, Me, Scheme 2) in comparison with traditional cationic mechanism.

## 2. Results

Propylene as an easiest α-olefin was chosen to minimize the time of calculations. For simulations of the reaction mechanisms, we used Gaussian-09 program package [36] at M-06x/DGDZVP [37,38] level of theory (for details, see Section 4.1). The results of the optimization of stationary points and transition states are provided in section S1 in the Supporting Information.

### 2.1. DFT Modeling of the Initiation Stage

**Scheme 3:**
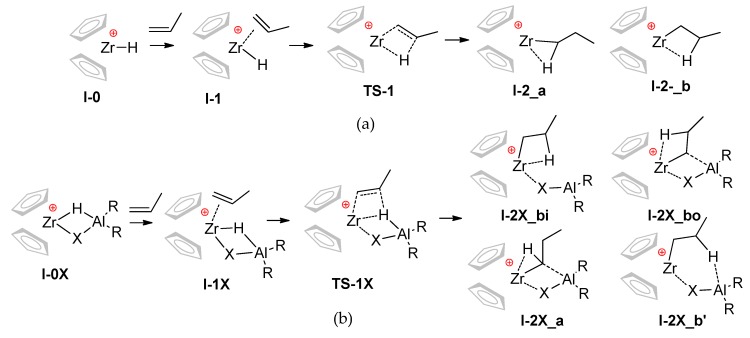
Stationary points and transition states of the initiation stage for (**a**) cationic and (**b**) binuclear mechanisms (X = H, Cl, and Me).

In our calculations, we considered [(η^5^-C_5_H_5_)_2_Zr-H]^+^ as a starting stationary point **I-0** for traditional cationic mechanism. The formation of π-complex **I-1** with propylene molecule was found to be highly exergonic (−17.5 kcal/mol), this complex easily transformed to [(η^5^-C_5_H_5_)_2_Zr–C_3_H_7_]^+^ via transition state **TS-1** with low activation barrier (2.1 kcal/mol). *n*-Propyl cationic complexes can be stabilized by additional agostic Zr-H bonding, the results of our calculations demonstrated that β-agostic complex **I-1_b** is 10.6 kcal/mol more stable than α-agostic complex **I-1_a** (Scheme 3).

The propensity of [(η^5^-C_5_H_5_)_2_Zr–H]^+^ to form stable adducts with R_2_Al–X (R = Me, ^i^Bu; X = H, Cl, Me) has been confirmed by considerable (more than 30 kcal/mol) lowering of the free energies in formation of the starting complexes **I-0X** by the formula [(η^5^-C_5_H_5_)_2_Zr(μ-H)(μ-X)AlR_2_]^+^. To compare the relative free energies and free enthalpies of the species that correspond to mononuclear and binuclear mechanisms, this negative change was accounted by the subtraction of G (R_2_AlX) and H (R_2_AlX) from the values of G and H calculated for Zr–XAlR_2_ species. The values of free energies and free enthalpies of stationary points and transition states for both mechanisms relative to G (**I-2_b**) and H (**I-2_b**) are presented in Table 1.

**Figure 1:**
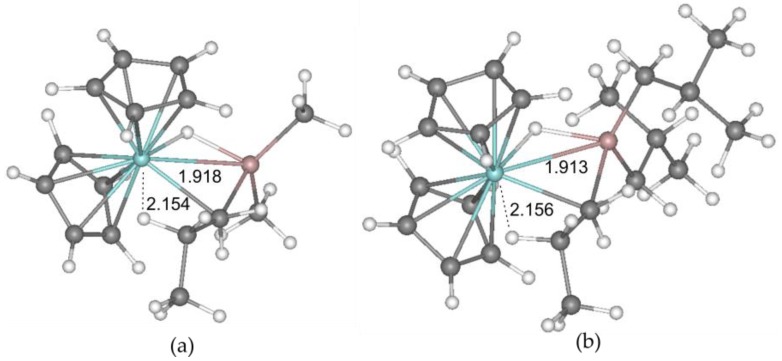
Calculated geometries of **I-2H-bo** complexes for (**a**) R = Me and (**b**) R = ^i^Bu. The distances Zr-H are specified (Å).

The coordination of propylene with a formation of **I-1X** is exergonic for X = H and endergonic for X = Cl and Me. The difference in free energies of the insertion transition state **TS-1X** and **I-1X** is minimal for X = Cl, but in general the value of the activation barrier of the formation of alkyl complexes **I-2X** is lower for X = H (Table 1). In the propyl cationic complexes **I-2X**, Zr-(μ-X)-Al coordination retains. Taking into consideration the possibility of the agostic Zr-H bonding, we calculated geometries and free energies of four types of **I-2X** and found that the most stable are agostic complexes **I-2X_a** and **I-2X_bo** (Scheme 3). The formation of β-agostic complexes by isobutyl fragment (for R = ^i^Bu) was also accounted and found to be insignificant (see section S1 the Supporting Information for details). The complexes **I-2H_bo** (Figure 1) are remarkably stable (−28.0 and −28.1 kcal/mol for R = Me and ^i^Bu, respectively) in comparison with other β-agostic complexes containing Zr-(μ-X)-Al fragments (X = Cl, Me). Higher stability of **I-2H_bo** with outside β-H coordination can be explained by the minima of steric distortions driven by Zr-(μ-X)-Al fragments for X = H. Extremely low free energies of **I-2H_bo** and closely related **I-4H_bo** (−39.7 and −42.5 kcal/mol for R = Me and ^i^Bu, respectively) are important factors that allow to explain the results of oligomerization experiments (see Section 2.3).

### 2.2. DFT Modeling of the Propagation Stage and Dimer Formation

**Scheme 4:**
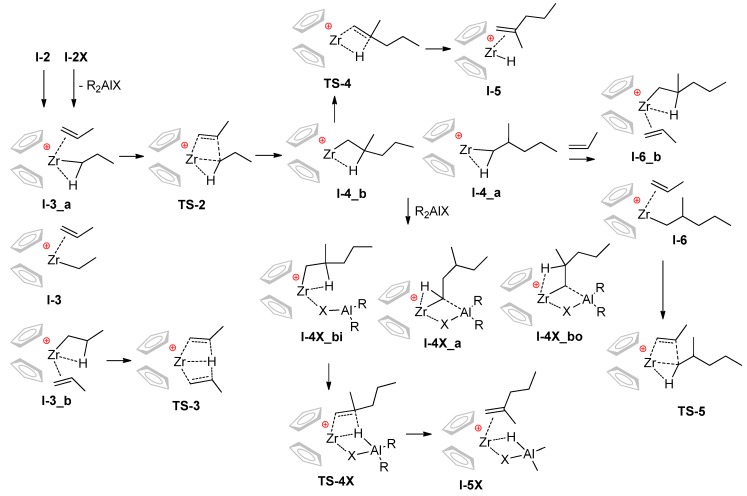
Stationary points and transition states of the propagation stages and termination events.

Stationary points and transition states for coordination/insertion of the second and third propylene molecules and for chain termination are presented in Scheme 4. For cationic mechanism, the coordination of the second propylene molecule was energetically favorable (−4.4 kcal/mol), the activation barrier of the second monomer insertion ΔG^≠^ = G(**TS-2**) − G(**I-3**) was 10.4 kcal/mol. The calculated activation barrier for chain release via transfer to monomer (**TS-3**, Scheme 4) was 17.1 kcal/mol. The most stable product of the second monomer insertion was β-agostic 2-methylpentyl zirconocene cation **I-4_b**. This complex can eliminate 2-methylpentene-1 via **TS-4** with a formation of **I-5** (ΔG^≠^ = 8.7 kcal/mol) or coordinate (**I-6**) and insert (**TS-5)** the third propylene molecule (ΔG^≠^ = 8.4 kcal/mol). Therefore, the chain propagation in the case of [(η^5^-C_5_H_5_)_2_Zr–C_6_H_13_]^+^ was found to be preferable, with the minimal difference in free energies between transition states of chain propagation and chain termination via β-hydride elimination (0.3 kcal/mol).

Within the framework of binuclear mechanism, we failed to find stationary points containing coordinated R_2_AlX and propylene molecules. Hence, the coordination of propylene resulted in loss of Zr-X bonding, and the stationary points **I-3** and **I-6** as well as transition states **TS-2**, **TS-3,** and **TS-5** had been common for mononuclear and binuclear mechanisms. The fundamental difference between mononuclear and binuclear mechanisms had arisen at the stage of the formation of 2-methylpentyl complexes **I-4X** followed by β-hydride elimination via **TS-4X** to π-complexes **I-5X**.

**Figure 2:**
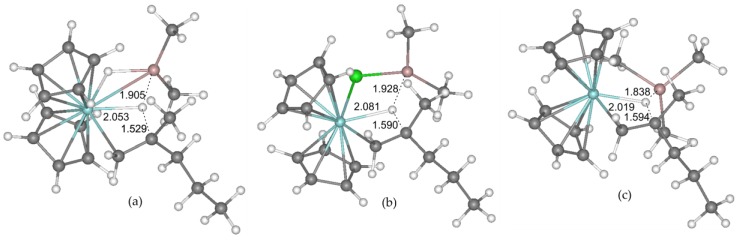
Calculated geometries of the transition states **TS-4X** (R = Me): (**a**) X = H; (**b**) X = F; (**c**) X = Cl; (**d**) X = Me. The distances Zr-H, Al–H and βC-H are specified (Å).

We optimized the geometries of **I-4X**, **TS-4X**, and **I-5X** for R = Me and ^i^Bu. The results of our calculations clearly demonstrated that β-agostic complexes **I-4X_bo** have lower free energies in comparison with isomeric β-agostic complexes **I-4X_bi** and α-agostic complexes **I-4X_a** (Scheme 4). The only exception was **I-4Cl_a** (R = Me) that was 1.2 kcal/mol more stable in comparison with **I-4X_bo**. The nature and geometries of the transition states **TS-4X** deserves separate consideration. In these transition states, Al atoms demonstrated cooperative effect (Figure 2). For R = Me, the values of the activation barriers ΔG^≠^ = G(**TS-4X**) − G(**I-4X**) were 17.7 (H), 13.6 (Cl), and 16.2 (Me) kcal/mol. Thus, β-hydride elimination had been the most affected by Me_2_AlCl coordination at Zr atom. This pattern was also manifested for R = ^i^Bu (Table 1).

The comparison of the relative free energies of chain termination transition states **TS-4X** and chain propagation transition state **TS-5** allowed us to make the conclusion that β-hydride elimination is energetically preferable for X = H and Cl. For X = Me, β-hydride elimination and dissociation of [(η^5^-C_5_H_5_)_2_Zr(C_6_H_13_)–AlMe_3_]^+^ with subsequent coordination-insertion of propylene molecule being equal. Apart from that, in the case of [(η^5^-C_5_H_5_)_2_Zr(C_6_H_13_)–AlMe^i^Bu_2_]^+^, the chain propagation was preferable (Table 1).

### 2.3. Dimerization and Oligomerization of 1-Hexene: Experimental Study

We performed a series of experiments using (η^5^-C_5_H_5_)_2_ZrCl_2_ as a precatalyst. In these experiments, (η^5^-C_5_H_5_)_2_ZrCl_2_ (0.1 mmol) was suspended in 200 mmol of 1-hexene and treated with TIBA solution (2 mmol) at 60 °C. After 10 min of stirring (no reaction detected), the calculated amount of MMAO-12 solution was added. The mixtures were analyzed by GC and ^1^H NMR spectroscopy after 1, 2, 3, and 4 h. The final results of these experiments are presented in Table 2. We found that after activation of (η^5^-C_5_H_5_)_2_ZrCl_2_ by 200 eq. MMAO-12 typical oligomerization proceeds (Table 2, run 1), the ratio of dimer, trimer, tetramer, and pentamer of 1-hexene can be interpreted by standard Flory distribution. The same results have been obtained by Kissin [39,40]. In the presence of 100 eq. MMAO-12 (Table 2, run 2), the relative rate of the dimer increased. In the following experiments, the rate of higher oligomers decreased with lowering of MMAO/Zr ratios (Table 2, runs 3–6, see Figure S2 in the Supporting Information). It should be noted that the rate of the reaction had a local minima at MMAO/Zr = 20 (Table 2, run 4).

The addition of R_2_AlCl resulted in an increase in the relative proportion of dimer. The best results were obtained when Me_2_AlCl was added (Table 2, run 7). The increasing of Et_2_AlCl/Zr ratio resulted in slowing down the reaction (Table 2, runs 8–11, Figure S3 in the Supporting Information). We also found that the addition of trimethylaluminium (Table 2, run 12) resulted in deceleration of the reaction with a formation of large amounts of higher oligomers. The activity of the catalyst and the selectivity of dimerization substantially increased in the atmosphere of the molecular hydrogen (Table 2, run 13; this issue is discussed below).

## 3. Discussion

**Figure 3:**
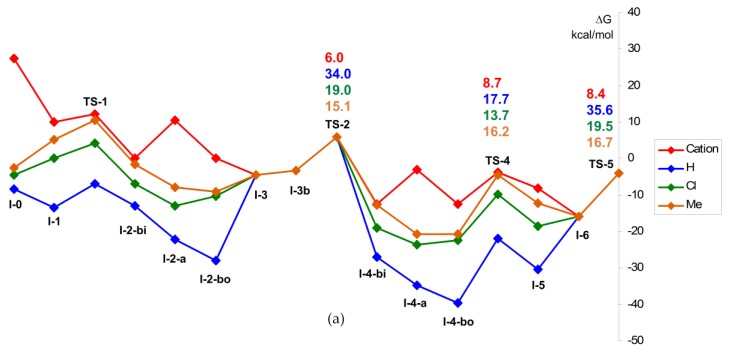

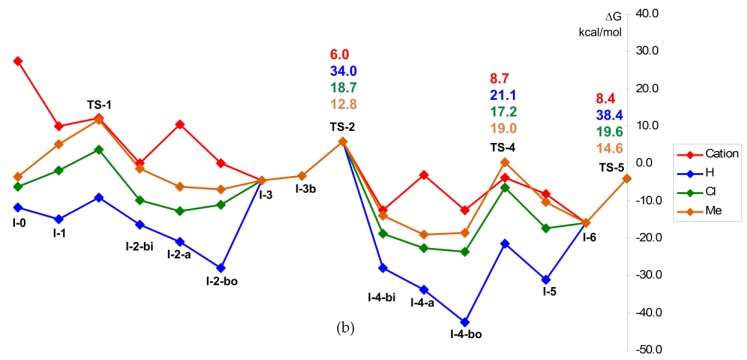
Reaction profiles for zirconocene-catalyzed oligomerization of propylene for mononuclear and binuclear reaction mechanisms for R = Me (**a**) and ^i^Bu (**b**). The free energies of stationary points and transition states are presented relative to **I-2_b**. The free activation energies for insertion of the second propylene molecule (via **TS-2**), β-hydride elimination with a formation of dimer (**TS-4**), and insertion of the third propylene molecule (**TS-5**) are presented in color.

Using the data presented in Table 1, we drawn free energy profiles for the oligomerization of propylene catalyzed by zirconocene cation and by (η^5^-C_5_H_5_)_2_Zr–XAlR_2_ cationic complexes for X = H, Cl, and Me. These profiles are presented in Figure 3a,b for R = Me and ^i^Bu, respectively. We calculated the free activation energies for two reaction pathways, namely, chain termination with a formation of 2-methylpentene-1 (vinylidene dimer of propylene) via **TS-4** and chain propagation via **TS-5**. These values were determined as a differences between G (**TS-4**) or G (**TS-5**) and free energies of the most stable intermediates **I-4**. The values of the free activation energies for insertion of the second monomer molecule **TS-2**, β-hydride elimination after this insertion **TS-4**, and insertion of the third monomer molecule **TS-5** are presented in Figure 3. It is the difference between **TS-4** or **TS-4X** and **TS-5** that determines the main direction of the reaction as a selective dimerization or oligomerization.

The first conclusion from the analysis of the reaction profiles (Figure 3) is the preference of β-hydride elimination after insertion of the second molecule of monomer in (η^5^-C_5_H_5_)_2_Zr-XAlR_2_ cationic complexes for the case of X = H and Cl. Within the framework of cationic mechanism and for X = Me, oligomerization and dimerization of propylene seem to be equal by the value of the activation barrier. This conclusion allows to explain higher oligomer content at higher MAO/Zr ratios (Table 1, runs 1 and 2) by the formation of the catalytic particles that are mononuclear cationic Zr complex or binuclear Zr complex with Me_3_Al. At high MAO/Zr ratios (100 and more) the large excess of MAO plays the role of “R_2_AlX sponge”, the ability of MAO to form stable complexes with Me_2_AlCl and Me_3_Al had been confirmed experimentally [41,42,43,44]. Note that the formation of oligomers was detected by Bergman when (η^5^-C_5_H_5_)_2_ZrMe_2_ was activated with 10 eq. of MAO instead of (η^5^-C_5_H_5_)_2_ZrCl_2_ [10].

When X = Cl, DFT modeling predicted the preferable formation of vinylidene dimers at low MAO/Zr ratios. In our experiments (Table 2, runs 7–9), we detected the highly positive effect of the addition of R_2_AlCl to the reaction mixture. Bergman [9,10] demonstrated that the presence of Cl is crucial for high selectivity of dimerization; recently the chemists of Idemitsu [45,46] and our laboratory [13,47] applied the addition of R_2_AlCl to reach maximum yields of α-olefin dimers.

**Figure 4:**
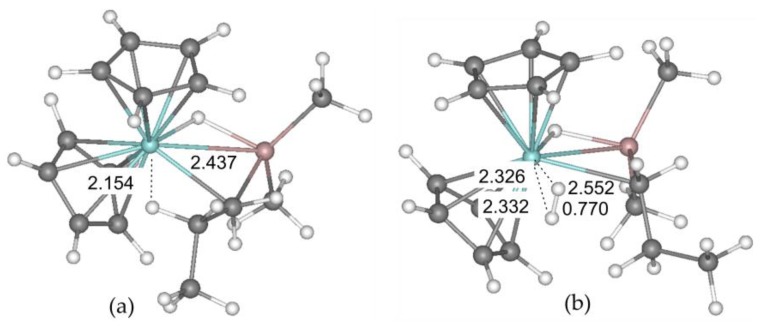
Calculated geometries of **I-2H_bo** (**a**) and dihydrogen complex **I-2H_H2** (**b**). The distances Zr–H, Zr–C(Alkyl), and H–H are specified (Å).

Our calculations predicted that hydride complex **I-2H_bo** is ~17 kcal/mol more stable than **I-2Cl_bo** and can, therefore, be considered as a typical “dormant site” for the binuclear mechanism. Evidently, the formation of alkyl-hydride Zr-Al complexes is more than likely under the reaction conditions used in our experiments. Moreover, these inactive species can make up the most of Zr-containing particles. Keeping in mind that the metal-hydride agostic interaction makes the main contribution to the stabilization of **I-2H_bo**, we proposed that the molecular hydrogen can activate this dormant site by the cleavage of agostic Zr-βH bonding. We made optimizations of different structures formed from **I-2H_bo** and H_2_ molecule and found a novel stationary point **I-2H_H2** (Figure 4). In this complex, the agostic bonding Zr-H is absent that potentially facilitates the formation of stationary points and transition states of the propagation stage which is completed by selective formation of the dimer when X = H.

In complex **I-2H_H2**, the molecule of H_2_ plays the role of the additional activator. The interatomic distance d(H-H) in **I-2H_H2** was only 0.1 Å higher than in hydrogen molecule. This complex may be stabilized with a formation of α-agostic complex **I-2H_a_H2** (see S1.9 in the Supporting Information). The free energy of this complex is 12.2 kcal/mol higher than **I-2H_bo**. It is evident that the similar complex can be formed from **I-4H_bo**. Apparently, the molecular hydrogen can activate the “dormant” β-agostic hydride complexes with an increase in selectivity of the dimer formation. To confirm this assumption, we performed the catalytic experiment at 1 bar of H_2_ (Table 2, run 13) and detected 99% conversion of the monomer with 89% dimer selectivity without the formation of saturated hydrocarbons. This experiment has clearly demonstrated the role of the molecular hydrogen as an activator, but not as a reactant.

## 4. Materials and Methods

### 4.1. DFT Calculations

The initial cartesian coordinates of the stationary points had been found by PRIRODA program (version 4.0, M.V. Lomonosov Moscow University, Moscow, Russia) [48] using the 3ζ basis. The final optimization and determination of the thermodynamic parameters for stationary points and transition states were carried out using Gaussian 09 program [36] for gas phase at 298.15 K, the root mean square (RMS) force criterion was 3 × 10^−4^. The M-06x functional [37] and DGDZVP basis set [38,49] were used in the optimizations. As was demonstrated earlier, M-06x functional is one of the most correct functionals for calculations of the free energies in DFT modeling of zirconocene-catalyzed reactions [50]. Transition states were found by energy scanning with sequential changing of key geometric parameters with a step of 0.01 Å followed by Berny optimization and confirmed by intrinsic reaction coordinate (IRC) simulations (see Figure S1 and find *_IRC.gif animation files in the Supporting Information).

### 4.2. General Experimentsl Remarks

TIBA (1 M solution in hexane, Merck, Darmstadt, Germany), MMAO-12 (1.52 M solution in toluene, Merck), (η^5^-C_5_H_5_)_2_ZrCl_2_ (Merck), and CDCl_3_ (99.8% ^2^H, Cambridge Isotope Laboratories, Inc., MS, USA) were used as purchased. 1-Hexene (Merck) was stored over Na wire and distilled under argon. The ^1^H NMR spectra were recorded on a Bruker AVANCE 400 spectrometer (400 MHz, Bruker, MS, USA) at 20 °C. The chemical shifts are reported in ppm relative to the solvent residual peaks. The distribution of oligomers produced in zirconocene-catalyzed reactions was measured by gas chromatography (GC) method. GC analysis was carried out with a KRISTALL-2000M gas chromatograph (Meta-chrom Ltd., Yoshkar-Ola, Russian Federation) equipped with a SolGel-1ms (60 m × 0.25 mm × 0.25 μm) column and a flame ionization detector. Helium was used as a carrier gas at a rate of 1.364 cc/min and with a split ratio of 73.3:1. The injection temperature was 320 °C, and the column temperature was 200 °C within 5 min and then increased from 200 °C to 300 °C at a rate of 10 °C/min.

### 4.3. Dimerization and Oligomerization Experiments

1-Hexene (25 mL, 200 mmol) and TIBA (2 mL of 1 M solution in hexane, 20 mmol) were mixed in a two-necked flask prefilled with argon, which was then placed in a thermostated bath with diethylene glycol. After maintaining the external bath at 60 °C for 2 min, zirconocene precatalyst (0.1 mmol) was added to the flask. After 20 min of stirring, given amounts of MMAO-12 (1.52 M solution in toluene) and R_2_AlCl (if applied, 1 M solutions in hexane) were added. The reaction probes were analyzed by NMR and GC after 1, 2, 3, and 4 h. After 4 h of the reaction, 1 mL of methanol and 1 mL of water were added, the organic phase was separated, and the residue was extracted with hexane. The combined organic phases were evaporated under reduced pressure, the residue was stirred at 250 °C (0.1 Torr) to remove C12–C30 reaction products and to determine the weight of the higher oligomers.

## 5. Conclusions

In the present paper, we report the results of DFT modeling of the binuclear mechanism of zirconocene-catalyzed oligomerization of α-olefins (by the case of propylene) that consider the catalytic particles as a cationic species with coordinated R_2_AlX fragments (X = H, F, Cl, Me). We have proposed this mechanism earlier [13,28] for specific reaction conditions, when starting zirconocene dichloride precatalyst has been activated by minimal excess of MAO (up to 10 equivalents). The comparison of the reaction profiles for traditional mononuclear [(η^5^-C_5_H_5_)_2_Zr-Alkyl]^+^ and binuclear [(η^5^-C_5_H_5_)_2_Zr-Alkyl(R_2_AlX)]^+^ species demonstrated the qualitative difference between mononuclear and binuclear mechanisms. Without R_2_AlX coordination, oligomerization is a preferable reaction pathway. If X = H, highly stable β-agostic complexes **I-2X_bo** form, and the reactions slow down. If X = Cl, the formation of vinylidene dimers becomes a main direction of the reaction. The transition states of β-hydride elimination **TS-4X** (X = H, Cl) demonstrate explicit Zr-Al cooperative effect. For X = Me, there is no significant assistance for β-hydride elimination. These results of DFT modeling correlate with the results obtained in the experimental study of 1-hexene oligomerization.

We also found that molecular hydrogen under “low MAO” conditions demonstrates the chemical behavior that is not typical for Ziegler-Natta processes. In the presence of hydrogen, the dimerization accelerates with substantial increase of selectivity, without the formation of the products of hydrogenolysis. We used the “molecular hydrogen” probe in DFT calculations to find and visualize the mechanism of this effect and found that the complex **I-2H_bo** can react with H_2_ without cleavage of the H–H bond but with the complete loss of β-agostic coordination. Thus, H_2_ acts as an additional activator for hydride complex that grows as an active and selective dimerization catalyst.

Obviously, the mechanistic concept proposed and presented in our paper is approximate and something speculative. However, we believe that Zr-Al binuclear mechanistic concept, which takes into account the direct impact of R_2_AlX in catalytic process, will be a valuable addition to traditional mechanism of single-site polymerization of α-olefins. The simplicity of the model is attractive to compare in silico the reactivity of zirconium complexes with different ligand environment under industrially important “low MAO” conditions and for the design of prospective zirconocene oligomerization and polymerization catalysts. An extensive experimental and theoretical work in this field is underway in our laboratory.

## Figures and Tables

**Table 1:** Calculated free energies and free enthalpies (kcal/mol) of stationary points and transition states for mononuclear and binuclear reaction mechanisms. ^1^

Structure		Cationic Mechanism	R = Me			R = ^i^Bu		
			X=H	X=Cl	X=Me	X=H	X=Cl	X=Me
**I-0** **I-0X**	G	27.5	−8.4	−4.6	−2.7	−11.8	−6.3	−3.5
	H	40.8	−9.9	−6.7	−2.6	−11.2	−8.6	−6.6
**I-1** **I-1X**	G	10.0	−13.5	0.0	5.1	−14.9	−1.8	5.3
	H	9.8	−26.7	−14.0	−1.6	−29.7	−17.3	−12.2
**TS-1** **TS-1X**	G	12.1	−7.0	4.3	10.6	−9.2	3.7	11.8
	H	11.4	−21.9	−10.9	−4.1	−25.2	−12.8	−6.9
**I-2_b** **I-2X_bi**	G	0.0	−13.1	−7.0	−1.6	--16.3	−9.8	−1.4
	H	0.0	−26.9	−19.5	−11.6	−28.9	--23.0	−15.2
**I-2+_a** **I-2X_a**	G	10.6	−22.1	−13.0	−7.8	−21.0	−12.7	−6.1
	H	10.6	−35.9	−28.4	−22.6	−36.7	−29.3	−24.7
**I-2_b** **I-2X_bo**	G	0.0	−28.0	−10.4	−9.1	−28.1	−11.0	−6.8
	H	0.0	−41.9	−26.9	−24.0	−43.7	−28.8	−26.7
**I-3**	G	−4.4						
	H	−16.3						
**I-3_b**	G	−3.2						
	H	−15.8						
**TS-2**	G	6.0						
	H	−9.3						
**TS-3**	G	12.7	12.7	12.7	12.7	12.7	12.7	12.7
	H	−1.9	−1.9	−1.9	−1.9	−1.9	−1.9	−1.9
**I-4_b** **I-4X_bi**	G	−12.5	−27.1	−19.1	−12.7	−28.0	−22.4	−13.8
	H	−26.4	−52.0	−45.9	−38.0	−55.1	−49.8	−43.3
**I-4_a** **I-4X_a**	G	−3.0	−34.8	−23.6	−20.7	−33.9	−22.8	−19.0
	H	−15.7	−61.9	−51.7	−48.2	−62.8	−53.8	−50.6
**I-4_b** **I-4X_bo**	G	−12.5	−39.7	−22.4	−20.8	−42.5	−23.7	−18.7
	H	−26.4	−68.3	−51.8	−49.1	−71.0	−53.9	−51.6
**TS-4** **TS-4X**	G	−3.8	−22.0	−9.9	−4.6	−21.4	−6.5	0.3
	H	−17.9	−50.5	−38.9	−32.1	−51.6	−38.3	−33.2
**I-5** **I-5X**	G	−8.2	−30.5	−18.6	−12.3	−31.2	−17.4	−10.4
	H	−19.7	−56.6	−45.0	−37.7	−59.3	−45.6	−40.2
**I-6**	G	−15.7						
	H	−41.2						
**I-6_b**	G	−16.0						
	H	−41.3						
**TS-5**	G	−4.1						
	H	−32.5						

^1^ Relative to **I-2_b**.

**Table 2:** Zirconocene-catalyzed oligomerization of 1-hexene (in bulk, 60 °C, 4 h). ^1^

Run	MMAO-12 eq.	R_2_AlX eq.	1-Hexene Conv., %	Product Distribution (wt.%) ^2^					
				2-C6	C12	C18	C24	C30	Higher Oligomers
1	200	0	99	1.7	53.8	13.2	5.3	3.2	21.7
2	100	0	98	1.9	69.5	7.5	3.8	2.4	14.1
3	50	0	96	2.0	75.1	6.9	3.1	1.6	7.7
4	20	0	93	2.0	77.8	5.2	2.3	1.1	4.8
5	10	0	97	3.4	82.3	5.9	1.5	0.3	3.6
6	5	0	95	3.8	81.6	5.4	1.3	0.3	2.6
7	10	Me_2_AlCl, 1	98	3.6	87.4	3.2	1.1	0.3	2.5
8	10	Et_2_AlCl, 1	88	3.6	78.1	2.9	1.0	0.3	2.2
9	10	Et_2_AlCl, 2	97	3.8	85.7	3.3	1.2	0.4	2.7
10	10	Et_2_AlCl, 5	59	2.0	53.2	1.1	0.3	<0.1	<0.2
11	10	Et_2_AlCl, 10	31	0.6	29.1	0.3	<0.1	<0.1	<0.2
12	10	Me_3_Al, 2	61	0.9	36.6	9.6	3.3	1.4	9.2
13 ^3^	10	10	99	2.8	88.8	3.2	1.4	0.6	2.1

^1^ Activation by 20 eq. of TIBA for 10 min at 60 °C followed by the addition of given amount of MMAO-12. ^2^ The data were obtained by GC, the rate of higher oligomers was determined by the weighting of the residue after stirring the treated reaction mixture at 250 °C and 0.1 Torr. ^3^ Oligomerization at 1 bar of the molecular hydrogen.

## Supplementary Materials

The following are available online. DFT calculations data: plots of the molecular geometries, energies, and cartesian coordinates for all stationary points and transition states; Figure S1: The dependence of the composition of the reaction mixtures (1-hexene oligomerization in bulk, 60 °C, 4 h) from Al_MMAO-12_/Zr ratio; Figure S2: The effect of R_3_AlCl on the composition of the reaction mixtures (1-hexene oligomerization in bulk, 60 °C, 4 h, Al_MMAO-12_/Zr = 10), in pdf. format. 19 animation files for all transition states and 3 animation files demonstrating the IRC procedure (in gif. format).
