# Supplementary material for: Zirconocene-Catalyzed Dimerization of α-Olefins: DFT Modeling of the Zr-Al Binuclear Reaction Mechanism
Source: Molecules. 2019 Oct 2;24(19):3565. doi: 10.3390/molecules24193565 (PMC6803839; doi:10.3390/molecules24193565)
Supplement: Supplementary file 1 [file molecules-24-03565-s001.zip › IN_Zr_DFT__SI_rev.pdf]

Zirconocene-catalyzed dimerization of α-olefins: DFT modeling of the Zr-Al binuclear reaction mechanism

Ilya Nifant'ev [1,2,*], Alexander Vinogradov [1], Alexey Vinogradov [1], Stanislav Karchevsky [3] and Pavel Ivchenko [1,2]

[1] A.V. Topchiev Institute of Petrochemical Synthesis RAS, 29 Leninsky Pr., Moscow, Russia 119991; ilnif@yahoo.com (I.N.), amvvin@mail.ru (Alexander V.), vinasora@gmail.com (Alexey V.), phpasha1@yandex.ru (P.I.)

[2] Chemistry Department, M.V. Lomonosov Moscow State University, 1 Leninskie Gory Str., Building 3, Moscow, Russia 119991; inif@org.chem.msu.ru (I.N.), inpv@org.chem.msu.ru (P.I.)

[3] Joint-stock company "Institute of petroleum refining and petrochemistry", 12 Iniciativnaya Str., Ufa, Republic of Bashkortostan, Russia 450065; st_karchevsky@mail.ru (S.K.)

* Correspondence: ilnif@yahoo.com; Tel.: +7-495-939-4098 (I.N.)

SUPPORTING INFORMATION

S1. DFT calculations 2

S1.1. Olefins and organoaluminium compounds 2

S1.2. Reaction profile without Zr–Al coordination 10

S1.3. Me_2AlH reaction profile 28

S1.4. Me_2AlCl reaction profile 41

S1.5. Me_3Al reaction profile 54

S1.6. $^iBu_2AlH$ reaction profile 67

S1.7. $^iBu_2AlCl$ reaction profile 84

S1.8. $^iBu_2AlMe$ reaction profile 101

S1.9. Interaction with H_2 119

S1.10. Transition states search and proof (IRC) 121

S2. Oligomerization experiments 122

S1. DFT calculations data: molecular structures, energies and cartesian coordinates

S1.1. Olefins and organoaluminium compounds

C3 Propylene

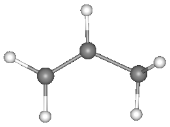

Zero-point vibrational energy	211398.7 (Joules/Mol)
	50.52549 (Kcal/Mol)
Zero-point correction	0.080517 (Hartree/Particle)
Thermal correction to Energy	0.084591
Thermal correction to Enthalpy	0.085535
Thermal correction to Gibbs Free Energy	0.055526
Sum of electronic and zero-point Energies	-117.762918
Sum of electronic and Thermal Energies	-117.758845
Sum of electronic and Thermal Enthalpies	-117.757900
Sum of electronic and Thermal Free Energies	-117.787910

cartesian

1	-0.27033332	1.58850002	0.00021111	1	1.70376670	0.18960002	-0.88008893
6	-0.23303331	0.49990001	0.00011111	1	1.70386672	0.18940002	0.88001108
6	-1.37853336	-0.18249999	0.00001111	1	-1.38873339	-1.27020001	-0.00008889
6	1.13336670	-0.12299998	-0.00008889	1	-2.34013319	0.32170001	0.00011111
1	1.06976664	-1.21340001	-0.00018889				

C6 2-methylpent-1-ene

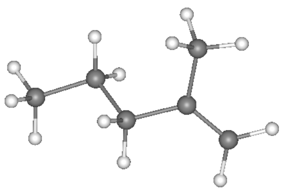

Zero-point vibrational energy	437755.2 (Joules/Mol)
	104.62601 (Kcal/Mol)
Zero-point correction	0.166732 (Hartree/Particle)
Thermal correction to Energy	0.174743
Thermal correction to Enthalpy	0.175687
Thermal correction to Gibbs Free Energy	0.133949

Sum of electronic and zero-point Energies	-235.567243
Sum of electronic and Thermal Energies	-235.559233
Sum of electronic and Thermal Enthalpies	-235.558288
Sum of electronic and Thermal Free Energies	-235.600026

cartesian

6	2.11586118	-1.19247782	0.22433333	6	-1.17973888	0.21752222	0.40563333
6	1.28416109	-0.19617778	-0.09466667	1	-0.29373884	-0.08927779	-1.53176665
6	1.72116113	1.24642217	-0.05856667	1	-0.32783884	-1.53517783	-0.52496666
6	-0.14513884	-0.45527780	-0.50626665	6	-2.61013889	-0.14387779	0.00683333
1	1.79176116	-2.22987771	0.20183334	1	-1.05713880	1.30512214	0.36623335
1	3.14576125	-1.00127780	0.51553333	1	-0.99213886	-0.08297779	1.44253337
1	1.46976113	1.75102222	-0.99796665	1	-3.34213877	0.34442219	0.65543336
1	1.21206117	1.79022217	0.74383336	1	-2.77293897	-1.22397780	0.07083333
1	2.79746103	1.33322215	0.10443333	1	-2.81703877	0.16242221	-1.02326667

Me_2AlH

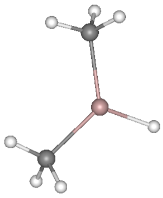

Zero-point vibrational energy	206187.7 (Joules/Mol)
	49.28004 (Kcal/Mol)
Zero-point correction	0.078533 (Hartree/Particle)
Thermal correction to Energy	0.084839
Thermal correction to Enthalpy	0.085783
Thermal correction to Gibbs Free Energy	0.049544
Sum of electronic and zero-point Energies	-322.675915
Sum of electronic and Thermal Energies	-322.669609
Sum of electronic and Thermal Enthalpies	-322.668665
Sum of electronic and Thermal Free Energies	-322.704904

cartesian

13	0.00000995	0.69409001	0.00005000	1	-2.31759000	0.04249001	-0.87424999
6	-1.71979010	-0.24191001	0.00005000	1	2.31751013	0.04159001	-0.87465000
6	1.71980989	-0.24191001	0.00005000	1	2.31810999	0.04289000	0.87395000
1	-1.62759006	-1.33160996	-0.00025000	1	1.62750995	-1.33160996	0.00085000
1	-2.31788993	0.04209000	0.87424999	1	-0.00009005	2.28389001	-0.00005000

Me_2AlF

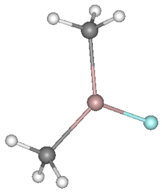

Zero-point vibrational energy	195987.5 (Joules/Mol)
	46.84214 (Kcal/Mol)
Zero-point correction	0.074648 (Hartree/Particle)
Thermal correction to Energy	0.081673
Thermal correction to Enthalpy	0.082617
Thermal correction to Gibbs Free Energy	0.043727
Sum of electronic and zero-point Energies	-422.008060
Sum of electronic and Thermal Energies	-422.001034
Sum of electronic and Thermal Enthalpies	-422.000090
Sum of electronic and Thermal Free Energies	-422.038981

cartesian

9	-0.00026001	2.27294993	-0.00003000	1	-2.33186007	0.07445005	-0.87582999
13	-0.00006001	0.58705002	-0.00003000	1	-1.71665990	-1.33574986	-0.00043000
6	-1.75565994	-0.24314994	-0.00003000	1	2.33153987	0.07435006	0.87617004
6	1.75574005	-0.24284995	-0.00003000	1	1.71684003	-1.33544993	-0.00013000
1	-2.33156013	0.07385004	0.87617004	1	2.33193994	0.07455003	-0.87582999

Me_2AlCl

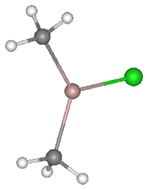

Zero-point vibrational energy	193594.6 (Joules/Mol)
	46.27022 (Kcal/Mol)
Zero-point correction	0.073736 (Hartree/Particle)
Thermal correction to Energy	0.081041
Thermal correction to Enthalpy	0.081985
Thermal correction to Gibbs Free Energy	0.041705
Sum of electronic and zero-point Energies	-782.313017
Sum of electronic and Thermal Energies	-782.305712
Sum of electronic and Thermal Enthalpies	-782.304768
Sum of electronic and Thermal Free Energies	-782.345048

cartesian

17	-2.68446016	-0.00012997	0.00012000	1	-0.02626008	-2.33113003	0.87361997
13	-0.55946004	-0.00002997	0.00052000	1	-0.01906008	-2.32693005	-0.87867999
6	0.28773999	-1.74942994	0.00012000	1	-0.01836008	2.32646990	-0.87927997
6	0.28753996	1.74947011	0.00022000	1	-0.02736008	2.33156991	0.87301999
1	1.37994003	-1.69572997	0.00462000	1	1.37974000	1.69587004	0.00572000

Me_3Al

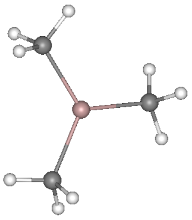

Zero-point vibrational energy	281420.0 (Joules/Mol)
	67.26100 (Kcal/Mol)
Zero-point correction	0.107187 (Hartree/Particle)
Thermal correction to Energy	0.114008
Thermal correction to Enthalpy	0.114952
Thermal correction to Gibbs Free Energy	0.076672
Sum of electronic and zero-point Energies	-361.960633
Sum of electronic and Thermal Energies	-361.953812
Sum of electronic and Thermal Enthalpies	-361.952868
Sum of electronic and Thermal Free Energies	-361.991148

cartesian

13	0.00673848	-0.00203843	0.01049999	1	-1.73296154	1.79556155	0.64690000
6	1.96193850	-0.15483843	0.00679999	1	-0.17346153	2.57096148	0.32240000
6	-0.84406149	1.76556158	0.00699999	6	-1.11116147	-1.61333847	-0.00300001
1	2.43583846	0.58226156	0.66439998	1	-1.29386151	-1.95633841	1.02420008
1	2.31593847	-1.14763844	0.30170000	1	-0.63736153	-2.45083833	-0.52580005
1	2.35833836	0.03676157	-0.99900001	1	-2.09526157	-1.44513845	-0.45260000
1	-1.19066155	2.01906157	-1.00349998				

$^{i}Bu_2AlH$

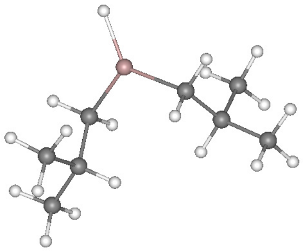

Zero-point vibrational energy	660384.6 (Joules/Mol)
	157.83571 (Kcal/Mol)
Zero-point correction	0.251527 (Hartree/Particle)
Thermal correction to Energy	0.265239
Thermal correction to Enthalpy	0.266183
Thermal correction to Gibbs Free Energy	0.210572
Sum of electronic and zero-point Energies	-558.270078
Sum of electronic and Thermal Energies	-558.256366
Sum of electronic and Thermal Enthalpies	-558.255422
Sum of electronic and Thermal Free Energies	-558.311033

cartesian

13	-0.00905002	1.78803933	0.04104284	1	3.35724998	0.96273929	1.08924282
6	1.42435002	0.79663926	-0.87575716	1	3.43794990	-1.97056055	-0.11415716
6	-1.40014994	0.71213931	0.92934281	1	3.90965009	-0.50746071	-0.99635720
1	1.03775001	0.36833930	-1.81295717	1	2.66144991	-1.57246065	-1.65705717
6	1.99284995	-0.33396071	0.00484284	6	-3.05664992	-1.20196068	0.65544283
1	2.24175000	1.46893930	-1.17325723	1	-1.18355000	-1.00696063	-0.36185715
6	-1.99205005	-0.34066069	-0.03015716	6	-2.57975006	0.33543932	-1.27325714
1	-2.21134996	1.35123932	1.30594277	1	-3.02935004	-0.38956070	-1.95875716
1	-0.98374999	0.20463930	1.81224287	1	-1.81464994	0.88173926	-1.84415722
6	2.56664991	0.23533930	1.30634284	1	-3.35505009	1.05503929	-0.98575717
6	3.06084991	-1.14796066	-0.73135716	1	-3.45315003	-1.96756077	-0.02015716
1	1.17375004	-1.01656067	0.27134284	1	-3.89385009	-0.57706070	0.98654282
1	2.99394989	-0.54726070	1.94084287	1	-2.64615011	-1.70436060	1.53634286
1	1.79834998	0.74753928	1.90314281	1	-0.04805002	3.37653923	0.05234284

$^{i}Bu_2AlF$

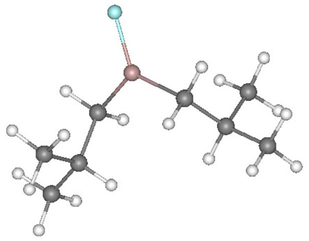

Zero-point vibrational energy	650028.3 (Joules/Mol)
	155.36048 (Kcal/Mol)
Zero-point correction	0.247583 (Hartree/Particle)
Thermal correction to Energy	0.262147
Thermal correction to Enthalpy	0.263092
Thermal correction to Gibbs Free Energy	0.204262
Sum of electronic and zero-point Energies	-657.602812

Sum of electronic and thermal Energies	-657.588247
Sum of electronic and thermal Enthalpies	-657.587303
Sum of electronic and thermal Free Energies	-657.646132

cartesian

13	-0.00083931	1.62581789	0.00134998	1	-3.27653933	1.03181791	-1.18494999
6	-1.48273921	0.71481788	0.88824999	1	-3.69283938	-1.86618221	0.04264998
6	1.48306072	0.71841788	-0.88605005	1	-4.07253933	-0.35048211	0.87914997
1	-1.15173924	0.26341787	1.83404994	1	-2.96673918	-1.51538217	1.62144995
6	-2.09923935	-0.37168211	-0.01665002	6	3.27536082	-1.06898212	-0.67275000
1	-2.25613928	1.44801784	1.15674996	1	1.33286071	-1.12918210	0.22544998
6	2.09946084	-0.37058210	0.01594998	6	2.54836082	0.22761787	1.35294998
1	2.25616074	1.45291781	-1.15155005	1	3.01356077	-0.52248210	1.99934995
1	1.15326071	0.26981789	-1.83354998	1	1.70636070	0.65481788	1.91715002
6	-2.54833937	0.23021787	-1.35195005	1	3.27666068	1.02961779	1.18814993
6	-3.27503920	-1.07208216	0.67025000	1	3.69326067	-1.86468208	-0.04735002
1	-1.33253932	-1.12968218	-0.22835001	1	4.07286072	-0.34658211	-0.87975001
1	-3.01363921	-0.51818210	-2.00035024	1	2.96716070	-1.50978208	-1.62514997
1	-1.70643926	0.65871787	-1.91534996	9	-0.00303931	3.30991793	0.00084998

$^{i}Bu_2AlCl$

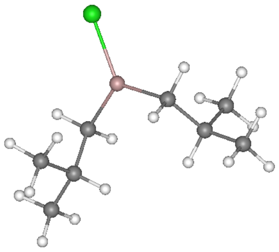

Zero-point vibrational energy	647950.8 (Joules/Mol)
	154.86396 (Kcal/Mol)
Zero-point correction	0.246791 (Hartree/Particle)
Thermal correction to Energy	0.261656
Thermal correction to Enthalpy	0.262600
Thermal correction to Gibbs Free Energy	0.202152
Sum of electronic and zero-point Energies	-1017.907594
Sum of electronic and thermal Energies	-1017.892730
Sum of electronic and thermal Enthalpies	-1017.891785
Sum of electronic and thermal Free Energies	-1017.952233

cartesian

13	-0.06489286	1.68887162	0.03645718	1	-3.32349300	0.89927155	-1.14404285

6	-1.48939288	0.67107153	0.90525717	1	-3.49249291	-2.05762839	0.00965718
6	1.46210718	0.84097153	-0.84174281	1	-3.98659301	-0.60142845	0.89065719
1	-1.12339282	0.22377151	1.84025717	1	-2.78029299	-1.68992829	1.59075713
6	-2.02699280	-0.43702847	-0.02354282	6	3.21220708	-0.99402851	-0.72484285
1	-2.31259298	1.34007156	1.19085717	1	1.27090716	-1.05602837	0.17645717
6	2.05410695	-0.30512851	0.00285718	6	2.51980710	0.21337152	1.36685717
1	2.24210715	1.58667159	-1.04854286	1	2.96510696	-0.58172852	1.97245717
1	1.15020716	0.45337152	-1.82264280	1	1.69090712	0.63567156	1.95235717
6	-2.53739285	0.16107154	-1.33854282	1	3.27080703	1.00127149	1.24025714
6	-3.13449287	-1.24852848	0.65475720	1	3.61580706	-1.82712841	-0.13994282
1	-1.20429277	-1.12512851	-0.26314282	1	4.02360725	-0.27942842	-0.90194285
1	-2.95069289	-0.60562843	-2.00064278	1	2.89050698	-1.38312840	-1.69504285
1	-1.73899281	0.66847152	-1.89944279	17	-0.20219284	3.80797148	0.01415718

$^{i}Bu_2AlMe$

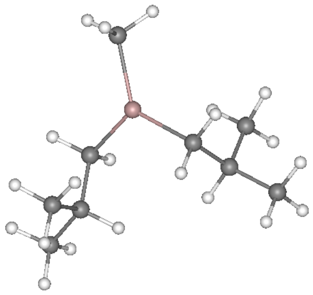

Zero-point vibrational energy	737304.7 (Joules/Mol)
	176.22006 (Kcal/Mol)
Zero-point correction	0.280825 (Hartree/Particle)
Thermal correction to Energy	0.296627
Thermal correction to Enthalpy	0.297571
Thermal correction to Gibbs Free Energy	0.235473
Sum of electronic and zero-point Energies	-597.554227
Sum of electronic and thermal Energies	-597.538424
Sum of electronic and thermal Enthalpies	-597.537480
Sum of electronic and thermal Free Energies	-597.599578

cartesian

13	0.05293867	1.37268722	-0.06253873	1	3.82563877	-1.15381277	-0.97243875
6	1.35613859	0.18758714	-0.95083874	1	2.54563880	-2.27741289	-1.45113873
6	-1.45526135	0.47948712	0.84056127	6	-3.17076135	-1.39791286	0.77626127
1	0.89103872	-0.36101288	-1.78273869	1	-1.25826132	-1.40891278	-0.18803872
6	1.94393861	-0.82491285	0.05426127	6	-2.57526135	-0.16751285	-1.32983863
1	2.17963886	0.76628715	-1.39473867	1	-3.02166128	-0.97101289	-1.92413867

6	-2.05246115	-0.67751288	0.01676127	1	-1.77416134	0.27608714	-1.93713868
1	-2.25116134	1.20058715	1.07966125	1	-3.34116125	0.60148716	-1.17553866
1	-1.11336136	0.09148714	1.81306136	1	-3.57396126	-2.23901272	0.20206128
6	2.56573868	-0.09781286	1.25176132	1	-3.99346113	-0.70381290	0.98286122
6	2.97973871	-1.74331284	-0.60073876	1	-2.80936122	-1.78201282	1.73476136
1	1.12683868	-1.45481277	0.43276128	6	0.29583865	3.31778717	-0.04273873
1	3.02243876	-0.79481286	1.96136129	1	-0.39176133	3.83878708	0.63046128
1	1.81953859	0.48318714	1.81386125	1	0.14733867	3.73778725	-1.04583871
1	3.34213877	0.59928715	0.91536123	1	1.31843865	3.58768725	0.24706128
1	3.36903882	-2.48461270	0.10556127				

S1.2. Reaction profile without Zr–Al coordination

I-0

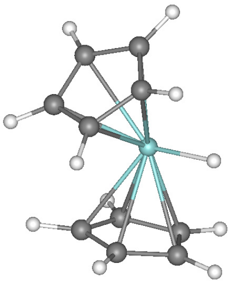

Zero-point vibrational energy	463865.2 (Joules/Mol)
	110.86645 (Kcal/Mol)
Zero-point correction	0.176677 (Hartree/Particle)
Thermal correction to Energy	0.186380
Thermal correction to Enthalpy	0.187324
Thermal correction to Gibbs Free Energy	0.139040
Sum of electronic and zero-point Energies	-3927.913963
Sum of electronic and thermal Energies	-3927.904260
Sum of electronic and thermal Enthalpies	-3927.903316
Sum of electronic and thermal Free Energies	-3927.951600

cartesian

6	1.65699995	1.40859997	-0.11290000	6	-1.66550004	0.75360000	1.15439999
6	1.66520000	0.75440001	1.15390003	6	-2.18300009	-0.55739999	0.97310001
6	2.18269992	-0.55669999	0.97380000	6	-2.45650005	-0.72270000	-0.41110000
6	2.45659995	-0.72320002	-0.41020000	1	-2.87129998	-1.61310005	-0.87099999
6	2.14969993	0.49720001	-1.07449996	1	-2.27640009	0.69980001	-2.13269997
1	2.27710009	0.69800001	-2.13290000	1	-1.35899997	2.43260002	-0.29990000
1	1.35930002	2.43239999	-0.30180001	1	-2.35560012	-1.28989995	1.74940002
1	2.35509992	-1.28859997	1.75059998	40	0.00000000	-0.44040000	-0.21110000
1	2.87140012	-1.61389995	-0.86930001	1	0.00000000	-1.79999995	0.99080002
6	-2.14930010	0.49810001	-1.07439995	1	-1.38110006	1.19669998	2.10080004
6	-1.65690005	1.40869999	-0.11190000	1	1.38059998	1.19830000	2.09990001

I-1

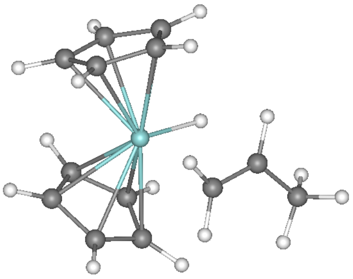

Zero-point vibrational energy	679571.4 (Joules/Mol)
	162.42147 (Kcal/Mol)
Zero-point correction	0.258835 (Hartree/Particle)
Thermal correction to Energy	0.272972
Thermal correction to Enthalpy	0.273916
Thermal correction to Gibbs Free Energy	0.217132
Sum of electronic and zero-point Energies	-4045.725664
Sum of electronic and thermal Energies	-4045.711527
Sum of electronic and thermal Enthalpies	-4045.710583
Sum of electronic and thermal Free Energies	-4045.767368

cartesian

6	-1.47160006	1.97000003	0.09150000	1	1.12310004	-2.31660008	1.93949997
6	-0.56910002	2.12630010	1.17659998	1	2.63319993	-0.09750000	1.92200005
6	0.72579998	2.33270001	0.63309997	40	0.11790000	0.02150000	0.00490000
6	0.61890000	2.32189989	-0.78759998	6	-1.86329997	-1.14859998	-1.27929997
6	-0.74040002	2.10500002	-1.11650002	6	-2.20580006	-1.71319997	-0.09920000
1	-1.14909995	2.05559993	-2.11969995	6	-3.37770009	-1.29939997	0.72960001
1	-2.53900003	1.80550003	0.17850000	1	-2.47569990	-0.36019999	-1.71150005
1	-0.82840002	2.11129999	2.22639990	1	-1.11179996	-1.61179996	-1.92429996
1	1.63170004	2.50999999	1.19939995	1	-0.59230000	-0.41630000	1.62849998
6	1.25500000	-2.18289995	-0.28979999	1	-3.08450007	-1.14110005	1.76950002
6	1.47889996	-1.80180001	1.05739999	1	-4.11250019	-2.11190009	0.72030002
6	2.28320003	-0.63069999	1.04639995	1	-3.85570002	-0.40059999	0.33710000
6	2.58380008	-0.31169999	-0.30500001	1	3.20120001	0.51190001	-0.64170003
6	1.94140005	-1.26110005	-1.13259995	1	1.42410004	2.48889995	-1.49220002
1	1.99220002	-1.29729998	-2.21510005	1	-1.63660002	-2.57159996	0.26040000
1	0.70889997	-3.05839992	-0.62210000				

I-2_a

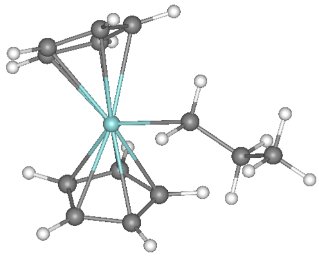

Zero-point vibrational energy	692418.1 (Joules/Mol)
	165.49189 (Kcal/Mol)
Zero-point correction	0.263728 (Hartree/Particle)
Thermal correction to Energy	0.278005
Thermal correction to Enthalpy	0.278949

Thermal correction to Gibbs Free Energy	0.221886
Sum of electronic and zero-point Energies	-4045.724543
Sum of electronic and thermal Energies	-4045.710267
Sum of electronic and thermal Enthalpies	-4045.709323
Sum of electronic and thermal Free Energies	-4045.766386

cartesian

6	1.25453222	-0.82727426	0.13028066	1	1.19003224	-1.90927410	-0.02301934
6	-1.45456779	-1.94137430	-0.85861933	6	2.61503220	-0.33497423	-0.34651935
6	-2.37816763	-0.90597433	-1.16931939	1	1.19263220	-0.69377428	1.26388061
6	-3.09626770	-0.58437425	0.01858066	6	3.77413225	-1.14177418	0.24828066
6	-2.59786797	-1.40057421	1.05988061	1	2.75553226	0.72402573	-0.11071934
6	-1.57646775	-2.23247433	0.52178067	1	4.73403215	-0.77677429	-0.12361935
1	-3.89386797	0.14262575	0.10568066	1	3.78363228	-1.06487417	1.33928061
1	-2.94536781	-1.40387416	2.08758068	1	3.69023228	-2.19967413	-0.01401935
6	-0.95916778	2.09132576	-1.12651932	1	-0.77886778	-2.42087412	-1.55491936
6	-1.65726781	2.42062569	0.07118066	1	-1.01036787	-2.97737432	1.06878066
6	-0.70566779	2.52432585	1.11248064	1	-2.55206776	-0.48367426	-2.15131927
6	0.58053219	2.26062584	0.56568062	1	-0.91856778	2.77552581	2.14618063
6	0.42583221	2.00902581	-0.82101929	1	-2.72306776	2.58802581	0.16358066
1	1.22183216	1.79592574	-1.52311933	1	-1.40016770	1.98142576	-2.10931921
1	1.51633227	2.27682590	1.11218059	1	2.63953209	-0.41937426	-1.43901932
40	-0.72606778	0.12802576	0.35578066				

I-2_b

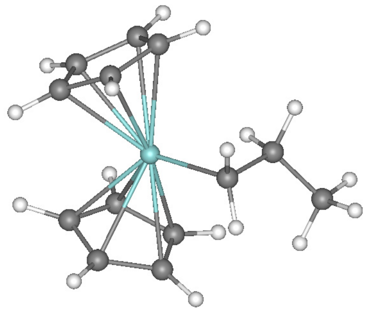

Zero-point vibrational energy	691916.6 (Joules/Mol)
	165.37203 (Kcal/Mol)
Zero-point correction	0.263537 (Hartree/Particle)
Thermal correction to Energy	0.277639
Thermal correction to Enthalpy	0.278583
Thermal correction to Gibbs Free Energy	0.221509
Sum of electronic and zero-point Energies	-4045.741248
Sum of electronic and thermal Energies	-4045.727146

Sum of electronic and thermal Enthalpies	-4045.726202
Sum of electronic and thermal Free Energies	-4045.783276

cartesian

6	-2.11848068	-1.58108389	-0.14251934	1	2.20761919	1.20691609	-1.50481939
6	-2.27318048	-0.70938390	-1.25841939	1	-0.07478060	2.50741625	-2.10351944
6	-2.69238043	0.55231607	-0.77201939	40	-0.37938058	0.23621611	0.09298065
6	-2.78158045	0.46861610	0.64678061	6	2.74611950	-1.76658392	-0.46151933
6	-2.44428062	-0.86048388	1.02758062	6	0.79401934	-1.34388387	1.16368067
1	-2.42968082	-1.24948394	2.03858066	6	1.23741937	-1.80138385	-0.21391934
1	-1.81658065	-2.62068367	-0.18491934	1	0.19241941	-2.08668375	1.68658066
1	-2.12528062	-0.97538388	-2.29921937	1	1.62921941	-1.02328384	1.78578067
1	-2.91318083	1.42621613	-1.37261939	1	0.79541945	-1.15248394	-1.06351936
6	1.65331936	1.61761606	0.62318063	1	2.99111938	-1.98818386	-1.50201941
6	1.50821936	1.62441611	-0.78911936	1	3.16401958	-0.78938389	-0.20491935
6	0.30511940	2.31311607	-1.10721934	1	-1.20548058	3.30591631	0.20648065
6	-0.28788060	2.73841619	0.10958065	1	-3.10838079	1.25901616	1.31188059
6	0.53871942	2.30051613	1.17718065	1	3.22941923	-2.51198387	0.17298065
1	0.36231941	2.47731590	2.23238063	1	0.81471944	-2.77388382	-0.47601935
1	2.48131943	1.20021605	1.18068063				

I-3

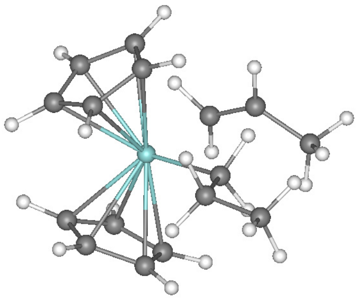

Zero-point vibrational energy	911019.4 (Joules/Mol)
	217.73885 (Kcal/Mol)
Zero-point correction	0.346989 (Hartree/Particle)
Thermal correction to Energy	0.366459
Thermal correction to Enthalpy	0.367403
Thermal correction to Gibbs Free Energy	0.299219
Sum of electronic and zero-point Energies	-4163.530477
Sum of electronic and thermal Energies	-4163.511007
Sum of electronic and thermal Enthalpies	-4163.510063
Sum of electronic and thermal Free Energies	-4163.578247

cartesian							
6	0.02170000	-1.44910002	-2.03469992	1	2.11450005	-1.92340004	1.23619998
6	-0.50770003	-2.28489995	-1.01929998	40	-0.40869999	0.01480000	-0.00610000
6	-1.85259998	-1.89600003	-0.78579998	1	2.29940009	-2.24810004	-0.47409999
6	-2.14610004	-0.79799998	-1.62689996	6	1.85060000	-0.17240000	-0.04180000
6	-0.98369998	-0.52380002	-2.40420008	6	2.52390003	-1.51100004	0.30500001
1	-0.90619999	0.22570001	-3.18350005	6	4.04409981	-1.38339996	0.44180000
1	1.01689994	-1.51479995	-2.45490003	1	2.23550010	0.60850000	0.63150001
1	0.01410000	-3.09649992	-0.52600002	1	2.16520000	0.11550000	-1.05820000
1	-2.53550005	-2.36389995	-0.08840000	1	4.50610018	-2.35039997	0.65560001
1	-3.09920001	-0.28529999	-1.69250000	1	4.30600023	-0.69700003	1.25209999
6	-0.28110000	1.26209998	2.19239998	1	4.48369980	-0.99510002	-0.48130000
6	0.23480000	-0.03180000	2.43390012	6	-0.70580000	2.53150010	-0.91079998
6	-0.81660002	-0.96499997	2.24020004	6	0.53799999	2.43989992	-1.42700005
6	-1.99839997	-0.23480000	1.92270005	6	1.73640001	3.14240003	-0.87599999
6	-1.66680002	1.13540006	1.88919997	1	-1.57149994	2.14129996	-1.45150006
1	-2.35599995	1.94620001	1.68359995	1	-0.90120000	3.17050004	-0.05350000
1	0.27710000	2.18889999	2.25850010	1	0.68480003	1.87320006	-2.34759998
1	1.25409997	-0.27020001	2.70810008	1	2.60139990	2.47830009	-0.82260001
1	-0.74519998	-2.03940010	2.36260009	1	1.54020000	3.57410002	0.10710000
1	-2.98200011	-0.65249997	1.75020003	1	2.00020003	3.95569992	-1.56140006

I-3_a

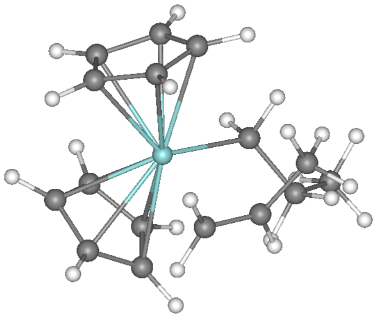

Zero-point vibrational energy	911998.7 (Joules/Mol)
	217.97293 (Kcal/Mol)
Zero-point correction	0.347362 (Hartree/Particle)
Thermal correction to Energy	0.366687
Thermal correction to Enthalpy	0.367631
Thermal correction to Gibbs Free Energy	0.300474
Sum of electronic and zero-point Energies	-4163.527795
Sum of electronic and thermal Energies	-4163.508470
Sum of electronic and thermal Enthalpies	-4163.507526
Sum of electronic and thermal Free Energies	-4163.574683

cartesian							
6	0.26818997	-2.53561258	-0.11927500	40	-0.77161002	-0.23171255	-0.02197501
6	-1.00251007	-2.65811253	0.50132501	1	2.86349010	0.68998742	-0.10307501
6	-1.99821007	-2.38921261	-0.47967499	6	1.19678998	0.00528744	1.09912503
6	-1.34471011	-2.05821252	-1.68327498	6	2.55209017	-0.23171255	0.40342501
6	0.06208995	-2.13901258	-1.45927501	6	3.65189028	-0.63451254	1.39072502
1	0.83698988	-1.97401249	-2.19977498	1	1.08908999	-0.72651255	1.91982496
1	1.22448993	-2.70611262	0.35742500	1	1.22958994	0.97618747	1.60962498
1	-1.18241000	-2.96021247	1.52632499	1	4.61659002	-0.74931252	0.89022499
1	-3.06971025	-2.42731261	-0.32807499	1	3.40828991	-1.58471251	1.87612498
1	-1.83481002	-1.81591249	-2.61897492	1	3.76209021	0.11948743	2.17472506
6	-1.36191010	1.85478747	1.32422495	6	-0.47171006	1.08268750	-2.47327495
6	-1.58151007	0.73658741	2.16422510	6	0.67548990	1.40638745	-1.84117496
6	-2.64070988	-0.02681255	1.60972500	6	0.94368994	2.70998740	-1.15517497
6	-3.07711029	0.62138748	0.42212498	1	-0.53421009	0.18768746	-3.08547497
6	-2.29030991	1.78908741	0.25522500	1	-1.30440998	1.78028750	-2.52627492
1	-2.40701008	2.52398753	-0.53167498	1	1.50808990	0.70228744	-1.89867496
1	-0.62631005	2.63288736	1.48542500	1	1.37388992	2.56218743	-0.16077501
1	-1.03331006	0.50548744	3.06932497	1	0.04578993	3.32738757	-1.08437502
1	-3.06341028	-0.92901254	2.03362489	1	1.68989003	3.25808740	-1.74137497
1	-3.89000988	0.30148745	-0.21857500	1	2.48739004	-0.99561250	-0.38257501

I-3_b

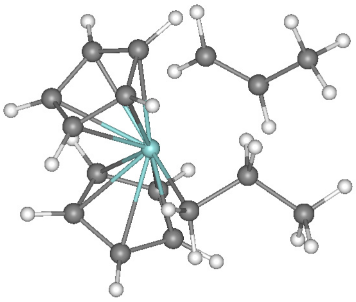

Zero-point vibrational energy	913477.1 (Joules/Mol)
	218.32626 (Kcal/Mol)
Zero-point correction	0.347925 (Hartree/Particle)
Thermal correction to Energy	0.366985
Thermal correction to Enthalpy	0.367929
Thermal correction to Gibbs Free Energy	0.300920
Sum of electronic and zero-point Energies	-4163.529300
Sum of electronic and thermal Energies	-4163.510240
Sum of electronic and thermal Enthalpies	-4163.509296
Sum of electronic and thermal Free Energies	-4163.576305

cartesian							
6	0.64131999	-2.50299239	-0.15420251	1	-2.28688002	2.10580754	-1.19280255
6	1.71011996	-2.15159249	0.72099745	1	-2.65637994	1.50290751	-2.81060243
6	2.70562005	-1.51049244	-0.05050251	6	-0.05888000	0.63030750	-2.08400249
6	2.24832010	-1.43409240	-1.39190257	6	-1.43247998	0.15750751	-1.65060258
6	0.98342001	-2.07109237	-1.45490253	1	0.33052000	0.10360751	-2.95280242
1	0.37911999	-2.19419241	-2.34430242	1	-0.05478001	1.70230758	-2.27790260
1	-0.26097998	-3.03849244	0.11759749	1	-1.44728005	-0.16219249	-0.55910254
1	1.77751994	-2.37509251	1.77839744	1	-3.50377989	0.83470750	-1.41160250
1	2.78062010	-0.99189252	-2.22500253	6	-0.65157998	-0.88539249	2.34319758
6	0.78711998	1.92920756	1.66569746	6	-1.74088013	-0.28729248	1.82159746
6	2.06172013	1.31680763	1.62759745	6	-2.93847990	-1.03849244	1.31619751
6	2.62551999	1.58000755	0.34439752	1	0.11412001	-0.33339250	2.88099742
6	1.68911994	2.32440758	-0.40500250	1	-0.59958005	-1.97009242	2.39229751
6	0.53711998	2.52140760	0.40469751	1	-1.82828009	0.79960752	1.86989748
1	-0.35007998	3.07740760	0.12509750	1	-3.35807991	-0.59629250	0.41059750
1	0.11802000	1.94850755	2.51789737	1	-2.70867991	-2.09019232	1.13269746
1	2.54952002	0.79620749	2.44289756	1	-3.71998000	-0.99119252	2.08199739
1	1.83491993	2.69230747	-1.41110253	1	3.61482000	1.29470754	0.01129749
40	0.71032000	0.01990751	-0.03390251	1	3.65622020	-1.14999247	0.32019749
6	-2.54038000	1.20920753	-1.76530254	1	-1.71768010	-0.77239251	-2.15070248

TS-2

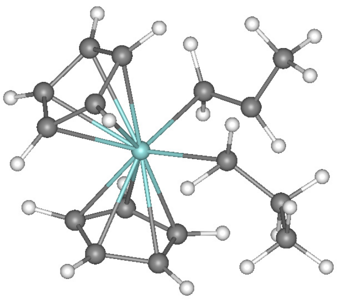

Zero-point vibrational energy	916072.3 (Joules/Mol)
	218.94652 (Kcal/Mol)
Zero-point correction	0.348913 (Hartree/Particle)
Thermal correction to Energy	0.366397
Thermal correction to Enthalpy	0.367341
Thermal correction to Gibbs Free Energy	0.304683
Sum of electronic and zero-point Energies	-4163.517412
Sum of electronic and thermal Energies	-4163.499929
Sum of electronic and thermal Enthalpies	-4163.498984
Sum of electronic and thermal Free Energies	-4163.561642

cartesian

6	-0.23428001	2.28946257	-1.20005500	40	-0.90178001	0.12046253	-0.05215501
6	-0.19028001	2.55216265	0.18854499	1	3.32031989	-0.04233747	-0.10835501
6	-1.50668001	2.43206263	0.70444500	6	1.29981995	-0.24453747	0.58984500
6	-2.37177992	2.13006258	-0.38245502	6	2.60112000	0.53916252	0.47784498
6	-1.58738005	2.03696251	-1.55615497	6	3.20971990	0.84326249	1.85044503
1	-1.96138000	1.83326256	-2.55185509	1	0.68751991	0.28586254	1.38804495
1	0.61052001	2.30816269	-1.87875497	1	1.47231996	-1.24063754	1.00634503
1	0.69271994	2.80306268	0.76474500	1	4.15952015	1.37146246	1.74454498
1	-1.80568004	2.59806252	1.73214495	1	2.54202008	1.46966255	2.45024490
1	-3.44657993	2.01376247	-0.32515502	1	3.39511991	-0.07853747	2.40804482
6	-1.29478002	-2.25733733	0.73824501	6	-0.18988001	-0.79013747	-2.12965512
6	-1.47328007	-1.42693746	1.87254500	6	1.10061991	-0.83013749	-1.55525506
6	-2.60088015	-0.60203749	1.64324498	6	1.79931998	-2.15523744	-1.37545502
6	-3.13698006	-0.94113743	0.36914498	1	-0.38398001	-0.00813747	-2.85755515
6	-2.33687997	-1.96943748	-0.18305501	1	-0.66158003	-1.74553752	-2.33925509
1	-2.51187992	-2.46573734	-1.12815499	1	1.75022006	0.01586253	-1.76735497
1	-0.52968001	-3.01463747	0.62204498	1	2.65192008	-2.11193728	-0.69705498
1	-0.85337996	-1.42023754	2.76144481	1	1.10601997	-2.92963743	-1.04135501
1	-3.00678015	0.12846252	2.33194494	1	2.17371988	-2.45393729	-2.36025524
1	-4.02417994	-0.51633751	-0.08345501	1	2.43741989	1.47336257	-0.07105501

TS-3

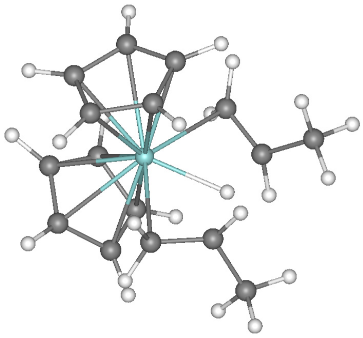

Zero-point vibrational energy	904319.0 (Joules/Mol)
	216.13742 (Kcal/Mol)
Zero-point correction	0.344437 (Hartree/Particle)
Thermal correction to Energy	0.362458
Thermal correction to Enthalpy	0.363403
Thermal correction to Gibbs Free Energy	0.299638
Sum of electronic and zero-point Energies	-4163.506150
Sum of electronic and thermal Energies	-4163.488129
Sum of electronic and thermal Enthalpies	-4163.487185

Sum of electronic and thermal Free Energies -4163.550949

cartesian

6	0.69806993	-2.56201506	0.10210251	1	-2.12783003	2.32648492	-0.96729749
6	1.92306995	-2.09351492	0.64990252	1	-3.18313003	1.52488494	-2.13369727
6	2.65977001	-1.49601507	-0.40149748	6	-0.39673007	0.55068499	-2.18329740
6	1.87786984	-1.54741502	-1.58069754	6	-1.58293009	0.28858498	-1.48019755
6	0.66736996	-2.22441506	-1.26609755	1	-2.00393009	-0.71101505	-1.60389745
1	-0.13163006	-2.45931506	-1.95959747	1	-0.01453006	-0.19981501	-2.86399746
1	-0.06673005	-3.10931492	0.63760251	1	-0.16813004	1.57868493	-2.44529748
1	2.25856996	-2.21831512	1.67180252	1	-1.33582997	0.00008499	0.00000251
1	2.17837000	-1.18441498	-2.55539727	1	-3.30573010	1.11568499	-0.42079750
6	0.66766995	2.22438502	1.26620245	6	-0.39673007	-0.55061501	2.18330264
6	1.87817001	1.54718494	1.58060253	6	-1.58293009	-0.28841501	1.48020256
6	2.65997005	1.49578500	0.40130252	6	-2.60512996	-1.37731504	1.21680248
6	1.92316985	2.09338498	-0.64989752	1	-0.01443005	0.19978499	2.86400270
6	0.69816995	2.56198502	-0.10209749	1	-0.16813004	-1.57861507	2.44530272
1	-0.06653005	3.10938501	-0.63749748	1	-2.00382996	0.71118498	1.60400248
1	-0.13133007	2.45928502	1.95980251	1	-3.30573010	-1.11551499	0.42090249
1	2.17866993	1.18418503	2.55530262	1	-2.12792993	-2.32631493	0.96730250
1	2.25856996	2.21818495	-1.67189753	1	-3.18313003	-1.52471507	2.13370252
40	0.66036993	-0.00001501	0.00000251	1	3.66017008	1.09268498	0.32430249
6	-2.60512996	1.37748504	-1.21679747	1	3.66006994	-1.09291506	-0.32449749

I-4_a

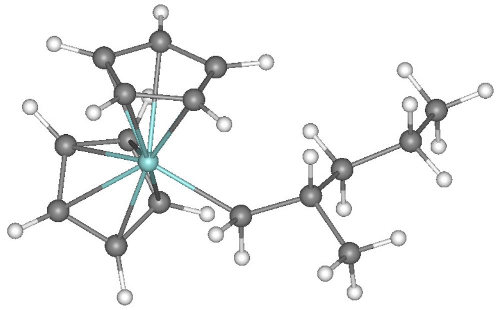

Zero-point vibrational energy 917695.1 (Joules/Mol)
219.33440 (Kcal/Mol)
Zero-point correction 0.349532 (Hartree/Particle)
Thermal correction to Energy 0.367821
Thermal correction to Enthalpy 0.368765
Thermal correction to Gibbs Free Energy 0.301980
Sum of electronic and zero-point Energies -4163.528347
Sum of electronic and thermal Energies -4163.510058

Sum of electronic and thermal Enthalpies	-4163.509114
Sum of electronic and thermal Free Energies	-4163.575899

cartesian

6	0.27660000	2.43810010	0.44749999	6	-0.83260000	-0.37979999	1.23679996
6	1.69649994	2.50600004	0.45739999	6	-2.23799992	0.17299999	1.02219999
6	2.17000008	2.02230000	-0.78689998	6	-2.84349990	-0.51200002	-0.21349999
6	1.03900003	1.64830005	-1.56680000	1	-0.90579998	-1.43929994	1.51090002
6	-0.13020000	1.91760004	-0.80430001	1	-0.34740001	0.13150001	2.12879992
1	-1.15190005	1.75940001	-1.12779999	1	-2.18210006	1.25150001	0.82620001
1	-0.37909999	2.74340010	1.25460005	6	-4.20989990	0.02870000	-0.63830000
1	2.30970001	2.88290000	1.26909995	1	-2.92630005	-1.59010005	-0.01370000
1	3.20630002	1.97060001	-1.09749997	1	-2.14439988	-0.41389999	-1.05820000
6	1.68499994	-2.34910011	0.72759998	1	-2.63380003	0.40799999	3.15499997
6	2.91499996	-1.70669997	0.40720001	1	-3.25799990	-1.09819996	2.46329999
6	2.84089994	-1.25430000	-0.92909998	1	1.20560002	-1.44149995	-2.44989991
6	1.55970001	-1.61230004	-1.44060004	1	1.06400001	1.27800000	-2.58389997
6	0.85740000	-2.31110001	-0.42120001	1	-4.08629990	0.43630001	2.15490007
1	-0.13480000	-2.73410010	-0.51029998	6	-4.69540024	-0.60920000	-1.93949997
1	1.43949997	-2.81500006	1.67509997	1	-4.94479990	-0.15640000	0.15060000
1	3.76679993	-1.59689999	1.07009995	1	-4.14870024	1.11759996	-0.75849998
1	3.62339997	-0.73930001	-1.47210002	1	-5.67460012	-0.22280000	-2.22979999
40	1.12940001	0.06400000	0.33149999	1	-4.00019979	-0.40889999	-2.76049995
6	-3.10570002	-0.03080000	2.27110004	1	-4.78329992	-1.69430006	-1.83299994

I-4_b

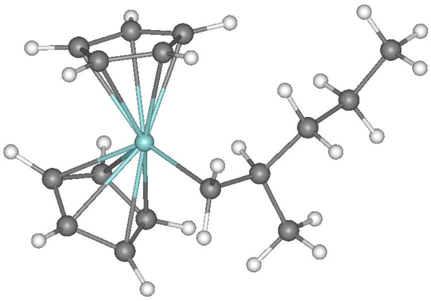

Zero-point vibrational energy	918784.4 (Joules/Mol)
	219.59475 (Kcal/Mol)
Zero-point correction	0.349947 (Hartree/Particle)
Thermal correction to Energy	0.367846
Thermal correction to Enthalpy	0.368790
Thermal correction to Gibbs Free Energy	0.303902
Sum of electronic and zero-point Energies	-4163.545046
Sum of electronic and thermal Energies	-4163.527147

Sum of electronic and thermal Enthalpies	-4163.526203
Sum of electronic and thermal Free Energies	-4163.591091

cartesian

6	0.25549507	2.35509753	0.96692002	6	-0.05660492	-0.68800247	1.67331994
6	-0.01800489	2.24369740	-0.42147997	6	-0.96850491	-0.96370250	0.49622002
6	1.21089506	2.36619735	-1.12608004	6	-2.29060483	-0.18170251	0.54332000
6	2.24189520	2.56249738	-0.17197998	1	-0.49130487	-0.00070250	2.39931989
6	1.65459514	2.54329753	1.11961997	1	0.29239509	-1.59430254	2.16872001
1	2.17869520	2.67769742	2.05942011	1	-0.48820496	-0.57560247	-0.49787995
1	-0.47070491	2.33469748	1.76852000	6	-3.08430481	-0.17680250	-0.76337999
1	-0.99590492	2.11339760	-0.87228000	1	-2.89600492	-0.62450248	1.34361994
1	1.33409500	2.34949756	-2.20248008	1	-2.09200501	0.85129750	0.85332000
6	2.42749500	-2.03290248	0.31212002	1	-1.61760497	-2.62700248	-0.77377999
6	2.03749514	-1.84130251	-1.03978002	1	-0.23800492	-3.01190257	0.26982000
6	2.81529498	-0.78010249	-1.57798004	1	4.43609524	0.45619750	-0.66387999
6	3.69199514	-0.32300252	-0.56028003	1	3.28989506	2.72279739	-0.39037997
6	3.44489503	-1.08860254	0.60872000	1	-1.85250497	-2.86500263	0.96661997
1	3.95959496	-0.98590249	1.55762005	6	-4.30530500	0.73919749	-0.68217999
1	2.04859495	-2.78540254	0.99052000	1	-3.40990496	-1.19260252	-1.00777996
1	1.29459500	-2.41800261	-1.57897997	1	-2.43400502	0.14569750	-1.58917999
1	2.77199507	-0.40880251	-2.59507990	1	-4.87470484	0.72309750	-1.61318004
40	1.35839510	0.23709750	0.18092002	1	-4.01070499	1.77469742	-0.48577997
6	-1.17630494	-2.45560265	0.21062002	1	-4.97320509	0.42529750	0.12452002

I-4_g

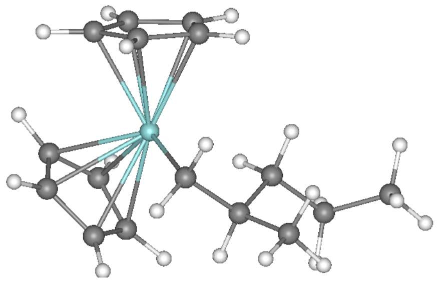

Zero-point vibrational energy	920011.5 (Joules/Mol)
	219.88803 (Kcal/Mol)
Zero-point correction	0.350414 (Hartree/Particle)
Thermal correction to Energy	0.368487
Thermal correction to Enthalpy	0.369431
Thermal correction to Gibbs Free Energy	0.304050
Sum of electronic and zero-point Energies	-4163.536704
Sum of electronic and thermal Energies	-4163.518631

Sum of electronic and thermal Enthalpies	-4163.517687
Sum of electronic and thermal Free Energies	-4163.583068

cartesian

6	-1.00805748	-2.36746240	0.76401752	40	-1.23915744	0.02073751	0.03131752
6	-2.08315754	-1.80846250	1.51451755	1	3.02844262	-1.33566248	0.90371752
6	-3.13335752	-1.51686251	0.61431748	6	1.49374247	0.13323750	0.48291752
6	-2.70685744	-1.87586248	-0.69378251	6	2.71984243	-0.35926250	1.29121745
6	-1.40235758	-2.42136240	-0.59598249	6	3.88924265	0.62683749	1.26041746
1	-0.81575751	-2.81106257	-1.41728246	1	2.43214250	-0.52226251	2.33291769
1	-0.06915754	-2.72596240	1.17121744	1	4.74504232	0.21973750	1.80281746
1	-2.10415745	-1.65706253	2.58761764	1	3.61074257	1.57013750	1.73831749
1	-4.09535742	-1.09396255	0.87611753	1	4.21314240	0.84933752	0.24151751
1	-3.28945756	-1.78286254	-1.60298252	6	0.10204250	-0.07616249	-1.74098253
6	-1.02385747	2.52543759	-0.27388248	6	1.34744239	-0.52896249	-0.91978252
6	-1.41905749	2.30973744	1.07301748	6	2.61734247	-0.25336251	-1.74338245
6	-2.71535754	1.73983753	1.06471753	1	-0.07735750	-0.78436249	-2.55208230
6	-3.12085748	1.60253751	-0.29278249	1	0.29794246	0.90333748	-2.18818235
6	-2.07665753	2.10013747	-1.11708248	1	1.29374242	-1.61166251	-0.75438249
1	-2.08295751	2.14093757	-2.19888234	1	3.50724268	-0.68986249	-1.28308249
1	-0.08305752	2.94993758	-0.60248250	1	2.77534246	0.82333750	-1.86068249
1	-0.83695751	2.55243754	1.95521748	1	2.50534248	-0.68796247	-2.73888230
1	-3.29965734	1.47063756	1.93611753	1	0.64384252	-0.07906249	1.19101751
1	-4.07615757	1.22623754	-0.63748252	1	1.53614259	1.22493756	0.38101754

TS-4

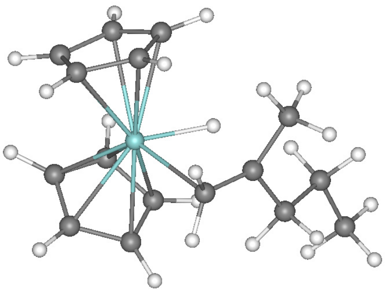

Zero-point vibrational energy	906276.5 (Joules/Mol)
	216.60528 (Kcal/Mol)
Zero-point correction	0.345182 (Hartree/Particle)
Thermal correction to Energy	0.363060
Thermal correction to Enthalpy	0.364004
Thermal correction to Gibbs Free Energy	0.299366
Sum of electronic and zero-point Energies	-4163.531410
Sum of electronic and thermal Energies	-4163.513533

Sum of electronic and thermal Enthalpies	-4163.512588
Sum of electronic and thermal Free Energies	-4163.577226

cartesian

6	-2.36525011	-2.05958486	0.22251251	6	0.22614992	-0.83908498	1.87041259
6	-1.93115008	-1.79458487	-1.10248744	6	1.10144997	-1.09188485	0.81771255
6	-2.69115019	-0.70088494	-1.59728742	6	1.18324995	-2.47228479	0.21381250
6	-3.61105013	-0.31058496	-0.58828747	1	-0.35195008	-1.66618490	2.27991247
6	-3.40264988	-1.13918495	0.53921252	1	0.46024990	-0.02528495	2.55551243
1	-3.95905018	-1.09958494	1.46901250	1	0.19284993	-0.36558497	-0.65458745
1	-2.01605010	-2.85828495	0.86451250	1	1.37114990	-2.44318485	-0.86008745
1	-1.17815006	-2.34258485	-1.65348744	1	0.28884989	-3.06368494	0.41351250
1	-2.60914993	-0.26948494	-2.58738756	6	2.94374990	-0.19498493	-0.77328748
6	-0.17995009	2.32681513	1.01381254	1	2.09065008	0.80981505	0.95191252
6	0.05994993	2.21611500	-0.38048750	1	-3.24755001	2.70441508	-0.25398749
6	-1.18965006	2.33481503	-1.04638743	1	-4.35335016	0.47271508	-0.67088747
6	-2.19425011	2.54321504	-0.06448749	1	3.05805016	-0.59408498	1.33681250
6	-1.57455003	2.52621508	1.20711255	6	4.06754971	0.83681506	-0.86698747
1	-2.07185006	2.67181516	2.15961242	1	2.17254972	0.01981506	-1.52308750
1	0.56864989	2.31981516	1.79621255	1	3.34284973	-1.18598485	-1.01048744
1	1.02324986	2.09281516	-0.85978746	1	4.85154963	0.63361502	-0.13248751
1	-1.34105015	2.31101513	-2.11848760	1	3.69324970	1.84851515	-0.68098748
40	-1.30395007	0.21201506	0.26451251	1	4.52654982	0.82711506	-1.85718751
6	2.31624985	-0.20918493	0.62171251	1	2.03294992	-2.98128486	0.68481255

I-5

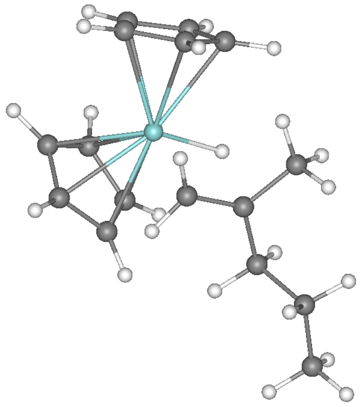

Zero-point vibrational energy	907054.2 (Joules/Mol)
	216.79116 (Kcal/Mol)
Zero-point correction	0.345479 (Hartree/Particle)
Thermal correction to Energy	0.364444
Thermal correction to Enthalpy	0.365388
Thermal correction to Gibbs Free Energy	0.296641
Sum of electronic and zero-point Energies	-4163.535388
Sum of electronic and thermal Energies	-4163.516423
Sum of electronic and thermal Enthalpies	-4163.515479
Sum of electronic and thermal Free Energies	-4163.584225

cartesian

6	-2.15187001	-2.20005250	0.61726499	6	0.63773000	-0.43695250	1.57796502
6	-1.95137000	-2.18965244	-0.78273499	6	1.39903009	-0.78105253	0.50266498
6	-2.91267014	-1.31425250	-1.35593498	6	1.43203008	-2.18635249	-0.02413500
6	-3.71497011	-0.79975253	-0.30983499	1	0.11682999	-1.20715249	2.14896512
6	-3.23497009	-1.32505250	0.91786498	1	0.77803004	0.53164750	2.05506492
1	-3.65877008	-1.14295256	1.89846504	1	-0.47057000	-0.03665251	-1.50003505
1	-1.60687006	-2.79745245	1.33836496	1	1.35413003	-2.19615245	-1.11523497
1	-1.20687008	-2.74975252	-1.33373499	1	2.40063000	-2.62845230	0.23406500
1	-3.01606989	-1.09665251	-2.41163492	1	0.65513003	-2.81655240	0.41156501
6	-0.53276998	2.48724747	0.24736500	1	2.09852982	0.28024748	-1.21043503
6	-1.14636993	2.38504767	-1.02803504	1	2.20602989	1.19084752	0.30546501
6	-2.53337002	2.16574764	-0.82003498	1	-3.74057007	2.03954744	1.06326497
6	-2.77747011	2.15674758	0.58216500	1	-4.54946995	-0.11965252	-0.42493498
6	-1.54136992	2.35554767	1.23866498	6	3.81162977	-0.22355250	0.01256500
1	-1.39446998	2.40794754	2.31166506	6	4.75342989	0.85994744	-0.50893497
1	0.51682997	2.67984748	0.42956501	1	3.98732996	-1.15365255	-0.53543502
1	-0.65206999	2.48264766	-1.98463511	1	4.02022982	-0.42635250	1.06836498
1	-3.28046989	2.05904746	-1.59703505	1	5.79442978	0.54634750	-0.41203499

40	-1.41936994	0.13664749	0.05176500	1	4.63103008	1.79194748	0.04936500
6	2.33723021	0.20134750	-0.14283499	1	4.56253004	1.06974745	-1.56483495

I-6

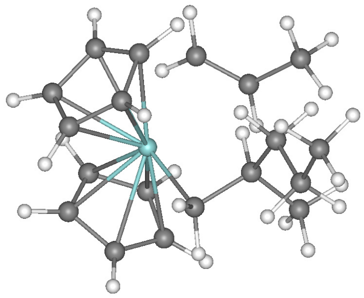

Zero-point vibrational energy	1137201.0 (Joules/Mol)
	271.79755 (Kcal/Mol)
Zero-point correction	0.433137 (Hartree/Particle)
Thermal correction to Energy	0.456374
Thermal correction to Enthalpy	0.457318
Thermal correction to Gibbs Free Energy	0.380895
Sum of electronic and zero-point Energies	-4281.331907
Sum of electronic and thermal Energies	-4281.308670
Sum of electronic and thermal Enthalpies	-4281.307725
Sum of electronic and thermal Free Energies	4281.384149

cartesian

6	0.64018160	-0.72293872	-2.39606524	6	-1.77121830	-1.76503873	1.06543469
6	0.74558163	-1.95013881	-1.69546533	1	-0.60611838	0.81356132	1.17053473
6	2.12268162	-2.22893882	-1.50486529	1	-0.57471842	1.04966140	-0.58036530
6	2.87168169	-1.16253877	-2.06256533	1	-3.09471846	0.68496132	1.14333475
6	1.94948149	-0.23413870	-2.62066531	1	-2.98331833	1.04776120	-0.57316530
1	2.20928168	0.66736132	-3.16396523	6	2.82458162	2.22926116	-0.47336531
1	-0.28211838	-0.24603869	-2.70456529	6	1.59028161	2.68606114	-0.76936531
1	-0.07981843	-2.57683873	-1.37976527	6	0.77818161	3.56086135	0.13303469
1	2.53058171	-3.11223865	-1.03006530	1	3.44348168	1.73076129	-1.22106528
1	3.95278168	-1.09173870	-2.09446526	1	3.30388165	2.51516128	0.45903468
6	2.20478153	0.35146132	2.29093480	1	1.17118168	2.47206116	-1.75426531
6	1.33968163	-0.76603872	2.33343458	1	-0.24421841	3.19166136	0.24093468
6	2.00528169	-1.85613871	1.71523476	1	1.23748159	3.66266131	1.11773467
6	3.30908155	-1.42113876	1.34023476	1	0.71128160	4.55596113	-0.32006532
6	3.43018174	-0.06283869	1.69093466	1	-1.88081837	-1.37403870	2.08433461
1	4.31238174	0.54866129	1.54293466	1	-2.59981823	-2.45513868	0.89023471
1	1.98848152	1.33786130	2.68533468	1	-0.84371835	-2.34813881	1.02523470

1	0.34248161	-0.78903872	2.75053477	6	-4.34901857	-0.54603869	-0.11156531
1	1.61108160	-2.85903883	1.60283470	1	-4.50321865	-1.29843879	0.66803467
1	4.07468176	-2.02573872	0.87043470	1	-4.26681852	-1.08933878	-1.06096530
40	1.64498162	-0.20613870	-0.10806531	6	-5.56041861	0.38546133	-0.15436532
1	-1.74681830	-1.05983877	-0.95936531	1	-5.67401838	0.91996127	0.79333472
6	-0.52931839	0.27396131	0.21353470	1	-6.48321867	-0.16943869	-0.33706534
6	-1.76701832	-0.62093866	0.04793468	1	-5.45461845	1.13116121	-0.94766527
6	-3.05061841	0.22176132	0.14763469				

TS-5

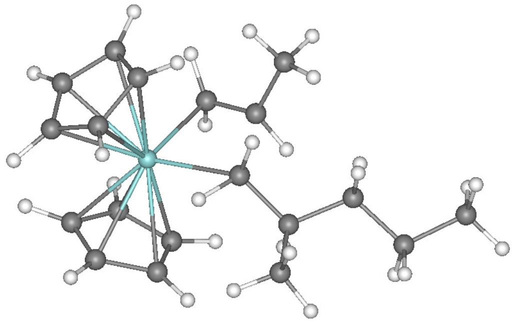

Zero-point vibrational energy	1142195.1 (Joules/Mol)
	272.99118 (Kcal/Mol)
Zero-point correction	0.435039 (Hartree/Particle)
Thermal correction to Energy	0.456539
Thermal correction to Enthalpy	0.457483
Thermal correction to Gibbs Free Energy	0.385706
Sum of electronic and zero-point Energies	-4281.316222
Sum of electronic and thermal Energies	-4281.294723
Sum of electronic and thermal Enthalpies	-4281.293778
Sum of electronic and thermal Free Energies	-4281.365555

cartesian

6	-1.12747550	-2.19465518	1.37963676	6	2.86632442	0.22094485	-0.23706324
6	-1.11807537	-2.56295514	0.01573674	1	-0.33147544	-0.31345513	-1.40266323
6	-2.44707537	-2.47755527	-0.47616327	1	0.55252457	1.17614484	-1.16566324
6	-3.28457546	-2.09325528	0.60683674	6	4.17112446	-0.57855511	-0.16076325
6	-2.47107553	-1.91155517	1.74913681	1	2.97302437	1.01294482	-0.99206322
1	-2.81827545	-1.62645519	2.73443675	1	2.73262453	0.72894490	0.72443676
1	-0.26467544	-2.16045523	2.03463674	6	-1.07837546	0.91314489	2.07633662
1	-0.24807543	-2.86075521	-0.55646324	6	0.15002453	1.09234476	1.39653683
1	-2.77117538	-2.71545529	-1.48216319	6	0.59272456	2.49244475	1.04453683
1	-4.35977554	-1.97065520	0.56863672	1	-1.10897541	0.14814484	2.84503675
6	-2.21507549	2.01844478	-1.22686327	1	-1.65577543	1.80604482	2.29633665

6	-2.76357555	0.95504493	-1.99706328	1	0.93782461	0.38484484	1.63933682
6	-3.86907530	0.42854485	-1.28976321	1	-0.24767545	3.09404492	0.68923676
6	-4.00077534	1.15324485	-0.07426326	1	0.96042454	2.95454478	1.96723676
6	-2.99217558	2.15004492	-0.05186326	6	1.83742464	-1.35315514	-1.91656315
1	-2.86097527	2.89784479	0.71873671	6	5.31352472	0.25964484	0.41223672
1	-1.37997544	2.64464474	-1.51506317	1	4.45212460	-0.93475515	-1.15566325
1	-2.41227531	0.62124485	-2.96646333	1	4.01682472	-1.46895516	0.46113673
1	-4.50637531	-0.38195515	-1.62056315	1	0.94652462	-1.90975523	-2.22536325
1	-4.76437521	1.00214481	0.67933673	1	2.66242456	-2.06555510	-1.85246325
40	-1.83397543	-0.11885515	0.08243674	1	5.09392452	0.57894486	1.43523681
1	1.50122464	-1.38195515	0.20833674	1	5.48052454	1.15724480	-0.19046324
6	0.34952462	0.22594485	-0.66586328	1	6.24682474	-0.30655515	0.43203673
6	1.62582457	-0.62415510	-0.57836324	1	2.06992435	-0.63405514	-2.70936322
				1	1.39792454	2.52794480	0.31143674

TS-6

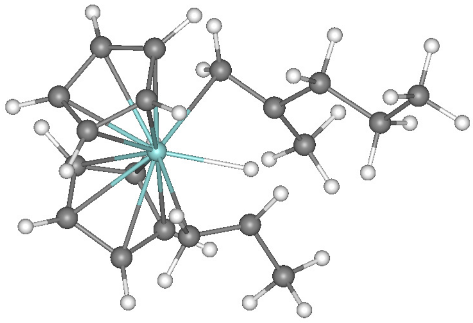

Zero-point vibrational energy	1127819.9 (Joules/Mol)
	269.55543 (Kcal/Mol)
Zero-point correction	0.429564 (Hartree/Particle)
Thermal correction to Energy	0.451801
Thermal correction to Enthalpy	0.452745
Thermal correction to Gibbs Free Energy	0.379531
Sum of electronic and zero-point Energies	-4281.305669
Sum of electronic and thermal Energies	-4281.283432
Sum of electronic and thermal Enthalpies	-4281.282488
Sum of electronic and thermal Free Energies	-4281.355702

cartesian

6	-1.58554292	-2.32785296	0.35126126	6	1.50915718	-1.61075306	1.63716125
6	-2.44114280	-1.98185301	-0.72603869	1	-0.40554282	-0.24655306	2.84546113
6	-3.44764280	-1.12305307	-0.23593874	1	0.10305721	1.42594695	2.31916118
6	-3.24044275	-0.96305311	1.16486132	1	0.57195717	-0.42385307	-0.20563874
6	-2.10634279	-1.72205305	1.52476132	1	1.99595714	-2.12135315	0.80676126

1	-1.72804284	-1.85035300	2.52926111	1	2.22965717	-1.56965303	2.46296120
1	-0.74154282	-3.00475311	0.30076125	1	0.65655714	-2.20245314	1.97416127
1	-2.36164284	-2.34525299	-1.74103868	1	1.81465721	1.58964694	0.29606125
1	-3.86184287	-0.39085308	1.84246123	6	-0.68984282	-0.10875309	-2.38213873
6	-1.13614273	2.57264686	-0.95963871	6	0.53785723	-0.49445310	-1.84033871
6	-2.46724272	2.10384703	-1.16813874	6	1.00645709	-1.92705297	-1.98303878
6	-3.08874273	2.00814700	0.09886126	1	-0.78724283	0.88284695	-2.80263877
6	-2.13734293	2.34734702	1.09026122	1	-1.32654285	-0.86455309	-2.82593894
6	-0.93804282	2.72634697	0.42106125	1	1.32045722	0.26574692	-1.82763875
1	-0.03304282	3.09274697	0.88876128	1	1.77745724	-2.20915294	-1.26693869
1	-0.40554282	2.79574704	-1.72793877	1	0.17175716	-2.62615299	-1.90153873
1	-2.94044280	1.91814697	-2.12423873	1	1.42975712	-2.03415298	-2.98693895
1	-2.31134272	2.37604690	2.15876126	1	-4.11474276	1.71444690	0.28016126
40	-1.32024288	0.19504693	0.02556126	1	-4.25384283	-0.69335306	-0.81753874
6	2.23335719	0.76174688	0.88146126	1	3.04755735	-0.45135310	-0.73483872
1	2.62815714	1.21694696	1.79916131	1	3.91605735	-0.58525312	0.77956128
6	3.40225720	0.12594692	0.12526126	6	4.39515686	1.18694699	-0.34983876
6	0.06315720	0.36854693	2.08646107	1	5.24865723	0.72944689	-0.85323870
6	1.11035728	-0.17925307	1.32936132	1	4.77655697	1.77044690	0.49266124
				1	3.92405725	1.88244700	-1.05123878

S1.3. Me_2AlH reaction profile

I-0H

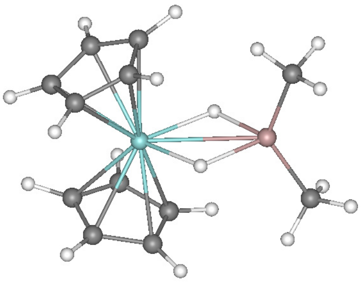

Zero-point vibrational energy	679540.8 (Joules/Mol)
	162.41414 (Kcal/Mol)
Zero-point correction	0.258823 (Hartree/Particle)
Thermal correction to Energy	0.274570
Thermal correction to Enthalpy	0.275514
Thermal correction to Gibbs Free Energy	0.214702
Sum of electronic and zero-point Energies	-4250.669473
Sum of electronic and thermal Energies	-4250.653726
Sum of electronic and thermal Enthalpies	-4250.652782
Sum of electronic and thermal Free Energies	-4250.713595

cartesian

6	1.82288432	-1.90424991	1.15558445	1	3.58228421	1.38665009	0.31368440
6	2.63388443	-1.61284995	0.02818439	1	2.37378430	1.76175010	-2.06411576
6	1.87768435	-1.89014995	-1.14011562	1	-0.14301562	2.61045003	-1.59161556
6	0.60298437	-2.37514997	-0.73471558	1	-0.49281561	2.73134995	1.07608438
6	0.56868434	-2.38364983	0.68458438	40	0.72808439	0.00415001	-0.00681561
1	-0.25921559	-2.71374989	1.29998446	1	-0.88371557	-0.02334999	1.09408438
1	2.11988449	-1.80964994	2.19408417	1	-0.87391561	-0.03134999	-1.12851560
1	3.65698433	-1.25914991	0.05418439	13	-2.28861570	-0.03524999	-0.02131561
1	2.22418427	-1.78444993	-2.16191578	6	-3.11041570	1.72725010	0.00058439
1	-0.19281563	-2.69914985	-1.39391565	6	-3.09901571	-1.80065000	0.00608439
6	2.55578423	1.69675004	0.16368438	1	-4.12461567	1.69525003	-0.41031560
6	1.91688442	1.89345002	-1.08991563	1	-3.20421576	2.10275006	1.02638435
6	0.59158438	2.34695005	-0.84061557	1	-2.55761576	2.48045015	-0.57031560
6	0.40728438	2.41094995	0.56558442	1	-3.87501574	-1.88974988	-0.76151562
6	1.62208438	2.00475001	1.18498445	1	-2.39351559	-2.62214994	-0.15121561
1	1.80838442	1.96175003	2.25228429	1	-3.59481573	-1.97994995	0.96688437

I–1H

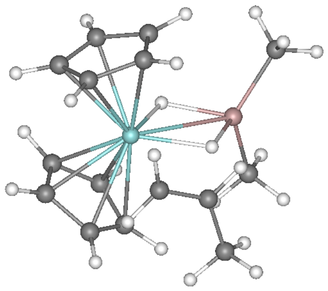

Zero-point vibrational energy	901973.9 (Joules/Mol)
	215.57695 (Kcal/Mol)
Zero-point correction	0.343544 (Hartree/Particle)
Thermal correction to Energy	0.364822
Thermal correction to Enthalpy	0.365766
Thermal correction to Gibbs Free Energy	0.293380
Sum of electronic and zero-point Energies	-4368.459600
Sum of electronic and thermal Energies	-4368.438321
Sum of electronic and thermal Enthalpies	-4368.437377
Sum of electronic and thermal Free Energies	-4368.509764

cartesian

6	-1.34009516	-2.25256109	0.69904149	1	0.72410482	0.94093907	1.27554142
6	-2.24829531	-1.43406093	1.40644145	1	1.06780481	0.04693904	-0.64055860
6	-1.51449513	-0.74176097	2.41314149	13	2.25710487	0.75943905	0.42284143
6	-0.15979518	-1.14236093	2.32164145	6	2.86430478	2.47483897	-0.27495858
6	-0.04059519	-2.05366111	1.23734140	6	3.35040474	-0.62576097	1.24874151
1	0.86580479	-2.55826116	0.92534137	1	3.95330477	2.49063897	-0.38585857
1	-1.59869516	-2.93856096	-0.09625857	1	2.44090486	2.71153903	-1.25745857
1	-3.31699514	-1.37576091	1.23944151	1	2.61120486	3.30493903	0.39504144
1	-1.92439520	-0.05916096	3.14704156	1	4.08660460	-0.18786095	1.93164146
1	0.64330482	-0.81586093	2.96924138	1	2.79580474	-1.38046098	1.81474149
6	-1.45969522	2.06483889	-1.24815857	1	3.92920494	-1.16066098	0.48554143
6	-2.63249516	1.46653903	-0.69585860	6	-1.34509516	-1.00266099	-2.29835844
6	-2.63099527	1.72253907	0.69644141	6	-0.00389519	-1.13206100	-2.40525866
6	-1.44629514	2.42663884	1.01614141	6	0.77520478	-2.38176107	-2.12525845
6	-0.73329520	2.65663886	-0.19715858	1	-1.97279513	-1.83566093	-1.99405861
1	0.20570481	3.18853903	-0.29015857	1	-1.84909523	-0.14706096	-2.73315859
1	-1.17539525	2.07813907	-2.29375863	1	1.11290479	-2.79196095	-3.08295846
1	-3.42239523	0.96793908	-1.24415851	1	1.66930485	-2.17716098	-1.53135860
1	-3.39969516	1.41923904	1.39594150	1	0.17200482	-3.14456105	-1.62995851
1	-1.14799523	2.76893902	1.99904144	1	0.55240482	-0.30896097	-2.85175848
40	-0.71489519	0.15933904	0.23614143				

TS–1H

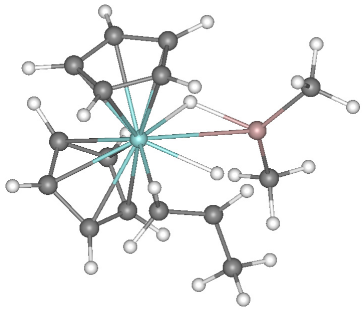

Zero-point vibrational energy	898825.0 (Joules/Mol)
	214.82433 (Kcal/Mol)
Zero-point correction	0.342344 (Hartree/Particle)
Thermal correction to Energy	0.362787
Thermal correction to Enthalpy	0.363732
Thermal correction to Gibbs Free Energy	0.294205
Sum of electronic and zero-point Energies	-4368.451149
Sum of electronic and thermal Energies	-4368.430706
Sum of electronic and thermal Enthalpies	-4368.429762
Sum of electronic and thermal Free Energies	-4368.499289

cartesian

6	-0.01282695	-2.32699752	-0.68525368	1	-1.18342686	0.97610247	2.24504638
6	0.60637307	-1.82679772	-1.86725354	6	-0.81252682	-0.03589760	2.07914639
6	1.98907316	-1.69919765	-1.61625361	6	0.54927307	-0.26239762	2.33864641
6	2.23827291	-2.12579751	-0.27905366	6	-1.83982694	-1.13259768	2.25014639
6	1.00487304	-2.54159737	0.27774635	1	-1.46282685	-2.09289742	1.89234650
1	0.87027311	-2.97439742	1.25984645	1	-2.78182697	-0.90679753	1.74674642
1	-1.06352687	-2.56959748	-0.57625365	1	-2.04222703	-1.22899771	3.32114625
1	0.10497306	-1.59549761	-2.79825377	1	-1.00742686	0.15550239	0.55124635
1	2.73257303	-1.35269761	-2.32305360	1	0.85097313	-1.25769770	2.64714622
1	3.20577312	-2.17969751	0.20534635	1	1.11847305	0.55200243	2.77084637
6	2.20667291	1.64550233	-1.40995359	13	-1.98312676	0.70570242	-0.98735368
6	3.11117291	1.08690238	-0.46805364	1	-0.37092692	0.53270245	-1.52625358
6	2.68677306	1.46050239	0.82624632	6	-2.44362688	2.54780269	-0.56675369
6	1.52917314	2.28320265	0.68634635	6	-3.10442710	-0.77159756	-1.57225358
6	1.25117314	2.41340256	-0.69055367	1	-3.41822696	-1.40669763	-0.73495370
1	0.44477305	2.99010253	-1.12675357	1	-2.60292697	-1.42229772	-2.29655361
1	2.26307297	1.55000234	-2.48715377	1	-4.02272701	-0.40789762	-2.04455352
1	3.97527289	0.47700238	-0.70035368	1	-3.46752691	2.63430262	-0.18885365
1	3.18277311	1.20160234	1.75314641	1	-2.39362693	3.17780256	-1.46325350
1	0.98457313	2.76110268	1.49274647	1	-1.78012693	3.00200248	0.17744637
40	0.88737309	-0.03519760	-0.11235365				

I-2H_a

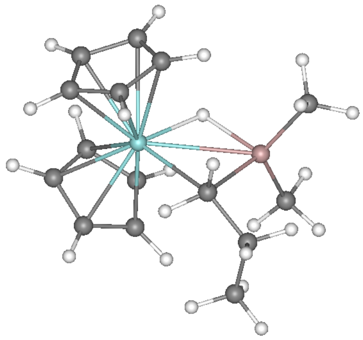

Zero-point vibrational energy	909914.3 (Joules/Mol)
	217.47475 (Kcal/Mol)
Zero-point correction	0.346568 (Hartree/Particle)
Thermal correction to Energy	0.367351
Thermal correction to Enthalpy	0.368295
Thermal correction to Gibbs Free Energy	0.296995
Sum of electronic and zero-point Energies	-4368.473827
Sum of electronic and thermal Energies	-4368.453044
Sum of electronic and thermal Enthalpies	-4368.452100
Sum of electronic and thermal Free Energies	-4368.523400

cartesian

6	1.72529995	-1.90310001	-1.28160000	1	1.31099999	2.03509998	2.37949991
6	0.32040000	-2.05739999	-1.42200005	1	3.35050011	0.39340001	1.75979996
6	-0.21160001	-2.38599992	-0.15050000	13	-1.96010005	0.78479999	-0.87309998
6	0.86900002	-2.45919991	0.77329999	6	-3.03040004	-0.60869998	-1.71840000
6	2.06450009	-2.16669989	0.07450000	6	-2.35290003	2.69519997	-0.94010001
40	0.71950001	-0.06530000	0.05400000	1	-3.10319996	-0.39060000	-2.79150009
6	-1.32449996	0.47830001	1.20360005	1	-4.06150007	-0.61229998	-1.34549999
1	-0.28830001	0.51840001	-1.46599996	1	-2.65310001	-1.63259995	-1.63189995
6	1.19640005	2.40479994	0.18160000	1	-1.70850003	3.21180010	-1.66190004
6	1.97529995	1.89250004	-0.89469999	1	-2.22569990	3.20449996	0.02230000
6	2.93700004	1.00590003	-0.35479999	1	-3.38240004	2.88269997	-1.26259995
6	2.75119996	0.95719999	1.05509996	1	-0.66960001	-0.19149999	1.82490003
6	1.68630004	1.83959997	1.38150001	1	-1.11090004	1.49710000	1.54400003
1	-1.25000000	-2.59030008	0.07240000	6	-2.75860000	0.13290000	1.69120002
1	0.79329997	-2.71860003	1.82369995	6	-3.09960008	-1.35350001	1.68700004
1	-0.24360000	-1.94589996	-2.33949995	1	-3.50760007	0.68650001	1.10510004
1	2.42000008	-1.67019999	-2.07949996	1	-4.07049990	-1.51880002	2.15869999
1	3.06279993	-2.15799999	0.49439999	1	-2.35770011	-1.92690003	2.25340009
1	3.69260001	0.46810001	-0.91360003	1	-3.15809989	-1.75790000	0.67430001
1	1.85769999	2.15100002	-1.93970001	1	-2.86409998	0.52730000	2.70650005
1	0.38270000	3.11509991	0.08990000				

I-2H_bi

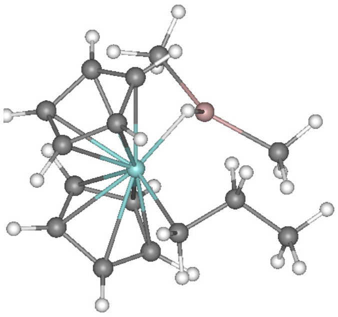

Zero-point vibrational energy	906854.6 (Joules/Mol)
	216.74345 (Kcal/Mol)
Zero-point correction	0.345403 (Hartree/Particle)
Thermal correction to Energy	0.366043
Thermal correction to Enthalpy	0.366987
Thermal correction to Gibbs Free Energy	0.295655
Sum of electronic and zero-point Energies	-4368.459386
Sum of electronic and thermal Energies	-4368.438745
Sum of electronic and thermal Enthalpies	-4368.437801
Sum of electronic and thermal Free Energies	-4368.509134

cartesian

6	2.64969993	-0.19610000	-1.34940004	1	-0.94970000	-0.18770000	-1.23099995
6	2.84920001	-1.00440001	-0.20200001	1	-0.07820000	1.69959998	-1.18579996
6	1.93610001	-2.09220004	-0.27320001	13	-2.54719996	-0.40930000	-0.82999998
6	1.14950001	-1.93320000	-1.43529999	6	-3.50690007	1.27269995	-0.75940001
6	1.59080005	-0.75349998	-2.10260010	6	-3.10229993	-2.25460005	-1.03009999
1	1.19970000	-0.36989999	-3.03719997	1	-3.91020012	1.53139997	-1.74510002
1	3.20810008	0.69520003	-1.60259998	1	-2.87290001	2.11019993	-0.44830000
1	3.58999991	-0.83929998	0.57090002	1	-4.36439991	1.23380005	-0.07920000
1	1.85749996	-2.90310001	0.44010001	1	-2.31830001	-2.96580005	-0.75070000
1	0.36289999	-2.59780002	-1.77119994	1	-3.36610007	-2.46880007	-2.07200003
6	-0.93269998	0.73689997	2.04329991	1	-3.99070001	-2.48830009	-0.43430001
6	0.33199999	0.40419999	2.57329988	6	1.53610003	2.00580001	0.31240001
6	0.53619999	-0.99030000	2.39129996	6	0.68260002	2.47779989	-0.85100001
6	-0.61299998	-1.53219998	1.76650000	6	-0.16190000	3.72510004	-0.56980002
6	-1.52830005	-0.46529999	1.55780005	1	2.60570002	2.09949994	0.13890000
1	-2.59759998	-0.59469998	1.38429999	1	1.27349997	2.53020000	1.23160005
1	-1.39800000	1.71589994	2.05399990	1	-0.81349999	3.97119999	-1.41120005
1	1.02639997	1.08749998	3.04279995	1	0.49349999	4.57700014	-0.37799999
1	1.40840006	-1.54849994	2.70860004	1	-0.78490001	3.57859993	0.31760001
1	-0.78829998	-2.57529998	1.53240001	1	1.27880001	2.60310006	-1.75899994
40	0.56339997	-0.02450000	0.07110000				

I-2H_b'

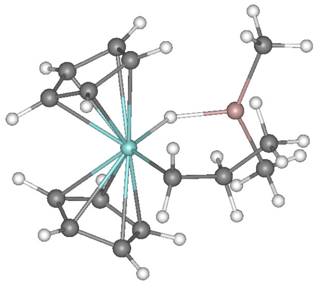

Zero-point vibrational energy	905923.6 (Joules/Mol)
	216.52093 (Kcal/Mol)
Zero-point correction	0.345048 (Hartree/Particle)
Thermal correction to Energy	0.366394
Thermal correction to Enthalpy	0.367338
Thermal correction to Gibbs Free Energy	0.293828
Sum of electronic and zero-point Energies	-4368.457077
Sum of electronic and thermal Energies	-4368.435731
Sum of electronic and thermal Enthalpies	-4368.434787
Sum of electronic and thermal Free Energies	-4368.508297

cartesian

6	1.61150002	2.22346354	-0.96474630	1	-0.56909990	0.26316339	1.43715358
6	0.73910010	2.52486348	0.10855365	6	-3.01130009	-1.44233668	1.20875359
6	1.40260005	2.18386340	1.32305360	6	-2.90100002	2.12356353	1.05995357
6	2.66899991	1.65826333	0.99275368	1	-3.94039989	2.18856359	1.39525354
6	2.79369998	1.65926337	-0.42564636	1	-2.88540006	2.46436357	0.01705365
1	3.65649986	1.32926333	-0.99184638	1	-2.31590009	2.84106350	1.64355361
1	1.41420007	2.39876342	-2.01374650	1	-4.01639986	-1.44163668	0.77625364
6	1.97640014	-1.45163667	1.87145364	1	-3.10739994	-1.82073665	2.23375368
6	2.83360004	-1.53673661	0.73545367	1	-2.40919995	-2.17403650	0.65925366
6	2.12980008	-2.19403648	-0.29904634	1	1.00670004	2.31126356	2.32295370
6	0.83320010	-2.51463652	0.19035365	1	-0.23899993	2.98516345	0.02545364
6	0.75530005	-2.08463645	1.53735363	1	2.51539993	-2.41993642	-1.28434646
1	-0.09449995	-2.20953655	2.19725370	1	0.06090006	-3.04223657	-0.35744631
1	2.22889996	-1.02233660	2.83315349	1	3.41669989	1.31476331	1.69665360
40	0.98760009	0.01606340	0.11495364	1	3.85529995	-1.18223667	0.68085366
6	-0.85069990	0.09866340	-1.88674641	6	-1.93679988	-0.88433659	-2.32654643
6	0.57500005	-0.33483660	-2.11134648	1	-1.04089987	1.10876334	-2.26044631
1	-1.05419993	0.25426340	-0.75354636	1	-2.93810010	-0.54663658	-2.04314637
1	1.10590005	0.27906340	-2.83504629	1	-1.76719987	-1.87233663	-1.89014649
1	0.62890005	-1.38323665	-2.40484643	1	-1.90659988	-0.98933655	-3.41314650
13	-2.21210003	0.32096338	1.20055366				

I-2H_bo

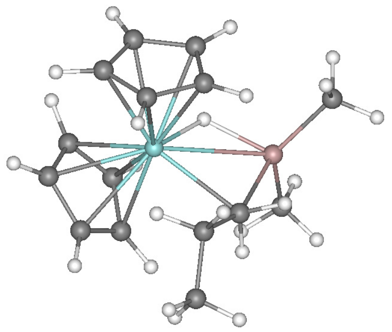

Zero-point vibrational energy	911044.0 (Joules/Mol)
	217.74473 (Kcal/Mol)
Zero-point correction	0.346998 (Hartree/Particle)
Thermal correction to Energy	0.367848
Thermal correction to Enthalpy	0.368792
Thermal correction to Gibbs Free Energy	0.297628
Sum of electronic and zero-point Energies	-4368.483495
Sum of electronic and thermal Energies	-4368.462645
Sum of electronic and thermal Enthalpies	-4368.461701
Sum of electronic and thermal Free Energies	-4368.532865

cartesian

6	-0.55215120	2.41029024	-0.87821954	6	3.00974870	-1.20140982	1.56788039
6	-1.86345124	1.94829011	-1.19521952	6	2.94754887	2.07719016	0.41238046
6	-2.61555123	1.90769017	0.00138044	1	2.33484888	-2.04440975	1.75418043
6	-1.77455127	2.33789039	1.06358051	1	3.46884871	-0.94110984	2.52788043
6	-0.51125121	2.67649031	0.51238048	1	3.82334900	-1.57660985	0.93598044
1	0.33714879	3.06689024	1.06008053	1	2.99124885	2.68329024	1.32518053
1	0.25014877	2.58599019	-1.58461952	1	2.43634868	2.67869020	-0.34721953
1	-2.23085117	1.69899011	-2.18381953	1	3.98574877	1.96349013	0.08018044
1	-2.05945110	2.44009018	2.10348034	6	0.13364878	-1.95320976	-2.86871958
6	-0.70195121	-2.23020983	0.92288047	6	1.15544879	-0.46440980	-1.04951954
6	-1.89645123	-2.08630967	0.16598044	6	0.08064878	-0.60950977	-2.13641953
6	-2.76955128	-1.22810984	0.87718046	1	0.13444880	0.22169019	-2.84261942
6	-2.12215137	-0.85060978	2.08648038	1	1.95484877	0.14599019	-1.49601960
6	-0.85375124	-1.48360980	2.12148046	1	1.57234883	-1.45730984	-0.84991950
1	-0.12975122	-1.41590989	2.92338061	1	-0.99045122	-0.54990977	-1.77411962
1	0.14974877	-2.84630966	0.66208047	1	0.04404879	-2.78300977	-2.16191959
1	-2.11285114	-2.56470966	-0.78261954	1	1.09194875	-2.05330968	-3.38241959
1	-2.54085112	-0.22310981	2.86348057	1	-3.76955128	-0.94680983	0.57228041
40	-0.80635118	0.18589020	0.24208045	1	-3.65475130	1.61749017	0.08918045
1	0.58164877	0.56099021	1.51158047	1	-0.66545117	-2.03630972	-3.60831952
13	2.13734889	0.33889019	0.75908047				

I-4H_a

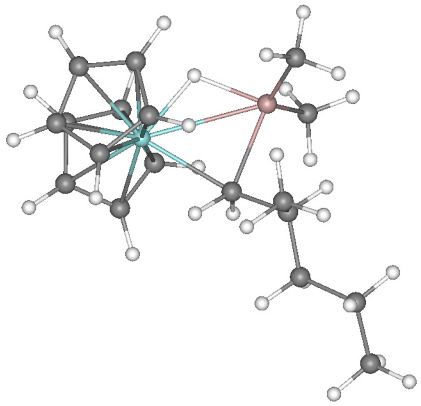

Zero-point vibrational energy	1135934.0 (Joules/Mol)
	271.49475 (Kcal/Mol)
Zero-point correction	0.432654 (Hartree/Particle)
Thermal correction to Energy	0.457474
Thermal correction to Enthalpy	0.458418
Thermal correction to Gibbs Free Energy	0.378226
Sum of electronic and zero-point Energies	-4486.277142
Sum of electronic and thermal Energies	-4486.252323
Sum of electronic and thermal Enthalpies	-4486.251378
Sum of electronic and thermal Free Energies	-4486.331571

cartesian

6	3.79535007	1.53163600	0.42124194	6	-0.57705003	-1.00206399	-3.04495811
6	2.67135000	2.24483609	-0.07365809	1	0.19114992	2.59593606	-3.24925804
6	1.67124987	2.23413610	0.92924196	1	-1.31675005	2.61943603	-2.36105824
6	2.18674994	1.53393602	2.05694175	1	0.18524992	3.06133604	-1.55365813
6	3.50014997	1.10823596	1.74604189	1	0.26644990	-1.48646402	-3.55165815
40	1.97904992	-0.15386397	0.21994191	1	-1.11525011	-1.78786397	-2.50085807
6	-0.41965014	-0.26406395	0.11154191	1	-1.24855018	-0.65226400	-3.83615804
1	1.77924991	0.30593604	-1.62315810	1	-0.07805008	-0.11676396	1.17224193
6	1.86034989	-2.49946380	-0.69595808	1	-0.53025013	-1.34636402	-0.02005809
6	3.10894990	-1.96166396	-1.11535811	6	-1.86115003	0.32623604	0.11694191
6	3.89864993	-1.73966396	0.03894191	6	-2.80145001	-0.65096396	0.84584194
6	3.13984990	-2.13526392	1.17604184	6	-1.90895009	1.73133600	0.71544194
6	1.88714981	-2.61906385	0.71334195	1	-2.23605013	0.38573605	-0.92045808
1	0.70154989	2.70923615	0.86754191	1	-2.92435002	2.13113618	0.68804193
1	1.67444992	1.37713599	2.99984193	1	-1.58575010	1.71223605	1.76384187
1	2.59314990	2.71393609	-1.04625809	1	-1.27775002	2.43033624	0.16264191
1	4.73084974	1.37973607	-0.10335809	6	-4.26865005	-0.21776398	0.88534194
1	4.16694975	0.56573600	2.40484190	1	-2.73655009	-1.62856400	0.35134190
1	4.91074991	-1.35506403	0.05434191	1	-2.43355012	-0.79456401	1.87194192
1	3.40415001	-1.77056396	-2.13935804	6	-5.16975021	-1.32906401	1.42374182

1	1.04294991	-2.77996397	-1.35005808	1	-4.59215021	0.06523603	-0.12345809
1	1.08874989	-3.00496387	1.33714187	1	-4.38105011	0.67083603	1.51354182
1	3.47654986	-2.12466383	2.20544195	1	-6.21054983	-1.00286400	1.47234190
13	0.00724989	0.46763602	-1.90015817	1	-4.86495018	-1.62556398	2.43154192
6	-0.25425011	2.36333609	-2.27365804	1	-5.12585020	-2.21586394	0.78534192

I-4H_bi

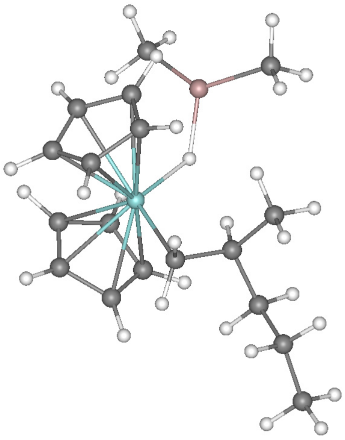

Zero-point vibrational energy	1132658.0 (Joules/Mol)
	270.71177 (Kcal/Mol)
Zero-point correction	0.431407 (Hartree/Particle)
Thermal correction to Energy	0.456920
Thermal correction to Enthalpy	0.457864
Thermal correction to Gibbs Free Energy	0.374211
Sum of electronic and zero-point Energies	-4486.262023
Sum of electronic and thermal Energies	-4486.236510
Sum of electronic and thermal Enthalpies	-4486.235565
Sum of electronic and thermal Free Energies	-4486.319218

cartesian

6	0.74059618	-2.64763188	0.74807203	6	-4.13320398	0.81736803	1.82127202
6	-0.39710388	-3.26193190	0.16937199	1	-1.54820383	3.85746813	1.60007203
6	-1.53660393	-2.88573194	0.93757200	1	-1.19450390	3.45346808	-0.07312801
6	-1.10700393	-2.01593208	1.95987189	1	-2.80740404	4.01726818	0.38817197
6	0.30379611	-1.85083199	1.83387196	1	-4.08170366	-0.26583201	1.97057211
1	0.93189609	-1.25483203	2.48547196	1	-4.35890388	1.26656806	2.79477191
1	1.76199615	-2.77703190	0.41977197	1	-5.00230360	1.02306807	1.18537199
1	-0.38940388	-3.94103193	-0.67452800	6	1.27469611	-0.51973200	-1.28932798
1	-2.55440378	-3.21563196	0.76947200	6	1.56589615	0.59946799	-0.30442804
1	-1.73480392	-1.55963194	2.71507192	6	1.54889607	1.99656796	-0.94052798
6	-1.61170387	0.46216801	-2.29222822	6	2.82599592	0.35586801	0.54927200
6	-1.44690382	-0.88843203	-2.66732812	1	2.01279593	-1.31973195	-1.26752794

6	-2.37600374	-1.66823196	-1.92862797	1	1.17049611	-0.15413201	-2.31162810
6	-3.12540388	-0.80273199	-1.09722805	1	1.73029602	2.77396798	-0.19212802
6	-2.65820408	0.52266800	-1.32942796	1	2.32029629	2.07026792	-1.71082795
1	-3.18700409	1.44276798	-1.07652795	1	0.58679610	2.20476794	-1.41732800
1	-1.07950389	1.30936801	-2.70662808	6	4.13439608	0.53656793	-0.22412801
1	-0.74140388	-1.26483202	-3.39642811	1	2.82159615	1.03626800	1.41007197
1	-2.50800371	-2.74013209	-2.00652814	1	2.79269600	-0.66303205	0.95487201
1	-3.94110394	-1.08463204	-0.44212800	6	5.34229612	0.12006798	0.61517203
40	-0.70920384	-0.82533205	-0.22892801	1	4.24569607	1.58406806	-0.52092803
1	-1.21230388	0.53636801	1.15707195	1	4.10329628	-0.05113202	-1.14902794
1	0.74629617	0.67096794	0.49017203	1	6.27499628	0.28006801	0.07057199
13	-2.50930405	1.56966805	1.07397199	1	5.28899622	-0.93873203	0.88497198
6	-1.96620393	3.39036798	0.70097202	1	5.39309645	0.69936800	1.54157197

I-4H_bo

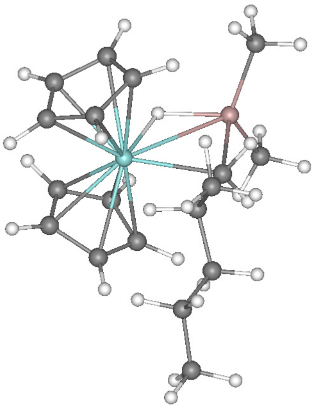

Zero-point vibrational energy	1139139.7 (Joules/Mol)
	272.26093 (Kcal/Mol)
Zero-point correction	0.433875 (Hartree/Particle)
Thermal correction to Energy	0.458266
Thermal correction to Enthalpy	0.459210
Thermal correction to Gibbs Free Energy	0.381441
Sum of electronic and zero-point Energies	-4486.286925
Sum of electronic and thermal Energies	-4486.262534
Sum of electronic and thermal Enthalpies	-4486.261590
Sum of electronic and thermal Free Energies	-4486.339359

cartesian

6	1.92533588	-0.78495610	2.07330608	1	2.53123593	2.94004393	-2.80459404
6	0.97023594	-1.83825612	2.12290597	1	1.17353594	3.77494383	-2.08329391
6	1.39263606	-2.85825610	1.23830605	1	4.20723581	2.58024383	0.46180600
6	2.61973596	-2.44485617	0.64750600	1	3.20363593	1.91594386	1.74660599
6	2.95713592	-1.17555606	1.18000603	1	2.92183614	3.54744387	1.14750600

1	3.84803605	-0.60475612	0.95150602	6	-2.46706390	0.79034388	0.20740598
1	1.91113591	0.12424389	2.66100597	6	-3.61456394	-0.13305610	0.62010598
1	0.07963598	-1.86415613	2.74020600	6	-4.82616425	0.02754390	-0.29829401
1	3.21733594	-3.01955605	-0.04879401	1	-3.27536392	-1.17865610	0.60660601
6	-0.35676402	-0.48655611	-2.35649395	1	-5.65096426	-0.61325610	0.01840599
6	-1.00306392	-1.58495617	-1.72439396	1	-4.57616425	-0.23135610	-1.33189404
6	-0.08806404	-2.66345620	-1.67149401	1	-5.18366432	1.06084383	-0.29239401
6	1.12523603	-2.23845601	-2.28109407	6	0.01843596	1.35424387	0.34610599
6	0.94993597	-0.90215611	-2.72119403	6	-1.13476396	0.51914394	0.92810595
1	1.68233585	-0.30425611	-3.24839401	6	-1.27256393	0.68734396	2.44660592
1	-0.79636401	0.47864392	-2.57619405	1	0.39013597	1.99734378	1.15630603
1	-2.02426386	-1.60285616	-1.35999405	1	-0.40446401	2.00254393	-0.43179402
1	2.01193595	-2.84245610	-2.42679405	1	-1.00086403	-0.60725611	0.79530597
40	0.87153596	-0.87595606	-0.20179401	1	-1.95856404	-0.04895610	2.87180591
1	2.30023599	0.21184391	-0.86399400	1	-1.67046404	1.68464386	2.65790606
1	-3.91156411	0.07824391	1.65180600	1	-0.31166404	0.59764385	2.95730591
13	1.97093606	1.88674378	-0.54809403	1	-2.31616402	0.72164392	-0.87629402
6	1.60593605	2.77984381	-2.23949409	1	-2.74236393	1.83294392	0.40880597
6	3.17913604	2.52764392	0.83870602	1	-0.28996402	-3.64865613	-1.27059400
1	0.92063594	2.23354387	-2.89689398	1	0.89073598	-3.80295610	1.07230604

TS-4H

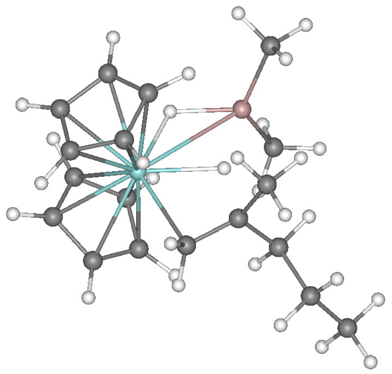

Zero-point vibrational energy	1126129.9 (Joules/Mol)
	269.15151 (Kcal/Mol)
Zero-point correction	0.428920 (Hartree/Particle)
Thermal correction to Energy	0.453290
Thermal correction to Enthalpy	0.454234
Thermal correction to Gibbs Free Energy	0.376326
Sum of electronic and zero-point Energies	-4486.258626
Sum of electronic and thermal Energies	-4486.234257
Sum of electronic and thermal Enthalpies	-4486.233313
Sum of electronic and thermal Free Energies	-4486.311221

cartesian							
6	2.16737223	1.84116399	1.12143600	1	-0.35892797	1.73096406	2.26183581
6	3.17787218	1.54126406	0.16233599	1	-1.76322806	2.10246396	1.23463607
6	3.83667183	0.36466402	0.57593602	1	-1.95472801	1.12216401	2.69613600
6	3.24047184	-0.07343599	1.79353607	1	-0.31092793	0.63176399	-0.08656400
6	2.23467207	0.86016405	2.14063597	1	-2.64212799	0.39746398	-0.37336400
1	1.63857210	0.83426404	3.04283595	1	-2.00602818	-1.24683595	-0.37686399
1	1.51587200	2.70706391	1.10213602	1	0.15937206	-0.77963597	2.64273596
1	3.40537214	2.12066388	-0.72296399	1	-0.50862789	-1.98393595	1.44043601
1	4.66047239	-0.11483599	0.06273600	13	-0.14272797	1.63626397	-1.69666398
1	3.54097223	-0.93323600	2.37993598	1	1.36157203	0.84706402	-1.55146396
6	2.69257212	-1.69493604	-1.70136404	6	-1.29182792	0.74136400	-2.98686409
6	2.78457212	-2.43263602	-0.48916399	6	-0.04772794	3.51176381	-1.18546402
6	1.48697209	-2.86173606	-0.13546400	1	0.98367202	3.88206387	-1.17616403
6	0.58687204	-2.40823603	-1.14336395	1	-0.61152792	4.14436436	-1.87826395
6	1.33587205	-1.71243596	-2.11746407	1	-0.46042794	3.69506383	-0.18646401
1	0.94497204	-1.26303601	-3.02196407	1	-2.28612804	1.19766402	-3.03766418
1	3.51497221	-1.23923600	-2.23886418	1	-0.86772799	0.81596404	-3.99606419
1	3.69197226	-2.63703609	0.06513600	1	-1.43062794	-0.32473600	-2.77626419
1	1.23227203	-3.46133614	0.72883600	6	-3.44142771	-0.86223596	1.18863606
1	-0.47502792	-2.61553621	-1.18776405	6	-4.60552788	-1.42143595	0.37173602
40	1.49457204	-0.33933598	0.02403600	1	-3.78922796	-0.01433600	1.78643596
6	-2.28962803	-0.41423601	0.27443600	1	-3.07642794	-1.61923599	1.89063597
6	-1.08642793	0.03966400	1.09043598	1	-5.42672777	-1.72563601	1.02323604
6	-0.23692793	-0.95233595	1.64683604	1	-4.29632759	-2.29563618	-0.20806399
6	-1.28962791	1.33596396	1.85343599	1	-4.99192762	-0.67403597	-0.32656398

I-5H

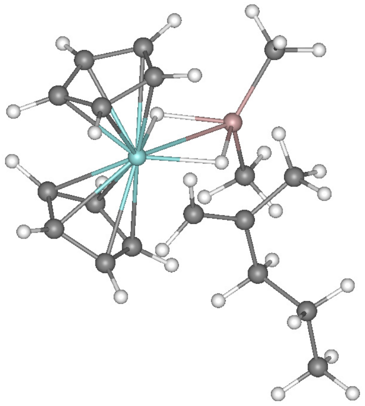

Zero-point vibrational energy	1127761.5 (Joules/Mol)
	269.54147 (Kcal/Mol)
Zero-point correction	0.429542 (Hartree/Particle)
Thermal correction to Energy	0.454940

Thermal correction to Enthalpy	0.455885
Thermal correction to Gibbs Free Energy	0.374290
Sum of electronic and zero-point Energies	-4486.269371
Sum of electronic and thermal Energies	-4486.243972
Sum of electronic and thermal Enthalpies	-4486.243028
Sum of electronic and thermal Free Energies	-4486.324622

cartesian

6	0.27738017	-2.27340412	-1.45063996	6	-0.42251986	2.48339605	-2.56674004
6	1.64218020	-2.61960411	-1.33133996	1	0.24998015	4.57549572	0.32236004
6	2.39988017	-1.64460397	-2.04323983	1	0.90948015	3.39349604	1.45046008
6	1.49738014	-0.70370400	-2.59204006	1	1.94748020	4.14899588	0.23986004
6	0.18058017	-1.07170391	-2.19933987	1	-0.32661986	3.49049616	-2.98513985
1	-0.73241985	-0.55820400	-2.47663999	1	-0.08011985	1.78359604	-3.33624005
1	-0.55191982	-2.84430408	-1.05514002	1	-1.49731982	2.30649614	-2.43463993
1	2.03578019	-3.49620390	-0.83133996	6	-0.54321980	-1.46150398	1.61926007
1	3.47478008	-1.64200401	-2.17353988	6	-1.42461979	-0.47810400	1.29255998
1	1.76428020	0.14299601	-3.21034002	6	-1.56641996	0.74979603	2.15186000
6	2.28388023	0.05889602	2.23415995	6	-2.43151999	-0.63250399	0.18516006
6	2.63878012	-1.29430401	1.94176006	1	-0.60081983	-2.44010401	1.14936006
6	3.59267998	-1.27340400	0.89966005	1	0.00218016	-1.39960396	2.55636001
6	3.79038000	0.07279602	0.50676006	1	-1.83351982	1.62889600	1.55956006
6	2.99978018	0.89759600	1.35876000	1	-2.37712002	0.57859600	2.86886001
1	2.95148039	1.97919595	1.32705998	1	-0.66011989	0.96429598	2.72076011
1	1.60938013	0.38869601	3.01476002	6	-3.86391973	-0.76000398	0.74016005
1	2.29317999	-2.17730379	2.46496010	1	-2.38741970	0.24229601	-0.47633997
1	4.07378006	-2.14000416	0.46396005	1	-2.20711994	-1.51810396	-0.41623998
1	4.46018028	0.42359602	-0.26853997	6	-4.87201977	-0.99090397	-0.38413996
40	1.41578019	-0.46090397	-0.08653995	1	-4.12951994	0.14789602	1.28965998
1	1.86578012	1.19379604	-1.00243998	1	-3.89971972	-1.58930397	1.45436001
1	-0.02061987	0.90959597	-0.01983995	1	-5.88431978	-1.07980394	0.01426005
13	0.52008015	2.32309580	-0.86703998	1	-4.64691973	-1.90910399	-0.93363994
6	0.94638014	3.73479605	0.40966004	1	-4.86301994	-0.16010398	-1.09524000

S1.4. Me_2AlCl reaction profile

I-0Cl

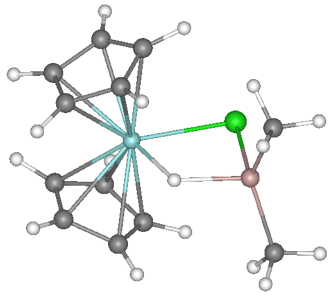

Zero-point vibrational energy	663989.4 (Joules/Mol)
	158.69729 (Kcal/Mol)
Zero-point correction	0.252900 (Hartree/Particle)
Thermal correction to Energy	0.270165
Thermal correction to Enthalpy	0.271110
Thermal correction to Gibbs Free Energy	0.207188
Sum of electronic and zero-point Energies	-4710.301965
Sum of electronic and thermal Energies	-4710.284700
Sum of electronic and thermal Enthalpies	-4710.283755
Sum of electronic and thermal Free Energies	-4710.347677

cartesian

6	1.81931889	2.07045937	-1.25137496	1	3.65041876	-1.26364052	-0.39337498
6	2.61931896	1.61115944	-0.17537498	1	2.33161879	-1.39874053	1.95592511
6	1.85041881	1.70965946	1.01562500	1	-0.10728115	-2.44374061	1.47752500
6	0.58321881	2.25915956	0.67512500	1	-0.30758116	-2.90054059	-1.17137504
6	0.56011885	2.46955943	-0.72627497	40	0.73171884	-0.00224057	-0.38787499
1	-0.26728114	2.86725950	-1.30117500	17	-1.28428113	-0.02334057	-1.98977494
1	2.11941886	2.12165952	-2.29237509	1	-0.80218112	-0.00834057	0.79312503
1	3.64481878	1.27195942	-0.24567498	13	-2.44878125	-0.00714057	0.07582501
1	2.18751884	1.46015954	2.01432490	6	-3.16248107	-1.78194046	0.38492504
1	-0.22078115	2.48135948	1.36562502	6	-3.14838123	1.77915943	0.35252503
6	2.63251877	-1.60334051	-0.25187498	1	-3.35298109	-1.95224047	1.45012510
6	1.93651879	-1.67584050	0.98622501	1	-4.12128162	-1.91184056	-0.12797499
6	0.65461886	-2.23784041	0.73602509	1	-2.50388122	-2.58344054	0.03572503
6	0.54961884	-2.48424053	-0.65667498	1	-3.30028105	1.98655951	1.41742504
6	1.77301884	-2.09704041	-1.26507497	1	-2.50088120	2.56515956	-0.04907499
1	2.00891876	-2.17274046	-2.32107496	1	-4.12478161	1.89495945	-0.12967499

I-1Cl

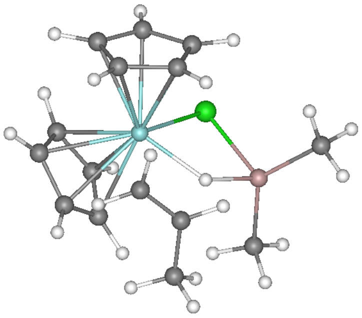

Zero-point vibrational energy	886300.5 (Joules/Mol)
	211.83090 (Kcal/Mol)
Zero-point correction	0.337574 (Hartree/Particle)
Thermal correction to Energy	0.360321
Thermal correction to Enthalpy	0.361265
Thermal correction to Gibbs Free Energy	0.286333
Sum of electronic and zero-point Energies	-4828.077044
Sum of electronic and thermal Energies	-4828.054296
Sum of electronic and thermal Enthalpies	-4828.053352
Sum of electronic and thermal Free Energies	-4828.128284

cartesian

6	1.52011204	1.95962679	1.03774643	17	-1.05428791	-1.84017313	1.72124624
6	2.51931214	1.04612684	1.43884635	1	-1.04538774	0.01212683	-0.27745366
6	1.95701218	0.20122683	2.44234633	13	-2.49988770	-0.75367320	0.32654634
6	0.62111211	0.61262685	2.65604639	6	-3.08978772	-1.92587316	-1.11035371
6	0.33071214	1.67402685	1.76224637	6	-3.48288774	0.60022682	1.31964636
1	-0.60638785	2.21302700	1.69674635	1	-3.64418769	-1.37327313	-1.87695360
1	1.65171218	2.76282692	0.32644635	1	-2.26448774	-2.44017315	-1.61465359
1	3.54111218	1.02512681	1.08104634	1	-3.76758790	-2.70067310	-0.73585361
1	2.47231221	-0.58597314	2.97894645	1	-4.33438778	0.14432684	1.83764625
1	-0.07178786	0.17102683	3.35934639	1	-2.87848783	1.09422684	2.08794641
6	1.32961226	-1.85487318	-1.64675355	1	-3.90038776	1.37732685	0.66984636
6	2.55641222	-1.27277327	-1.19535375	6	1.03871214	1.31382680	-2.06195354
6	2.84091210	-1.80537319	0.08394635	6	-0.29548785	1.48292685	-1.92375350
6	1.77311206	-2.65357304	0.45644635	6	-0.97128791	2.65372705	-1.27965355
6	0.85351211	-2.70347309	-0.63385367	1	1.74791217	2.05722666	-1.70945358
1	-0.06358786	-3.27837324	-0.65725362	1	1.42141223	0.56222683	-2.74205351
1	0.84841216	-1.68577313	-2.60285354	1	-1.45858788	3.23732662	-2.06815362
1	3.19911218	-0.60637319	-1.75735354	1	-1.75638795	2.33652687	-0.58735365
1	3.71551228	-1.58367312	0.68224633	1	-0.26928785	3.31142664	-0.76455361
1	1.67991209	-3.20027304	1.38684630	1	-0.95038790	0.77152681	-2.42635369
40	0.78701216	-0.35627317	0.31984636				

TS-1Cl

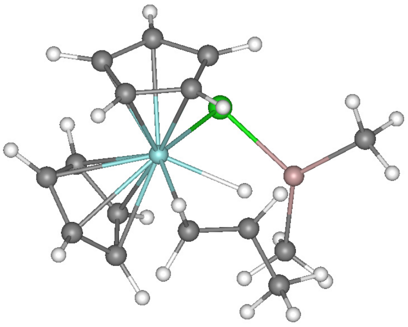

Zero-point vibrational energy	882100.4 (Joules/Mol)
	210.82706 (Kcal/Mol)
Zero-point correction	0.335974 (Hartree/Particle)
Thermal correction to Energy	0.357878
Thermal correction to Enthalpy	0.358822
Thermal correction to Gibbs Free Energy	0.285805
Sum of electronic and zero-point Energies	-4828.071225
Sum of electronic and thermal Energies	-4828.049321
Sum of electronic and thermal Enthalpies	-4828.048377
Sum of electronic and thermal Free Energies	-4828.121395

cartesian

6	0.73094153	2.45389509	0.27595359	17	-0.72865850	-0.76130480	2.40195370
6	2.07494140	2.16149521	-0.10484639	1	-0.93825847	-0.20400481	-0.33934641
6	2.72334146	1.58519518	1.00895357	13	-2.36355853	-0.42780480	0.92095363
6	1.77984142	1.47719514	2.06645346	6	-3.10505867	-2.07290483	0.20625360
6	0.56024146	2.05169511	1.61545360	6	-3.12155867	1.35149515	1.12085366
1	-0.35055852	2.13279510	2.19585371	1	-2.35075855	-2.85010481	0.04835361
1	-0.01745850	2.93179512	-0.34514642	1	-3.84585857	-2.49020481	0.89775360
1	2.53484154	2.38199520	-1.05954635	1	-3.62505865	-1.91480482	-0.74494636
1	3.76334143	1.28979516	1.04625368	1	-3.36505866	1.54659522	2.17155361
1	1.96494150	1.07569516	3.05545378	1	-2.44265866	2.15059519	0.80225360
6	1.58744144	-2.22710490	-0.90364635	1	-4.04915857	1.46999514	0.55125362
6	2.79384136	-1.51150477	-0.67234635	6	0.58274150	0.41609520	-2.12044621
6	3.01904154	-1.49240482	0.72985363	6	-0.72935849	-0.03110481	-1.91874635
6	1.93324161	-2.13600469	1.36065364	6	-1.91295862	0.88219517	-2.13104630
6	1.04564142	-2.59620476	0.34495360	1	0.71664149	1.47299516	-2.32914639
1	0.12654150	-3.14460492	0.51145363	1	1.29444146	-0.24960482	-2.59554625
1	1.15514147	-2.44860482	-1.87204635	1	-2.04785848	0.98729515	-3.21234655
1	3.45714140	-1.10810483	-1.42814636	1	-2.83965850	0.47739518	-1.71874630
1	3.87794137	-1.06660485	1.23105359	1	-1.73935866	1.87619519	-1.71474636
1	1.80424142	-2.27530479	2.42645359	1	-0.93165857	-1.09170485	-2.07224655
40	0.97804147	-0.07240481	0.29305360				

I-2Cl_a

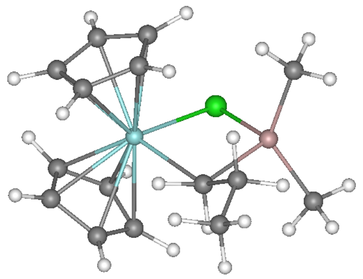

Zero-point vibrational energy	893577.6 (Joules/Mol)
	213.57016 (Kcal/Mol)
Zero-point correction	0.340346 (Hartree/Particle)
Thermal correction to Energy	0.361888
Thermal correction to Enthalpy	0.362832
Thermal correction to Gibbs Free Energy	0.290111
Sum of electronic and zero-point Energies	-4828.098749
Sum of electronic and thermal Energies	-4828.077207
Sum of electronic and thermal Enthalpies	-4828.076263
Sum of electronic and thermal Free Energies	-4828.148984

cartesian

6	-2.28972912	1.75091469	1.66371226	1	-1.32642913	-1.78958523	-2.33068776
6	-0.91312921	2.10431480	1.58761215	1	-3.54362917	-0.38498530	-1.75498772
6	-0.60262918	2.36191463	0.23611219	13	1.84377098	-0.46448532	1.17191219
6	-1.78762913	2.17091465	-0.53528780	6	2.55637097	1.21871471	1.84741211
6	-2.83262920	1.82361472	0.35631219	6	2.72697091	-2.18988514	1.01851225
40	-1.21112919	-0.07758530	0.36131218	1	2.54077077	1.22591472	2.94361234
6	0.92787081	-0.26558530	-0.80238777	1	3.60577106	1.34241474	1.55591226
17	-0.06372917	-0.88248533	2.51171231	1	2.03507090	2.12391472	1.51751208
6	-1.47002912	-2.51238537	-0.22158778	1	3.24137092	-2.44648528	1.95081210
6	-2.42632914	-2.24078536	0.79221219	1	2.02317095	-3.00428534	0.81411219
6	-3.36802912	-1.32008529	0.27611220	1	3.48587084	-2.21058536	0.22831219
6	-3.00462914	-1.02998531	-1.07198787	1	0.08757085	0.17641470	-1.40438771
6	-1.84462929	-1.78398526	-1.37888789	1	1.00807071	-1.28998530	-1.18778777
1	0.36317080	2.66301465	-0.15008783	6	2.14607096	0.52951467	-1.34428787
1	-1.88542914	2.31911469	-1.60468769	6	2.22877097	0.47621471	-2.87108779
1	-0.22312918	2.14261484	2.42181230	1	3.07937098	0.11761469	-0.93818778
1	-2.83232903	1.50221479	2.56871223	1	3.10207081	1.02381480	-3.23178768
1	-3.86472917	1.64021480	0.08561221	1	2.30737090	-0.55618531	-3.22148776
1	-4.22822905	-0.92748529	0.80431223	1	1.33937073	0.92341465	-3.32598782
1	-2.41742921	-2.64948535	1.79501224	1	2.10687089	1.56811476	-1.00328779
1	-0.62012917	-3.17858529	-0.12978780				

I-2Cl_bi

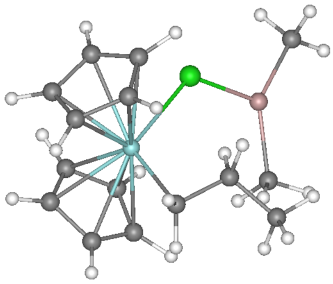

Zero-point vibrational energy	892664.4 (Joules/Mol)
	213.35190 (Kcal/Mol)
Zero-point correction	0.339998 (Hartree/Particle)
Thermal correction to Energy	0.362896
Thermal correction to Enthalpy	0.363840
Thermal correction to Gibbs Free Energy	0.286421
Sum of electronic and zero-point Energies	-4828.085847
Sum of electronic and thermal Energies	-4828.062949
Sum of electronic and thermal Enthalpies	-4828.062005
Sum of electronic and thermal Free Energies	-4828.139424

cartesian

6	-2.22079992	-1.09200001	1.86969995	17	1.58840001	-1.34500003	-0.05870000
6	-3.11680007	-0.95260000	0.77929997	1	0.67930001	0.79259998	1.36880004
6	-2.66709995	-1.80739999	-0.26280001	13	3.55819988	-0.33820000	-0.14420000
6	-1.47469997	-2.43409991	0.16329999	6	3.35859990	1.56710005	-0.41909999
6	-1.19970000	-1.99030006	1.48959994	6	4.99060011	-1.60630000	0.05920000
1	-0.36269999	-2.30340004	2.10220003	1	4.26639986	2.11520004	-0.14990000
1	-2.30119991	-0.58759999	2.82360005	1	2.53130007	1.98029995	0.16830000
1	-4.00229979	-0.32920000	0.75950003	1	3.15260005	1.79209995	-1.47259998
1	-3.15149999	-1.95510006	-1.21940005	1	4.90070009	-2.41149998	-0.67699999
1	-0.88429999	-3.13949990	-0.40880001	1	4.94840002	-2.07920003	1.04610002
6	-0.28150001	1.90369999	-1.58369994	1	5.97629976	-1.14859998	-0.05400000
6	-1.70229995	1.87020004	-1.54620004	6	-1.33589995	1.70340002	1.40380001
6	-2.12430000	0.65009999	-2.12030005	6	0.02070000	1.42449999	2.03760004
6	-0.96509999	-0.07700000	-2.52449989	6	0.85619998	2.67750001	2.32599998
6	0.16310000	0.71730000	-2.21650004	1	-0.07500000	0.81150001	2.93849993
1	1.19190001	0.44990000	-2.41989994	1	-2.15869999	1.61740005	2.11100006
1	0.34599999	2.71499991	-1.23230004	1	-1.35529995	2.69740009	0.95630002
1	-2.35069990	2.64269996	-1.15649998	1	1.84909999	2.42820001	2.70989990
1	-3.15229988	0.34040001	-2.25799990	1	0.34540001	3.29119992	3.07089996
1	-0.95160002	-1.04190004	-3.01629996	1	0.97229999	3.28230000	1.42200005
40	-0.93699998	-0.00050000	-0.03180000				

I-2Cl_bo

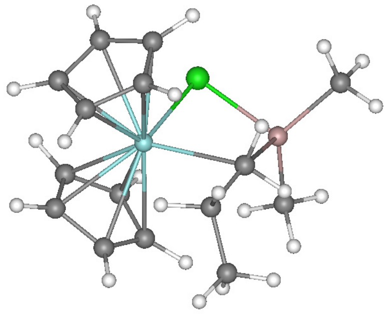

Zero-point vibrational energy	897505.9 (Joules/Mol)
	214.50906 (Kcal/Mol)
Zero-point correction	0.341842 (Hartree/Particle)
Thermal correction to Energy	0.363494
Thermal correction to Enthalpy	0.364438
Thermal correction to Gibbs Free Energy	0.293390
Sum of electronic and zero-point Energies	-4828.096474
Sum of electronic and thermal Energies	-4828.074822
Sum of electronic and thermal Enthalpies	-4828.073878
Sum of electronic and thermal Free Energies	-4828.144926

cartesian

6	1.74558544	-1.90562677	-1.35178053	6	-3.06921458	1.04487324	1.31771946
6	2.84668541	-1.15162683	-0.84918046	6	-3.40121460	-2.04962683	-0.28008050
6	3.03618550	-1.52202678	0.50891954	1	-3.14421463	1.86887324	0.59751952
6	2.02738523	-2.44582677	0.85731953	1	-2.48741460	1.40507317	2.17241955
6	1.23208547	-2.68862677	-0.30148047	1	-4.08621454	0.87787318	1.69101954
1	0.37538540	-3.34992671	-0.35048050	1	-4.13761473	-2.45152664	0.42431951
1	1.36478543	-1.87192678	-2.36528063	1	-2.76891470	-2.89012671	-0.58608049
1	3.47138548	-0.47602680	-1.42158043	1	-3.96181440	-1.72312677	-1.16348052
1	1.88728535	-2.90572667	1.82791948	6	-0.49411458	2.10327339	-2.25248051
6	0.20588541	2.02077341	1.19911957	6	-0.99941456	-0.19112681	-1.19978046
6	1.43208539	2.18257332	0.49171954	6	-0.00321458	0.67877316	-1.99518049
6	2.44088531	1.48217320	1.18961954	1	0.23938541	0.18797319	-2.94118071
6	1.84608543	0.88967323	2.34131956	1	-1.09171462	-1.17292678	-1.67398059
6	0.47778541	1.25197315	2.35611963	1	-1.96441460	0.31007320	-1.37218046
1	-0.24201459	0.96027315	3.10971975	1	1.02288532	0.78127313	-1.54158044
1	-0.75041461	2.45917320	0.94401950	1	-0.80111456	2.59817338	-1.32788050
1	1.57398546	2.76047325	-0.41408050	1	-1.36101460	2.07457328	-2.91668034
1	2.35688543	0.30507320	3.09711933	1	3.48558545	1.43157315	0.90921950
40	0.85878539	-0.27692682	0.36881953	1	3.81178546	-1.15302682	1.16771960
17	-0.85721457	-1.55542684	1.93821955	1	0.27568540	2.71067333	-2.73378038
13	-2.39321470	-0.60402679	0.52831954				

I-4Cl_a

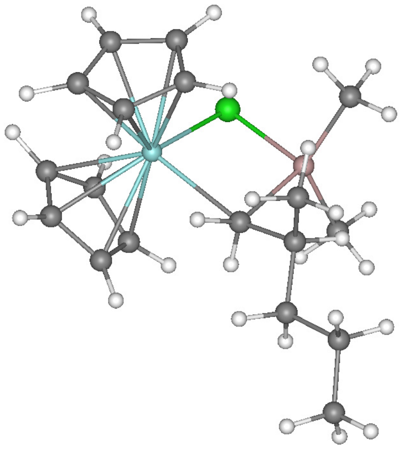

Zero-point vibrational energy	1121463.1 (Joules/Mol)
	268.03612 (Kcal/Mol)
Zero-point correction	0.427143 (Hartree/Particle)
Thermal correction to Energy	0.453303
Thermal correction to Enthalpy	0.454248
Thermal correction to Gibbs Free Energy	0.371644
Sum of electronic and zero-point Energies	-4945.898348
Sum of electronic and thermal Energies	-4945.872188
Sum of electronic and thermal Enthalpies	-4945.871243
Sum of electronic and thermal Free Energies	-4945.953847

cartesian

6	-3.85750389	0.51789600	-1.51404810	6	0.90799600	2.01249599	2.65585208
6	-2.77520394	1.40999603	-1.75204802	1	-0.09090394	4.32889605	-0.11764802
6	-1.69720387	0.66319597	-2.26854801	1	1.29529619	3.52269602	-0.82704800
6	-2.11020398	-0.70000398	-2.36664796	1	-0.32900396	3.17499614	-1.41724801
6	-3.45330381	-0.77830398	-1.92854786	1	0.78809601	2.98659611	3.14245200
40	-2.08080387	-0.18720397	0.07555199	1	0.45999604	1.27139604	3.32735205
6	0.36529607	-0.03070398	0.27485198	1	1.98449612	1.80979598	2.62435198
17	-2.24750400	2.03099608	1.35465193	1	0.00319606	-0.83250403	-0.42584801
6	-1.94300389	-1.25110400	2.35325193	1	0.51289600	-0.54180402	1.23315191
6	-3.30500388	-0.89850402	2.15845203	6	1.79939604	0.23929602	-0.29474801
6	-3.81170392	-1.66960406	1.08775198	6	2.71369600	-0.93880403	0.08285198
6	-2.76250386	-2.51480389	0.62045199	6	1.78699613	0.50829601	-1.79784799
6	-1.61920404	-2.26680398	1.42045188	1	2.23349619	1.12679601	0.19335198
1	-0.73390394	1.05479598	-2.56234789	1	2.79939604	0.67829597	-2.16964793
1	-1.51760387	-1.52050400	-2.75594807	1	1.37279606	-0.34950399	-2.34324789
1	-2.77510405	2.47219610	-1.54144812	1	1.20849609	1.40129602	-2.04234791
1	-4.83260393	0.79069597	-1.12704802	6	4.17359591	-0.78160399	-0.34864801

1	-4.06500387	-1.67130399	-1.91064787	1	2.68369603	-1.06190395	1.17325187
1	-4.82420397	-1.63800406	0.70495200	1	2.30049610	-1.86280406	-0.34774801
1	-3.84970379	-0.14910397	2.71925211	6	5.06279612	-1.86440396	0.26265198
1	-1.27980399	-0.82420403	3.09635210	1	4.53609610	0.20819601	-0.04644802
1	-0.65780395	-2.75650382	1.31805205	1	4.25189590	-0.82690400	-1.43884802
1	-2.83740401	-3.25110388	-0.17004803	1	6.09769630	-1.75690401	-0.06814802
13	0.08729607	2.02329612	0.88985199	1	4.72119617	-2.86170387	-0.02944802
6	0.25749606	3.36639619	-0.51174802	1	5.05239630	-1.81190395	1.35485196

I-4Cl_bi

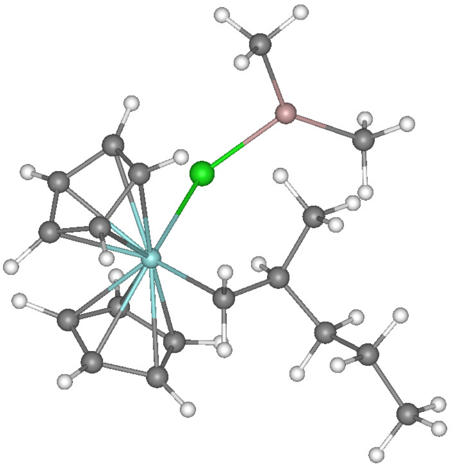

Zero-point vibrational energy	1119945.6 (Joules/Mol)
	267.67343 (Kcal/Mol)
Zero-point correction	0.426565 (Hartree/Particle)
Thermal correction to Energy	0.453157
Thermal correction to Enthalpy	0.454101
Thermal correction to Gibbs Free Energy	0.369415
Sum of electronic and zero-point Energies	-4945.889515
Sum of electronic and thermal Energies	-4945.862922
Sum of electronic and thermal Enthalpies	-4945.861978
Sum of electronic and thermal Free Energies	-4945.946664

cartesian

6	1.25066984	-2.47885180	-1.32474601	6	-2.48753023	3.07384801	-1.69074607
6	2.62376976	-2.39255190	-0.98774600	1	-0.91933024	4.97424793	1.14855397
6	3.20726991	-1.36635208	-1.78484607	1	0.18696982	3.67574811	1.59285402
6	2.19056988	-0.79925203	-2.57924581	1	0.69576979	4.90564775	0.44005403
6	0.97276986	-1.47845209	-2.28784585	1	-2.47463012	3.72124815	-2.57494593
1	0.01356983	-1.28725207	-2.75514579	1	-2.64323020	2.05224800	-2.05194592
1	0.54846984	-3.19655180	-0.92474604	1	-3.35433006	3.36514807	-1.09054601
1	3.14476991	-3.03065181	-0.28454599	6	0.04706985	-1.23505199	1.10515404

1	4.24916983	-1.07185209	-1.78184605	6	-1.03293014	-0.49245203	0.31695399
1	2.31216979	0.01234798	-3.28614593	6	-1.63173008	0.69124794	1.08975399
6	2.05426979	0.99994797	2.00615406	6	-2.11823010	-1.41845202	-0.26614600
6	2.75146985	-0.23855202	2.08165407	1	-0.07433015	-2.31695199	1.06375396
6	3.77436972	-0.21825202	1.10655403	1	0.07356983	-0.92735207	2.15155411
6	3.69876981	1.01994801	0.40715399	1	-2.40673018	1.20224798	0.50265396
6	2.65566993	1.77964795	0.99515402	1	-2.10303020	0.35154799	2.01625419
1	2.36596966	2.78024817	0.69955397	1	-0.85853016	1.41354799	1.38875401
1	1.23036981	1.29814792	2.64345407	6	-3.04083014	-2.03555202	0.78695405
1	2.54626989	-1.04375207	2.77505422	1	-2.72383022	-0.85795200	-0.99064600
1	4.49796963	-1.00405204	0.93105400	1	-1.62613010	-2.22185183	-0.82904601
1	4.35446978	1.34934795	-0.38924599	6	-3.98953009	-3.06285191	0.16885400
40	1.63516986	-0.24965201	-0.18164599	1	-3.63003016	-1.24895203	1.26975393
17	0.76306981	1.99504793	-1.64904606	1	-2.44153023	-2.50775194	1.57355392
1	-0.59763014	-0.03565201	-0.61784601	1	-4.66583014	-3.47685194	0.91935396
13	-0.83333015	3.23924804	-0.71484601	1	-3.43443012	-3.89305186	-0.27694601
6	-0.15673018	4.29484797	0.75675404	1	-4.60002995	-2.60875201	-0.61684597

I-4Cl_bo

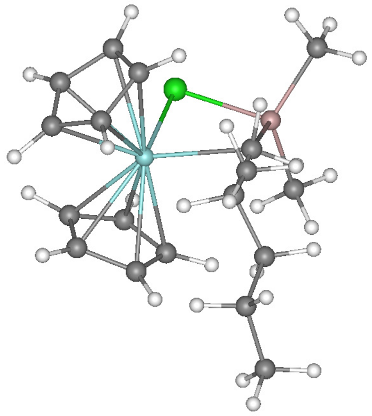

Zero-point vibrational energy	1124103.1 (Joules/Mol)
	268.66709 (Kcal/Mol)
Zero-point correction	0.428148 (Hartree/Particle)
Thermal correction to Energy	0.453960
Thermal correction to Enthalpy	0.454904
Thermal correction to Gibbs Free Energy	0.374265
Sum of electronic and zero-point Energies	-4945.898112
Sum of electronic and thermal Energies	-4945.872300
Sum of electronic and thermal Enthalpies	-4945.871356
Sum of electronic and thermal Free Energies	-4945.951995

cartesian

6	1.88147402	-1.40067208	2.08391380	1	0.93317401	2.69902802	-2.69308615
6	1.13177407	-2.49937201	1.56531405	1	0.89007401	4.24642801	-1.87358594
6	1.88127398	-3.07407212	0.51121402	1	2.95017409	4.17752838	1.03381395
6	3.06767416	-2.31247211	0.34661400	1	2.69927406	2.78552794	2.07831383
6	3.07547402	-1.30127203	1.34481406	1	1.42597413	3.99492812	1.88791406
1	3.85147405	-0.56127203	1.48901403	6	-2.26642585	0.56752801	0.28871399
1	1.61407399	-0.77787203	2.92681384	6	-3.45242572	-0.40177202	0.27301401
1	0.18237406	-2.86147213	1.94371402	6	-4.48212624	-0.01817201	-0.78948599
1	3.85487413	-2.49787211	-0.37418601	1	-3.10382581	-1.42947209	0.09991400
6	-0.06992596	-0.11327201	-2.37778616	1	-5.34562635	-0.68527198	-0.76498598
6	-0.71052593	-1.28997207	-1.90208602	1	-4.04972601	-0.06367201	-1.79438603
6	0.20647407	-2.36067200	-1.98598599	1	-4.84262609	1.00182796	-0.63048601
6	1.42017412	-1.85397208	-2.53708601	6	0.14627406	1.05822790	0.94181395
6	1.23227406	-0.47817200	-2.80208611	6	-1.03602600	0.07152799	1.06551397
1	1.96957397	0.19022797	-3.22628617	6	-1.38872600	-0.22967201	2.52811384
1	-0.51422590	0.86922795	-2.47368622	1	0.63817406	1.16302788	1.91371405
1	-1.73262596	-1.36397207	-1.55178595	1	-0.35752594	2.02322793	0.76781398
1	2.31087399	-2.42897201	-2.75878620	1	-0.82082599	-0.95357198	0.62931401
40	1.17717409	-0.79677200	-0.28058600	1	-2.15072584	-1.00907207	2.60301399
17	3.10797405	0.95862800	-0.79718602	1	-1.78112578	0.67912799	2.99471378
1	-3.93242574	-0.41227204	1.25611401	1	-0.51922590	-0.54887199	3.10511398
13	1.49947405	2.46732783	-0.17078601	1	-1.97552586	0.82152802	-0.73708600
6	0.62877405	3.18792796	-1.76128602	1	-2.58562589	1.51142788	0.74871403
6	2.20487404	3.44042802	1.35211396	1	-0.00322592	-3.39037204	-1.72788596
1	-0.46622592	3.14992785	-1.70858598	1	1.60607409	-3.95027208	-0.05998601

TS-4Cl

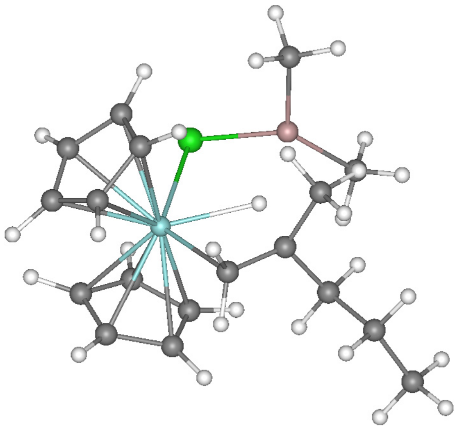

Zero-point vibrational energy	1109215.9 (Joules/Mol)
	265.10896 (Kcal/Mol)
Zero-point correction	0.422478 (Hartree/Particle)
Thermal correction to Energy	0.448209

Thermal correction to Enthalpy	0.449153
Thermal correction to Gibbs Free Energy	0.367905
Sum of electronic and zero-point Energies	-4945.877504
Sum of electronic and thermal Energies	-4945.851774
Sum of electronic and thermal Enthalpies	-4945.850829
Sum of electronic and thermal Free Energies	-4945.932077

cartesian

6	-0.65492600	-2.45834994	1.22455001	6	1.75667405	2.34835005	2.13194990
6	-2.00132608	-2.69575000	0.86074996	1	0.88317400	4.09644985	-0.99984998
6	-2.82352614	-1.88354993	1.69704986	1	-0.61442602	3.26364994	-1.41534996
6	-1.98232603	-1.12915003	2.53604984	1	-0.62872601	4.56134987	-0.22985002
6	-0.63152599	-1.45795000	2.22665000	1	1.68027401	3.16904998	2.85564995
1	0.24637401	-1.05875003	2.72144985	1	1.76037383	1.42035007	2.71224999
1	0.20387399	-2.97224998	0.81604999	1	2.73317385	2.45274997	1.64754987
1	-2.34652591	-3.41284990	0.12584996	6	0.16907400	-1.12564993	-1.39645004
1	-3.90572596	-1.85895002	1.70114994	6	1.09647393	-0.17874998	-0.89035004
1	-2.30502605	-0.41514999	3.28164983	6	1.51957393	0.96364999	-1.79135013
6	-2.29072595	0.72075003	-2.04365015	6	2.15647388	-0.63604999	0.09484997
6	-2.78782606	-0.61675000	-2.02955008	1	0.34837401	-2.16864991	-1.15534997
6	-3.75852609	-0.70515001	-1.00865006	1	-0.17562598	-0.95535004	-2.41235018
6	-3.83352613	0.55445004	-0.35625005	1	2.08567405	1.72784996	-1.25014997
6	-2.94892597	1.44115007	-1.02835011	1	2.17507386	0.54405004	-2.56165004
1	-2.79392600	2.48315001	-0.77845001	1	0.67357403	1.43445003	-2.29325008
1	-1.56482601	1.12185001	-2.74065018	6	3.35767388	-1.24994993	-0.64925003
1	-2.50552607	-1.41355002	-2.70565009	1	2.50517392	0.20535001	0.70144999
1	-4.33952570	-1.58544993	-0.76575005	1	1.74527383	-1.38265002	0.77924997
1	-4.48142576	0.81375003	0.47224995	6	4.35527372	-1.85874999	0.33544996
40	-1.56362605	-0.33604997	0.15934998	1	3.85627413	-0.47904998	-1.24425006
17	-1.66102600	1.88625002	1.84504986	1	3.00587392	-2.01615000	-1.34864998
1	0.23197401	0.71495003	0.10064997	1	5.21427393	-2.27784991	-0.19185004
13	0.26827401	2.47285008	0.89244998	1	3.89377403	-2.66225004	0.91634995
6	-0.04942602	3.70024991	-0.58475006	1	4.72647381	-1.10595000	1.03594995

I-5Cl

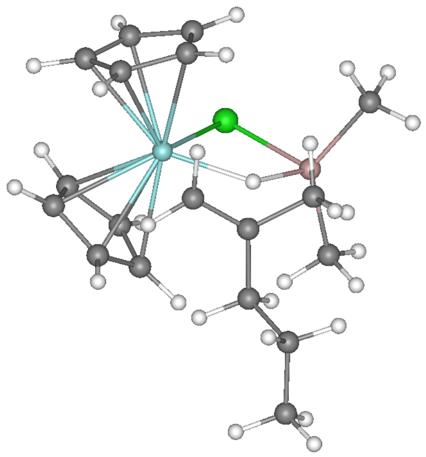

Zero-point vibrational energy	1111432.5 (Joules/Mol)
	265.63873 (Kcal/Mol)
Zero-point correction	0.423322 (Hartree/Particle)
Thermal correction to Energy	0.450254
Thermal correction to Enthalpy	0.451199
Thermal correction to Gibbs Free Energy	0.365918
Sum of electronic and zero-point Energies	-4945.888445
Sum of electronic and thermal Energies	-4945.861513
Sum of electronic and thermal Enthalpies	-4945.860569
Sum of electronic and thermal Free Energies	-4945.945849

cartesian

6	-0.52789795	-2.31204200	1.42805803	6	1.09200203	2.66735792	2.04695797
6	-1.91659796	-2.55604196	1.37955797	1	0.58060205	4.04815817	-1.36214197
6	-2.56339788	-1.54694211	2.15365791	1	-0.90289795	3.20575809	-1.80704200
6	-1.56049800	-0.70564210	2.69035792	1	-0.96549797	4.60625792	-0.74264205
6	-0.30269796	-1.14644206	2.21265793	1	1.01990199	3.62675810	2.57135797
1	0.65870202	-0.71334207	2.45875788	1	0.94660199	1.88505793	2.79995775
1	0.23340201	-2.93374205	0.97785795	1	2.12420225	2.59225798	1.68505800
1	-2.39639807	-3.38744187	0.87905800	6	0.42090201	-1.43794203	-1.52874196
1	-3.62729788	-1.46674204	2.34115791	6	1.33890212	-0.51464206	-1.13394201
1	-1.72999799	0.15235795	3.32645798	6	1.57480204	0.73045790	-1.94504201
6	-2.35969782	-0.03494206	-2.22314215	6	2.27900219	-0.73934209	0.01675797
6	-2.69549799	-1.37484205	-1.85184205	1	0.40920198	-2.43504190	-1.09844196
6	-3.70929813	-1.30594206	-0.86754203	1	-0.06849796	-1.31904209	-2.49014211
6	-3.95029783	0.05685795	-0.57584202	1	1.90080202	1.56545794	-1.31724203
6	-3.13279819	0.84055793	-1.44244206	1	2.38160181	0.53085792	-2.65954208
1	-3.10099792	1.92255795	-1.47094202	1	0.69100201	1.03335798	-2.51044202
1	-1.64979792	0.26255795	-2.98514223	6	3.74280214	-0.83174211	-0.45764205

1	-2.29869795	-2.28274202	-2.28974223	1	2.19360209	0.10035795	0.72045791
1	-4.20229816	-2.14974189	-0.40224203	1	2.02340221	-1.65804207	0.55435795
1	-4.65759802	0.44565794	0.14635795	6	4.68240213	-1.12684202	0.71005797
40	-1.60219789	-0.38744205	0.17165796	1	4.03550196	0.10845795	-0.93424201
17	-2.27229786	2.00445795	1.36645794	1	3.82700205	-1.61614203	-1.21694195
1	-0.14999795	0.92485791	0.03655796	1	5.71870232	-1.17784202	0.37095797
13	-0.19959795	2.57145810	0.59305799	1	4.43800211	-2.08214188	1.18265796
6	-0.38709795	3.70615792	-0.97954202	1	4.61680222	-0.34524205	1.47235799

S1.5. Me_3Al reaction profile

I-0Me

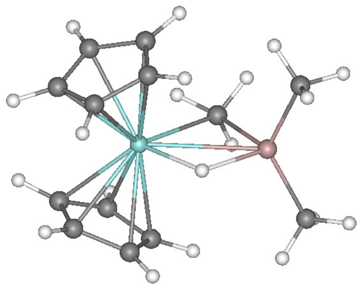

Zero-point vibrational energy	756308.5 (Joules/Mol)
	180.76206 (Kcal/Mol)
Zero-point correction	0.288063 (Hartree/Particle)
Thermal correction to Energy	0.306404
Thermal correction to Enthalpy	0.307348
Thermal correction to Gibbs Free Energy	0.241768
Sum of electronic and zero-point Energies	-4289.944549
Sum of electronic and thermal Energies	-4289.926207
Sum of electronic and thermal Enthalpies	-4289.925263
Sum of electronic and thermal Free Energies	-4289.990843

cartesian

6	1.87194288	2.07636285	-0.97703713	1	0.53124285	-2.24743700	2.15216279
6	2.60044265	1.72656274	0.18646285	1	-0.33765718	-2.96673703	-0.28883713
6	1.73514283	1.86256278	1.30386281	40	0.79464281	0.01516281	-0.07983715
6	0.47844282	2.33396292	0.83226287	6	-0.98575711	-0.15933719	-1.65943718
6	0.55694282	2.45326281	-0.57603717	1	-0.67775714	-0.04123720	1.15516281
1	-0.23205718	2.80126286	-1.23163712	13	-2.17385721	-0.05953719	0.19286285
1	2.25974274	2.09016299	-1.98973703	6	-3.00885725	-1.80603719	0.40866286
1	3.63704276	1.41396272	0.21836285	6	-3.00695729	1.69266284	0.35676286
1	1.99694288	1.68186283	2.33976293	1	-4.01125717	-1.71743727	0.83936286
1	-0.38615718	2.56306291	1.44276285	1	-3.12455726	-2.33653712	-0.54363716
6	2.69874263	-1.56183720	-0.30443716	1	-2.43865728	-2.46363711	1.07486284
6	2.35464263	-1.53013718	1.07676280	1	-3.19015718	1.94626284	1.40766287
6	1.06844282	-2.10943699	1.22216284	1	-2.42955732	2.51636291	-0.07713715
6	0.61064279	-2.49113703	-0.06833715	1	-3.98955750	1.70446277	-0.12873715
6	1.62264287	-2.16513705	-1.00583720	1	-1.27295721	-1.15063727	-2.02363706
1	1.58214283	-2.34603715	-2.07423711	1	-0.05875719	0.08756280	-2.23053718
1	3.63274264	-1.22593725	-0.73823714	1	-1.68745720	0.56886280	-2.09123707
1	2.97944283	-1.15613723	1.87866282				

I-1Me

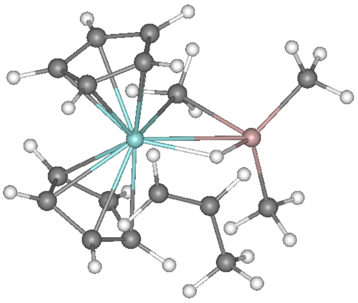

Zero-point vibrational energy	976497.1 (Joules/Mol)
	233.38840 (Kcal/Mol)
Zero-point correction	0.371928 (Hartree/Particle)
Thermal correction to Energy	0.395214
Thermal correction to Enthalpy	0.396158
Thermal correction to Gibbs Free Energy	0.321046
Sum of electronic and zero-point Energies	-4407.715485
Sum of electronic and thermal Energies	-4407.692199
Sum of electronic and thermal Enthalpies	-4407.691255
Sum of electronic and thermal Free Energies	-4407.766368

cartesian

6	1.21529317	2.30450678	0.81071818	1	-0.93450677	-0.01689314	-0.67538184
6	2.21419334	1.53380692	1.44131815	13	-2.16730666	-0.83109313	0.20821816
6	1.57749319	0.70910686	2.41421819	6	-2.83330679	-2.28829312	-0.90168184
6	0.19079325	0.99730688	2.38501835	6	-3.28770685	0.50490683	1.09581816
6	-0.04490677	1.95500696	1.36941814	1	-3.56970668	-1.93279314	-1.63078189
1	-1.00500679	2.38860679	1.11671817	1	-2.04750681	-2.80169320	-1.46698189
1	1.38589323	3.06210685	0.05921817	1	-3.34420681	-3.04949331	-0.30058181
1	3.27779317	1.58660686	1.24391818	1	-4.19280720	0.03500685	1.49871814
1	2.07429314	0.02450686	3.09171820	1	-2.81100678	1.02810693	1.93241811
1	-0.56430674	0.55330688	3.02071834	1	-3.64310670	1.27370691	0.39891815
6	1.75029325	-1.58899307	-1.64098179	6	1.17199326	1.48630691	-2.30208182
6	2.83529329	-0.86309314	-1.06218183	6	-0.17760676	1.48910689	-2.31828165
6	3.02439332	-1.34769309	0.25461817	6	-1.05470681	2.59780669	-1.82328188
6	2.03559327	-2.32319331	0.51081818	1	1.74019325	2.32980680	-1.92098176
6	1.25589323	-2.48109317	-0.67168182	1	1.72429323	0.74990684	-2.87328172
1	0.43099326	-3.16959310	-0.80648184	1	-1.51300681	3.08470678	-2.69058180
1	1.36689329	-1.48649311	-2.64888167	1	-1.87110686	2.21820688	-1.20268190
1	3.45419335	-0.12359314	-1.55478179	1	-0.49610674	3.35640669	-1.27168190
1	3.78859329	-1.02029312	0.94831812	1	-1.58510673	-1.53429306	2.20991826
1	1.92019320	-2.88619328	1.42831814	1	0.09719324	-1.64719307	2.24221826
40	0.80119324	-0.13319315	0.20031816	1	-0.77830672	-2.72949314	1.19101810
6	-0.72900677	-1.68909311	1.52671814	1	-0.68260676	0.67490685	-2.83608174

TS-1Me

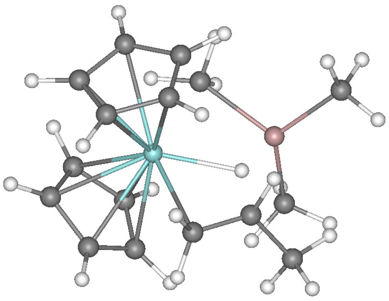

Zero-point vibrational energy	975298.2 (Joules/Mol)
	233.10186 (Kcal/Mol)
Zero-point correction	0.371471 (Hartree/Particle)
Thermal correction to Energy	0.393554
Thermal correction to Enthalpy	0.394498
Thermal correction to Gibbs Free Energy	0.322593
Sum of electronic and zero-point Energies	-4407.708612
Sum of electronic and thermal Energies	-4407.686530
Sum of electronic and thermal Enthalpies	-4407.685585
Sum of electronic and thermal Free Energies	-4407.757490

cartesian

6	-1.17124319	2.48448658	0.28614089	6	0.73735678	0.21368636	-2.15925908
6	-2.05224323	1.90878642	1.24614096	6	-0.53704315	-0.29631364	-2.42135906
6	-3.08704329	1.24798632	0.54944092	1	0.89485681	0.28268635	-0.57145911
6	-2.84084320	1.38428640	-0.84205908	1	2.85695672	-0.19271363	-1.88005912
6	-1.67134321	2.17648649	-0.99635911	1	1.84825683	-1.64531362	-2.08315897
1	-1.23754311	2.49408650	-1.93685913	1	-1.29274321	0.35598636	-2.84415913
1	-0.29034317	3.07658648	0.50254089	1	-0.59564316	-1.33971369	-2.71375918
1	-1.96544313	1.99288642	2.32174087	13	2.02805686	0.34808636	0.87544090
1	-3.92684317	0.73688638	1.00234091	6	0.49965683	0.58448637	2.22494102
1	-3.46574330	0.99908638	-1.63895905	6	2.92665672	-1.39001369	0.91324091
6	-1.83184314	-1.76481366	1.63324094	6	2.91675687	2.05048656	0.53204089
6	-2.64214325	-1.86801362	0.46884087	1	3.61145687	1.98828638	-0.31335911
6	-1.83134317	-2.32191348	-0.59095907	1	2.22685671	2.87568641	0.32404089
6	-0.51214314	-2.51701355	-0.08405910	1	3.52115679	2.35828638	1.39364088
6	-0.52454317	-2.19951344	1.29124093	1	3.86785674	-1.36251366	0.35314089
1	0.32635686	-2.25181341	1.95974088	1	3.19495678	-1.67191362	1.93954086
1	-2.17154312	-1.46571362	2.61774087	1	2.34265685	-2.22381353	0.50824088
1	-3.70094323	-1.65311360	0.40564087	1	1.21485686	0.10158636	2.91954088
1	-2.17024302	-2.52531362	-1.59775913	1	0.52415681	1.65468633	2.45334101
1	0.33765683	-2.89011359	-0.64345908	1	-0.44134316	0.20198636	2.62354088
40	-0.96274316	-0.03001363	0.04884089	1	2.18425679	-0.59471363	-3.46585917
6	1.98415685	-0.60631365	-2.38955903	1	0.87795687	1.29318631	-2.22185898

I-2Me_a

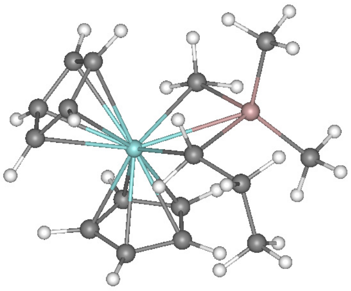

Zero-point vibrational energy	988223.4 (Joules/Mol)
	236.19106 (Kcal/Mol)
Zero-point correction	0.376394 (Hartree/Particle)
Thermal correction to Energy	0.398577
Thermal correction to Enthalpy	0.399521
Thermal correction to Gibbs Free Energy	0.327728
Sum of electronic and zero-point Energies	-4407.738140
Sum of electronic and thermal Energies	-4407.715958
Sum of electronic and thermal Enthalpies	-4407.715014
Sum of electronic and thermal Free Energies	-4407.786807

cartesian

6	-1.66079998	1.92719996	-1.37240005	6	3.15790009	0.67970002	-1.43840003
6	-2.06949997	2.16249990	-0.03370000	1	3.54579997	-2.72350001	-0.00020000
6	-0.91219997	2.41890001	0.73920000	1	2.92630005	-2.98379993	-1.61539996
6	0.21430001	2.37820005	-0.13230000	1	1.90009999	-3.30150008	-0.22260000
6	-0.24730000	2.07940006	-1.43210006	1	2.71280003	1.67949998	-1.49199998
1	0.36690000	1.98959994	-2.31979990	1	3.44449997	0.41530001	-2.46499991
1	-2.31739998	1.71809995	-2.20930004	1	4.10330009	0.78240001	-0.89279997
1	1.24039996	2.56640005	0.14900000	6	1.26370001	-0.42309999	1.28069997
6	-2.25149989	-1.92060006	-0.55269998	6	2.62809992	0.01370000	1.89479995
6	-3.06229997	-0.89109999	-0.02120000	6	2.88809991	1.51590002	1.91589999
6	-2.69039989	-0.70039999	1.34070003	1	0.52460003	0.20100001	1.84840000
6	-1.65970004	-1.62489998	1.64100003	1	1.08669996	-1.44830000	1.62399995
6	-1.36919999	-2.36360002	0.46799999	1	3.45849991	-0.49300000	1.38590002
1	-0.62540001	-3.14549994	0.37009999	1	3.78940010	1.73130000	2.49410009
1	-2.29710007	-2.30629992	-1.56369996	1	2.06019998	2.05430007	2.39050007
1	-3.84629989	-0.36149999	-0.54830003	1	3.04480004	1.91960001	0.91380000
40	-0.73559999	0.03820000	-0.02920000	1	-1.17209995	-1.73549998	2.60209990
1	-3.08850002	2.14730000	0.33199999	1	-0.89200002	2.64870000	1.79869998
1	-3.14339995	-0.00460000	2.03640008	1	0.23170000	-2.00000000	-2.10069990
6	0.26240000	-0.91189998	-1.99090004	1	0.97570002	-0.52160001	-2.73210001
13	2.04410005	-0.75529999	-0.70889997	1	-0.68980002	-0.55260003	-2.42389989
6	2.65070009	-2.60929990	-0.62260002	1	2.65510011	-0.36849999	2.91969991

I-2Me_bi

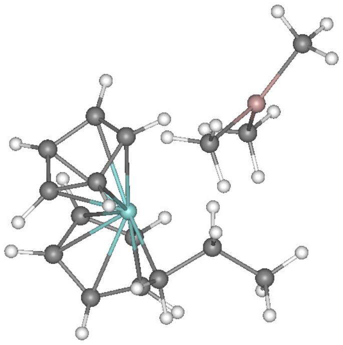

Zero-point vibrational energy	983941.6 (Joules/Mol)
	235.16768 (Kcal/Mol)
Zero-point correction	0.374764 (Hartree/Particle)
Thermal correction to Energy	0.398247
Thermal correction to Enthalpy	0.399191
Thermal correction to Gibbs Free Energy	0.319781
Sum of electronic and zero-point Energies	-4407.722020
Sum of electronic and thermal Energies	-4407.698537
Sum of electronic and thermal Enthalpies	-4407.697592
Sum of electronic and thermal Free Energies	-4407.777003

cartesian

6	2.30430007	1.86909997	-0.94700003	1	0.02810000	1.63789999	1.23450005
6	2.74790001	0.67570001	-1.56710005	13	-3.87030005	0.08950000	-0.22380000
6	1.70589995	0.21060000	-2.41759992	6	-4.35699987	-1.76950002	0.08350000
6	0.61269999	1.09410000	-2.29299998	6	-4.99149990	1.62890005	-0.58870000
6	0.97799999	2.11820006	-1.37390006	1	-5.43720007	-1.93649995	0.05000000
1	0.36730000	2.96639991	-1.08780003	1	-4.00869989	-2.12220001	1.06200004
1	2.88150001	2.48490000	-0.27100000	1	-3.90750003	-2.43600011	-0.66310000
1	3.72449994	0.22310001	-1.44560003	1	-6.05569983	1.38230002	-0.62730002
1	1.74460006	-0.66570002	-3.05229998	1	-4.72690010	2.09829998	-1.54330003
1	-0.33019999	1.01680005	-2.82069993	1	-4.86049986	2.40240002	0.17700000
6	0.64859998	-2.08949995	1.44089997	6	1.98780000	0.74839997	1.75899994
6	1.96949995	-2.10529995	0.91119999	6	0.84780002	1.73169994	2.00340009
6	1.87399995	-2.29270005	-0.48550001	6	0.15090001	1.58070004	3.35829997
6	0.49360001	-2.38829994	-0.82880002	1	2.95810008	1.22790003	1.64510000
6	-0.25170001	-2.29859996	0.37250000	1	2.05089998	0.01730000	2.56640005
1	-1.33120000	-2.36209989	0.45390001	1	-0.68470001	2.27659988	3.46300006
1	0.38499999	-1.97880006	2.48629999	1	0.86040002	1.77260005	4.16569996
1	2.88619995	-2.00629997	1.47720003	1	-0.23170000	0.56300002	3.48790002
1	2.70670009	-2.37400007	-1.17240000	1	-1.65369999	1.46519995	-0.30599999

1	0.09210000	-2.56719995	-1.81889999	1	-1.62220001	0.07610000	0.88270003
40	0.89340001	-0.03970000	-0.05350000	1	-1.48500001	-0.25080001	-0.91409999
6	-1.87919998	0.41180000	-0.13100000	1	1.17700005	2.76209998	1.84119999

I-2Me_bo

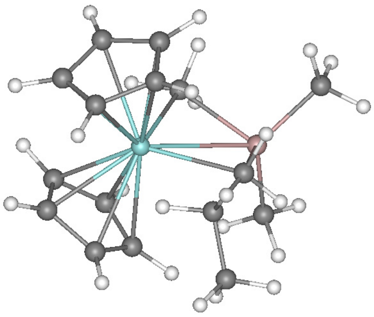

Zero-point vibrational energy	990513.4 (Joules/Mol)
	236.73838 (Kcal/Mol)
Zero-point correction	0.377267 (Hartree/Particle)
Thermal correction to Energy	0.399244
Thermal correction to Enthalpy	0.400188
Thermal correction to Gibbs Free Energy	0.328627
Sum of electronic and zero-point Energies	-4407.740304
Sum of electronic and thermal Energies	-4407.718327
Sum of electronic and thermal Enthalpies	-4407.717383
Sum of electronic and thermal Free Energies	-4407.788944

cartesian

6	1.81025004	-1.84350681	-1.33039093	6	-2.93874979	-2.28250670	0.10210910
6	2.87745023	-0.96920681	-0.98009092	1	-3.25204992	1.68839324	0.22940910
6	3.13735008	-1.13940680	0.40530911	1	-2.56874990	1.61699319	1.84880900
6	2.21115017	-2.07810688	0.91280907	1	-4.02355003	0.72079319	1.46250916
6	1.38884997	-2.51450682	-0.16459090	1	-3.46894979	-2.68030667	0.97530907
1	0.58725011	-3.24040675	-0.10249090	1	-2.24534988	-3.06560683	-0.22799090
1	1.39125013	-1.96950674	-2.32099080	1	-3.69334984	-2.17490673	-0.68569088
1	3.43315005	-0.33320680	-1.65939093	6	-0.62824988	1.90789318	-2.70649076
1	2.15575004	-2.42750669	1.93640900	6	-0.96934992	-0.26360682	-1.36899090
6	0.05075008	2.28669333	0.72270912	6	-0.04044992	0.56379318	-2.27699089
6	1.27375007	2.44809318	0.01310910	1	0.23055008	-0.02480681	-3.15729094
6	2.31675005	1.91159320	0.79920912	1	-1.02144980	-1.29450679	-1.73029089
6	1.74664998	1.42759323	2.01150918	1	-1.97174978	0.16179320	-1.53119087
6	0.35485008	1.68639326	1.96880913	1	0.97465008	0.78549320	-1.84809089
1	-0.35774994	1.45909321	2.75130916	1	-0.96114987	2.49669313	-1.84829092
1	-0.92854995	2.62649322	0.41110909	1	-1.49615002	1.73639321	-3.34749079

1	1.38985014	2.91549325	-0.95769089	1	3.36695004	1.89919317	0.53590906
1	2.28995013	0.98279315	2.83730912	1	3.91155005	-0.64010686	0.97410911
40	0.86915010	-0.05340681	0.20040910	1	0.32335010	-0.96310681	2.53970909
6	-0.54454988	-1.17110682	1.90180922	1	-0.60974991	-2.25980687	1.82470918
13	-2.06344986	-0.59000683	0.51100910	1	-1.36084986	-0.85210681	2.57420921
6	-3.04274988	1.01209319	1.06810904	1	0.09575009	2.49749327	-3.27329087

I-4Me_a

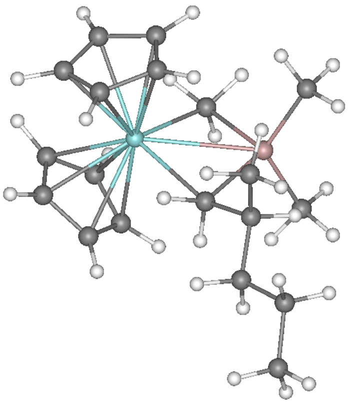

Zero-point vibrational energy	1214479.1 (Joules/Mol)
	290.26747 (Kcal/Mol)
Zero-point correction	0.462571 (Hartree/Particle)
Thermal correction to Energy	0.488850
Thermal correction to Enthalpy	0.489795
Thermal correction to Gibbs Free Energy	0.408318
Sum of electronic and zero-point Energies	-4525.541047
Sum of electronic and thermal Energies	-4525.514768
Sum of electronic and thermal Enthalpies	-4525.513823
Sum of electronic and thermal Free Energies	-4525.595301

cartesian

6	3.68165469	1.04619050	1.27288687	1	-0.07034528	3.89369059	-1.44131315
6	3.30075479	0.02129056	2.17718673	1	-1.43244529	3.37219048	-0.48331314
6	1.96095467	0.25649056	2.56698680	6	-2.88064528	-1.00580943	0.45018685
6	1.52265477	1.45279050	1.92898679	6	-0.52264529	-0.27410945	-0.04671317
6	2.58315468	1.94009042	1.13518679	6	-1.95584512	0.21909055	0.33348686
1	2.56505466	2.83849049	0.53068686	6	-1.96624517	1.07329059	1.59948683
1	4.65525436	1.15379047	0.80818683	1	-0.17024529	-0.75060946	0.90638685
1	0.55525476	1.91749048	2.05418682	1	-0.66624528	-1.10710943	-0.74451315
6	3.13125491	-1.70420945	-1.57491314	1	-2.36204529	0.83139056	-0.48751318
6	3.67055488	-1.99910951	-0.30091316	1	-2.98474526	1.37039053	1.85768688
6	2.65125489	-2.62810946	0.47038680	1	-1.55784512	0.51259053	2.45008683

6	1.49275470	-2.72320938	-0.33891320	1	-1.39434528	1.99439049	1.47138679
6	1.77815485	-2.13200951	-1.59441316	1	-2.83414531	-1.56380951	-0.49391317
1	1.09455466	-2.04220939	-2.43031335	1	-2.48724532	-1.67580950	1.22888684
1	3.65915489	-1.23150945	-2.39371324	1	0.54705477	-3.15880942	-0.03841317
1	4.68705463	-1.80790949	0.02048683	1	1.38455474	-0.34510943	3.26078677
40	1.90465474	-0.26570946	0.11468682	6	-4.34614563	-0.68260950	0.75018686
1	3.92595482	-0.79810947	2.50868678	1	1.98875475	0.69179052	-2.78671336
1	2.75435472	-3.00700951	1.47978687	1	1.95465469	2.24529052	-1.93591321
6	2.06885481	1.15929055	-1.80091321	1	3.14225483	1.10259056	-1.54141319
13	-0.09464526	1.36059058	-1.37651312	6	-5.23174524	-1.92240942	0.62518686
6	-0.87114531	0.75739056	-3.06461334	1	-4.69654560	0.09299055	0.05868683
6	-0.37304527	3.16649055	-0.67611319	1	-4.44424534	-0.27290946	1.75968683
1	-1.96664524	0.79429054	-3.05541325	1	-6.27254534	-1.68920946	0.85858685
1	-0.55614525	1.39359057	-3.90001321	1	-4.90284538	-2.70780945	1.31198680
1	-0.59354526	-0.26970944	-3.33071327	1	-5.19804525	-2.32850957	-0.38971317
1	0.17175475	3.43549061	0.23588683				

I-4Me_bi

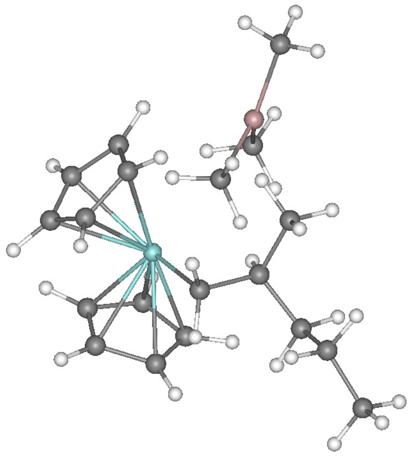

Zero-point vibrational energy	1209446.8 (Joules/Mol)
	289.06473 (Kcal/Mol)
Zero-point correction	0.460654 (Hartree/Particle)
Thermal correction to Energy	0.487280
Thermal correction to Enthalpy	0.488224
Thermal correction to Gibbs Free Energy	0.403106
Sum of electronic and zero-point Energies	-4525.525096
Sum of electronic and thermal Energies	-4525.498470
Sum of electronic and thermal Enthalpies	-4525.497526
Sum of electronic and thermal Free Energies	-4525.582644

cartesian

6	-1.59097171	-1.47669435	-2.02832079	1	3.79692841	2.87390566	1.91007924

6	-1.06317163	-2.77479434	-1.82632077	1	4.59122801	1.33650565	2.18707919
6	0.32752836	-2.73589420	-2.13322091	1	5.49582815	2.29030561	-2.03972077
6	0.65822840	-1.41229439	-2.48772073	1	4.89062834	0.67850566	-2.40752077
6	-0.52587163	-0.62429434	-2.40782094	1	3.89062834	2.07950568	-2.74352074
1	-0.60977161	0.42930564	-2.64932084	6	-1.97667158	-0.63629436	0.79827923
1	-2.62897158	-1.19469428	-1.92402077	6	-1.49557161	0.79480565	0.58047920
1	-1.63087165	-3.65119433	-1.53892076	6	-1.10677171	1.50770569	1.88297927
1	1.01022840	-3.57579422	-2.10592079	6	-2.44247150	1.63610578	-0.29682076
1	1.63952827	-1.05979431	-2.78252077	1	-2.90017152	-0.86239433	0.26717925
6	0.31092834	-1.98219430	2.42617917	1	-2.11577153	-0.86359429	1.85567927
6	-0.43157163	-3.03509426	1.82177925	1	-0.74937165	2.52380562	1.68857932
6	0.40792835	-3.67109442	0.88027924	1	-1.96827161	1.56940579	2.55227923
6	1.66222835	-2.99949431	0.87597924	1	-0.31707165	0.96810567	2.41727924
6	1.60212839	-1.97759426	1.86097932	6	-3.75697160	2.00450563	0.39467925
1	2.40882850	-1.30529428	2.12827921	1	-1.93037164	2.55660582	-0.60572076
1	-0.04947165	-1.31879437	3.20257926	1	-2.66507149	1.07830572	-1.21582067
1	-1.45177162	-3.31059432	2.05567908	1	1.47172832	0.86810565	-0.99352080
1	0.14562836	-4.52999449	0.27597925	1	1.65512836	0.65030575	0.81677920
1	2.52822852	-3.27059436	0.28427926	1	2.41192842	-0.55119431	-0.34962076
40	-0.00957164	-1.38149428	-0.04322076	6	-4.72307158	2.69830561	-0.56522077
6	2.08762836	0.48440567	-0.17522076	1	-3.55397153	2.66660571	1.24237931
1	-0.54257166	0.80130565	-0.03252076	1	-4.22387171	1.10190570	0.80537921
13	3.82432842	1.51160574	-0.24552077	1	-5.64877176	2.97760582	-0.05812076
6	4.49052811	2.15260577	1.46157932	1	-4.98387194	2.04530573	-1.40322077
6	4.60052824	1.66300559	-2.01992083	1	-4.28027201	3.60980582	-0.97702080
1	5.46452808	2.64290571	1.38367927				

I-4Me_bo

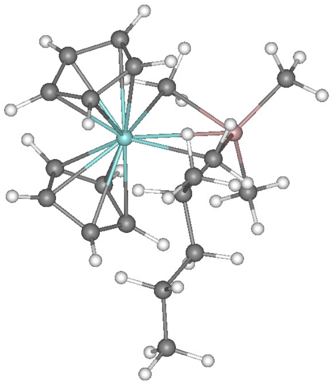

Zero-point vibrational energy	1215723.6 (Joules/Mol)
	290.56491 (Kcal/Mol)
Zero-point correction	0.463045 (Hartree/Particle)
Thermal correction to Energy	0.489087
Thermal correction to Enthalpy	0.490031

Thermal correction to Gibbs Free Energy	0.409666
Sum of electronic and zero-point Energies	-4525.542152
Sum of electronic and thermal Energies	-4525.516110
Sum of electronic and thermal Enthalpies	-4525.515166
Sum of electronic and thermal Free Energies	-4525.595531

cartesian

6	1.77506614	-1.37285662	2.06002069	1	2.92746615	3.83044338	1.07942069
6	1.02806616	-2.47475672	1.55212069	1	2.52306604	2.50444341	2.16422081
6	1.74676621	-3.02325654	0.46402076	1	1.40196621	3.84474325	1.93952060
6	2.92506599	-2.25335670	0.28152072	6	-2.54303384	0.38414335	0.41672072
6	2.94876599	-1.25155663	1.28652072	6	-3.66523409	-0.65765667	0.38882074
1	3.73386598	-0.52405667	1.44092071	6	-4.76123381	-0.28335667	-0.60867923
1	1.52526617	-0.76355666	2.91852069	1	-3.25863385	-1.64815664	0.14062074
1	0.09166616	-2.85065651	1.94842076	1	-5.57543373	-1.01025665	-0.59447926
1	3.69736600	-2.43525672	-0.45647928	1	-4.36833382	-0.23665665	-1.62947929
6	-0.34913382	0.00744335	-2.29927921	1	-5.18343401	0.69704336	-0.37157926
6	-0.89033377	-1.24535656	-1.90287924	6	-0.14463383	0.98794335	1.03562069
6	0.10056616	-2.23515654	-2.08347917	6	-1.26303387	-0.06825665	1.13772070
6	1.26086617	-1.60105658	-2.61277938	6	-1.55663383	-0.45435664	2.59362078
6	0.96886611	-0.22485664	-2.76567936	1	0.33546615	1.10814345	2.01122069
1	1.63766623	0.52434331	-3.16857934	1	-0.67073381	1.93784332	0.84562075
1	-0.86583376	0.95834333	-2.31727934	1	-0.99373376	-1.05235660	0.64742076
1	-1.89533389	-1.41935658	-1.53807926	1	-2.25593400	-1.29185665	2.65182066
1	2.18346620	-2.09525657	-2.89357924	1	-2.00443387	0.40184331	3.10722065
40	0.99916613	-0.72885668	-0.27467927	1	-0.65173388	-0.73065668	3.13782072
6	2.79956603	0.92204338	-0.74897921	1	-2.29943395	0.69704336	-0.60477924
1	-4.10203409	-0.75635666	1.38692069	1	-2.90543389	1.28614342	0.92592078
13	1.26466620	2.25484347	-0.07657926	1	-0.02493384	-3.29405665	-1.89967930
6	0.41946617	3.18414330	-1.58067930	1	1.46196616	-3.89415669	-0.11067926
6	2.10506606	3.18754339	1.41422069	1	3.23006606	0.05444334	-1.26507926
1	-0.67233384	3.24864340	-1.48887932	1	3.52276611	1.17984343	0.02932074
1	0.62906617	2.75924349	-2.56927919	1	2.88266611	1.69594336	-1.52997935
1	0.77526617	4.22074318	-1.61427927				

TS-4Me

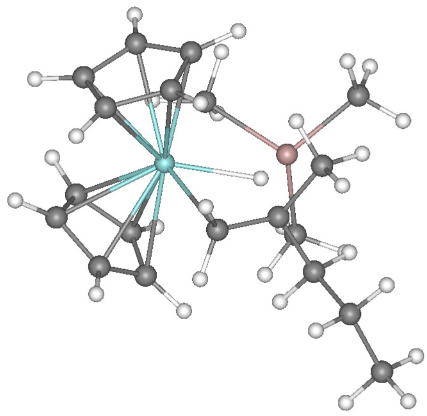

Zero-point vibrational energy	1201249.4 (Joules/Mol)
	287.10549 (Kcal/Mol)
Zero-point correction=	0.457532 (Hartree/Particle)
Thermal correction to Energy=	0.483782
Thermal correction to Enthalpy=	0.484726
Thermal correction to Gibbs Free Energy=	0.403324
Sum of electronic and zero-point Energies=	-4525.515384
Sum of electronic and thermal Energies=	-4525.489134
Sum of electronic and thermal Enthalpies=	-4525.488190
Sum of electronic and thermal Free Energies=	-4525.569592

cartesian

6	2.24909449	1.15650380	1.92583585	1	-1.93640554	-0.16109625	3.01843596
6	3.33119440	1.14360380	0.99923581	1	-0.33690554	0.51560372	0.18973584
6	3.84129429	-0.16699624	0.93233585	1	-2.70150566	0.34430376	-0.10066415
6	3.07809448	-0.98299628	1.81463587	1	-2.05660534	-1.16969621	-0.74026418
6	2.12889433	-0.14939624	2.45473599	1	0.19129446	-1.86269617	2.15253592
1	1.43989444	-0.46299624	3.22663593	1	-0.48270553	-2.48259640	0.57113582
1	1.67099452	2.02680373	2.21433592	13	-0.45960552	1.96280372	-0.93646419
1	3.71229458	1.99740374	0.45573583	6	1.56749451	2.11490369	-1.21106410
1	4.67819452	-0.48929623	0.32603583	6	-1.46570551	1.35080385	-2.50176406
1	3.23599434	-2.03679633	2.01023579	6	-1.04970551	3.37360358	0.27703583
6	2.73649454	-0.88939625	-2.11686420	1	-2.11950541	3.31690359	0.50683582
6	2.77659464	-2.05529618	-1.30436409	1	-0.50790548	3.38330364	1.22973585
6	1.46289444	-2.55089641	-1.18276405	1	-0.89280558	4.35820389	-0.18066417
6	0.60209447	-1.70329618	-1.93976414	1	-2.53070545	1.59120381	-2.40796399
6	1.39329445	-0.69599628	-2.53206420	1	-1.12150550	1.85730386	-3.41296411
1	1.03209448	0.09690376	-3.17606401	1	-1.40330553	0.27570376	-2.70306420
1	3.59099436	-0.29299623	-2.41366410	1	1.26069450	2.57320356	-2.17156410
1	3.66089439	-2.49469638	-0.86086416	1	1.83859444	2.94340372	-0.54926413

1	1.17479444	-3.44059634	-0.63886416	1	2.49999452	1.63650382	-1.51556408
1	-0.46280551	-1.83849621	-2.08126402	6	-3.46680546	-1.41899621	0.87783581
40	1.49989450	-0.29679623	-0.01916416	6	-4.64610529	-1.62739623	-0.07156416
6	-2.33080530	-0.64809626	0.18193583	1	-3.80130529	-0.86369628	1.75903594
6	-1.12540555	-0.52999622	1.09763587	1	-3.09070539	-2.38489628	1.23103595
6	-0.23230553	-1.62129617	1.18323588	1	-5.45990562	-2.15399623	0.43033585
6	-1.32830548	0.38430378	2.28883600	1	-4.35100555	-2.21739626	-0.94356418
1	-0.39450550	0.66130376	2.77623582	1	-5.03610563	-0.67049628	-0.42886415
1	-1.86310542	1.29470384	2.00963593				

I-5Me

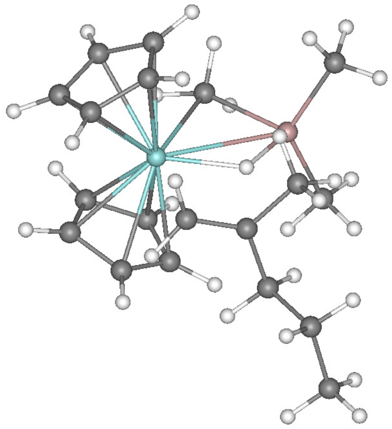

Zero-point vibrational energy	1201249.4 (Joules/Mol)
	287.10549 (Kcal/Mol)
Zero-point correction	0.457532 (Hartree/Particle)
Thermal correction to Energy	0.483782
Thermal correction to Enthalpy	0.484726
Thermal correction to Gibbs Free Energy	0.403324
Sum of electronic and zero-point Energies	-4525.515384
Sum of electronic and thermal Energies	-4525.489134
Sum of electronic and thermal Enthalpies	-4525.488190
Sum of electronic and thermal Free Energies	-4525.569592

cartesian

6	0.30487162	-2.28994155	-1.48408866	1	0.85947156	3.17795849	1.83051133
6	1.69587159	-2.51814151	-1.53198874	1	0.95997161	4.47655869	0.64471132
6	2.29237151	-1.43084145	-2.23328876	1	-0.80872840	3.47915840	-2.54448867
6	1.25467157	-0.55114150	-2.63038874	1	-0.74502844	1.75395846	-2.87728858
6	0.02807161	-1.05854154	-2.13998866	1	-1.97892833	2.41205835	-1.80658865
1	-0.94792837	-0.61384153	-2.29038858	6	-0.63732845	-1.68244147	1.56531131
1	-0.42592841	-2.96174169	-1.05588865	6	-1.52532840	-0.69794154	1.27081132
1	2.20957160	-3.38444161	-1.13458872	6	-1.69662833	0.48975846	2.17971134

1	3.34557176	-1.32624149	-2.46698880	6	-2.50172830	-0.80874157	0.13281132
1	1.37557161	0.36315849	-3.19708872	1	-0.66652840	-2.63564157	1.04611135
6	2.15797162	-0.37384152	2.32601142	1	-0.11172837	-1.66414154	2.51421142
6	2.49117160	-1.66884148	1.82541132	1	-1.96742845	1.39005852	1.62031126
6	3.52667165	-1.50834155	0.87421131	1	-2.51522827	0.28115848	2.87751126
6	3.79007173	-0.12674153	0.73841131	1	-0.79952842	0.69545853	2.76711130
6	2.95227170	0.57245851	1.65541136	6	-3.94942856	-0.94394147	0.64341134
1	2.93167162	1.64295852	1.81531131	1	-2.43412828	0.09035848	-0.49508870
1	1.43597162	-0.15004152	3.10101128	1	-2.26522827	-1.67624152	-0.49258870
1	2.08087158	-2.61364150	2.16051126	1	1.95887160	2.37875843	-1.94058871
1	4.02497149	-2.30414152	0.33541131	1	2.99087167	1.12015855	-1.49088871
1	4.53597164	0.31785849	0.09161133	1	2.77777171	2.44255829	-0.37728870
40	1.42487168	-0.46704152	-0.10498868	6	-4.92972851	-1.13254154	-0.51248866
6	2.23337150	1.77715850	-1.05478871	1	-4.22352839	-0.05194152	1.21451128
1	-0.05562842	0.76265848	0.15231133	1	-4.00862837	-1.79464149	1.33041131
13	0.24547160	2.30535841	-0.52528870	1	-5.95242834	-1.22724152	-0.14268868
6	0.37977159	3.59305835	0.93611133	1	-4.69602823	-2.03454161	-1.08478868
6	-0.92052835	2.49005842	-2.08458877	1	-4.89842844	-0.27994153	-1.19678867
1	-0.60292834	3.96475816	1.24671125				

S3.6. $^{i}Bu_2AlH$ reaction profile

I-0H

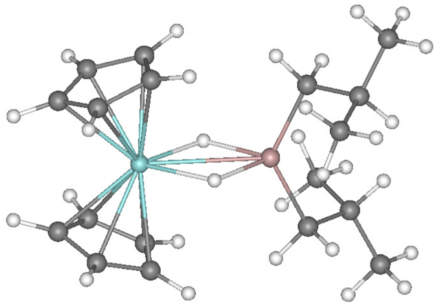

Zero-point vibrational energy	1134721.8 (Joules/Mol)
	271.20502 (Kcal/Mol)
Zero-point correction	0.432193 (Hartree/Particle)
Thermal correction to Energy	0.456539
Thermal correction to Enthalpy	0.457484
Thermal correction to Gibbs Free Energy	0.373872
Sum of electronic and zero-point Energies	-4486.266924
Sum of electronic and thermal Energies	-4486.242577
Sum of electronic and thermal Enthalpies	-4486.241633
Sum of electronic and thermal Free Energies	-4486.325245

cartesian

6	-3.42163610	1.52515006	1.65658605	6	1.56366396	-1.64485002	-0.57961398
6	-4.19173622	1.57825005	0.46678603	1	2.01516390	1.50534999	1.59268606
6	-3.38523602	2.14665008	-0.55361396	6	2.63566399	2.12145019	-0.39971399
6	-2.12053609	2.46795011	0.01268600	1	0.82786393	2.46835017	0.73948604
6	-2.14083600	2.08075023	1.37868607	6	2.65516400	-2.11554980	0.40668601
1	-1.33253598	2.20485020	2.08898592	1	0.83746397	-2.46024990	-0.71701401
1	-3.76123595	1.15495002	2.61738586	1	2.00936389	-1.47885001	-1.57081401
1	-5.22033596	1.25744998	0.36018598	6	3.32476401	3.40105009	0.08018599
1	-3.69143605	2.33525014	-1.57631397	6	2.02676392	2.33135009	-1.78921402
1	-1.29273605	2.93685007	-0.50481397	1	3.39986396	1.33685005	-0.48091400
6	-4.17973614	-1.59614992	-0.48251402	6	3.33596396	-3.39364982	-0.08861400
6	-3.39493608	-1.47025001	-1.65911400	6	2.06616402	-2.33034992	1.80408597
6	-2.11723614	-2.03914976	-1.39881396	1	3.42106414	-1.33194995	0.47988600
6	-2.10863590	-2.49894977	-0.05541400	1	4.13176441	-3.71154976	0.59128600
6	-3.38363600	-2.21944976	0.51158601	1	3.77446389	-3.24894977	-1.07951403
1	-3.70063615	-2.46644974	1.51858604	1	2.60736394	-4.20924997	-0.15901400
1	-5.21123600	-1.28684998	-0.36951399	1	4.11016369	3.71585011	-0.61321402
1	-3.72553611	-1.04855001	-2.60151410	1	3.77836394	3.25945020	1.06478596
1	-1.29963613	-2.12004995	-2.10451412	1	2.59776402	4.21725035	0.15898600
1	-1.28463602	-2.99284983	0.44478601	1	2.76966405	2.69105005	-2.50621414

40	-2.31443596	-0.01794996	0.04678600	1	1.22226393	3.07625008	-1.74611402
1	-0.70073605	-0.34654996	1.09818602	1	1.60496402	1.40455008	-2.20351410
1	-0.72603607	0.32815003	-1.01891398	1	2.81736398	-2.69884992	2.50778580
13	0.70166397	-0.00144996	0.02798600	1	1.25566399	-3.06924987	1.76898599
6	1.55806398	1.65614998	0.60418600	1	1.65686393	-1.40324998	2.23028588

I-0H_bo

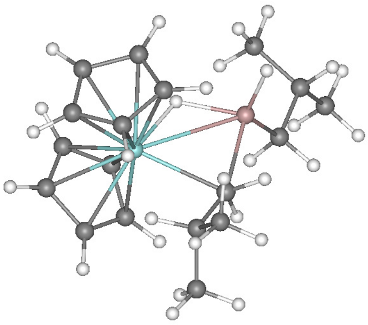

Zero-point vibrational energy	1138711.1 (Joules/Mol)
	272.15848 (Kcal/Mol)
Zero-point correction	0.433712 (Hartree/Particle)
Thermal correction to Energy	0.457216
Thermal correction to Enthalpy	0.458160
Thermal correction to Gibbs Free Energy	0.381281
Sum of electronic and zero-point Energies	-4486.265712
Sum of electronic and thermal Energies	-4486.242208
Sum of electronic and thermal Enthalpies	-4486.241264
Sum of electronic and thermal Free Energies	-4486.318143

cartesian

6	-1.97790194	-0.45687792	-2.45889997	1	0.41819799	1.82142198	-0.71950006
6	-2.36900187	-1.74417794	-2.01889992	13	1.30609798	0.27172208	1.02049994
6	-1.19210196	-2.47617793	-1.70300007	1	1.19829798	0.27852207	2.59529996
6	-0.07340199	-1.64467788	-1.96970010	6	2.90639782	0.55772209	-0.05730003
6	-0.55660200	-0.38967791	-2.41799998	6	4.13219786	-0.19927791	0.49449998
1	0.05639800	0.44882208	-2.72449994	1	2.72229815	0.23362209	-1.09460008
1	-2.64690208	0.32812208	-2.79250002	1	3.13419819	1.63102210	-0.12110004
1	-3.38490200	-2.11357784	-1.95940006	6	5.36079788	0.00892209	-0.39370003
1	-1.15020204	-3.50357795	-1.36380005	6	3.82539797	-1.69227791	0.64399993
1	0.96659803	-1.92247796	-1.85520005	1	4.36229801	0.19882210	1.49099994
6	-3.34920216	-1.56407785	1.09549999	1	6.24329805	-0.48397791	0.02439997
6	-2.26350212	-2.43537807	1.39629996	1	5.58929777	1.07212210	-0.50770009
6	-1.35010195	-1.73037791	2.21670008	1	5.18429804	-0.40657791	-1.39220011
6	-1.85020196	-0.41297793	2.40400004	1	4.69749784	-2.25147796	0.99409997
6	-3.09980202	-0.32517791	1.73219991	1	3.52249813	-2.12077785	-0.31960005

1	-3.75660181	0.53662211	1.71409988	1	3.01759815	-1.87797785	1.36669993
1	-4.23260212	-1.81667793	0.52269995	6	-2.23670197	2.84602189	1.03469992
1	-2.17250204	-3.46857810	1.08589995	1	-2.39850187	1.21772206	-0.31280002
1	-0.43360201	-2.12287807	2.63779998	6	-1.74220204	2.87582207	-1.43660009
1	-1.38120198	0.35292208	3.00920010	1	-3.29870176	3.05372190	0.87940001
40	-1.32710195	-0.62057793	-0.03880003	1	-1.71610200	3.80462217	1.11459994
6	-0.21890199	1.56532204	0.13299996	1	-2.11630201	2.32242203	1.98529994
1	0.44219798	-1.14207792	0.48499995	1	-2.77960205	3.14052200	-1.65860009
1	0.13989800	2.15762186	0.99210000	1	-1.32950199	2.33782196	-2.29340005
6	-1.64920199	2.05882192	-0.14190003	1	-1.17260194	3.80152178	-1.31730008

I-1H

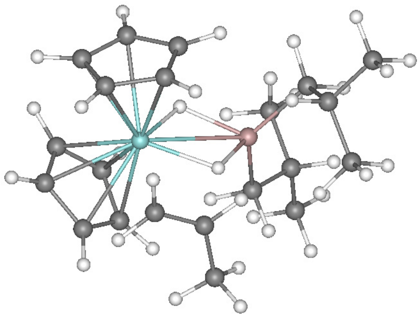

Zero-point vibrational energy	1356959.8 (Joules/Mol)
	324.32118 (Kcal/Mol)
Zero-point correction	0.516839 (Hartree/Particle)
Thermal correction to Energy	0.545555
Thermal correction to Enthalpy	0.546499
Thermal correction to Gibbs Free Energy	0.457341
Sum of electronic and zero-point Energies	-4604.058621
Sum of electronic and thermal Energies	-4604.029905
Sum of electronic and thermal Enthalpies	-4604.028960
Sum of electronic and thermal Free Energies	-4604.118119

cartesian

6	-2.65861034	-2.27949309	-1.15430343	1	-1.47221017	-0.96759319	-3.49750352
6	-3.45611024	-2.30199313	0.01249658	1	-1.08941019	1.79580677	-2.11910343
6	-2.60261035	-2.60389328	1.11369658	13	1.09728980	-0.07769318	0.39239657
6	-1.28681016	-2.76279330	0.61819655	6	2.11408973	-1.61049318	-0.27930343
6	-1.31081021	-2.52939320	-0.78490341	6	1.79778981	1.65520680	0.98199660
1	-0.46201020	-2.59639311	-1.45540345	1	2.23378968	-1.52379322	-1.37040341
1	-3.02061033	-2.12679315	-2.16210341	6	3.50418997	-1.71139324	0.38369656
1	-4.52941036	-2.16099310	0.05559658	1	1.58298969	-2.56079316	-0.11810343
1	-2.90961027	-2.72909307	2.14439654	6	1.35778975	2.80770683	0.05409658
1	-0.41591018	-3.02469325	1.20469654	1	1.52228975	1.87780678	2.02229667

6	-2.66781020	1.99760675	0.90239662	1	2.89738965	1.61190677	0.98369658
6	-3.81021023	1.14790678	1.00709653	6	4.29988956	-2.89659309	-0.16820343
6	-3.59481025	0.27150682	2.09809661	6	3.36448979	-1.82219315	1.90479648
6	-2.31231022	0.53610682	2.63329649	1	4.06448984	-0.79389322	0.15979658
6	-1.74871016	1.62240684	1.90039659	6	1.83868980	2.55570674	-1.37890351
1	-0.77971017	2.07340670	2.07719660	6	1.86488986	4.16250706	0.55519658
1	-2.52541018	2.79990673	0.18779658	1	0.25608981	2.84800673	0.03499658
1	-4.71191025	1.20160675	0.40939659	1	1.52698970	3.35470676	-2.05880356
1	-4.29000998	-0.47889319	2.45279646	1	1.45518970	1.60770679	-1.78560352
1	-1.85101008	0.03260682	3.47349644	1	2.93238974	2.50040674	-1.41030347
40	-1.92521024	-0.34659317	0.31619656	1	5.29738998	-2.95139313	0.27779657
1	-0.30151021	-0.59039319	1.35029650	1	4.41798973	-2.82249308	-1.25290346
1	-0.29471019	0.26200682	-0.60650343	1	3.78148985	-3.83679318	0.05069657
6	-2.94131017	0.80590683	-2.08550358	1	4.33668995	-1.92679322	2.39409661
6	-1.63331008	0.89820683	-2.41560340	1	2.76338983	-2.70019317	2.17099643
6	-0.90421021	-0.05309319	-3.31700349	1	2.88148975	-0.93629318	2.34149647
1	-3.56571031	0.00370681	-2.46910357	1	1.53698969	4.98090696	-0.09310342
1	-3.45351028	1.64150679	-1.62220347	1	2.95958972	4.16960716	0.57969660
1	-0.75211018	0.44230682	-4.28200340	1	1.50778985	4.36690712	1.56869650
1	0.08408982	-0.30869317	-2.92710352				

TS-1H

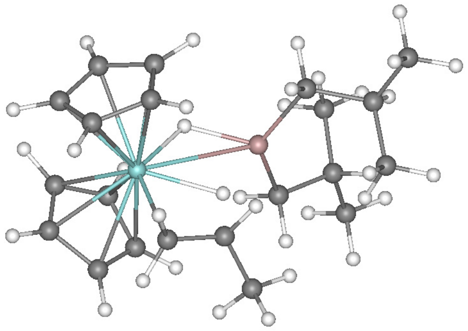

Zero-point vibrational energy	1353632.3 (Joules/Mol)
	323.52589 (Kcal/Mol)
Zero-point correction	0.515571 (Hartree/Particle)
Thermal correction to Energy	0.543544
Thermal correction to Enthalpy	0.544488
Thermal correction to Gibbs Free Energy	0.457231
Sum of electronic and zero-point Energies	-4604.050627
Sum of electronic and thermal Energies	-4604.022655
Sum of electronic and thermal Enthalpies	-4604.021711
Sum of electronic and thermal Free Energies	-4604.108967

cartesian

6	1.85980844	2.42909479	-0.42229149
6	2.10580850	2.40209484	0.98080844
6	3.44800854	2.01589489	1.18260849
6	4.04690838	1.80889487	-0.09469152
6	3.07110834	2.09699488	-1.07889152
1	3.23280859	2.09039474	-2.14829159
1	0.93100846	2.72409487	-0.89739150
1	1.39010847	2.64289474	1.75620854
1	3.93960857	1.91029489	2.14170861
1	5.07890844	1.53749490	-0.28179151
6	2.93800831	-1.12210512	2.32390857
6	4.12310839	-1.10220516	1.54270852
6	3.91900826	-1.92130518	0.40960848
6	2.60990858	-2.48030519	0.50550848
6	2.01740837	-2.00310516	1.69340849
1	1.02880847	-2.25440526	2.05740857
1	2.77740860	-0.60060507	3.25920844
1	5.02700853	-0.55150509	1.77110851
1	4.64650822	-2.11800504	-0.36779150
1	2.16030836	-3.17720509	-0.19249150
40	2.32710838	0.01739488	0.18270847
1	0.59670842	-1.64520514	-2.07489157
6	1.13770843	-0.70910507	-2.21849155
6	2.53780842	-0.76890510	-2.17279148
6	0.42150843	0.32749486	-3.05359149
1	0.92580843	1.29519486	-3.00759149
1	-0.62299156	0.45169488	-2.76139140
1	0.44500846	-0.01480512	-4.09269190
1	0.60140848	-0.21940511	-0.82599151
1	3.09980822	-0.03340512	-2.73809147
1	2.99940825	-1.74520516	-2.08349156
1	0.74140847	0.26479489	1.31520855
13	-0.74729156	0.05779488	0.49130848
6	-1.62359154	1.76719487	0.11920848
6	-1.48909152	-1.73240519	0.76380849
1	-1.54469144	2.01629496	-0.94999152
6	-3.10429144	1.80619490	0.55370849
1	-1.08359158	2.57119489	0.64480847
6	-2.86919165	-1.94430518	0.11010848
1	-0.78939152	-2.48340511	0.35960847
1	-1.55219162	-1.94800520	1.84110856
6	-3.72559166	3.17609477	0.26890847
6	-3.24279165	1.45539486	2.03810835
1	-3.65879154	1.05789483	-0.02849152
6	-3.36779165	-3.37710524	0.31500849
6	-2.81709146	-1.60360515	-1.38239145
1	-3.58879137	-1.26640511	0.58780849
1	-4.36159134	-3.51980519	-0.11909152
1	-3.42369127	-3.62620521	1.37810850
1	-2.68569136	-4.08970499	-0.16229150
1	-4.78189135	3.19979477	0.55210847
1	-3.65389156	3.43089485	-0.79199153
1	-3.20669174	3.95469475	0.83890843
1	-4.28509140	1.49629486	2.36570859
1	-2.67379141	2.16229486	2.65350842
1	-2.87799168	0.44269487	2.26160860
1	-3.77979136	-1.77270520	-1.87269151
1	-2.07349157	-2.22980523	-1.89279151
1	-2.55449152	-0.54920512	-1.55319142

I-2H_a

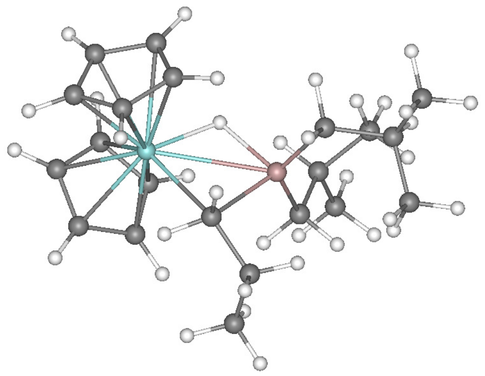

Zero-point vibrational energy	1365071.3 (Joules/Mol)
	326.25987 (Kcal/Mol)
Zero-point correction	0.519928 (Hartree/Particle)
Thermal correction to Energy	0.548206
Thermal correction to Enthalpy	0.549150
Thermal correction to Gibbs Free Energy	0.461586
Sum of electronic and zero-point Energies	-4604.069359
Sum of electronic and thermal Energies	-4604.041082
Sum of electronic and thermal Enthalpies	-4604.040138
Sum of electronic and thermal Free Energies	-4604.127702

cartesian

6	3.41094589	1.67184234	1.17716777	1	-0.20835423	1.85004234	-2.57713223
6	2.24434566	2.17704248	0.54206777	13	-0.61325419	-0.09695762	0.00306778
6	2.41054583	2.03294230	-0.85603219	6	-1.21695423	1.77244234	-0.03773221
6	3.69244576	1.45604241	-1.08963227	6	-1.59135425	-1.75725758	0.40286779
6	4.31324577	1.24714243	0.16516778	1	-2.12775421	1.86504233	-0.64923221
40	2.34764576	-0.29625762	0.08646779	6	-1.52395427	2.34054232	1.36746776
6	0.73014575	-0.75995761	-1.62403226	1	-0.47755420	2.42444229	-0.52353221
1	0.78224576	0.01374238	1.14416778	6	-3.13385439	-1.74215758	0.36676779
6	1.91674578	-2.67765760	0.78896779	1	-1.24845421	-2.54205751	-0.29313222
6	2.38544583	-1.98945761	1.94286776	1	-1.26645422	-2.10035753	1.39866769
6	3.72594595	-1.60065758	1.70546770	6	-2.57315445	1.49714243	2.09586763
6	4.08984566	-2.04225779	0.40256780	6	-1.97745430	3.80014229	1.27926779
6	2.97264576	-2.72195768	-0.15123221	1	-0.60535419	2.31014228	1.97226775
1	1.70654583	2.34254241	-1.61703229	6	-3.69625425	-3.12715769	0.70086777
1	4.13124561	1.24644244	-2.05903220	6	-3.65695429	-1.27325761	-0.99233216
1	1.38144577	2.60064244	1.04076779	1	-3.49595404	-1.04125762	1.12766778
1	3.59584570	1.65744233	2.24446774	1	-4.78995419	-3.11945772	0.72006780
1	5.30304575	0.83734238	0.32426780	1	-3.34425402	-3.47395754	1.67666769
1	4.36794567	-1.07225764	2.39906764	1	-3.37875414	-3.85875750	-0.05103222
1	1.81754577	-1.80705762	2.84646773	1	-2.83565426	1.93724227	3.06206775

1	0.92694581	-3.10145760	0.66506779	1	-2.21995401	0.47594237	2.28936768
1	2.93634558	-3.18665767	-1.13003230	1	-3.49015427	1.42794240	1.49886775
1	5.06094599	-1.92825758	-0.06343221	1	-2.15195417	4.22554255	2.27196765
1	1.73824573	-0.53795761	-2.07093215	1	-2.91195440	3.87134242	0.71246779
1	0.67284578	-1.85175765	-1.56103230	1	-1.23125422	4.41874218	0.77096778
6	-0.26475424	-0.32695761	-2.73543239	1	-4.74895430	-1.31585765	-1.03493226
6	-0.02775425	1.06444240	-3.31363225	1	-3.27145433	-1.91375756	-1.79573226
1	-1.30195427	-0.40175763	-2.37523222	1	-3.36565399	-0.23755762	-1.20843232
1	-0.70515418	1.24334240	-4.15133190	1	-0.20395422	-1.06785762	-3.53873229
1	0.99544579	1.16234243	-3.69223237				

I-2H_bi

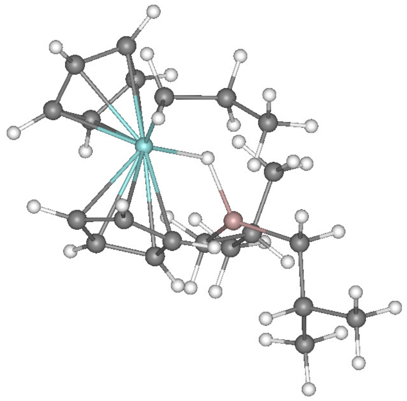

Zero-point vibrational energy	1362944.2 (Joules/Mol)
	325.75147 (Kcal/Mol)
Zero-point correction	0.519118 (Hartree/Particle)
Thermal correction to Energy	0.548034
Thermal correction to Enthalpy	0.548978
Thermal correction to Gibbs Free Energy	0.456332
Sum of electronic and zero-point Energies	-4604.057475
Sum of electronic and thermal Energies	-4604.028559
Sum of electronic and thermal Enthalpies	-4604.027615
Sum of electronic and thermal Free Energies	-4604.120260

cartesian

6	-4.18676949	-1.67005587	0.10166949	1	-1.81656957	3.39204407	1.10836947
6	-3.45776939	-2.18865609	-0.99433053	1	-2.64466953	0.97214413	2.83156967
6	-2.21226954	-2.67595601	-0.50843048	13	1.08293045	-0.29475588	0.02226950
6	-2.19206953	-2.48385596	0.89676952	6	1.78163040	1.41334414	0.65376949
6	-3.39606953	-1.84215581	1.27026951	6	1.90723050	-1.88895583	-0.73743051
1	-3.67626953	-1.55015588	2.27516937	1	2.47323036	1.21364415	1.48766947
1	-5.17426920	-1.22975588	0.05746950	6	2.53573036	2.26214409	-0.39493051
1	-3.79796934	-2.22945595	-2.02143049	1	0.96493053	2.00244403	1.09726954

1	-1.43806958	-3.15285587	-1.09693050	6	2.99843025	-2.40985608	0.22536948
1	-1.38786960	-2.76715589	1.56416953	1	1.15583050	-2.67985606	-0.88113052
6	-1.06056952	1.79844415	-1.42873049	1	2.34293032	-1.70035589	-1.72773051
6	-2.40056944	1.73654413	-1.86003053	6	3.73373079	1.49604416	-0.96373051
6	-2.58266973	0.51894414	-2.57323027	6	2.99203062	3.59234405	0.20996949
6	-1.34016955	-0.15915588	-2.61763048	1	1.85573041	2.49464393	-1.22793055
6	-0.39546952	0.62694412	-1.91023052	6	3.63043070	-3.70265603	-0.29423052
1	0.68463051	0.50944412	-2.01743054	6	2.41543055	-2.62265587	1.62636948
1	-0.59136951	2.61224413	-0.88673049	1	3.78943062	-1.65105581	0.29776949
1	-3.15776968	2.49004412	-1.69703054	1	4.42273045	-4.05455589	0.37266952
1	-3.50156975	0.19634412	-3.04723072	1	4.06333065	-3.55775595	-1.28753054
1	-1.13386953	-1.09265590	-3.12713051	1	2.87483025	-4.49255610	-0.36743051
40	-2.17176962	-0.20305587	-0.20443051	1	4.28863049	2.10384393	-1.68323052
1	-0.42496952	-0.59725589	0.66176951	1	3.43713045	0.57224417	-1.47713053
1	-1.46666956	0.93684411	1.56916952	1	4.42413044	1.21864414	-0.15893051
6	-3.56516933	1.22354412	0.87956953	1	3.50033045	4.21304417	-0.53353047
6	-2.43686962	1.45004416	1.87086952	1	3.68983078	3.41334391	1.03496945
6	-2.03776956	2.91644406	2.06826973	1	2.14473057	4.16084385	0.60506952
1	-4.44576931	0.77734417	1.33676946	1	3.16053057	-3.01485586	2.32336950
1	-3.85056973	2.14524412	0.37536949	1	1.58323038	-3.33605599	1.59156954
1	-1.16196954	3.01454401	2.71396971	1	2.03863049	-1.68705583	2.07116938
1	-2.86616945	3.46144414	2.52536964				

I-2H_bo

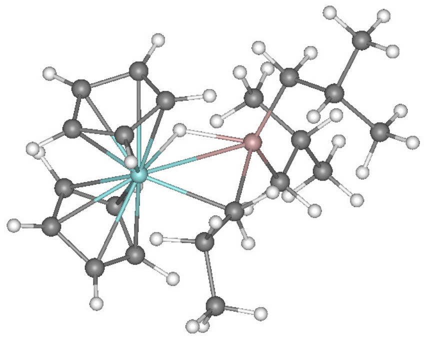

Zero-point vibrational energy	1367094.0 (Joules/Mol)
	326.74332 (Kcal/Mol)
Zero-point correction	0.520699 (Hartree/Particle)
Thermal correction to Energy	0.548879
Thermal correction to Enthalpy	0.549823
Thermal correction to Gibbs Free Energy	0.461991
Sum of electronic and zero-point Energies	-4604.080319
Sum of electronic and thermal Energies	-4604.052139
Sum of electronic and thermal Enthalpies	-4604.051195
Sum of electronic and thermal Free Energies	-4604.139027

cartesian

6	1.61622548	-2.28887296	1.10839832	1	4.81002569	-1.30877292	1.28319836
6	2.94332528	-2.31967306	0.60099828	1	2.79842544	-0.96687287	-3.98620200
6	3.74712539	-1.47647297	1.40399837	13	-0.91267461	0.13922711	0.21269830
6	2.92162538	-0.93847287	2.43189812	6	-1.82037473	-1.42397296	0.98109829
6	1.61412549	-1.45427299	2.25999832	6	-1.78547478	1.77192700	-0.41950169
1	0.76402539	-1.25097299	2.89809823	1	-2.71377468	-1.05357289	1.50879836
1	0.76802540	-2.84287286	0.72189832	6	-2.28897476	-2.47207284	-0.05280171
1	3.28632522	-2.89457297	-0.25090170	1	-1.22227454	-1.92127299	1.75719833
1	3.24562526	-0.28527290	3.23229814	6	-2.88587475	2.24932694	0.55049831
6	2.13332534	2.25042701	-0.88960171	1	-1.05387473	2.58582711	-0.55200171
6	3.42402530	1.67882705	-1.06530166	1	-2.22387457	1.60812700	-1.41480172
6	4.04372549	1.58942699	0.20489830	6	-3.19637465	-1.83307290	-1.10960174
6	3.14082527	2.11592698	1.16959834	6	-3.00937462	-3.63967299	0.62689829
6	1.96952534	2.53822708	0.49009830	1	-1.40707469	-2.88757300	-0.56720173
1	1.10372543	3.00322700	0.94419825	6	-3.56807446	3.52252698	0.04469829
1	1.41882539	2.47412705	-1.67250168	6	-2.31087470	2.47292709	1.95249832
1	3.86832523	1.38222706	-2.00880170	1	-3.64957452	1.46272707	0.62059832
1	3.33002543	2.22012711	2.23059821	1	-4.36967468	3.84202695	0.71739829
40	2.08302546	0.03272711	0.29049829	1	-3.99977469	3.37042713	-0.94830167
1	0.47622538	0.56562710	1.18199837	1	-2.84187460	4.34012747	-0.02620171
6	1.89132547	-0.54267287	-3.55040169	1	-3.55757451	-2.57207298	-1.83010173
6	0.43942538	-0.43237290	-1.44040167	1	-2.69157457	-1.04167295	-1.68050170
6	1.67982531	-1.03167295	-2.11510181	1	-4.07087421	-1.37807298	-0.63100171
1	1.64412546	-2.12227297	-2.07500172	1	-3.31587458	-4.39817286	-0.09960170
1	-0.34177464	-1.20387292	-1.51610172	1	-3.90777469	-3.28057289	1.13989830
1	0.10992539	0.42882711	-2.03080177	1	-2.36847472	-4.12017250	1.37199831
1	2.66712523	-0.80097288	-1.61020172	1	-3.07137465	2.83382702	2.65029812
1	1.96702528	0.54792714	-3.58100176	1	-1.50917459	3.22162700	1.92169833
1	1.04012537	-0.83687288	-4.16750145	1	-1.89387465	1.55092704	2.38289809
1	5.04282570	1.22052705	0.39959830				

I-4H_a

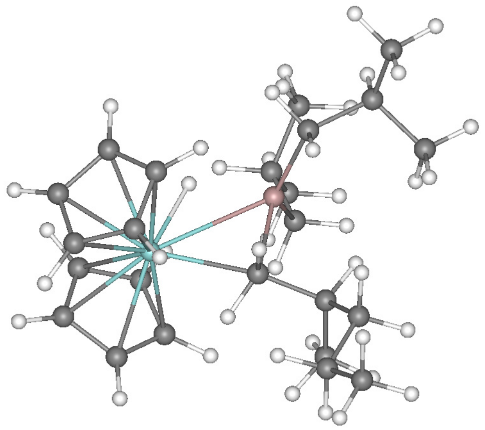

Zero-point vibrational energy	1592853.6 (Joules/Mol)
	380.70114 (Kcal/Mol)
Zero-point correction	0.606686 (Hartree/Particle)
Thermal correction to Energy	0.638819
Thermal correction to Enthalpy	0.639763
Thermal correction to Gibbs Free Energy	0.543141
Sum of electronic and zero-point Energies	-4721.872707
Sum of electronic and thermal Energies	-4721.840574
Sum of electronic and thermal Enthalpies	-4721.839629
Sum of electronic and thermal Free Energies	-4721.936252

cartesian

6	3.61376929	-2.25745869	0.43127060	6	0.20796928	4.01534128	1.35607064
6	2.44126940	-2.17995882	1.23077059	6	-0.20163071	5.46624088	1.09997058
6	2.50286913	-0.98265880	1.98187065	1	1.08776927	3.77654123	0.74397063
6	3.72586918	-0.32425880	1.66177058	1	0.51346928	3.90564132	2.40227056
6	4.41646957	-1.12145889	0.71777064	1	0.61056930	6.15614128	1.33807063
40	2.35176945	-0.28675881	-0.42382944	1	-1.06443071	5.74044085	1.71287060
6	0.53156924	1.10364115	0.26827061	1	-0.47643077	5.61724091	0.05217059
1	0.93486929	-1.48775887	-0.88472939	13	-0.58153069	-0.71945882	-0.24742940
6	1.84626925	0.64174116	-2.71442938	6	-1.04073071	-1.95775878	1.20877063
6	2.46776915	-0.62215883	-2.91432929	6	-1.62733078	-0.09505881	-1.79262936
6	3.79926920	-0.53415883	-2.44132948	1	-2.01413083	-1.68555880	1.64507055
6	4.00466919	0.78284115	-1.94242942	6	-1.12083077	-3.42985868	0.74427062
6	2.79836917	1.50904119	-2.12932944	1	-0.32563072	-1.88725889	2.04117060
1	1.76666927	-0.63805884	2.69587064	6	-3.15953064	-0.27505881	-1.78382945
1	4.08236933	0.60564119	2.09077072	1	-1.41953063	0.98034120	-1.93282938
1	1.64296925	-2.91085863	1.25827062	1	-1.22163069	-0.58075881	-2.69482946
1	3.86796927	-3.06525874	-0.24452940	6	-2.14173079	-3.60265875	-0.38342941
1	5.38656950	-0.90295881	0.28857058	6	-1.45313072	-4.35805893	1.91567063

1	4.53746939	-1.32625878	-2.46742940	1	-0.14173073	-3.73275876	0.34327060
1	2.00386930	-1.49165881	-3.36242938	6	-3.78083086	0.30634120	-3.05732942
1	0.82436925	0.89034116	-2.97632933	6	-3.79273081	0.36664119	-0.54702944
1	2.63596916	2.54654121	-1.85892940	1	-3.38643074	-1.34735882	-1.76082945
1	4.92976904	1.17834115	-1.54122937	1	-4.86193085	0.14094120	-3.08462930
1	1.47966933	1.43064117	0.77487063	1	-3.34713078	-0.14745881	-3.95312929
1	0.44276929	1.73084116	-0.62632936	1	-3.60513067	1.38694119	-3.10832930
6	-0.59413075	1.55434120	1.24697065	1	-2.24803066	-4.65385914	-0.66592938
6	-0.92933071	3.04324126	1.04047060	1	-1.85503078	-3.05035877	-1.28762937
6	-0.26783073	1.22814119	2.70607066	1	-3.12653065	-3.23905873	-0.06702940
1	-1.53223062	1.02454114	1.00517058	1	-1.46903062	-5.40655899	1.60347056
1	-1.01723075	1.67004120	3.36827064	1	-2.43903065	-4.11285877	2.32487059
1	0.70856929	1.63164115	2.99567056	1	-0.72213078	-4.25535917	2.72387052
1	-0.26933074	0.15104121	2.88877058	1	-4.88403082	0.30084121	-0.57892942
1	-1.79353070	3.28744125	1.67017066	1	-3.52923059	1.43074119	-0.48632944
1	-1.25543070	3.18964124	0.00207059	1	-3.46733093	-0.12125881	0.38017058

I-4H_bi

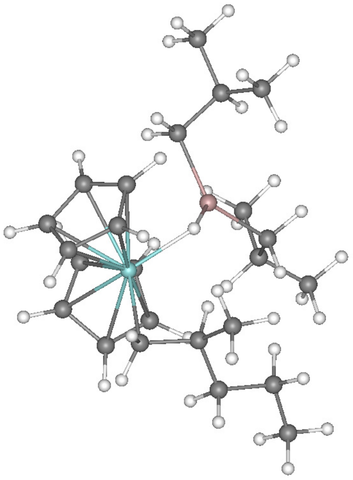

Zero-point vibrational energy	1589067.4 (Joules/Mol)
	379.79623 (Kcal/Mol)
Zero-point correction	0.605244 (Hartree/Particle)
Thermal correction to Energy	0.638056
Thermal correction to Enthalpy	0.639001
Thermal correction to Gibbs Free Energy	0.539447
Sum of electronic and zero-point Energies	-4721.861016
Sum of electronic and thermal Energies	-4721.828203
Sum of electronic and thermal Enthalpies	-4721.827259
Sum of electronic and thermal Free Energies	-4721.926813

cartesian

6	-2.80794263	-3.53673816	0.00375737	6	2.69075727	-1.76053822	-0.11804263
6	-1.80404258	-3.80743814	-0.95384264	1	2.48825741	1.76246190	1.13385737
6	-0.53574264	-3.65253830	-0.32464263	6	2.17145729	2.38806176	-0.90374261
6	-0.76614261	-3.31633830	1.03285742	1	0.81195736	2.09076190	0.77575737
6	-2.16324258	-3.21833825	1.23055732	6	4.00375748	-1.49593818	0.65215737
1	-2.65974259	-2.98253822	2.16345739	1	2.27005744	-2.71513820	0.23015738
1	-3.87604260	-3.57423830	-0.16514263	1	2.90615749	-1.89953816	-1.18794262
1	-1.97014260	-4.10433817	-1.98194265	6	3.46085739	1.79146171	-1.47524261
1	0.43045738	-3.81853819	-0.78544265	6	2.36295748	3.87666202	-0.60024261
1	-0.00464264	-3.15323830	1.78575742	1	1.39015734	2.31106186	-1.67314267
6	-1.04664266	0.69616175	-1.87054265	6	5.03145742	-2.59953809	0.39135736
6	-2.20554256	0.09736177	-2.39994264	6	3.72175741	-1.37113822	2.15225744
6	-1.85394263	-1.19023824	-2.89634252	1	4.42985725	-0.54593825	0.30235738
6	-0.46454263	-1.37323821	-2.70744252	1	5.97015762	-2.40013838	0.91625738
6	0.04115736	-0.21543823	-2.06294250	1	5.25305748	-2.68933821	-0.67554265
1	1.09935737	0.05136177	-2.03994274	1	4.64875746	-3.56483817	0.74025738
1	-0.97194266	1.68706179	-1.43314266	1	3.79255724	2.33676171	-2.36274266
1	-3.19084263	0.53946173	-2.44284272	1	3.35005736	0.73646176	-1.76464260
1	-2.52904272	-1.88643825	-3.37884259	1	4.26425743	1.84216189	-0.73154265
1	0.11805737	-2.23303819	-3.01464272	1	2.65385747	4.43086147	-1.49724269
40	-1.57074261	-1.38133824	-0.40934262	1	3.14725733	4.01376152	0.15145737
1	-0.05804265	-0.77073824	0.75035739	1	1.44245744	4.32086134	-0.20954263
1	-1.79684258	0.42306176	0.87195742	1	4.63525724	-1.18073821	2.72165728
6	-3.62334251	-0.60703826	0.17205736	1	3.27625728	-2.29633832	2.53615737
6	-2.93394256	0.46946177	0.99505734	1	3.03025746	-0.54613823	2.38135743
6	-3.27644253	1.89036179	0.51545739	1	-4.17824268	0.48536178	2.76135731
1	-4.18314266	-1.30573821	0.79465735	1	-2.50564265	0.98496175	3.08005738
1	-4.28234291	-0.20553824	-0.59714264	1	-2.88654256	-0.72003824	2.82745743
6	-2.38144255	2.99746180	1.07095742	6	-2.68644261	4.35326147	0.43435735
1	-4.32164240	2.08446169	0.78725737	1	-1.32854259	2.73716187	0.89235735
1	-3.24144268	1.92006183	-0.58034265	1	-2.50004268	3.07066178	2.15645742
6	-3.13104272	0.29636177	2.50715733	1	-2.04494262	5.13686132	0.84285736
13	1.43095744	-0.29513824	0.15645736	1	-3.72514272	4.64356136	0.61465734
6	1.71565747	1.62466168	0.36075735	1	-2.53314257	4.32306147	-0.64904261

I-4H_bo

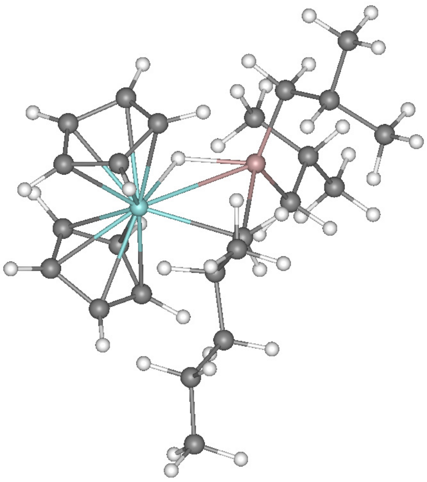

Zero-point vibrational energy	1591929.3 (Joules/Mol)
	380.48023 (Kcal/Mol)
Zero-point correction	0.606334 (Hartree/Particle)
Thermal correction to Energy	0.638491
Thermal correction to Enthalpy	0.639436
Thermal correction to Gibbs Free Energy	0.542098
Sum of electronic and zero-point Energies	-4721.885706
Sum of electronic and thermal Energies	-4721.853548
Sum of electronic and thermal Enthalpies	-4721.852604
Sum of electronic and thermal Free Energies	-4721.949942

cartesian

6	-0.62101316	1.18547797	2.63040280	1	-2.78311324	2.97777796	-0.20739710
6	-2.04071307	1.19587803	2.58180285	1	-1.37281322	3.16987777	-1.25949717
6	-2.51521325	0.00997794	3.19090295	1	-1.14741325	2.83397794	0.45960289
6	-1.38591325	-0.72762209	3.64660287	1	-2.41051316	-0.24542207	-2.30199718
6	-0.22101320	0.00397794	3.31410289	1	-1.95311320	1.34797800	-2.87159705
1	0.79608679	-0.28152207	3.54920292	1	-3.90641332	-2.39892220	1.79190290
1	0.04408678	1.96137798	2.26840305	1	-3.55461335	-0.26152205	3.32670283
1	-2.65911317	1.97687805	2.15540290	13	1.51678681	-0.28472206	0.12930287
1	-1.41411328	-1.66462207	4.18800259	6	2.53508687	1.04847801	1.15160286
6	-1.65671325	-2.41412210	-0.68199718	6	2.23518682	-1.42202199	-1.29199708
6	-2.94141316	-2.05152202	-0.18899715	1	3.58818674	0.72517794	1.14800286
6	-3.04081321	-2.50112224	1.14970291	6	2.48448682	2.48647785	0.59350288
6	-1.81891322	-3.14412212	1.48820293	1	2.24978685	1.05807793	2.21260285
6	-0.97281325	-3.10542226	0.35020289	6	3.62088680	-1.99492204	-0.92949712
1	0.01958680	-3.53232217	0.28020287	1	1.55178678	-2.26022220	-1.50679708
1	-1.27831316	-2.24102211	-1.68199706	1	2.30838680	-0.85692209	-2.23279715
1	-3.71961331	-1.53962207	-0.74439716	6	3.02858686	2.53707790	-0.83809710

1	-1.58881319	-3.62112212	2.43250299	6	3.25688672	3.46047783	1.48760283
40	-1.26751316	-0.73322207	1.14770293	1	1.43588674	2.82697797	0.56900287
1	0.57128680	-1.25842202	1.22630286	6	4.15998697	-2.90212226	-2.03809714
1	-4.05241299	2.32197785	-2.01899719	6	3.56058669	-2.75522208	0.39900288
6	-2.49341321	0.82417792	-2.07339716	1	4.32048702	-1.15692210	-0.80839717
6	-3.96661329	1.23327804	-2.08949709	1	5.15928698	-3.27762222	-1.79819703
6	-4.67581320	0.74977791	-3.35419703	1	4.21778679	-2.36842203	-2.99069715
1	-4.47281313	0.82537788	-1.20289707	1	3.49948668	-3.76562214	-2.17589712
1	-5.72201300	1.06107795	-3.36409712	1	2.99808669	3.55217791	-1.24379706
1	-4.64871311	-0.34162205	-3.42969704	1	2.46828675	1.89127791	-1.52779710
1	-4.19681311	1.15717793	-4.24849701	1	4.07088709	2.20027781	-0.85689712
6	-0.34651321	0.52757794	-0.73249710	1	3.19848680	4.48507786	1.10820293
6	-1.76511323	1.11597800	-0.74999714	1	4.31348705	3.17577791	1.53040290
6	-1.77371323	2.61097789	-0.40789711	1	2.86788678	3.45317793	2.51040292
1	0.33538678	1.39267802	-0.77279711	1	4.52658701	-3.19792223	0.65770292
1	-0.18891320	-0.04982206	-1.64929712	1	2.82868671	-3.57052207	0.33890289
1	-2.46761322	0.64797789	0.01860288	1	3.27208686	-2.10542202	1.23790288

TS-4H

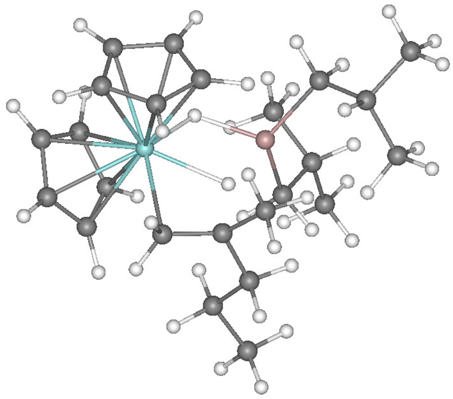

Zero-point vibrational energy	1580072.3 (Joules/Mol)
	377.64633 (Kcal/Mol)
Zero-point correction	0.601818 (Hartree/Particle)
Thermal correction to Energy	0.633588
Thermal correction to Enthalpy	0.634532
Thermal correction to Gibbs Free Energy	0.539886
Sum of electronic and zero-point Energies	-4721.854443
Sum of electronic and thermal Energies	-4721.822673
Sum of electronic and thermal Enthalpies	-4721.821729
Sum of electronic and thermal Free Energies	-4721.916374

cartesian

6	-2.07542920	-2.21796179	1.30722070		6	1.60037065	2.05113816	0.99902058
6	-2.81762934	-1.42776167	2.23362064		1	2.36197066	-1.46936166	-0.37327939
6	-4.05502939	-1.10296166	1.62892067		6	2.91507077	-1.43786168	1.72832060
6	-4.06182957	-1.63376176	0.31602061		1	1.22217059	-2.28486180	0.68162060
6	-2.84552932	-2.34816194	0.13482061		6	2.17897081	2.78053808	-0.23147939
1	-2.57122922	-2.91306186	-0.74457937		1	0.91337067	2.72013807	1.53372061
1	-1.09842932	-2.65116191	1.48612070		1	2.43277073	1.88093829	1.70352066
1	-2.50442934	-1.16226172	3.23572063		6	3.71427059	-2.74226189	1.68232071
1	-4.85772943	-0.53766179	2.08432055		6	2.24907064	-1.26556170	3.09742069
1	-4.87172937	-1.56036174	-0.39937943		1	3.61747074	-0.60586178	1.58302069
6	-3.33482933	1.89063823	1.79352069		6	3.09397078	1.85803831	-1.04127932
6	-3.97352934	1.93993831	0.52072060		6	2.93387079	4.04633808	0.18392061
6	-3.03122926	2.42493820	-0.41687942		1	1.34807062	3.09323812	-0.87997943
6	-1.80062938	2.63013816	0.25772062		1	3.54117060	2.37693810	-1.89407945
6	-2.00162935	2.31753826	1.63182068		1	2.55887079	0.98223823	-1.43547940
1	-1.26372933	2.40103817	2.41892076		1	3.91077089	1.48283827	-0.41317940
1	-3.79272914	1.59523833	2.72862077		1	4.49437094	-2.76016188	2.44912076
1	-5.01022911	1.70173824	0.31512061		1	4.19227076	-2.87656188	0.70812058
1	-3.23392916	2.62693810	-1.45987940		1	3.05277085	-3.59866190	1.85462070
1	-0.88292933	3.01663828	-0.17127939		1	2.97247076	-1.34496176	3.91342068
40	-2.25522923	0.13513824	0.36082059		1	1.48547077	-2.03746176	3.25222063
6	-0.52242929	1.47813833	-2.67097926		1	1.76057076	-0.28516176	3.20502067
6	-0.96062928	0.11593823	-2.16267943		1	3.29677081	4.59793806	-0.68847942
6	-2.35592937	-0.14036176	-2.06837940		1	3.79957056	3.78613806	0.80232060
6	0.01557070	-0.98186177	-2.57307935		1	2.29407072	4.71433830	0.76712060
6	-0.23582929	-2.39846182	-2.07377934		1	0.51067072	1.70453823	-2.39617944
1	1.03347063	-0.67226177	-2.30567932		1	-1.17172933	2.28733826	-2.33837938
1	-0.01782930	-0.98016173	-3.67177939		1	-0.57582927	1.43863833	-3.76447940
1	-0.47362930	0.23853824	-0.71687943		6	0.87167066	-3.35416174	-2.51987934
1	-2.68712926	-1.15826166	-2.24797940		1	-1.19542933	-2.76346183	-2.45577931
1	-3.02542925	0.62403822	-2.45237923		1	-0.29772928	-2.39566183	-0.97887939
1	-0.66822928	0.08883823	1.52492070		1	0.68007070	-4.36876202	-2.16517925
13	0.83107072	0.26843822	0.72542059		1	1.84277081	-3.03936195	-2.12787938
6	1.85857081	-1.39136171	0.60072058		1	0.94297069	-3.38716173	-3.61047935

I-5H

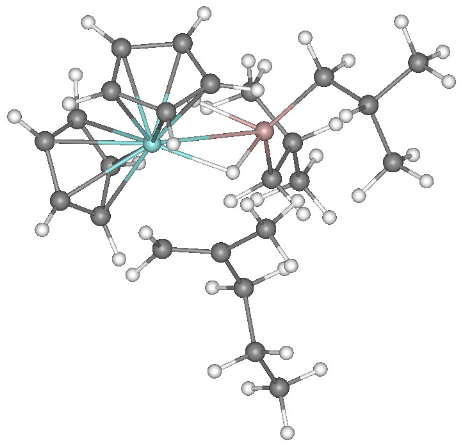

Zero-point vibrational energy	1579373.0 (Joules/Mol)
	377.47920 (Kcal/Mol)
Zero-point correction	0.601551 (Hartree/Particle)
Thermal correction to Energy	0.633967
Thermal correction to Enthalpy	0.634911
Thermal correction to Gibbs Free Energy	0.536965
Sum of electronic and zero-point Energies	-4721.867363
Sum of electronic and thermal Energies	-4721.834948
Sum of electronic and thermal Enthalpies	-4721.834003
Sum of electronic and thermal Free Energies	-4721.931949

cartesian

6	3.63226628	-0.11810585	1.98063087	1	0.33316630	2.15319419	1.63293087
6	3.91916633	-1.44790590	1.60383093	6	-1.07463360	4.49129438	1.99563098
6	2.75856638	-2.23060584	1.82583094	1	-0.17423373	4.73259401	0.05063094
6	1.76996636	-1.38910592	2.41013098	1	0.99206626	4.55089426	1.35273087
6	2.29656625	-0.08580586	2.49143100	1	-1.14113379	5.56279421	2.19323111
1	1.79216623	0.77639419	2.91053104	1	-0.88233370	3.98599434	2.94623113
1	4.32716608	0.71169418	1.94383097	1	-2.04813361	4.15629435	1.62723088
1	4.85746622	-1.80330586	1.19693089	13	-0.86703366	-1.03900588	-0.12396906
1	2.65196633	-3.29180574	1.63753092	6	-1.88453364	-0.78800583	1.53073096
1	0.78156626	-1.70230591	2.72083116	6	-1.63583374	-1.52480590	-1.86386907
6	3.08996630	-0.07580586	-2.25926900	1	-2.45203376	0.15179415	1.44833088
6	3.99066639	-0.94670582	-1.60356903	6	-2.86353374	-1.94930589	1.80293095
6	3.34176636	-2.20540595	-1.44956911	1	-1.23473382	-0.65150583	2.40853095
6	2.04396629	-2.10010576	-2.00216913	6	-2.06423354	-0.32230586	-2.73276901
6	1.87476635	-0.77160585	-2.48356891	1	-0.98093373	-2.18330574	-2.45046902
1	0.99256629	-0.38410586	-2.97926903	1	-2.53443360	-2.12780595	-1.65696907
1	3.30746627	0.94119412	-2.55606890	6	-3.66723347	-1.71810591	3.08483100

1	5.00836658	-0.71100587	-1.31736910	6	-2.11623359	-3.28410578	1.87523091
1	3.77496624	-3.09720588	-1.01466906	1	-3.57313347	-2.00490594	0.96633095
1	1.31156635	-2.89410567	-2.05926895	6	-2.90583372	0.67259419	-1.92826903
40	2.12746620	-0.60810584	0.02253094	6	-2.82573366	-0.78040582	-3.97956896
1	0.67006630	-1.88570595	0.10753094	1	-1.16093373	0.20559414	-3.07386899
1	0.33396631	0.20039414	-0.25916907	1	-3.26063371	1.49929416	-2.55086899
6	2.48286629	2.15429425	0.07193094	1	-2.33813357	1.11439407	-1.09606910
6	1.20196629	2.38839412	-0.32066908	1	-3.78353357	0.17669414	-1.49726903
6	0.81046629	2.55869412	-1.76016903	1	-4.39183378	-2.52020574	3.25373101
6	0.12666631	2.68089414	0.69563091	1	-4.21333361	-0.77170587	3.04213095
1	2.73716640	2.25789428	1.12293088	1	-2.99783373	-1.68110585	3.95173097
1	3.30656624	2.18709421	-0.63726908	1	-2.79203367	-4.11680603	2.08913112
1	-0.03973371	1.91579401	-2.01016903	1	-1.36283374	-3.25860572	2.67283106
1	0.47936630	3.59209418	-1.91156912	1	-1.60073376	-3.52270579	0.93373090
1	1.63116622	2.36699414	-2.45256901	1	-3.08813357	0.06469415	-4.62306929
6	0.02726626	4.19179440	0.98063093	1	-3.75373363	-1.28500593	-3.69016886
1	-0.84223372	2.32219434	0.32973093	1	-2.23153377	-1.48540592	-4.56766939

S3.7. $^{i}Bu_2AlCl$ reaction profile

I-0Cl

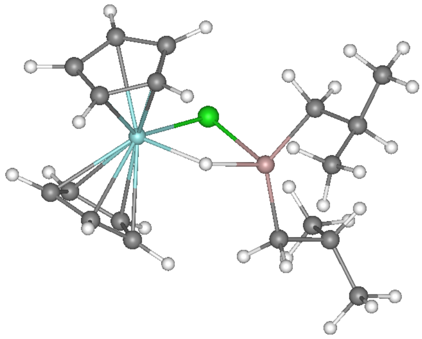

Zero-point vibrational energy	1119848.7 (Joules/Mol)
	267.65027 (Kcal/Mol)
Zero-point correction	0.426528 (Hartree/Particle)
Thermal correction to Energy	0.452154
Thermal correction to Enthalpy	0.453098
Thermal correction to Gibbs Free Energy	0.369347
Sum of electronic and zero-point Energies	-4945.900440
Sum of electronic and thermal Energies	-4945.874813
Sum of electronic and thermal Enthalpies	-4945.873869
Sum of electronic and thermal Free Energies	-4945.957621

cartesian

6	-3.43925595	1.94134998	1.52393806	6	1.38784409	1.80414999	0.36613801
6	-4.44755602	1.54955006	0.60443801	1	1.36404395	-1.83284998	-1.44116199
6	-3.95105600	1.78174996	-0.71056199	6	2.78894401	-1.91114998	0.20383801
6	-2.63965583	2.30804992	-0.59676194	1	0.71694404	-2.51594996	0.04453801
6	-2.31985593	2.39575005	0.78533804	6	2.84104395	1.89915001	-0.14706200
1	-1.38275599	2.74675012	1.20203805	1	0.78184408	2.54935002	-0.17306200
1	-3.50315595	1.88454998	2.60483813	1	1.34554410	2.09225011	1.42633808
1	-5.43445587	1.18355000	0.86033797	6	3.31814384	-3.30905008	-0.12516201
1	-4.49005604	1.60895002	-1.63406193	6	2.82764411	-1.66425002	1.71513808
1	-1.99345589	2.59555006	-1.41696191	1	3.45084381	-1.17935002	-0.27866200
6	-4.41365576	-1.49304998	-0.63116205	6	3.41994381	3.29434991	0.10243801
6	-3.39445591	-1.45525002	-1.61736190	6	2.91644406	1.54955006	-1.63596201
6	-2.25095582	-2.12325001	-1.09286201	1	3.45574379	1.17684996	0.40713799
6	-2.55595589	-2.54305005	0.22353801	1	4.45774412	3.35945010	-0.23706198
6	-3.89255595	-2.14785004	0.51343799	1	3.39474392	3.55005002	1.16523802
1	-4.42735577	-2.33964992	1.43713808	1	2.84034395	4.04885006	-0.44106200
1	-5.41505575	-1.09445000	-0.73596203	1	4.34494400	-3.43785000	0.22943801

1	-3.48355603	-1.03215003	-2.61086202	1	3.30624390	-3.49324989	-1.20296192
1	-1.31255591	-2.28244996	-1.60956192	1	2.69814396	-4.07375002	0.35603800
1	-1.88915586	-3.06734991	0.89713800	1	3.82514381	-1.84235001	2.12573791
40	-2.58945584	-0.04455002	0.26353800	1	2.12844396	-2.33084989	2.23373795
17	-0.79275590	-0.36265004	2.05763793	1	2.55884409	-0.62994999	1.97693801
1	-0.93835592	0.18874997	-0.71976197	1	3.94054413	1.61975002	-2.01246190
13	0.62074405	0.02064998	0.15833801	1	2.29934406	2.24055004	-2.22236204
6	1.35984397	-1.71194994	-0.34856197	1	2.56704402	0.52875000	-1.84296203

I-0Cl_bo

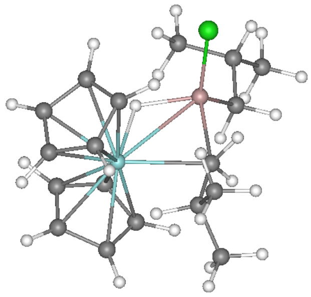

Zero-point vibrational energy	1123566.6 (Joules/Mol)
	268.53886 (Kcal/Mol)
Zero-point correction	0.427944 (Hartree/Particle)
Thermal correction to Energy	0.452808
Thermal correction to Enthalpy	0.453752
Thermal correction to Gibbs Free Energy	0.372702
Sum of electronic and zero-point Energies	-4945.899293
Sum of electronic and thermal Energies	-4945.874429
Sum of electronic and thermal Enthalpies	-4945.873485
Sum of electronic and thermal Free Energies	-4945.954535

cartesian

6	2.06536794	2.21805620	-0.67464805	1	-0.41083199	-0.32044393	-1.98104811
6	2.46676803	2.39315605	0.67135197	13	-1.39463198	-0.89634395	0.14105196
6	1.29386806	2.49715614	1.46885192	17	-1.63243198	-2.79774380	1.04805195
6	0.16946800	2.40605617	0.60885197	6	-2.89883208	0.06755607	-0.61674798
6	0.64246798	2.21025610	-0.71384811	6	-4.13183212	0.09225607	0.31075197
1	0.02376799	2.13365626	-1.59974802	1	-2.59863210	1.10415602	-0.84274805
1	2.73046803	2.12965608	-1.52624810	1	-3.17303205	-0.37704393	-1.58364809
1	3.48716784	2.46775627	1.02505195	6	-5.28433228	0.86905599	-0.32984802
1	1.26096809	2.66715622	2.53755188	6	-3.77643204	0.68935609	1.67505193
1	-0.86823207	2.47875619	0.91005194	1	-4.46293211	-0.94124389	0.46985197
6	3.28446794	-0.47154394	1.99325192	1	-6.17323208	0.85795605	0.30715197

6	2.17026806	-0.33644393	2.87005186	1	-5.55753231	0.44225606	-1.29874802
6	1.24626803	-1.36644399	2.56685185	1	-4.99803209	1.91455615	-0.49064800
6	1.76796806	-2.12214398	1.48245192	1	-4.64993191	0.74065602	2.33045197
6	3.04006791	-1.58114398	1.14925194	1	-3.39123201	1.71045601	1.55905199
1	3.71616793	-1.95704401	0.38985199	1	-3.01733208	0.09555608	2.20335197
1	4.18116808	0.13515607	2.00025201	6	2.18596792	-2.38904381	-2.01574802
1	2.06656790	0.39755610	3.65925193	1	2.39146781	-0.45594394	-1.17244804
1	0.30706799	-1.55824399	3.07065201	6	1.76946807	-0.18504393	-3.16684794
1	1.29356802	-2.99474382	1.05025196	1	3.24896789	-2.37124395	-2.26994801
40	1.32876801	0.16855606	0.57785201	1	1.65386808	-2.88494396	-2.83224797
6	0.19206800	-0.95134389	-1.31774807	1	2.04966807	-2.99224377	-1.11594808
1	-0.46293199	0.01225607	1.26645195	1	2.81416798	-0.13234393	-3.48494816
1	-0.18183200	-1.98244393	-1.42394805	1	1.37816799	0.83165610	-3.08294797
6	1.63546801	-0.96724391	-1.85404801	1	1.20226800	-0.69824398	-3.94814801

I-1Cl

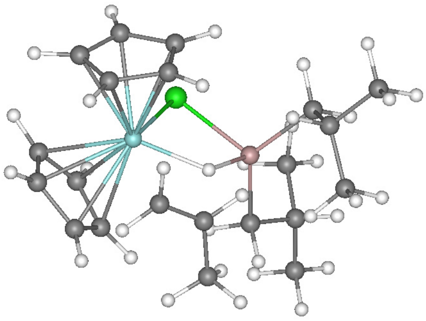

Zero-point vibrational energy	1340335.2 (Joules/Mol)
	320.34781 (Kcal/Mol)
Zero-point correction	0.510507 (Hartree/Particle)
Thermal correction to Energy	0.540765
Thermal correction to Enthalpy	0.541709
Thermal correction to Gibbs Free Energy	0.448968
Sum of electronic and zero-point Energies	-5063.676833
Sum of electronic and thermal Energies	-5063.646575
Sum of electronic and thermal Enthalpies	-5063.645631
Sum of electronic and thermal Free Energies	-5063.738372

cartesian

6	-2.73219657	-2.01865935	-1.46533215	1	-1.10809660	-0.69795930	-3.51973224
6	-3.74439669	-2.04245949	-0.48023215	1	-0.66049665	1.80604064	-1.72613215
6	-3.18379664	-2.61755943	0.69996786	13	1.22750330	-0.17745931	0.66956782
6	-1.83629656	-2.94415951	0.42536786	6	1.79800344	1.61084068	1.23236787
6	-1.53609669	-2.53595924	-0.89953220	6	2.22050333	-1.57875931	-0.26693216

1	-0.58589667	-2.66125917	-1.40543222	1	2.89430332	1.61984074	1.33066785
1	-2.85359669	-1.68935931	-2.48713207	6	1.39010334	2.71534061	0.23856786
1	-4.77229643	-1.72825933	-0.61433220	1	1.41590333	1.84304059	2.23586798
1	-3.70619655	-2.81585932	1.62756777	6	3.66650343	-1.68085933	0.26856786
1	-1.14119673	-3.39615917	1.11946785	1	1.72550344	-2.55285931	-0.12813213
6	-2.75599670	1.95464075	0.93006790	1	2.24800348	-1.40145934	-1.35163212
6	-3.94619656	1.19024074	0.73406786	6	1.96170330	2.42484045	-1.15343213
6	-4.03999662	0.26114067	1.80006778	6	1.83020329	4.10204077	0.71476781
6	-2.89049673	0.39944071	2.60766792	1	0.28960335	2.72354078	0.16276786
6	-2.10089660	1.46164072	2.07206798	6	4.43770361	-2.80565929	-0.42583212
1	-1.15599668	1.81344068	2.46956778	6	3.66990328	-1.88665926	1.78646779
1	-2.40369678	2.77104092	0.31066787	1	4.18130350	-0.73465931	0.05256785
1	-4.68929672	1.33984065	-0.03953215	1	5.47390318	-2.85245943	-0.07783213
1	-4.84549665	-0.44535932	1.95516777	1	4.45020342	-2.66515923	-1.51033223
1	-2.65279651	-0.18155931	3.48956776	1	3.96830320	-3.77325916	-0.21613213
40	-2.06379676	-0.41785932	0.38426787	1	1.65980339	3.17974091	-1.88713217
17	-0.15869668	-1.09195924	2.24346781	1	1.64800334	1.44234073	-1.53653216
1	-0.26839668	0.10984069	-0.21573213	1	3.05680346	2.41554070	-1.11833215
6	-2.58569670	1.07814074	-2.12173223	1	1.51470327	4.88674068	0.01986787
6	-1.23909664	1.01884067	-2.20903206	1	2.92130327	4.14334059	0.79696786
6	-0.46689668	0.05614069	-3.05903220	1	1.41350341	4.33004045	1.69986784
1	-3.22379661	0.40524065	-2.68743229	1	4.68510342	-2.00895929	2.17356777
1	-3.07199669	1.93454063	-1.67033219	1	3.09860349	-2.78305912	2.05466795
1	0.01640332	0.62194067	-3.86283207	1	3.22690344	-1.03615928	2.32446790
1	0.32950330	-0.43755931	-2.49483228				

TS-1Cl

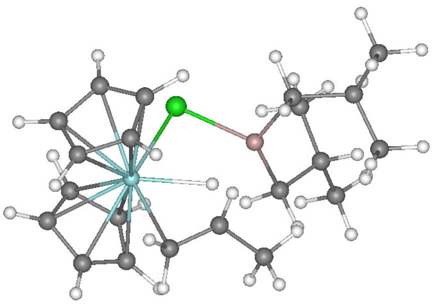

Zero-point vibrational energy	1336885.7 (Joules/Mol)
	319.52335 (Kcal/Mol)
Zero-point correction	0.509193 (Hartree/Particle)
Thermal correction to Energy	0.538692
Thermal correction to Enthalpy	0.539636
Thermal correction to Gibbs Free Energy	0.448350
Sum of electronic and zero-point Energies	-5063.668796

Sum of electronic and thermal Energies -5063.639296

Sum of electronic and thermal Enthalpies -5063.638352

Sum of electronic and thermal Free Energies -5063.729638

cartesian

6	2.57411003	2.25316954	-1.02201188	1	0.16791016	0.80236953	-2.84051180 1
6	3.88921022	1.69696951	-1.06941187	0.79261017	-1.95683050	-1.58831191	
6	4.39601040	1.67216945	0.24698816	13	-0.93638980	0.10036954	0.49388814
6	3.39141011	2.17396951	1.11988819	6	-1.64888978	-1.70873046	0.71478814
6	2.28551006	2.56876969	0.32008815	6	-1.70918989	1.73626947	-0.24771182
1	1.36301017	2.99736953	0.68968815	1	-1.47458982	-2.00373030	1.76008809
1	1.92411017	2.43446970	-1.86991191	6	-3.14728975	-1.86543036	0.37508816
1	4.42621040	1.39106965	-1.95771194	1	-1.08018982	-2.43363047	0.10938817
1	5.38200998	1.33206964	0.53558815	6	-3.08898973	2.00916958	0.39968818
1	3.47081017	2.28566957	2.19448829	1	-1.04628980	2.58576965	-0.02301185
6	3.02271008	-2.44453049	0.25268817	1	-1.81578994	1.71276951	-1.33931184
6	4.24400997	-1.73983049	0.41288817	6	-3.64208984	-3.26833034	0.73818815
6	4.20261002	-1.09173036	1.67918813	6	-3.41189003	-1.57713056	-1.10461187
6	2.94921017	-1.35473037	2.26708817	1	-3.72248983	-1.14303041	0.97038817
6	2.20851016	-2.18573046	1.37748814	6	-3.63598967	3.37316966	-0.02811183
1	1.21051025	-2.57213044	1.55138814	6	-3.01208973	1.91936946	1.92738807
1	2.75791025	-3.07263041	-0.58881181	1	-3.79158974	1.24056959	0.05138816
1	5.08201027	-1.74353051	-0.27411181	1	-4.63348961	3.54736948	0.38568819
1	4.99711037	-0.50543046	2.12248826	1	-3.70208979	3.44866967	-1.11701190
1	2.60711002	-0.99203044	3.22728801	1	-2.97898984	4.17446947	0.32748818
40	2.49221015	0.04026954	0.21528816	1	-4.71038961	-3.37973046	0.53148818
17	0.53211015	0.61926955	2.10658813	1	-3.47789001	-3.48363042	1.79758811
1	0.64241016	-0.30903047	-0.54491180	1	-3.10508990	-4.02353048	0.15328816
6	2.41111016	-0.68773049	-2.16611195	1	-4.46598959	-1.72333050	-1.35631192
6	1.05501020	-0.96263045	-1.95191193	1	-2.82508993	-2.25553036	-1.73701191
6	-0.02158982	-0.26923046	-2.75101185	1	-3.15468979	-0.54593045	-1.37891185
1	2.64311028	0.13076954	-2.84041190	1	-3.97238970	2.16026950	2.39148808
1	3.12441015	-1.50443053	-2.15891194	1	-2.26428986	2.61706948	2.32218814
1	-0.00718981	-0.70313048	-3.75611186	1	-2.74378991	0.91036952	2.27718806
1	-1.01828980	-0.42703047	-2.33361197				

I-2Cl_a

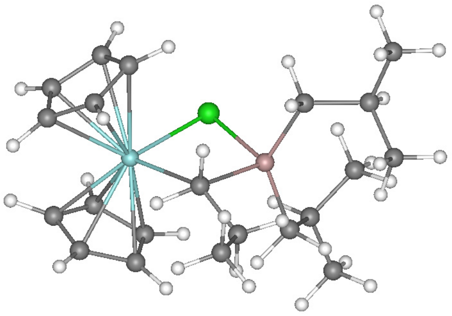

Zero-point vibrational energy	1349531.2 (Joules/Mol)
	322.54569 (Kcal/Mol)
Zero-point correction	0.514009 (Hartree/Particle)
Thermal correction to Energy	0.543849
Thermal correction to Enthalpy	0.544793
Thermal correction to Gibbs Free Energy	0.453673
Sum of electronic and zero-point Energies	-5063.695489
Sum of electronic and thermal Energies	-5063.665649
Sum of electronic and thermal Enthalpies	-5063.664705
Sum of electronic and thermal Free Energies	-5063.755825

cartesian

6	3.35046101	2.24961877	0.77360332	1	1.20366096	-1.27238142	-3.86959672
6	2.14476109	2.43821883	0.04100335	1	-0.11673898	0.46501866	-2.58759665
6	2.31876111	1.87341869	-1.23949671	13	-0.75833899	-0.04178134	0.25740334
6	3.63526106	1.32781863	-1.30799663	6	-1.31753898	1.74061859	-0.35539666
6	4.27726126	1.59071863	-0.07189667	6	-1.71873903	-1.68058133	0.76020336
40	2.43686104	-0.04068134	0.39200336	1	-2.16883898	1.61121869	-1.04199672
6	0.73846102	-0.90958136	-1.11199665	6	-1.75713897	2.70281887	0.76700330
17	0.62086105	0.47061867	2.12980342	1	-0.54403895	2.22621870	-0.96949673
6	2.43616104	-2.36538124	1.35400331	6	-3.25883889	-1.62028134	0.82500339
6	3.11436105	-1.54468131	2.29340339	1	-1.44633889	-2.48168135	0.05320334
6	4.30016088	-1.06698132	1.68890333	1	-1.32873893	-2.01058125	1.73470330
6	4.36846113	-1.60618138	0.37060332	6	-2.81183910	2.04851866	1.66400325
6	3.22596097	-2.42228127	0.17820334	6	-2.28513908	4.01951885	0.19070333
1	1.58426106	1.85691857	-2.03539658	1	-0.88723892	2.93561864	1.39750326
1	4.08456087	0.84801865	-2.16989660	6	-3.83393908	-2.95688128	1.30240333
1	1.24266100	2.90651870	0.41730332	6	-3.85213900	-1.24828136	-0.53599668
1	3.53386092	2.58051872	1.78960335	1	-3.55453920	-0.85098135	1.54890335
1	5.29676104	1.32891858	0.18170333	1	-4.92453909	-2.91578126	1.37800336
1	5.03776121	-0.42618135	2.15600348	1	-3.43713903	-3.23178124	2.28380322
1	2.76616096	-1.30508137	3.29030323	1	-3.57573891	-3.75578117	0.59790337

1	1.48696113	-2.86318135	1.51670337	1	-3.18133903	2.74371886	2.42300320
1	2.99026108	-2.97778130	-0.72169662	1	-2.41393900	1.17341864	2.19570327
1	5.16886091	-1.45348132	-0.34289667	1	-3.67083883	1.71771860	1.06730330
1	1.76166105	-0.81248134	-1.56789672	1	-2.54973912	4.72451830	0.98440337
1	0.64756107	-1.98278129	-0.90809667	1	-3.18213892	3.83811879	-0.41119668
6	-0.17413896	-0.59608138	-2.32789660	1	-1.54143906	4.49771833	-0.45439664
6	0.17576104	-1.45838141	-3.54249668	1	-4.94533920	-1.23598135	-0.50519669
1	-1.22643900	-0.78588134	-2.07019663	1	-3.55253887	-1.98078144	-1.29639673
1	-0.48873895	-1.23678136	-4.38029623	1	-3.52543879	-0.25708133	-0.87239671
1	0.08106107	-2.52158117	-3.30649662				

I-2Cl_bi

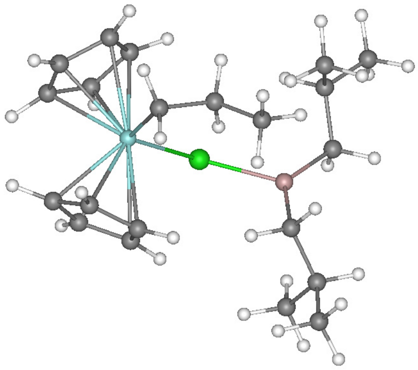

Zero-point vibrational energy	1346417.7 (Joules/Mol)
	321.80156 (Kcal/Mol)
Zero-point correction	0.512823 (Hartree/Particle)
Thermal correction to Energy	0.543408
Thermal correction to Enthalpy	0.544352
Thermal correction to Gibbs Free Energy	0.447967
Sum of electronic and zero-point Energies	-5063.686234
Sum of electronic and thermal Energies	-5063.655649
Sum of electronic and thermal Enthalpies	-5063.654705
Sum of electronic and thermal Free Energies	-5063.751090

cartesian

6	3.30522704	-2.38622546	-0.42100850	1	0.51762718	0.02107456	-4.06690836
6	4.38762712	-1.64132535	0.11169152	1	0.10972717	1.27077460	-2.88340855
6	4.13512707	-1.43922544	1.49589145	13	-1.95447278	-0.04112544	0.65519154
6	2.88222718	-2.01192546	1.80409145	6	-3.31437278	-1.37552536	1.00149155
6	2.36642718	-2.60022545	0.61239153	6	-1.83767295	1.62387455	-0.34270850
1	1.42582726	-3.13142538	0.52459157	1	-4.22407293	-1.10472536	0.44689155
1	3.21162724	-2.72172546	-1.44530845	6	-2.81967282	-2.76172543	0.52909154
1	5.26572704	-1.31682539	-0.43350849	1	-3.59757280	-1.41172540	2.06019163

1	4.78782701	-0.93272543	2.19539165	6	-2.83727288	2.64107466	0.25809154
1	2.40372729	-2.01532555	2.77579141	1	-0.82017285	2.03837466	-0.28220847
6	2.09542727	2.39847469	-0.02970847	1	-2.04987288	1.47427464	-1.40880847
6	3.48022723	2.12857461	-0.20720848	6	-2.25087285	-2.67572546	-0.89210856
6	3.98942709	1.64867461	1.02009153	6	-3.94197273	-3.80012536	0.59449148
6	2.92142701	1.62017465	1.96529150	1	-2.01377296	-3.08642530	1.19929147
6	1.76432729	2.11197472	1.31639147	6	-2.70617294	4.00647449	-0.41940847
1	0.78702718	2.21857452	1.77059150	6	-2.63407278	2.76907468	1.77109146
1	1.42412710	2.78857470	-0.78630853	1	-3.85487270	2.26987457	0.08179152
1	4.04752731	2.27387452	-1.11660850	1	-3.43557310	4.71857452	-0.02350849
1	5.01972723	1.37807465	1.21229148	1	-2.86107278	3.92707467	-1.49860847
1	2.98972702	1.31557465	3.00249147	1	-1.70557284	4.42027473	-0.24960849
40	2.40142727	-0.10842544	0.25269154	1	-1.93617296	-3.65332532	-1.26840854
17	-0.03487283	-0.61232543	1.62549150	1	-1.35787272	-2.02622557	-0.94250846
1	0.51012719	-0.32752544	-1.04740846	1	-2.99597287	-2.27372551	-1.58870852
6	2.47012711	-0.02462544	-2.01100850	1	-3.58407307	-4.79112530	0.30039153
6	1.02682722	-0.51002544	-2.03690839	1	-4.75937271	-3.52152538	-0.07890847
6	0.12772718	0.19177458	-3.06110859	1	-4.34967279	-3.87252545	1.60579145
1	0.97252715	-1.59622538	-2.15890861	1	-3.32417297	3.49077463	2.21559167
1	3.14532709	-0.68692547	-2.55030847	1	-1.61447287	3.10187459	1.99869144
1	2.54652715	0.97867459	-2.43190861	1	-2.80367279	1.81717455	2.30149150
1	-0.89887279	-0.18502544	-3.02170849				

I-2Cl_bo

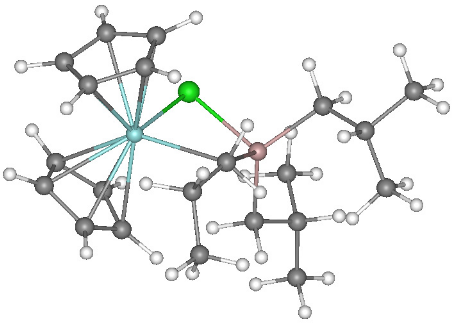

Zero-point vibrational energy	1352452.8 (Joules/Mol)
	323.24398 (Kcal/Mol)
Zero-point correction	0.515122 (Hartree/Particle)
Thermal correction to Energy	0.544525
Thermal correction to Enthalpy	0.545469
Thermal correction to Gibbs Free Energy	0.456382
Sum of electronic and zero-point Energies	-5063.694252
Sum of electronic and thermal Energies	-5063.664850
Sum of electronic and thermal Enthalpies	-5063.663906

Sum of electronic and thermal Free Energies -5063.752993

cartesian

6	-3.17361522	2.33361530	0.42350847	1	-5.26801491	-0.01598477	1.55290842
6	-4.29541540	1.48011518	0.20600849	1	-1.81921506	0.27441522	-3.98609138
6	-4.48141527	0.70811522	1.38380849	13	0.93698484	-0.00478477	0.60560846
6	-3.45001507	1.03361523	2.29050851	6	1.95848489	1.58131528	1.11830842
6	-2.64431524	2.04931521	1.69560850	6	1.59038496	-1.70478475	-0.11519152
1	-1.76971507	2.50421524	2.14450860	1	2.75758481	1.25531518	1.80290842
1	-2.78781509	3.06431532	-0.27669153	6	2.60808492	2.38681531	-0.02639151
1	-4.93441534	1.47551525	-0.66909146	1	1.32208490	2.24421525	1.72340846
1	-3.30031514	0.60161525	3.27240849	6	2.96218491	-2.08068466	0.48990849
6	-1.74521518	-2.20498466	-0.87349153	1	0.89878482	-2.53488469	0.09340848
6	-2.98171520	-1.76318479	-1.42659152	1	1.67818499	-1.65298474	-1.21199155
6	-3.96151519	-1.80648482	-0.41019151	6	3.52668476	1.50441527	-0.87629151
6	-3.33811522	-2.28408480	0.77890849	6	3.38748503	3.58691525	0.51960850
6	-1.98181510	-2.55548477	0.47730848	1	1.81718493	2.78011513	-0.68149149
1	-1.24481511	-2.93318486	1.17410851	6	3.50548506	-3.36868477	-0.13299152
1	-0.80741519	-2.32188487	-1.40129149	6	2.85758495	-2.22428489	2.01100850
1	-3.14931512	-1.46648479	-2.45549154	1	3.67498493	-1.27428472	0.27260849
1	-3.82221508	-2.45148468	1.73360848	1	4.49028492	-3.62178469	0.27050850
40	-2.33271527	-0.05058476	0.31790850	1	3.59878492	-3.27418470	-1.21869159
17	-0.58261514	-0.40178478	2.28170848	1	2.83098483	-4.20658493	0.07660848
6	-1.03671515	0.31511524	-3.22489142	1	4.00218487	2.07671523	-1.67799151
6	-0.48901516	1.03431523	-0.81779158	1	2.99058485	0.66721523	-1.34489155
6	-1.49971509	1.07491529	-1.98259151	1	4.32258511	1.07671523	-0.25609151
1	-1.72741508	2.11321521	-2.23799133	1	3.81898499	4.18501520	-0.28879151
1	-0.37501517	2.03651524	-0.39369151	1	4.20698500	3.24571514	1.16100848
1	0.46718484	0.81821525	-1.32159150	1	2.74398494	4.23771524	1.11860847
1	-2.52991509	0.68631524	-1.74419153	1	3.81318474	-2.52228475	2.45100856
1	-0.73261517	-0.70578480	-2.98159146	1	2.11608481	-2.98838472	2.27390862
1	-0.17241517	0.82281524	-3.65989137	1	2.55658484	-1.28808475	2.49980855
1	-5.00601530	-1.54638481	-0.52709150				

I-4Cl_a

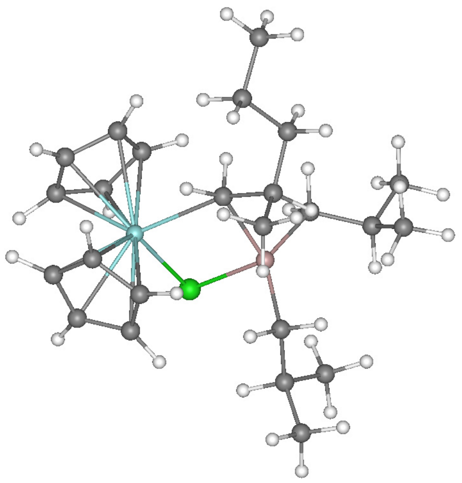

Zero-point vibrational energy	1575613.1 (Joules/Mol)
	376.58058 (Kcal/Mol)
Zero-point correction	0.600119 (Hartree/Particle)
Thermal correction to Energy	0.633129
Thermal correction to Enthalpy	0.634073
Thermal correction to Gibbs Free Energy	0.535962
Sum of electronic and zero-point Energies	-5181.495563
Sum of electronic and thermal Energies	-5181.462553
Sum of electronic and thermal Enthalpies	-5181.461609
Sum of electronic and thermal Free Energies	-5181.559720

cartesian

6	3.32843232	-2.84297204	0.51105881	6	0.96963227	3.73372769	1.25485873
6	2.10603237	-2.62567210	1.20745873	6	0.91613233	5.23652840	0.97635877
6	2.22683239	-1.42707205	1.93865883	1	1.78653240	3.28922796	0.66965878
6	3.52733231	-0.89037204	1.69945884	1	1.21513224	3.56842804	2.30955887
6	4.21413231	-1.78757203	0.84425879	1	1.86133218	5.72282839	1.22675884
40	2.36283231	-0.77877206	-0.49574119	1	0.12863231	5.71252823	1.56675875
6	0.62873226	0.82742798	0.12955879	1	0.70393229	5.43182802	-0.07864121
17	0.56383228	-2.22937202	-1.59724116	13	-0.83526772	-0.69117200	-0.39614120
6	2.40373230	0.56382799	-2.62994099	6	-1.41436768	-1.80257213	1.11955881
6	3.02293229	-0.67597204	-2.93104100	6	-1.78886771	0.26942798	-1.82454121
6	4.21143246	-0.77387202	-2.16984105	1	-2.24476767	-1.29207206	1.63335884
6	4.34313250	0.42372796	-1.40724123	6	-1.89196777	-3.21907210	0.73405880
6	3.23273230	1.25072789	-1.70634127	1	-0.62896770	-1.89277208	1.88335884
1	1.46993232	-0.99077201	2.57615900	6	-3.33126783	0.25802794	-1.81534123
1	3.93643212	0.01352796	2.13665891	1	-1.46566772	1.32412791	-1.80684125
1	1.22903228	-3.26017213	1.15945876	1	-1.43306780	-0.12397204	-2.78824115
1	3.55153227	-3.68607211	-0.13264120	6	-2.95606780	-3.16267204	-0.36424118

1	5.23453236	-1.68197203	0.49875879	6	-2.42096782	-3.97227216	1.95745885
1	4.90993214	-1.60157204	-2.18574119	1	-1.03596783	-3.78207207	0.33615881
1	2.63393211	-1.42827213	-3.60524106	6	-3.88536787	1.03432798	-3.01344109
1	1.46923232	0.92192793	-3.04624104	6	-3.88226795	0.83872795	-0.51074117
1	3.04603219	2.23302794	-1.28764117	1	-3.67806768	-0.77847207	-1.89924121
1	5.16383219	0.67612791	-0.74724120	1	-4.97866774	1.00072789	-3.03864121
1	1.61313224	0.99322796	0.64585876	1	-3.51276779	0.62532794	-3.95684099
1	0.66453230	1.47052789	-0.75614125	1	-3.58256769	2.08632803	-2.95924115
6	-0.37126774	1.52012789	1.11965883	1	-3.35166788	-4.15687180	-0.59054118
6	-0.35336769	3.04662800	0.91395879	1	-2.55606771	-2.75367212	-1.30154121
6	-0.12716770	1.13942790	2.58115888	1	-3.79686785	-2.53107214	-0.05174120
1	-1.40356779	1.21792793	0.88025880	1	-2.71086788	-4.99547195	1.70085883
1	-0.75596768	1.75032783	3.23485899	1	-3.30196786	-3.46547198	2.36615896
1	0.91463232	1.31372797	2.87325883	1	-1.66766787	-4.02257204	2.75005889
1	-0.37796772	0.09472796	2.77725887	1	-4.97526789	0.87732792	-0.52084118
1	-1.14796782	3.48202801	1.53225875	1	-3.51786757	1.86392784	-0.36424118
1	-0.62096769	3.26682782	-0.12784120	1	-3.58676767	0.24352798	0.36215878

I-4Cl_bi

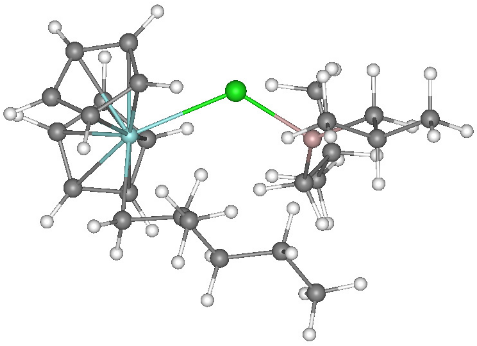

Zero-point vibrational energy	1571825.7 (Joules/Mol)
	375.67536 (Kcal/Mol)
Zero-point correction	0.598677 (Hartree/Particle)
Thermal correction to Energy	0.633010
Thermal correction to Enthalpy	0.633954
Thermal correction to Gibbs Free Energy	0.530101
Sum of electronic and zero-point Energies	-5181.490555
Sum of electronic and thermal Energies	-5181.456222
Sum of electronic and thermal Enthalpies	-5181.455278
Sum of electronic and thermal Free Energies	-5181.559131

cartesian

6	-3.87484121	1.96721029	0.72251761	6	1.99545884	-2.08998966	-0.41958237

6	-4.74384117	0.88931030	1.01711762	1	4.14845896	0.31941026	1.32691765
6	-4.22964144	0.21281029	2.15951777	6	3.10615897	2.19811034	0.99951762
6	-3.02954102	0.84621024	2.53921771	1	2.99005890	0.72491026	2.57971764
6	-2.79644108	1.92691040	1.63951766	6	2.71475887	-3.22388959	0.35441762
1	-1.95894122	2.61481023	1.68011761	1	0.99335879	-2.43038964	-0.71858239
1	-4.02764130	2.69491029	-0.06118236	1	2.53715897	-1.88198972	-1.34948242
1	-5.66034126	0.65291029	0.49081761	6	4.23205900	3.00521040	1.65301764
1	-4.68064117	-0.63998973	2.65081763	6	1.75425887	2.85901022	1.28571761
1	-2.39054108	0.55781025	3.36471772	1	3.27395892	2.21251035	-0.08608235
6	-2.20364118	-2.24978971	-1.16318238	6	2.59215879	-4.55538988	-0.38878238
6	-3.59874105	-1.97068965	-1.20618236	6	2.17955899	-3.35318971	1.78471756
6	-4.11924124	-2.16398978	0.09321763	1	3.77905893	-2.96628976	0.41771764
6	-3.04744101	-2.55168962	0.94911760	1	3.14835882	-5.34818983	0.11951763
6	-1.87724113	-2.63348961	0.15701763	1	2.97415900	-4.47299004	-1.40968239
1	-0.89634115	-2.91538978	0.51761764	1	1.54175901	-4.86379004	-0.44478238
1	-1.52394116	-2.21398973	-2.00598216	1	4.24365902	4.03591013	1.28681767
1	-4.16454124	-1.67508972	-2.07968235	1	5.20925903	2.56071043	1.44651759
1	-5.15634108	-2.05308962	0.38301763	1	4.09745884	3.03381038	2.73941779
1	-3.11984110	-2.79178977	2.00251770	1	1.75685883	3.90721011	0.97141761
40	-2.60894108	-0.18618971	0.28241763	1	1.53305888	2.83091044	2.35901785
17	-0.15214115	-0.28928971	1.64891768	1	0.92405885	2.36011028	0.76751763
1	-0.92584115	1.21361029	-0.41558236	1	2.65525889	-4.17809010	2.32181764
6	-2.64974117	0.84571028	-1.74148238	1	1.09825873	-3.53508973	1.78811765
6	-1.27824116	1.46231031	-1.46278238	1	2.36775899	-2.44948959	2.38761783
6	-0.18804115	0.87901026	-2.37768221	1	-1.48144114	3.33961034	-2.50728226
6	-1.29834116	2.99641037	-1.48438239	1	-0.35654116	3.42861032	-1.13648236
1	-3.41494107	1.59441030	-1.94478238	1	-2.09474111	3.39381027	-0.85128236
1	-2.60854101	0.15131028	-2.58218217	6	2.28395891	0.65681028	-2.93998218
6	1.24015880	1.27321029	-2.01258230	1	1.43325901	0.97031027	-0.96458232
1	-0.40024114	1.19721031	-3.40638232	1	1.35745883	2.36161041	-1.99698246
1	-0.26114115	-0.21728972	-2.37518215	1	3.30035901	0.85301030	-2.58838224
13	1.87245893	-0.51458973	0.71301764	1	2.19115901	1.08071029	-3.94338226
6	3.14295888	0.73171026	1.48971760	1	2.15045881	-0.42498970	-3.02588224

I-4Cl_bo

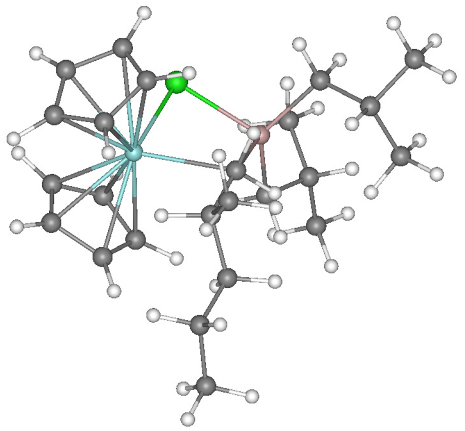

Zero-point vibrational energy	1577004.0 (Joules/Mol)
	376.91300 (Kcal/Mol)
Zero-point correction	0.600649 (Hartree/Particle)
Thermal correction to Energy	0.634267
Thermal correction to Enthalpy	0.635211
Thermal correction to Gibbs Free Energy	0.535875
Sum of electronic and zero-point Energies	-5181.496412
Sum of electronic and thermal Energies	-5181.462794
Sum of electronic and thermal Enthalpies	-5181.461850
Sum of electronic and thermal Free Energies	-5181.561186

cartesian

6	2.40123534	-1.30292070	-2.57488370	1	2.73113537	2.70547938	-1.91708374
6	3.59213543	-0.84212065	-1.93868375	1	1.00143540	2.75747943	-2.30178380
6	4.10023546	-1.90062070	-1.14988375	1	1.97873545	1.38247931	-2.81768370
6	3.21613550	-3.00622058	-1.27278376	1	0.89123535	1.92807937	1.33911622
6	2.18913531	-2.63952065	-2.18038368	1	0.32313538	3.03647923	0.11121619
1	1.36463547	-3.26732063	-2.48978376	1	4.64543533	-1.01242065	1.59521627
1	1.79133534	-0.75262070	-3.27878380	1	5.01643562	-1.88062072	-0.57548380
1	4.05103540	0.13167930	-2.06678367	13	-1.36046457	-0.95142066	-0.15488382
1	3.33423543	-3.97652078	-0.80588382	6	-2.39816451	-0.91972065	-1.81258380
6	1.41543531	-0.52532071	2.30001616	6	-1.96446466	-0.58292067	1.67241621
6	2.70593548	-0.00492066	2.00591636	1	-3.31146455	-1.51272070	-1.64598370
6	3.58593535	-1.09032071	1.79891622	6	-2.82036448	0.46797931	-2.33798385
6	2.84483552	-2.29422069	1.97851634	1	-1.84816456	-1.46112061	-2.59678364
6	1.51763535	-1.93862069	2.31441617	6	-3.43736458	-1.00602067	1.87411630
1	0.71123540	-2.63142061	2.51591635	1	-1.36056471	-1.12922072	2.41301632
1	0.52923536	0.04837930	2.54001617	1	-1.85686445	0.48497933	1.92001629
1	2.97763538	1.04317927	1.96761620	6	-3.60446453	1.24897933	-1.27928376
1	3.23753548	-3.30162072	1.91591620	6	-3.64866447	0.34357929	-3.62038374

40	1.92933536	-1.20922065	-0.08418381	1	-1.91856456	1.04667926	-2.58528376
17	-0.13566458	-2.89152074	-0.14308381	6	-3.92786455	-0.64552069	3.27841616
1	2.47853541	4.10077953	-0.10858381	6	-3.60916471	-2.50622082	1.61801624
6	1.19403541	2.44487929	0.42121619	1	-4.06086445	-0.46382070	1.15081620
6	2.36123538	3.39117932	0.71581620	1	-4.98046446	-0.91102070	3.41371632
6	2.15233541	4.16107941	2.01981616	1	-3.82176447	0.42597932	3.47111630
1	3.30443549	2.82877922	0.76351619	1	-3.34576464	-1.18392062	4.03481627
1	2.96783543	4.86357975	2.20071626	1	-3.91396475	2.22967935	-1.65208375
1	2.09643531	3.48147941	2.87641621	1	-3.02256465	1.41517937	-0.36148381
1	1.22003531	4.73157978	1.98861623	1	-4.50976467	0.70017934	-0.99648380
6	0.29473537	0.42367929	-0.84828383	1	-3.92096472	1.32637930	-4.01738405
6	1.46743536	1.41307926	-0.68518382	1	-4.57396460	-0.20722067	-3.42088366
6	1.82613540	2.09997940	-2.00918388	1	-3.09916449	-0.19652069	-4.39658356
1	0.10163540	0.26557934	-1.91368377	1	-4.64016438	-2.82512069	1.79511631
1	-0.57116467	0.99887931	-0.47698382	1	-2.96106458	-3.08692074	2.28511620
1	2.43613529	0.91697931	-0.36478382	1	-3.35946465	-2.78682065	0.58631617

TS-4Cl

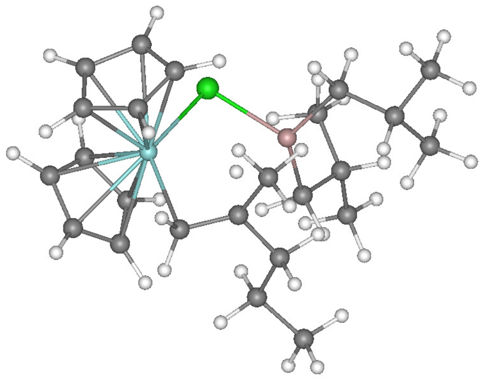

Zero-point vibrational energy	1565700.3 (Joules/Mol)
	374.21135 (Kcal/Mol)
Zero-point correction	0.596344 (Hartree/Particle)
Thermal correction to Energy	0.629335
Thermal correction to Enthalpy	0.630279
Thermal correction to Gibbs Free Energy	0.533415
Sum of electronic and zero-point Energies	-5181.470810
Sum of electronic and thermal Energies	-5181.437819
Sum of electronic and thermal Enthalpies	-5181.436874
Sum of electronic and thermal Free Energies	-5181.533738

cartesian

6	-2.60541773	-2.37368989	-0.70739561	6	1.80268228	-1.57938969	0.44980437
6	-3.93871784	-1.87228978	-0.68539560	1	2.05448198	1.89421022	2.15620446

6	-4.34391785	-1.79048967	0.66650438	6	2.66368198	2.45941019	0.16990438
6	-3.24841785	-2.16718984	1.48200440	1	0.77038217	2.63131022	1.24750447
6	-2.18081784	-2.55418992	0.62110436	6	2.98738241	-1.88628972	1.39850450
1	-1.21131778	-2.91308975	0.94320440	1	1.08358216	-2.40968990	0.52000439
1	-2.02871776	-2.59518981	-1.59419560	1	2.16698217	-1.58378971	-0.58489561
1	-4.55011797	-1.64398968	-1.54979563	6	3.84888220	1.49991024	0.04010439
1	-5.31981802	-1.47888970	1.01490438	6	3.14448214	3.84551024	0.60890436
1	-3.23331785	-2.19878983	2.56450438	1	2.21928215	2.56541014	-0.82909566
6	-3.62571764	2.23471022	0.05710438	6	3.56948185	-3.26908994	1.09330440
6	-4.61051798	1.35161030	0.55240440	6	2.57728243	-1.78968978	2.87080431
6	-4.25321770	1.01221025	1.89250445	1	3.77428198	-1.14398968	1.21920443
6	-3.04761791	1.66941023	2.20030427	1	4.44438219	-3.47858977	1.71570444
6	-2.63001776	2.39921021	1.05470443	1	3.87278223	-3.34698987	0.04520439
1	-1.75021780	3.02841020	0.99450439	1	2.82368231	-4.04788971	1.28780437
1	-3.65501785	2.71971011	-0.90839559	1	4.62688208	1.91911030	-0.60419559
1	-5.50541782	1.03981030	0.02790439	1	3.55218220	0.53511024	-0.38969558
1	-4.81981802	0.38391024	2.56760430	1	4.29798222	1.30851030	1.02190447
1	-2.51691771	1.61141026	3.14060426	1	3.86868191	4.25801039	-0.09939560
40	-2.54501772	-0.05028976	0.37360439	1	3.62828207	3.78561020	1.58980441
17	-0.69881779	-0.33408976	2.41840434	1	2.31008244	4.54861021	0.69010437
1	-0.61541778	0.39741024	-0.31179559	1	3.40048218	-2.07788992	3.53010440
6	-2.26191783	0.42491025	-2.02789569	1	1.72838223	-2.44958973	3.08510423
6	-0.93141782	0.86101019	-1.80829573	1	2.28548241	-0.76958978	3.15740442
6	0.25138217	0.13351025	-2.43409562	1	0.15498221	2.57581019	-1.01329565
1	-2.39941788	-0.49928975	-2.57869577	1	-1.55671787	2.90541005	-1.35639572
1	-2.99931765	1.19011033	-2.24869561	1	-0.41001779	2.71431017	-2.67929578
1	0.33468223	0.58051026	-3.43519568	6	1.42718232	-1.91928971	-3.29779577
1	1.17538226	0.42291024	-1.91249561	1	-0.69881779	-1.65928972	-3.16699576
6	0.18568218	-1.38098967	-2.58389568	1	0.08898222	-1.84698975	-1.59659553
6	-0.67811781	2.35171008	-1.68079567	1	1.40388215	-3.00898981	-3.35809565
13	0.91158223	0.10061024	0.91600436	1	2.34398222	-1.63088977	-2.77559566
6	1.60118210	1.91751027	1.14950442	1	1.48898208	-1.52648973	-4.31639576

I-5Cl

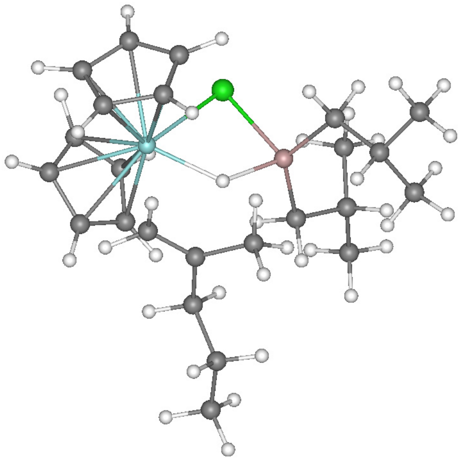

Zero-point vibrational energy	1567247.8 (Joules/Mol)
	374.58121 (Kcal/Mol)
Zero-point correction	0.596933 (Hartree/Particle)
Thermal correction to Energy	0.631370
Thermal correction to Enthalpy	0.632314
Thermal correction to Gibbs Free Energy	0.529704
Sum of electronic and zero-point Energies	-5181.483996
Sum of electronic and thermal Energies	-5181.449559
Sum of electronic and thermal Enthalpies	-5181.448615
Sum of electronic and thermal Free Energies	-5181.551225

cartesian

6	3.29454541	1.48925149	1.28334713	6	-0.08085442	1.01095152	-2.36625290
6	4.23164558	0.46655157	1.53904712	6	0.63854557	2.72735167	-0.62605286
6	3.62964559	-0.46114841	2.44024706	1	3.14754558	1.87585163	-1.40565288
6	2.33524561	0.01895159	2.75204706	1	2.56084561	0.63265157	-2.64045286
6	2.10484552	1.20105159	2.00994730	1	-1.00215435	0.93675160	-1.77815282
1	1.20884562	1.80765152	2.04474711	1	-0.29955447	1.66335154	-3.21965265
1	3.47234559	2.36345148	0.67224717	1	0.17994556	0.02275157	-2.75195265
1	5.24054527	0.42195156	1.14954710	6	-0.05635446	3.88885164	-1.36325288
1	4.09594584	-1.34674847	2.85474730	1	-0.05905443	2.32395148	0.12084714
1	1.62644553	-0.45904842	3.41444731	1	1.51334548	3.11765146	-0.09675285
6	2.70564556	-1.91074848	-1.87565291	6	-0.37835449	5.03265142	-0.40325284
6	3.96854544	-1.35824847	-1.49355292	1	-0.97895449	3.52995157	-1.82865298
6	4.39624548	-2.03004837	-0.32515287	1	0.59344554	4.24465132	-2.16965294
6	3.38294554	-2.94004846	0.05754714	1	-0.88425440	5.84745169	-0.92435288
6	2.35394549	-2.88574839	-0.92685282	1	0.53194559	5.43785143	0.04694714
1	1.44654560	-3.47554851	-0.91925287	1	-1.03335452	4.69295168	0.40424713
1	2.12444568	-1.64434850	-2.74995279	6	-3.54525471	0.75595152	3.35344696

1	4.53384542	-0.61024845	-2.03585291	6	-2.79175448	-1.61914849	3.00824690
1	5.32954550	-1.86474848	0.19724713	1	-3.82525444	-0.40644842	1.57904708
1	3.39664555	-3.59354854	0.92114711	6	-3.13085461	-2.84264851	-2.89455271
40	2.36754560	-0.66074842	0.30214715	1	-2.07945442	-0.99764836	-2.66225290
17	0.61774558	-2.29924846	1.62374711	6	-3.76125431	-0.97514844	-1.33755291
1	0.50594556	-0.23564842	-0.14955285	1	-4.47835445	0.42705157	3.82044697
13	-0.91445440	-1.06314838	0.43304715	1	-3.72585440	1.72715163	2.88364697
6	-1.59785461	-2.25294828	-0.96995282	1	-2.80375457	0.89795160	4.14784718
6	-1.76955462	0.24215159	1.62044716	1	-3.66895437	-1.95314848	3.56944704
6	-2.59925461	-1.71114850	-2.00955296	1	-1.95075440	-1.55384851	3.70924711
1	-0.75245446	-2.72634840	-1.49175286	1	-2.55825448	-2.40854836	2.28074694
1	-2.08615446	-3.08124852	-0.42905289	1	-4.50485468	-0.64954841	-2.07075310
6	-3.04095459	-0.26784843	2.33294725	1	-3.42595434	-0.08444841	-0.79115289
1	-1.03325438	0.52805161	2.39114714	1	-4.26735449	-1.63414848	-0.62155288
1	-2.00745440	1.17025161	1.07874715	1	-3.81035471	-2.46234846	-3.66325283
6	2.32914567	1.28115153	-1.80135286	1	-3.68175459	-3.57034850	-2.28885269
6	1.03504562	1.62495160	-1.56665289	1	-2.31515455	-3.37314844	-3.39455271

S3.8. $^{i}Bu_2AlMe$ reaction profile

I-0Me

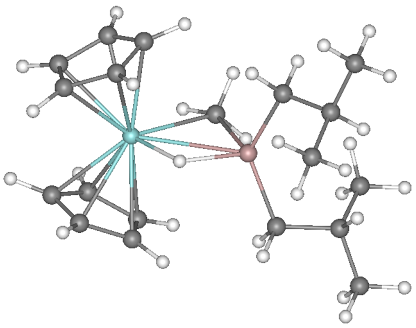

Zero-point vibrational energy	1209895.9 (Joules/Mol)
	289.17207 (Kcal/Mol)
Zero-point correction	0.460825 (Hartree/Particle)
Thermal correction to Energy	0.486866
Thermal correction to Enthalpy	0.487810
Thermal correction to Gibbs Free Energy	0.403556
Sum of electronic and zero-point Energies	-4525.543269
Sum of electronic and thermal Energies	-4525.517228
Sum of electronic and thermal Enthalpies	-4525.516284
Sum of electronic and thermal Free Energies	-4525.600538

cartesian

6	-3.38580179	1.90520573	1.37410378	6	1.36719811	-1.69039428	-0.40929621
6	-4.36060190	1.51810563	0.41930377	6	1.37989795	1.78040564	0.29200378
6	-3.83450174	1.78890562	-0.87529624	1	1.34979808	-1.84479427	-1.49849617
6	-2.53860188	2.34410572	-0.71509624	6	2.81989813	-1.81909430	0.09710379
6	-2.25630188	2.40360570	0.67600381	1	0.77939808	-2.52229428	0.01380379
1	-1.34530199	2.78450561	1.12200379	6	2.78319788	1.90980566	-0.33489621
1	-3.48820210	1.83650565	2.45140386	1	0.73569798	2.55670571	-0.15349621
1	-5.34420204	1.12150562	0.63930380	1	1.43349802	2.02680564	1.36330378
1	-4.34590197	1.62600565	-1.81639624	6	3.40479803	-3.19439435	-0.23449621
1	-1.88060200	2.67150569	-1.51019621	6	2.89839792	-1.55549431	1.60380375
6	-4.24540186	-1.57829428	-0.71859622	1	3.43069792	-1.06309438	-0.41349620
6	-3.24500179	-1.55619431	-1.72419620	6	3.35699797	3.31280565	-0.12039621
6	-2.07340193	-2.16479445	-1.19279623	6	2.74289799	1.57240570	-1.82809615
6	-2.34070206	-2.53019428	0.14740379	1	3.45269823	1.19410563	0.16020378
6	-3.68550205	-2.16179442	0.44520378	1	4.36529827	3.39980578	-0.53549623
1	-4.20330191	-2.33609438	1.38190377	1	3.40619802	3.56160569	0.94340378
1	-5.25910187	-1.21149433	-0.82199621	1	2.72529793	4.06090546	-0.61269623
1	-3.36240196	-1.17899430	-2.73319602	1	4.44769812	-3.27159429	0.08680379
1	-1.14140201	-2.32269430	-1.72089624	1	3.36639833	-3.39089441	-1.30939627

1	-1.65190196	-3.02349424	0.82260376	1	2.83759785	-3.98269439	0.27310380
40	-2.44640207	-0.03129431	0.08350379	1	3.91719818	-1.67579436	1.98220384
6	-0.80440199	-0.23319432	1.79000378	1	2.26039791	-2.26139426	2.15100384
1	-0.88380194	0.23170570	-1.00559616	1	2.58109808	-0.53559434	1.86580384
1	-0.15010196	-1.05879438	2.10480380	1	3.72789812	1.67630565	-2.29179621
1	-1.77720201	-0.55639434	2.23500395	1	2.05669785	2.24770570	-2.35369611
1	-0.52360195	0.65090567	2.37090397	1	2.40809822	0.54210567	-2.01079607
13	0.53869808	0.02720569	0.04480379				

I-0Me_a

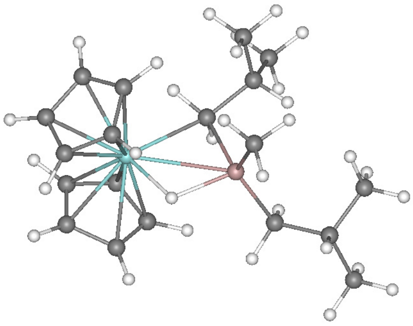

Zero-point vibrational energy	1212783.3 (Joules/Mol)
	289.86216 (Kcal/Mol)
Zero-point correction	0.461925 (Hartree/Particle)
Thermal correction to Energy	0.487740
Thermal correction to Enthalpy	0.488684
Thermal correction to Gibbs Free Energy	0.406455
Sum of electronic and zero-point Energies	-4525.542068
Sum of electronic and thermal Energies	-4525.516252
Sum of electronic and thermal Enthalpies	-4525.515308
Sum of electronic and thermal Free Energies	-4525.597537

cartesian

6	3.55746412	1.63991702	0.10151130	6	-2.00773597	-1.39618301	0.35551131
6	4.31506443	0.56221700	0.61941129	6	-1.05133581	1.43671703	2.16981125
6	3.71206403	0.14921699	1.83881128	1	-1.58143592	-2.35128307	0.70151132
6	2.59846425	0.99461693	2.08751106	6	-3.43903613	-1.25858295	0.91631132
6	2.49266410	1.90681696	1.00921130	1	-2.07713580	-1.49178302	-0.74118870
1	1.75886416	2.69551706	0.91021127	1	-0.17153585	2.04201698	2.41371107
1	3.76956415	2.18541694	-0.81138873	1	-1.84583592	2.13361692	1.87921131
1	5.20396423	0.13511698	0.17201129	6	-4.27533579	-2.50038290	0.59591132
1	4.06576443	-0.64028299	2.49071121	6	-4.12413549	-0.00438301	0.36811128
1	1.94936419	0.95111698	2.95281124	1	-3.38163590	-1.16308296	2.00871134
6	3.41796422	-1.98158300	-1.29168868	1	-5.28363562	-2.42048287	1.01271129

6	3.27526402	-2.55648303	0.00201130	1	-3.81363606	-3.40558290	1.00051129
6	1.90906405	-2.87718296	0.19241129	1	-4.36863565	-2.62568307	-0.48878872
6	1.20276415	-2.50598288	-0.98518872	1	-5.15593576	0.07371698	0.72171128
6	2.13576412	-1.96648300	-1.90238869	1	-4.15063572	-0.03118301	-0.72848868
1	1.91316414	-1.59938300	-2.89798880	1	-3.60803580	0.91331702	0.67481130
1	4.34496403	-1.66068292	-1.75098872	6	-1.19593573	1.81111705	-2.81128883
1	4.07506418	-2.73318291	0.71021128	6	-0.37943584	3.10141706	-0.80548871
1	1.47606421	-3.33708286	1.07171130	1	-1.80923581	1.50601709	-0.80248868
1	0.13876414	-2.63228297	-1.14848876	1	-1.94023585	2.58771706	-3.00488853
40	2.07086420	-0.37298301	0.04341129	1	-1.59623599	0.86021692	-3.17318869
6	0.16666415	0.60741699	-1.05608869	1	-0.29843587	2.05081701	-3.39168882
1	0.76936418	-0.76498300	1.38291132	1	-1.10773587	3.87971711	-1.04658866
1	-0.10373586	-0.21618301	-1.72648871	1	0.56706417	3.37591696	-1.28728867
1	1.12566423	1.02931702	-1.46528876	1	-0.24083585	3.10911703	0.27771130
6	-0.86313587	1.74531698	-1.31648874	1	-1.38483596	0.97651702	3.10871124
13	-0.75623578	0.03341699	0.84401131				

I-0Me_bo

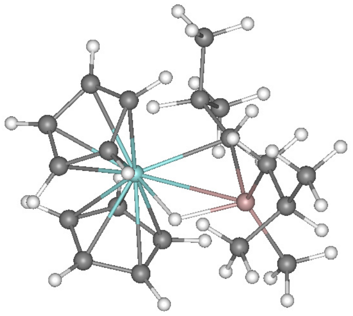

Zero-point vibrational energy	1212997.1 (Joules/Mol)
	289.91326 (Kcal/Mol)
Zero-point correction	0.462006 (Hartree/Particle)
Thermal correction to Energy	0.487794
Thermal correction to Enthalpy	0.488738
Thermal correction to Gibbs Free Energy	0.406857
Sum of electronic and zero-point Energies	-4525.551292
Sum of electronic and thermal Energies	-4525.525504
Sum of electronic and thermal Enthalpies	-4525.524560
Sum of electronic and thermal Free Energies	-4525.606441

cartesian

6	1.72263396	-1.61271322	2.13605475	6	-1.41226602	1.69148684	-2.32694530
6	2.24903393	-2.54761314	1.21355462	6	-2.73966599	0.25968680	0.52695471
6	1.16863394	-3.04921341	0.43475473	1	-2.06626606	1.18358684	-3.04584527

6	-0.02216601	-2.42771339	0.88535476	1	-0.46176600	1.87738669	-2.83894539
6	0.31973398	-1.51811326	1.92135477	6	-4.01106596	-0.14761320	-0.24814527
1	-0.37906605	-0.90941322	2.48255467	1	-2.49326611	-0.54021323	1.24285483
1	2.29353380	-1.07591319	2.88475466	1	-2.96226597	1.14358675	1.14285469
1	3.28443408	-2.85641336	1.14415479	6	-5.19256592	-0.37841320	0.69725472
1	1.23793399	-3.80521297	-0.33704528	6	-3.75396585	-1.39931321	-1.09184527
1	-1.01966608	-2.61321306	0.50715470	1	-4.27946615	0.67078680	-0.92944527
6	3.46073389	-1.15031314	-1.49574518	1	-6.09996605	-0.64121318	0.14565472
6	2.35023403	-1.58381319	-2.27214527	1	-5.40446615	0.51548678	1.29025483
6	1.61533403	-0.43661320	-2.66514540	1	-4.96996593	-1.19751322	1.39015484
6	2.24783397	0.70348680	-2.10624528	1	-4.64606619	-1.70211315	-1.64694524
6	3.39993405	0.25988680	-1.39734530	1	-3.46566606	-2.24031305	-0.44894528
1	4.11953402	0.88948679	-0.88674527	1	-2.95306611	-1.24851322	-1.82964516
1	4.23533392	-1.78301322	-1.08144522	6	2.59473395	2.82398701	0.72965473
1	2.12813401	-2.60551310	-2.55364537	1	2.45973396	0.74778682	1.09135485
1	0.73013401	-0.42981321	-3.28824544	6	1.60293400	1.71758676	2.76265478
1	1.93593395	1.73078680	-2.24904537	1	3.58723402	2.83458662	1.18845463
40	1.40003395	-0.63571322	-0.16224527	1	2.08743382	3.75478697	0.99785477
6	0.41843396	1.50058675	0.51125473	1	2.71343398	2.80948687	-0.35574529
1	-0.30966604	-0.75771320	-1.01324534	1	2.57413387	1.68668675	3.26425457
1	0.33023399	2.34628677	-0.18314527	1	0.98153400	0.90938675	3.15475464
6	1.76613402	1.63878679	1.23965478	1	1.11953402	2.66428661	3.01935458
1	-0.36776602	1.60238683	1.27095461	1	-1.87186599	2.66958666	-2.14264536
13	-1.25266600	0.66138679	-0.67934525				

I-1Me

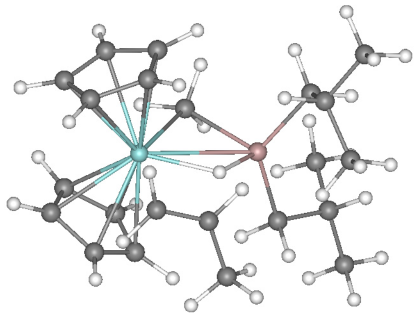

Zero-point vibrational energy	1431865.4 (Joules/Mol)
	342.22404 (Kcal/Mol)
Zero-point correction	0.545369 (Hartree/Particle)
Thermal correction to Energy	0.575964
Thermal correction to Enthalpy	0.576908
Thermal correction to Gibbs Free Energy	0.485519
Sum of electronic and zero-point Energies	-4643.314598

Sum of electronic and thermal Energies -4643.284003
Sum of electronic and thermal Enthalpies -4643.283058
Sum of electronic and thermal Free Energies -4643.374448

cartesian

6	-2.69293213	-2.09581947	-1.40069187	1	0.57376766	-1.44681942	2.22470808
6	-3.57553244	-2.17311931	-0.30239192	1	-1.07793236	-1.31291938	2.54560804
6	-2.82423210	-2.60671926	0.82900810	1	-0.07723227	0.08578062	2.81800818
6	-1.48663235	-2.80881929	0.40830809	1	-0.70173228	1.79238057	-2.13719177
6	-1.39063227	-2.46041942	-0.95989192	13	1.04066777	-0.07851937	0.54240811
1	-0.49543229	-2.52351928	-1.56739187	6	1.75536764	1.67858064	1.05610812
1	-2.96973228	-1.84051943	-2.41369200	6	2.06016779	-1.55231941	-0.26279193
1	-4.63983250	-1.97441947	-0.32599193	1	2.84866762	1.62138057	1.16710806
1	-3.22023249	-2.79691935	1.81990814	6	1.43336773	2.76278067	0.00650809
1	-0.66933227	-3.15551925	1.02710807	1	1.38286769	1.99628055	2.04050803
6	-2.74803209	2.06588078	0.61200809	6	3.51116753	-1.61001945	0.26310807
6	-3.91503239	1.25758064	0.46900809	1	1.58186781	-2.52801919	-0.07459191
6	-4.00533247	0.42538065	1.61180806	1	2.08596754	-1.44781947	-1.35819185
6	-2.88183212	0.67828065	2.42970800	6	2.01356792	2.38138080	-1.35969186
6	-2.10773230	1.70348060	1.81180811	6	1.95006764	4.13958025	0.43190807
1	-1.18653226	2.13088059	2.18980813	1	0.33766770	2.83438063	-0.09709191
1	-2.40203238	2.82948065	-0.07439191	6	4.29886770	-2.74891925	-0.38869193
1	-4.64413261	1.31008053	-0.32989192	6	3.53376770	-1.75201941	1.78780806
1	-4.79403257	-0.28741938	1.81790817	1	4.00726748	-0.66431934	0.00520809
1	-2.66773224	0.20448063	3.37920809	1	5.33786774	-2.76401925	-0.04649191
40	-1.98033226	-0.32391936	0.28390810	1	4.30246782	-2.65281940	-1.47819185
6	-0.26013228	-0.72991937	2.11160803	1	3.84776783	-3.71541929	-0.13739192
1	-0.30183229	0.27508062	-0.48169193	1	1.78256762	3.13008070	-2.12439179
6	-2.61463213	0.97148067	-2.37939191	1	1.63806760	1.41198063	-1.71989191
6	-1.27263236	0.94338065	-2.51429200	1	3.10436773	2.29638076	-1.29789186
6	-0.49753228	-0.07721937	-3.28999186	1	1.70386779	4.90898037	-0.30669191
1	-3.25093222	0.22498064	-2.84569192	1	3.03916788	4.11508036	0.54240811
1	-3.10973215	1.85348058	-1.99119186	1	1.52496779	4.44168043	1.39330816
1	-0.11033228	0.40158063	-4.19579220	1	4.55456781	-1.84151947	2.16950822
1	0.36776769	-0.43951935	-2.72779179	1	2.98706770	-2.65191936	2.09760809
1	-1.11493230	-0.92381936	-3.59669185	1	3.08126783	-0.88721937	2.29310822

TS-1Me

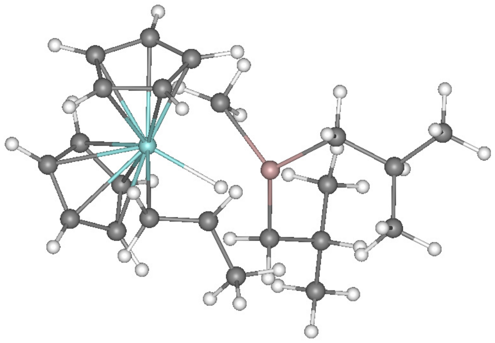

Zero-point vibrational energy	1430503.6 (Joules/Mol)
	341.89856 (Kcal/Mol)
Zero-point correction	0.544850 (Hartree/Particle)
Thermal correction to Energy	0.574495
Thermal correction to Enthalpy	0.575439
Thermal correction to Gibbs Free Energy	0.485963
Sum of electronic and zero-point Energies	-4643.305193
Sum of electronic and thermal Energies	-4643.275548
Sum of electronic and thermal Enthalpies	-4643.274604
Sum of electronic and thermal Free Energies	-4643.364080

cartesian

6	2.32494211	-2.28705168	1.20394516	1	0.38244197	-0.73685163	2.58134532
6	3.06564188	-1.41925168	2.05774522	1	1.40214193	0.68114841	2.45234537
6	4.25264168	-1.05385160	1.38774514	1	0.01304197	-0.68405163	-3.99355483
6	4.24374199	-1.66455150	0.10684520	1	0.84414196	-2.00375175	-1.90195489
6	3.06354189	-2.45085168	0.01394520	13	-0.76095808	-0.03725161	0.41474518
1	2.77794194	-3.07245159	-0.82575476	6	-1.53765798	1.64064837	-0.26255479
1	1.37494195	-2.75535178	1.43444514	6	-1.66175795	-1.77195168	0.62854522
1	2.78714204	-1.12145162	3.06014538	1	-1.58705807	1.65624833	-1.36045480
1	5.03374195	-0.41815162	1.78484511	6	-2.94945812	1.92134833	0.30454519
1	5.02234173	-1.58335161	-0.64195478	1	-0.89015806	2.48984838	0.00874520
6	3.21364188	2.10254836	1.12144518	6	-3.15345812	-1.83345151	0.24174519
6	4.09384203	1.81094837	0.03954519	1	-1.12575805	-2.55005169	0.05864520
6	3.37354207	1.96444845	-1.16195488	1	-1.55295801	-2.07445168	1.68164515
6	2.03664207	2.33954835	-0.83515477	6	-3.45705795	3.29664826	-0.13605481
6	1.95894194	2.45974827	0.57204521	6	-2.96485806	1.81274843	1.83194518
1	1.08094192	2.74944830	1.13524520	1	-3.63965797	1.16624832	-0.09265481
1	3.47584200	2.10304832	2.17294526	6	-3.75745821	-3.19505167	0.59734523
1	5.14074183	1.54654837	0.11844520	6	-3.35255814	-1.54015160	-1.24675488
1	3.78614187	1.85524845	-2.15475464	1	-3.69565821	-1.06785154	0.81344521
1	1.23884201	2.56214833	-1.53475487	1	-4.82245827	-3.23645163	0.35034519

40	2.35394192	-0.00485160	0.08614519	1	-3.64925814	-3.40915155	1.66434515
6	-0.00735807	-0.27655160	-2.97745466	1	-3.25125813	-3.99225163	0.04144520
6	1.07584190	-0.97965163	-2.19675469	1	-4.47445822	3.47984838	0.22184519
6	2.42124200	-0.63125163	-2.34985471	1	-3.45985794	3.38484836	-1.22615480
1	0.60684198	-0.44725162	-0.76765478	1	-2.81215811	4.08724833	0.26414520
1	-1.00235808	-0.45035160	-2.56455469	1	-3.94755793	2.05974841	2.24344540
1	0.17844194	0.79774839	-3.04075480	1	-2.23935795	2.50624824	2.27674532
1	3.17444205	-1.41035151	-2.35175467	1	-2.72545815	0.79674840	2.17934537
1	2.63044190	0.22484839	-2.98155475	1	-4.40645790	-1.61125159	-1.53085482
6	0.53194195	0.18774839	2.01594520	1	-2.79705811	-2.26425171	-1.85665488
1	-0.26735806	0.88374841	2.33944535	1	-3.01175809	-0.53255159	-1.51525486

I-2Me_a

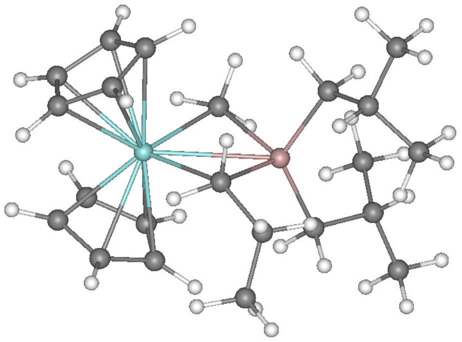

Zero-point vibrational energy	1441645.2 (Joules/Mol)
	344.56147 (Kcal/Mol)
Zero-point correction	0.549094 (Hartree/Particle)
Thermal correction to Energy	0.578996
Thermal correction to Enthalpy	0.579940
Thermal correction to Gibbs Free Energy	0.490410
Sum of electronic and zero-point Energies	-4643.333929
Sum of electronic and thermal Energies	-4643.304027
Sum of electronic and thermal Enthalpies	-4643.303083
Sum of electronic and thermal Free Energies	-4643.392612

cartesian

6	-3.52572870	-2.09423542	0.73813713	1	-0.10582894	-0.97483546	2.20883703
6	-2.18292904	-2.47163534	0.46183711	1	-1.72322893	-0.53913546	2.44973707
6	-1.93322897	-2.23363543	-0.90706289	1	-0.52542889	0.72476453	2.49733710
6	-3.12792873	-1.72133553	-1.49006283	13	0.61807108	0.06056455	0.26253712
6	-4.11352873	-1.65313542	-0.47796288	6	1.55577099	1.70786452	0.78893709
40	-2.36502886	0.02956454	0.14613712	6	1.48237097	-1.68393552	-0.04616288
6	-0.63032889	0.79546452	-1.36576283	1	2.17357111	1.49236453	1.67603719

6	-0.81002891	-0.17463546	1.94153714	6	2.45687103	2.39496469	-0.25856286
6	-2.50562906	2.43566465	0.93463707	1	0.80427110	2.43346477	1.13993716
6	-3.27752876	1.67056453	1.84993720	6	2.74467087	-1.92763543	0.81483710
6	-4.38412905	1.13236451	1.15383720	1	0.78627110	-2.52003527	0.13463712
6	-4.30592871	1.57196450	-0.19876289	1	1.77557099	-1.77683544	-1.10206282
6	-3.15752888	2.39356470	-0.32056287	6	3.63217115	1.49666452	-0.65236288
1	-1.00562894	-2.43553543	-1.42316282	6	2.97907114	3.73826456	0.26093712
1	-3.27022886	-1.46383548	-2.53346300	1	1.86437118	2.60176468	-1.16266286
1	-1.47422886	-2.87233543	1.17613721	6	3.39537096	-3.26663542	0.45713714
1	-4.02862883	-2.17833543	1.69473720	6	2.43547106	-1.87673545	2.31333709
1	-5.13722897	-1.32503545	-0.60716289	1	3.46987104	-1.13293552	0.59923708
1	-5.16612911	0.51496452	1.57843721	1	4.31627083	-3.42853546	1.02533710
1	-3.05732870	1.52736449	2.90063715	1	3.64237118	-3.31353545	-0.60726291
1	-1.59662890	2.97756457	1.16633713	1	2.71277094	-4.09453583	0.68073708
1	-2.83082914	2.89026475	-1.22606289	1	4.26547098	1.97516453	-1.40486288
1	-5.02332926	1.36106455	-0.98256296	1	3.30597115	0.53066450	-1.05766284
1	-1.62322891	0.66356450	-1.87336290	1	4.25607109	1.28666449	0.22413711
1	-0.57212889	1.87236452	-1.16556287	1	3.60477114	4.23926449	-0.48396286
6	0.37147105	0.50406456	-2.52076292	1	3.58357096	3.58566475	1.16183710
6	0.11157107	-0.78063548	-3.30116296	1	2.15617085	4.41106462	0.52003711
1	1.40487111	0.49906456	-2.15216303	1	3.31847095	-2.12633538	2.90823698
1	0.73427105	-0.80513549	-4.19816256	1	1.65297115	-2.60243535	2.57293701
1	-0.93262893	-0.84443545	-3.62656307	1	2.10487127	-0.88183546	2.63693714
1	0.35187113	-1.67283547	-2.72106290	1	0.32457107	1.35406458	-3.20906305

I-2Me_bi

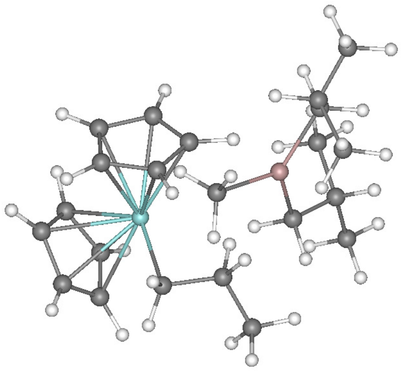

Zero-point vibrational energy	1438065.7 (Joules/Mol)
	343.70594 (Kcal/Mol)
Zero-point correction	0.547730 (Hartree/Particle)
Thermal correction to Energy	0.578739
Thermal correction to Enthalpy	0.579683
Thermal correction to Gibbs Free Energy	0.482591

Sum of electronic and zero-point Energies	-4643.319937
Sum of electronic and thermal Energies	-4643.288928
Sum of electronic and thermal Enthalpies	-4643.287984
Sum of electronic and thermal Free Energies	-4643.385077

cartesian

6	-3.63423848	1.96965325	0.01978227	1	-0.20193857	0.61075324	0.11188227
6	-4.33253860	1.30425322	1.05638230	1	-0.10143858	-1.17774677	-0.34761775
6	-3.42953849	1.11315322	2.13968229	1	-0.14953858	-0.67374676	1.40828228
6	-2.17173862	1.62425327	1.75678229	1	-2.36533856	1.26135325	-2.70471764
6	-2.29463863	2.14985347	0.43858224	13	2.14006138	-0.18174675	0.35148227
1	-1.51123857	2.64085340	-0.12951772	6	2.71456146	1.65885329	0.68888223
1	-4.05253887	2.28695345	-0.92571777	6	3.19036150	-1.76474679	-0.08461772
1	-5.37913895	1.02505326	1.03928232	1	3.66996169	1.84625328	0.17628227
1	-3.66383862	0.65545326	3.09248233	6	1.68816137	2.72775340	0.26628226
1	-1.27553856	1.62975323	2.36638236	1	2.94276142	1.77355325	1.75898230
6	-2.83073854	-2.87484670	-0.09341772	6	4.71326113	-1.60104680	0.09088227
6	-4.12503862	-2.39364672	0.25138226	1	2.84636140	-2.62024665	0.51798224
6	-4.07003880	-1.91804671	1.57958233	1	2.96106148	-2.04684663	-1.12451768
6	-2.73993850	-2.09444666	2.06218219	6	1.39156151	2.64105344	-1.23491764
6	-1.99393868	-2.71964669	1.03338230	6	2.16116142	4.13895321	0.62758225
1	-0.95373857	-3.01484656	1.09418225	1	0.75126141	2.54565334	0.81678224
1	-2.54803848	-3.31664658	-1.04171765	6	5.45936108	-2.85364652	-0.37611777
1	-4.99993896	-2.40074658	-0.38491774	6	5.05956125	-1.28564680	1.54828227
1	-4.90103865	-1.50924671	2.14028215	1	5.04876137	-0.75814676	-0.52771777
1	-2.38443851	-1.85484672	3.05688238	1	6.54216146	-2.73104668	-0.28001773
40	-2.63703847	-0.35644674	0.26268226	1	5.23606110	-3.08124661	-1.42221773
6	0.12766141	-0.39574674	0.38518226	1	5.16366148	-3.71884656	0.22738227
1	-1.44433856	0.33275324	-1.58731771	1	0.66466147	3.39575338	-1.55291772
6	-3.54543853	-0.33034673	-1.80811763	1	0.99716145	1.65715325	-1.53301764
6	-2.24203849	0.22525325	-2.37561774	1	2.30796146	2.80775332	-1.81191766
6	-1.60633850	-0.62964672	-3.47541761	1	1.41776156	4.89435339	0.35408229
1	-4.41023874	0.29645324	-2.01931763	1	3.09046149	4.37285328	0.09788227
1	-3.73813844	-1.33304679	-2.19331765	1	2.35626149	4.22535324	1.69968235
1	-0.65093857	-0.21604675	-3.80771780	1	6.13906145	-1.18724680	1.69138229
1	-2.27413869	-0.68454677	-4.33761787	1	4.70926142	-2.08864665	2.20718241
1	-1.43403864	-1.65134680	-3.12251759	1	4.60246134	-0.34634674	1.88628232

I-2Me_bo

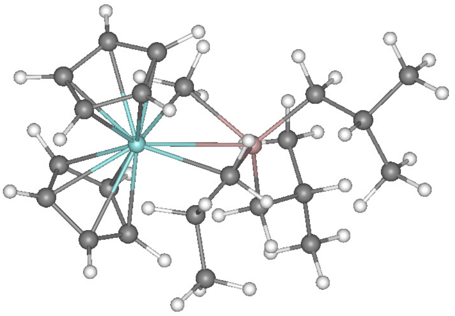

Zero-point vibrational energy	1445477.8 (Joules/Mol)
	345.47749 (Kcal/Mol)
Zero-point correction	0.550553 (Hartree/Particle)
Thermal correction to Energy	0.579911
Thermal correction to Enthalpy	0.580855
Thermal correction to Gibbs Free Energy	0.493244
Sum of electronic and zero-point Energies	-4643.336456
Sum of electronic and thermal Energies	-4643.307099
Sum of electronic and thermal Enthalpies	-4643.306155
Sum of electronic and thermal Free Energies	-4643.393766

cartesian

6	-3.12375951	2.33801627	0.08799675	1	-1.42365956	-0.72818387	2.53339672
6	-4.23115969	1.44631612	0.09369675	1	-0.37485963	0.66981614	2.60379672
6	-4.27935982	0.82761616	1.37209678	1	0.22524035	-0.95898384	2.27349687
6	-3.18795967	1.30501616	2.13059688	1	-1.92195964	-0.06658386	-4.16770315
6	-2.46745968	2.23731613	1.33259678	13	0.78584039	0.02891614	0.38569674
1	-1.58055961	2.78301620	1.63219678	6	1.83474040	1.60551620	0.91679680
1	-2.82925963	2.97691607	-0.73540330	6	1.58634043	-1.67228389	-0.20230326
1	-4.94735956	1.31121612	-0.70820320	1	2.61074042	1.28541613	1.63069677
1	-2.95275950	1.02771616	3.15039682	6	2.53324032	2.39301610	-0.21370324
6	-1.67495966	-2.26418376	-0.86890328	1	1.19724035	2.29921627	1.48729682
6	-2.95025969	-1.88348389	-1.37350321	6	2.96884036	-1.94618380	0.43169674
6	-3.86445951	-1.86368382	-0.29750326	1	0.94924033	-2.54218388	0.02039675
6	-3.16145968	-2.24418378	0.88089681	1	1.69734049	-1.66948378	-1.29910326
6	-1.81925964	-2.51388383	0.51729679	6	3.48804045	1.50321615	-1.01400328
1	-1.03365970	-2.84478378	1.18479681	6	3.28994036	3.60211611	0.34449676
1	-0.76935959	-2.40888381	-1.44380319	1	1.77034044	2.77591610	-0.90670323
1	-3.18465972	-1.66348386	-2.40850329	6	3.59624052	-3.22078395	-0.13890325
1	-3.58975959	-2.35258389	1.87049675	6	2.86334038	-2.04668379	1.95639682
40	-2.21565962	-0.03078386	0.20219675	1	3.63534021	-1.10738385	0.19549675
6	-0.57745963	-0.24048385	2.03189683	1	4.59184027	-3.39678383	0.27939677

6	-1.11095965	0.04191614	-3.44400311	1	3.69204044	-3.16108394	-1.22680318
6	-0.47505963	0.98271620	-1.13830316	1	2.97414041	-4.09158373	0.09749675
6	-1.53165960	0.90581614	-2.25570321	1	3.98114061	2.06511617	-1.81250322
1	-1.77725959	1.91531610	-2.59640312	1	2.97854042	0.64841616	-1.47920322
1	-0.37375963	2.01501608	-0.79070330	1	4.26984024	1.10161614	-0.35950324
1	0.47934043	0.74771619	-1.63710320	1	3.76294041	4.18241596	-0.45360324
1	-2.54135966	0.53741616	-1.93000317	1	4.07584047	3.27141619	1.03209674
1	-0.79055965	-0.95348382	-3.12810326	1	2.62104034	4.26761627	0.89809680
1	-0.26785964	0.51361614	-3.95440340	1	3.82404041	-2.31188393	2.40679669
1	-4.91815948	-1.62308383	-0.36370325	1	2.14194036	-2.82348394	2.24209690
1	-5.02525949	0.11891615	1.70949674	1	2.54824042	-1.10078382	2.41599679

I-4Me_a

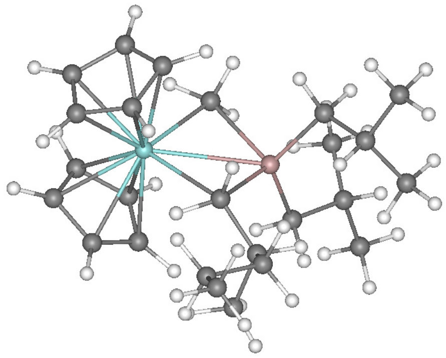

Zero-point vibrational energy	1668209.5 (Joules/Mol)
	398.71165 (Kcal/Mol)
Zero-point correction	0.635387 (Hartree/Particle)
Thermal correction to Energy	0.669386
Thermal correction to Enthalpy	0.670330
Thermal correction to Gibbs Free Energy	0.571478
Sum of electronic and zero-point Energies	-4761.137088
Sum of electronic and thermal Energies	-4761.103090
Sum of electronic and thermal Enthalpies	-4761.102145
Sum of electronic and thermal Free Energies	-4761.200997

cartesian

6	3.43406200	-2.66643810	0.76999581	1	1.79796195	3.32066178	0.77989584
6	2.10206199	-2.72813821	1.26329577	1	1.32886195	3.42906189	2.47299576
6	1.87066197	-1.57763815	2.04739571	1	1.97166193	5.67136192	1.57789576
6	3.06746197	-0.80463821	2.05939579	1	0.26766199	5.66046190	2.04229593
6	4.03526211	-1.48683810	1.28619587	1	0.71696198	5.54986191	0.33789581
40	2.25566196	-0.76163816	-0.32090417	1	-0.01763803	-2.92883825	-0.93450421
6	0.55136198	0.88356179	0.21849582	1	1.57916200	-2.80543804	-1.49140418

6	0.66796196	-2.18913817	-1.36750424	1	0.34266198	-1.98053813	-2.39250422
6	2.35296202	0.33546180	-2.60340428	13	-0.73523808	-0.76303822	-0.38790417
6	3.11006212	-0.86223817	-2.70550418	6	-1.65523803	0.00166181	-1.95020413
6	4.23846197	-0.74513817	-1.86170423	6	-1.64433801	-1.74633813	1.05809581
6	4.18856192	0.53496182	-1.23890424	1	-2.29873800	-0.77233821	-2.39970422
6	3.03576183	1.20416188	-1.71870422	6	-2.51503801	1.26116180	-1.71920419
1	0.95386195	-1.34583831	2.57009578	1	-0.90373802	0.22466180	-2.72440410
1	3.22286177	0.12606180	2.59339571	6	-2.89313793	-2.53843808	0.60089582
1	1.38576198	-3.51673818	1.06909585	1	-0.96483803	-2.44623804	1.57369578
1	3.92306185	-3.41273808	0.15469584	1	-1.97333801	-1.04243827	1.83619583
1	5.05656195	-1.16863823	1.11919582	6	-3.69643784	0.96776181	-0.79050416
1	5.01506233	-1.48823810	-1.72940421	6	-3.02483797	1.83646178	-3.04430413
1	2.86506176	-1.71603823	-3.32500410	1	-1.89173806	2.03416181	-1.24510419
1	1.43386197	0.55466181	-3.13300419	6	-3.57483768	-3.21063805	1.79619586
1	2.72416210	2.20336175	-1.43890417	6	-2.56363821	-3.58273816	-0.46870419
1	4.92406225	0.94466180	-0.55750418	1	-3.60633802	-1.82923818	0.16169584
1	1.55966198	1.11556172	0.65599585	1	-4.48903799	-3.73093820	1.49509585
1	0.50646198	1.49606180	-0.69210416	1	-3.83903790	-2.47763824	2.56379580
6	-0.40983802	1.55716181	1.24589586	1	-2.90313816	-3.94723821	2.25159574
6	-0.33153802	3.09226179	1.12189579	1	-4.28723812	1.86876178	-0.60170418
6	-0.16193801	1.10606170	2.68699574	1	-3.37963820	0.56846184	0.18109581
1	-1.44683802	1.29246187	0.99299586	1	-4.36013794	0.22416180	-1.24670422
1	-0.72693801	1.73706174	3.37889576	1	-3.62503767	2.73756194	-2.88440418
1	0.89596200	1.18796182	2.95989585	1	-3.65163803	1.10066175	-3.55950427
1	-0.48443806	0.07746181	2.85619593	1	-2.19633818	2.09556198	-3.71030426
1	-1.08933806	3.52236176	1.78799582	1	-3.44483805	-4.17893839	-0.72200418
1	-0.61653805	3.38666177	0.10389582	1	-1.79353797	-4.27833796	-0.10920417
6	1.02686203	3.71066189	1.45809579	1	-2.20943785	-3.12763810	-1.40190423
6	0.99706197	5.23596191	1.34739578				

I-4Me_bi

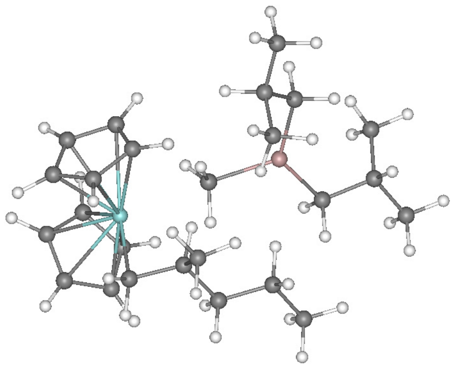

Zero-point vibrational energy 1665611.7 (Joules/Mol)

	398.09075 (Kcal/Mol)
Zero-point correction	0.634398 (Hartree/Particle)
Thermal correction to Energy	0.668909
Thermal correction to Enthalpy	0.669853
Thermal correction to Gibbs Free Energy	0.567649
Sum of electronic and zero-point Energies	-4761.126077
Sum of electronic and thermal Energies	-4761.091567
Sum of electronic and thermal Enthalpies	-4761.090622
Sum of electronic and thermal Free Energies	-4761.192827

cartesian

6	-3.76286173	1.82410979	-0.52804798	6	2.46563816	2.16410995	0.72625208
6	-4.52256203	1.51990986	0.62715209	6	3.16203833	-1.29519022	0.95335203
6	-3.71416187	1.78990984	1.76785207	1	3.42003822	2.24830985	0.18515204
6	-2.45166183	2.22250986	1.31485200	6	1.40043831	3.02251005	0.01615204
6	-2.47386169	2.23410988	-0.11004797	1	2.67003822	2.58880997	1.72105193
1	-1.66126180	2.54621005	-0.75834793	6	4.67413807	-0.99769014	0.97465205
1	-4.11776161	1.76690984	-1.54704785	1	2.90713835	-1.93779016	1.81115198
1	-5.55316162	1.18690979	0.63745201	1	2.91163826	-1.90209019	0.06835204
1	-4.01486206	1.68390989	2.80255198	6	1.12353826	2.49430990	-1.39534807
1	-1.61536181	2.50540996	1.94365215	6	1.81463814	4.49591017	-0.04494795
6	-2.91196179	-2.70409012	1.08725202	1	0.46363819	2.96771002	0.59395206
6	-4.22316170	-2.15288997	1.10295200	6	5.48923826	-2.28928995	0.86435205
6	-4.30416203	-1.26359022	2.19795203	6	5.05853844	-0.23189013	2.24305201
6	-3.03796172	-1.23879015	2.84745193	1	4.92113829	-0.36819014	0.10955204
6	-2.19486189	-2.15918994	2.17245197	1	6.56363821	-2.08349013	0.85255206
1	-1.17326176	-2.39778996	2.44065189	1	5.23983812	-2.83959007	-0.04764795
1	-2.54016161	-3.43409014	0.37895203	1	5.28053808	-2.94259000	1.71875215
1	-5.02176189	-2.38688993	0.41085204	1	0.40563822	3.12180996	-1.93494797
1	-5.18286180	-0.70719016	2.49785209	1	0.72453821	1.46820986	-1.38574791
1	-2.78696179	-0.67309016	3.73625207	1	2.04733825	2.47610998	-1.98474813
40	-2.70476174	-0.19559012	0.60035205	1	1.04443824	5.10991001	-0.52194798
6	0.01663822	-0.05559013	1.10085201	1	2.73853827	4.60340977	-0.62254798
1	-1.25616169	0.04790987	-1.17344785	1	1.99723816	4.89541006	0.95605206
6	-3.19966173	-0.93159014	-1.48524785	1	6.13293839	-0.03259013	2.28015208
6	-1.78196180	-0.58639014	-1.94574809	1	4.79753828	-0.81469017	3.13405204
6	-0.89616179	-1.83479011	-2.09214807	1	4.54713821	0.73710984	2.31025195
1	-3.96906185	-0.42449012	-2.06694794	1	-2.14546180	-0.25019014	-4.04834795
1	-3.38206172	-2.00659013	-1.52804804	1	-0.75666177	0.66550982	-3.42664790
6	0.58813822	-1.56079018	-2.33734798	1	-2.40256166	1.18690979	-3.05064797
1	-1.29566169	-2.44168997	-2.91494799	6	1.41723824	-2.84439015	-2.31884813

1	-0.99516177	-2.45599008	-1.18964791	1	0.96553826	-0.86889017	-1.56694794
1	-0.36076176	0.80780983	0.54285204	1	0.72553825	-1.04939020	-3.29494810
1	-0.14276177	-1.03219020	0.62875205	1	2.47423816	-2.64089012	-2.50564814
1	-0.28176177	-0.04209013	2.15425205	1	1.07053828	-3.53919005	-3.08904791
6	-1.76316178	0.31060988	-3.18984795	1	1.33503819	-3.35229015	-1.35244799
13	2.00433826	0.27340987	0.94625205				

I-4Me_bo

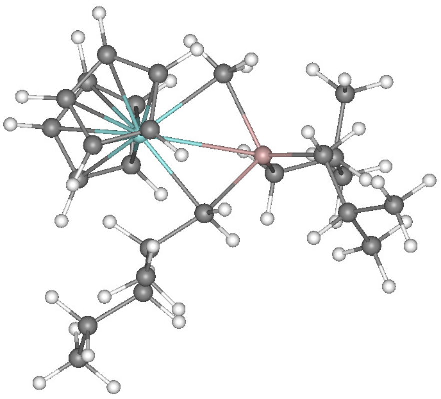

Zero-point vibrational energy	1671569.5 (Joules/Mol)
	399.51469 (Kcal/Mol)
Zero-point correction	0.636667 (Hartree/Particle)
Thermal correction to Energy	0.670064
Thermal correction to Enthalpy	0.671008
Thermal correction to Gibbs Free Energy	0.574389
Sum of electronic and zero-point Energies	-4761.138220
Sum of electronic and thermal Energies	-4761.104823
Sum of electronic and thermal Enthalpies	-4761.103878
Sum of electronic and thermal Free Energies	-4761.200498

cartesian

6	2.09623671	-2.15178728	-1.97207606	1	2.01823664	0.33881265	-3.15957594
6	3.34543681	-1.58198738	-1.59627604	1	1.14743674	2.46521282	0.51042396
6	3.83313680	-2.29548740	-0.47737604	1	0.66263676	3.11081266	-1.04047608
6	2.88493681	-3.30108738	-0.14917605	1	4.52553654	-0.65588737	1.89052391
6	1.82763672	-3.22268724	-1.09077609	1	4.78103638	-2.12828732	0.01602396
1	0.96463668	-3.87298727	-1.13157606	1	0.51603675	-3.05658722	1.39822388
1	1.48053670	-1.86068738	-2.81347609	1	-0.69676328	-3.11658716	0.15462396
1	3.85243678	-0.76398736	-2.09457588	1	-0.98026323	-2.31418753	1.70162392
1	2.98263669	-4.03488731	0.64202398	13	-1.22986329	-0.68008733	0.16382396
6	1.40663671	0.47941267	2.27842402	6	-2.31626320	-1.33128738	-1.34107614
6	2.74693680	0.68131262	1.85062397	6	-1.93206334	0.38131267	1.66782391

6	3.47083664	-0.50948739	2.08192396	1	-3.20376325	-1.84518731	-0.93737608
6	2.58213687	-1.45458734	2.66862392	6	-2.80326319	-0.28778735	-2.36977601
6	1.31943667	-0.83078736	2.81002402	1	-1.75336313	-2.11578751	-1.87277603
1	0.43663672	-1.27798736	3.24812412	6	-3.41526318	0.08401266	1.98332393
1	0.60713673	1.20881259	2.26312399	1	-1.36426330	0.21171266	2.59612393
1	3.15183687	1.59611261	1.43472385	1	-1.82376313	1.45751262	1.45612395
1	2.84243679	-2.45438719	2.99502397	6	-3.62666345	0.81871259	-1.70547605
40	1.75883675	-1.09668732	0.32222396	6	-3.62106347	-0.95078737	-3.48227596
6	-0.24456325	-2.44688749	0.89232397	1	-1.92866325	0.18291265	-2.84147596
1	2.91453671	3.77761269	-1.60687613	6	-3.94036317	1.00481260	3.08782411
6	1.47453678	2.57811260	-0.52937603	6	-3.60816312	-1.38218737	2.38102412
6	2.74413681	3.43261242	-0.58277601	1	-4.00966358	0.26771265	1.07972395
6	2.65093684	4.64061260	0.34922397	1	-4.99936342	0.82091260	3.29212403
1	3.62203670	2.82661247	-0.31937605	1	-3.82666326	2.05701256	2.81142402
1	3.54393673	5.26481295	0.28172398	1	-3.38506317	0.83951271	4.01822376
1	2.53743672	4.32671261	1.39182389	1	-3.97296309	1.55201268	-2.43967605
1	1.78773677	5.26111269	0.09382395	1	-3.05976319	1.36301267	-0.93807608
6	0.32313672	0.35341263	-0.99877602	1	-4.51206350	0.39311266	-1.21967614
6	1.60163677	1.19371259	-1.18477607	1	-3.94206309	-0.22138736	-4.23227596
6	1.98793674	1.31231260	-2.66507602	1	-4.51816368	-1.41948736	-3.06347609
1	0.07423672	-0.13758735	-1.94377601	1	-3.04436326	-1.72918737	-3.99047613
1	-0.47786328	1.09271264	-0.82707602	1	-4.64506340	-1.58698738	2.66182399
1	2.51293683	0.73401260	-0.69597602	1	-2.97886324	-1.63308740	3.24492407
1	2.96273685	1.79031265	-2.79017591	1	-3.35766315	-2.07178736	1.56442392
1	1.23863673	1.92111266	-3.18067598				

TS-4Me

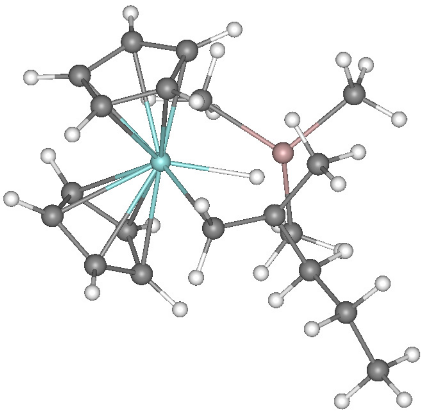

Zero-point vibrational energy	1658046.0 (Joules/Mol)
	396.28251 (Kcal/Mol)
Zero-point correction	0.631516 (Hartree/Particle)
Thermal correction to Energy	0.664706

Thermal correction to Enthalpy	0.665650
Thermal correction to Gibbs Free Energy	0.569894
Sum of electronic and zero-point Energies	-4761.108600
Sum of electronic and thermal Energies	-4761.075410
Sum of electronic and thermal Enthalpies	-4761.074466
Sum of electronic and thermal Free Energies	-4761.170222

cartesian

6	-1.71688437	2.62534785	-0.09747748	6	1.92181563	-1.32375216	0.60122252
6	-1.98158431	2.50264788	1.29032254	6	1.57041574	2.10954785	0.72782254
6	-3.33738446	2.14904785	1.45462251	1	2.18921566	-1.54485214	-0.44007745
6	-3.93028426	2.07694793	0.15912253	6	3.23261571	-1.21845210	1.41232252
6	-2.93348432	2.39204788	-0.79017746	1	1.36371565	-2.20635223	0.95412254
1	-3.08968425	2.47454786	-1.85677755	6	2.18521571	2.72244787	-0.54487753
1	-0.76568437	2.90914798	-0.53217745	1	0.88271558	2.83584785	1.18462253
1	-1.26198435	2.64864779	2.08532262	1	2.39001560	2.01384783	1.46442246
1	-3.84338427	1.99334788	2.39952254	6	4.06621552	-2.49425220	1.26202250
1	-4.97008419	1.86864781	-0.05957748	6	2.96161556	-0.93605202	2.89272261
6	-3.39378428	-1.20645213	2.13832259	1	3.82041550	-0.38235211	1.01002252
6	-4.46258450	-0.75915205	1.33052254	6	3.16971564	1.75704789	-1.21007752
6	-4.33378410	-1.35665214	0.05352252	6	2.87921572	4.05334806	-0.23967749
6	-3.21868443	-2.23925209	0.10382252	1	1.37971568	2.93664789	-1.26137745
6	-2.62918425	-2.13865209	1.37512255	1	3.58851576	2.17894793	-2.12857747
1	-1.76608431	-2.69475222	1.71912253	1	2.70551562	0.79484791	-1.46707749
1	-3.22588444	-0.94245213	3.17472267	1	4.00481558	1.54154789	-0.53377748
1	-5.23978424	-0.06905209	1.63282251	1	5.02121544	-2.41335225	1.78982246
1	-5.00258446	-1.21855211	-0.78727746	1	4.27861547	-2.70615220	0.20992252
1	-2.88898444	-2.89095211	-0.69207752	1	3.52481556	-3.35255218	1.67582250
40	-2.32348442	0.16854790	0.30192250	1	3.88451576	-0.95925206	3.47872257
6	0.02121562	-1.33355212	-2.41077733	1	2.28281569	-1.68855214	3.31202269
6	-0.89348435	-0.14265209	-2.16857743	1	2.51171565	0.05234791	3.05232263
6	-2.30178452	-0.30535209	-2.10777736	1	3.29241562	4.50534821	-1.14647746
1	-0.47798437	0.09444790	-0.67297745	1	3.70311546	3.89834785	0.46512252
1	1.05241561	-1.03905213	-2.18967748	1	2.18391562	4.76824808	0.20962253
6	-0.30648437	-2.64875221	-1.71537745	1	-0.96028435	2.00504804	-2.58667731
1	-2.90428448	0.45504791	-2.59477735	1	-0.33348435	0.96824795	-3.86597729
1	-2.67938447	-1.31415212	-2.23327732	1	0.68391562	1.32434785	-2.46787739
6	-0.55228436	0.00214791	2.25122261	6	0.74921560	-3.71785212	-2.00247741
1	-0.39678437	-1.02565217	2.58952260	1	-1.27848434	-3.01675224	-2.05947733
1	0.22181565	0.60694790	2.76402259	1	-0.38068438	-2.48345208	-0.63197744
1	-1.44098437	0.37804788	2.76142263	1	0.49631560	-4.65865183	-1.50977755

1	-0.01828438	-1.48585212	-3.49907732	1	1.73431563	-3.40675211	-1.64467752
6	-0.34338436	1.12854791	-2.78177738	1	0.82431567	-3.91125202	-3.07617736
13	0.80521560	0.29144791	0.71902251				

I-5Me

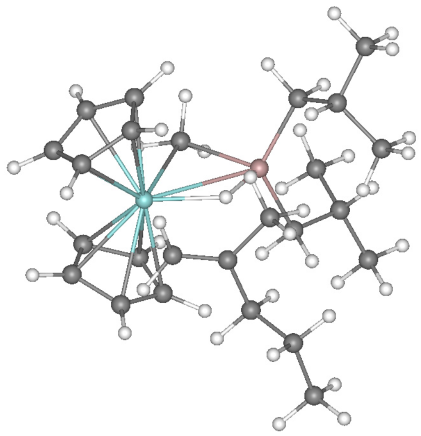

Zero-point vibrational energy	1658285.3 (Joules/Mol)
	396.33969 (Kcal/Mol)
Zero-point correction	0.631608 (Hartree/Particle)
Thermal correction to Energy	0.666340
Thermal correction to Enthalpy	0.667284
Thermal correction to Gibbs Free Energy	0.565558
Sum of electronic and zero-point Energies	-4761.121319
Sum of electronic and thermal Energies	-4761.086587
Sum of electronic and thermal Enthalpies	-4761.085643
Sum of electronic and thermal Free Energies	-4761.187368

cartesian

6	2.92140126	1.48615634	1.66519439	1	3.09070134	2.35915637	-0.86220562
6	3.88130140	0.47635639	1.88119447	1	2.81860137	1.36025643	-2.38850570
6	3.23360133	-0.60424364	2.54689431	1	-0.83129871	1.25335646	-2.11930561
6	1.88140130	-0.23524362	2.75529432	1	-0.02799869	2.36055636	-3.23070550
6	1.67200136	1.03885639	2.17809439	1	0.57400131	0.70625639	-3.06700563
1	0.74170136	1.59385645	2.18679452	6	-0.23399866	4.16235638	-0.98350567
1	3.11620140	2.45315623	1.22389436	1	-0.34249866	2.32995629	0.14689437
1	4.93000126	0.53025639	1.61679447	1	1.17430139	3.22375631	0.35659435
1	3.70650125	-1.52004361	2.88209438	1	-0.02299869	-2.45954370	1.90799439
1	1.12850142	-0.83034360	3.25549436	1	1.64290130	-2.64524364	1.71269441
6	2.91060138	-1.26544356	-2.07600570	1	0.59880131	-3.27294374	0.46799436
6	4.08740139	-0.81834358	-1.40570557	6	-0.80119860	5.06615639	0.10959436
6	4.37000132	-1.74214363	-0.37030566	1	-1.03639865	3.86245608	-1.66390562

6	3.35220122	-2.72254372	-0.36500564	1	0.50490135	4.70885658	-1.57900560
6	2.45510149	-2.43134379	-1.43220556	1	-1.27549863	5.94955635	-0.32190564
1	1.57600141	-3.00084376	-1.70630562	1	-0.01399869	5.40695667	0.78799433
1	2.44480133	-0.80454361	-2.93750548	1	-1.55349874	4.53785658	0.70269436
1	4.69510126	0.03745639	-1.67290556	6	-2.85859871	-2.34434366	-3.30560565
1	5.21600151	-1.70344365	0.30409437	1	-1.33429861	-0.93444359	-2.80170560
1	3.28970122	-3.57054377	0.30479437	6	-3.16039872	-0.32044363	-1.85560560
40	2.23300123	-0.53654361	0.25699437	6	-3.80119848	0.19635640	3.08059430
6	0.75330132	-2.40904379	1.12199438	6	-2.88449860	-2.10234356	2.64829445
1	0.43830132	-0.10204361	-0.40320563	1	-3.67939854	-0.76864362	1.17259443
13	-0.75629866	-1.12344360	0.30529436	1	-3.83789873	-2.52404380	2.97879434
6	-1.64239860	-2.19944382	-1.08160555	1	-2.21489859	-2.08344364	3.51759434
6	-1.69899869	-0.08174361	1.68429446	1	-2.46579862	-2.80014372	1.91139436
6	-2.18249869	-1.41474354	-2.29370570	1	-4.78469849	-0.21334362	3.32999444
1	-0.97419870	-2.99664378	-1.43860555	1	-3.94599867	1.20455635	2.68179440
1	-2.48829865	-2.72724366	-0.61180562	1	-3.22659874	0.28085637	4.00999451
6	-3.06119871	-0.69234359	2.07759452	1	-3.51119852	0.27045637	-2.70750570
1	-1.09239864	0.02165639	2.59899449	1	-2.71729875	0.37495643	-1.12830555
1	-1.86409855	0.94775641	1.32979441	1	-4.03979874	-0.76654363	-1.37690556
6	2.39690137	1.82465637	-1.50290561	1	-3.22179866	-1.79204357	-4.17800570
6	1.05980134	2.04235625	-1.43340564	1	-3.71529865	-2.84674382	-2.84380555
6	0.14420134	1.54625642	-2.51760554	1	-2.16799855	-3.11654377	-3.65670562
6	0.42820132	2.90965629	-0.38010564				

S1.9. Interaction with H_2

H2 Hydrogen

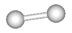

Zero-point vibrational energy	27227.7 (Joules/Mol)
	6.50757 (Kcal/Mol)
Zero-point correction	0.010370 (Hartree/Particle)
Thermal correction to Energy	0.012731
Thermal correction to Enthalpy	0.013675
Thermal correction to Gibbs Free Energy	-0.001099
Sum of electronic and zero-point Energies	-1.153736
Sum of electronic and thermal Energies	-1.151375
Sum of electronic and thermal Enthalpies	-1.150431
Sum of electronic and thermal Free Energies	-1.165205

cartesian

1	0.00000000	0.00000000	0.36780000	1	0.00000000	0.00000000	-0.36780000

_I-2H_a_H2

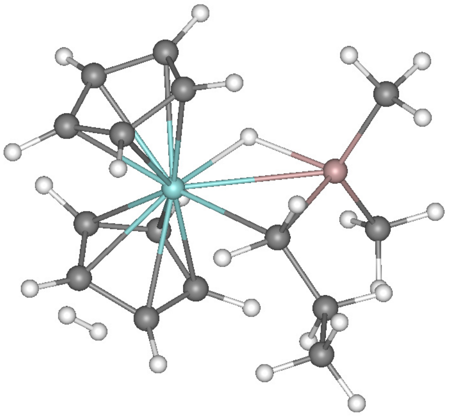

Zero-point vibrational energy	945988.3 (Joules/Mol)
	226.09663 (Kcal/Mol)
Zero-point correction=	0.360308 (Hartree/Particle)
Thermal correction to Energy=	0.382778
Thermal correction to Enthalpy=	0.383723
Thermal correction to Gibbs Free Energy=	0.310018
Sum of electronic and zero-point Energies=	-4369.628294
Sum of electronic and thermal Energies=	-4369.605823
Sum of electronic and thermal Enthalpies=	-4369.604879
Sum of electronic and thermal Free Energies=	-4369.678583

cartesian

6	-0.12040000	-2.23469996	-0.91839999
6	0.52300000	-2.49469995	0.31639999
6	1.89900005	-2.17490005	0.17600000
6	2.10780001	-1.73529994	-1.16159999
6	0.86240000	-1.77230000	-1.83650005
1	0.68629998	-1.50689995	-2.87159991
1	-1.16910005	-2.38910007	-1.14069998
1	0.04660000	-2.87540007	1.21340001
1	3.05819988	-1.44760001	-1.59340000
6	0.93199998	2.34240007	0.77609998
6	1.73899996	1.53269994	1.61940002
6	2.79200006	0.99519998	0.83829999
6	2.63949990	1.47780001	-0.49140000
6	1.49880004	2.32170010	-0.52420002
1	1.12860000	2.85960007	-1.38759995
1	0.05810000	2.90540004	1.08000004
1	1.58140004	1.36259997	2.67860007
1	3.30110002	1.27260005	-1.32379997
40	0.69550002	-0.01320000	-0.05690000
1	-0.29290000	0.68250000	-1.54980004
13	-1.99269998	0.77300000	-1.02760005
6	-2.52979994	2.63569999	-0.84410000
6	-2.94689989	-0.58560002	-2.05620003
1	-1.71469998	3.30730009	-0.55430001
1	-2.94129992	3.02080011	-1.78310001
1	-3.32150006	2.75399995	-0.09400000
1	-2.33929992	-0.97399998	-2.88229990
1	-3.28870010	-1.44780004	-1.47370005
1	-3.84170008	-0.14760000	-2.51200008
6	-2.85540009	-0.97350001	2.81669998
6	-1.42780006	0.16270000	1.03869998
6	-2.61400008	-0.78369999	1.31710005
1	-3.52800012	-0.37880000	0.86309999
1	-1.64129996	1.13360000	1.50619996
1	-0.60140002	-0.22849999	1.69760001
1	-2.44619989	-1.75370002	0.83710003
1	-1.98210001	-1.42719996	3.29679990
1	-3.04859996	-0.01550000	3.30609989
1	3.58960009	0.35609999	1.19700003
1	2.66090012	-2.29320002	0.93680000
1	-3.71320009	-1.62530005	2.99519992
1	1.72679996	-1.33060002	3.55399990
1	1.12179995	-1.61360002	3.24130011

S1.10. Transition states search and proof

As was mentioned in the main article, transition states were found by draft scanning using Priroda program [48] followed by presize scanning with sequential changing of key geometric parameters with a step of 0.01 Å and then by Berny optimization using Gaussian-09 program package [36]. Verification of the geometries of the transition states was performed using the standard IRC procedure as implemented in Gaussian-09 with the command line:
irc=(maxpoints=20,calcfc).
Note that in some cases the IRC sequences included one artifact point (see Fig. 1a for example), and IRC procedures were repeated in duplicative mode for reverse and forward directions. In view of the visibility of the IRC procedure for the confirmation of the TS identity, we attached animation files named *_IRC.gif to the SI data in addition to animation files that reflect the imaginary frequency vibration for all transition states mentioned in the manuscript.

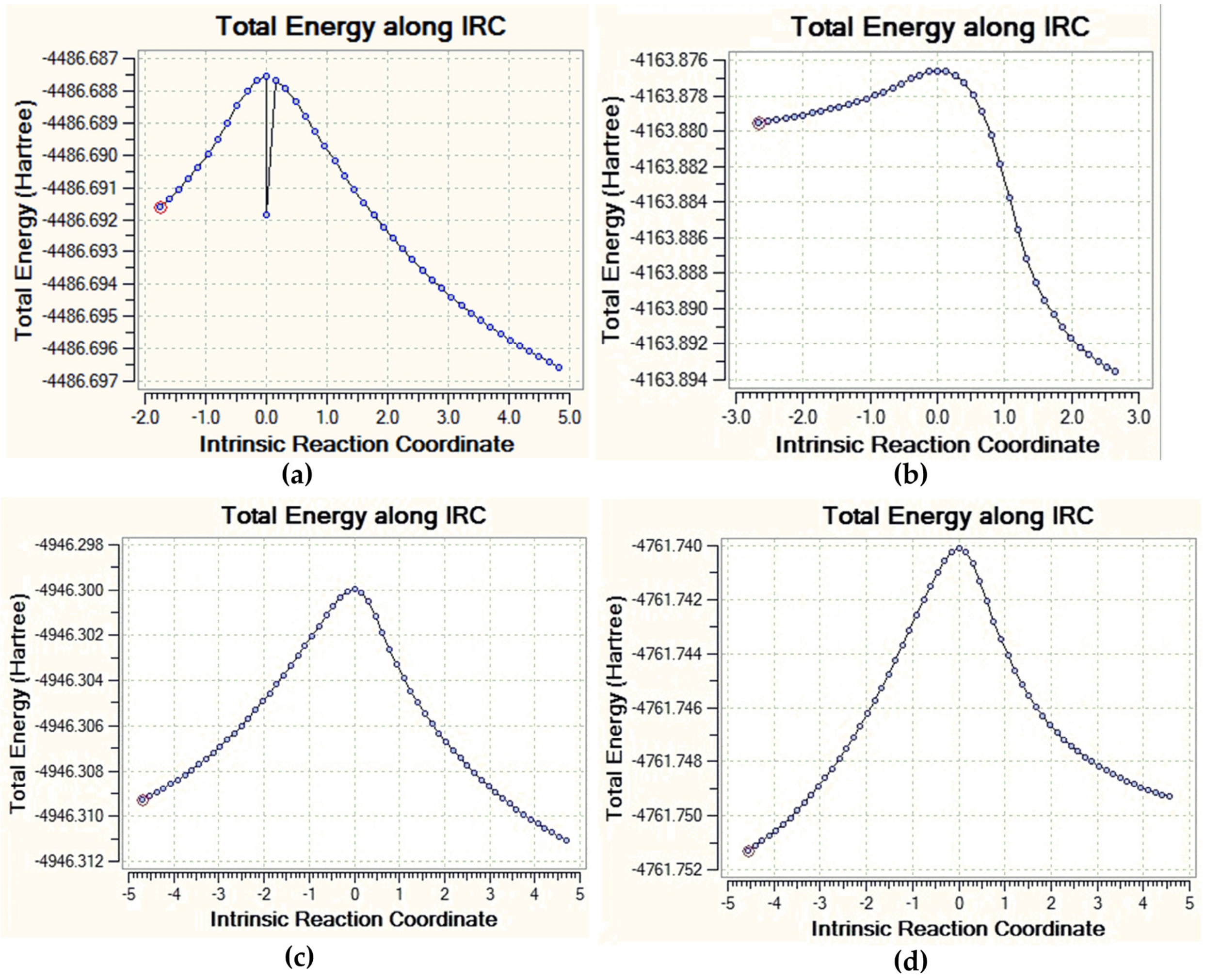

Figure S2. IRC plots (GaussView) for transition states: **(a) TS-4H** (one artifact point); **(b) TS-4-**; **(c) TS-4Cl**; **(d) TS-4Me_dibu**. Animations for **(b–d)** can be found in the Supporting Information as *_IRC.gif.

S2. Oligomerization experiments

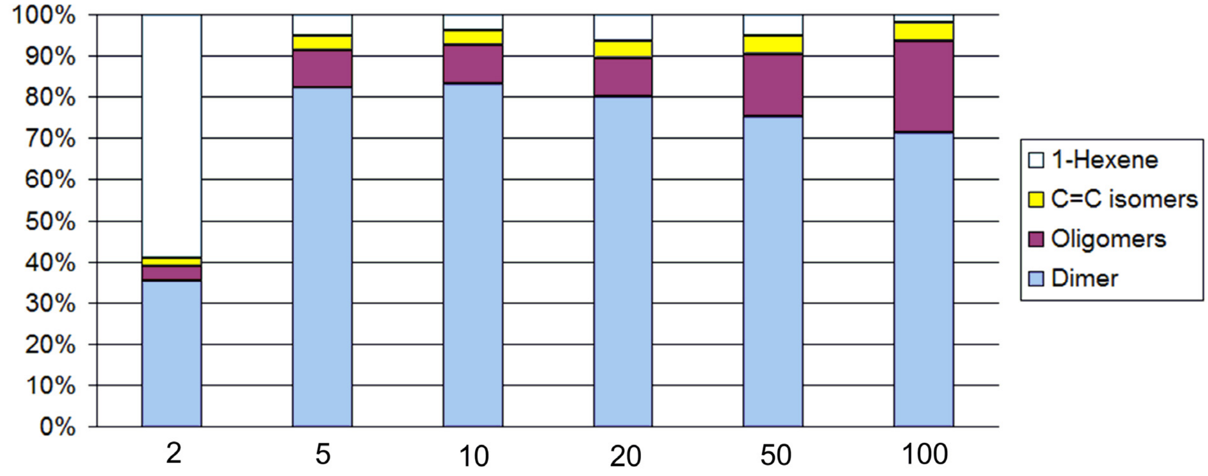

Figure S2. The dependence of the composition of the reaction mixtures (1-hexene oligomerization in bulk, 60 °C, 4 h) from $Al_{MMAO-12}/Zr$ ratio.

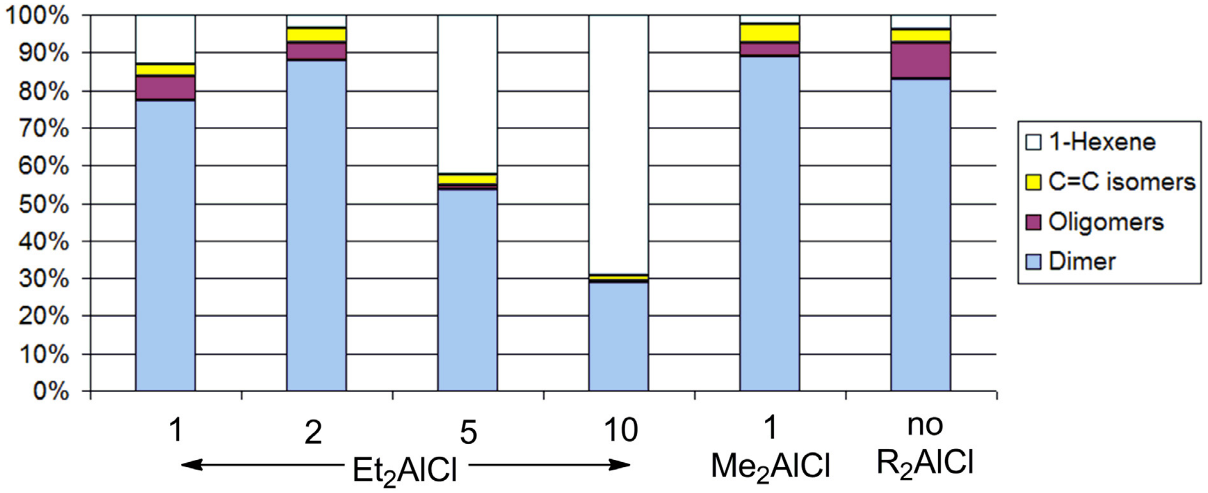

Figure S3. The affect of R_3AlCl on the composition of the reaction mixtures (1-hexene oligomerization in bulk, 60 °C, 4 h, $Al_{MMAO-12}/Zr = 10$).
