# Supplementary figures and images for: Zirconocene-Catalyzed Dimerization of α-Olefins: DFT Modeling of the Zr-Al Binuclear Reaction Mechanism
Source: Molecules. 2019 Oct 2;24(19):3565. doi: 10.3390/molecules24193565 (PMC6803839; doi:10.3390/molecules24193565)

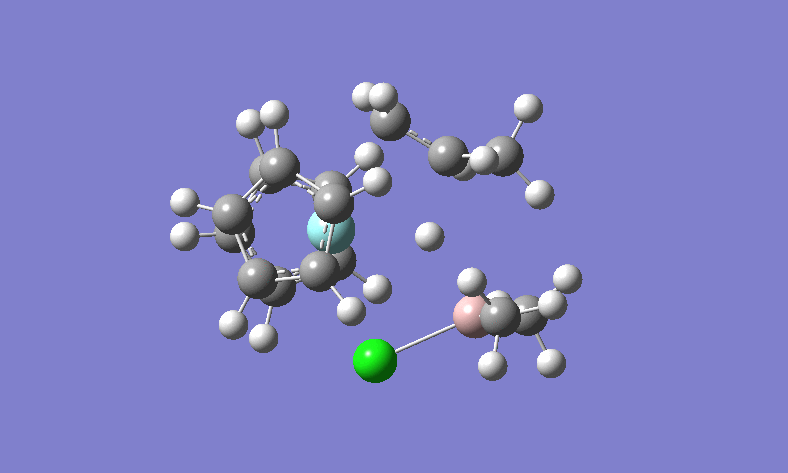

Supplement: Supplementary file 1 [file molecules-24-03565-s001.zip › TS_animation/TS-1Cl.gif]

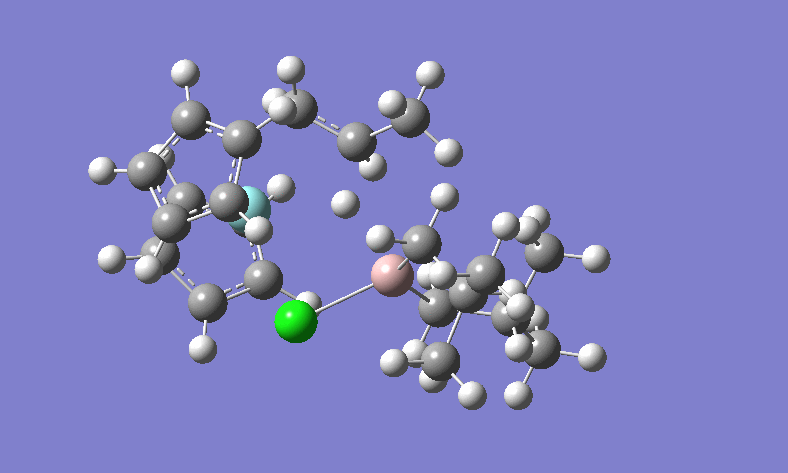

Supplement: Supplementary file 1 [file molecules-24-03565-s001.zip › TS_animation/TS-1Cl_dibu.gif]

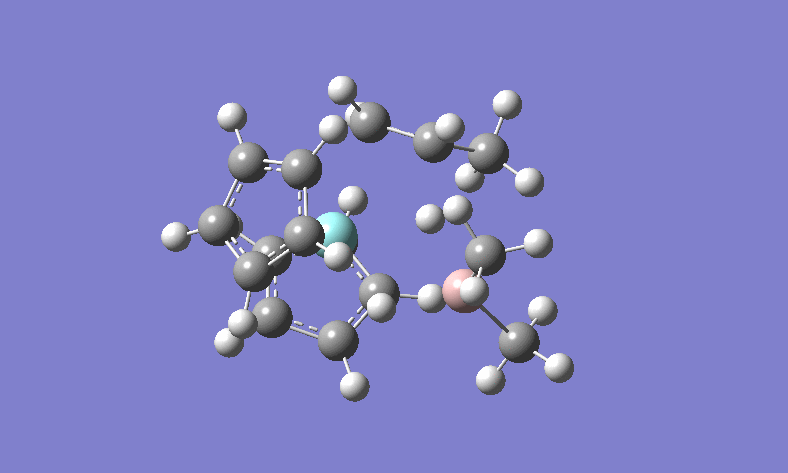

Supplement: Supplementary file 1 [file molecules-24-03565-s001.zip › TS_animation/TS-1H.gif]

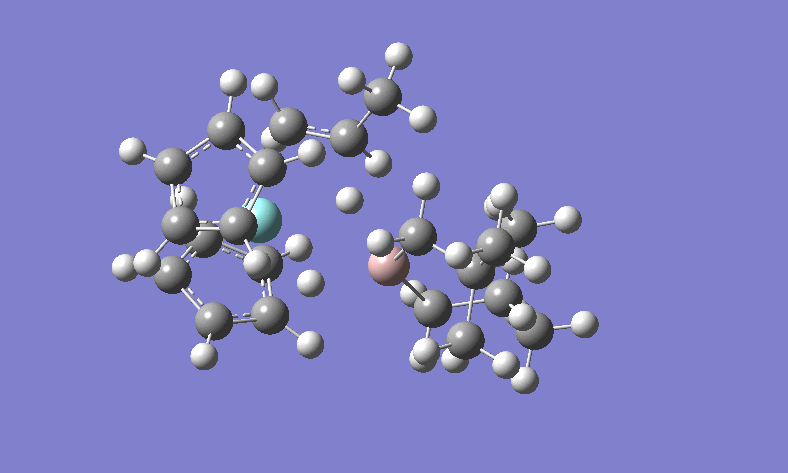

Supplement: Supplementary file 1 [file molecules-24-03565-s001.zip › TS_animation/TS-1H_dibu.gif]

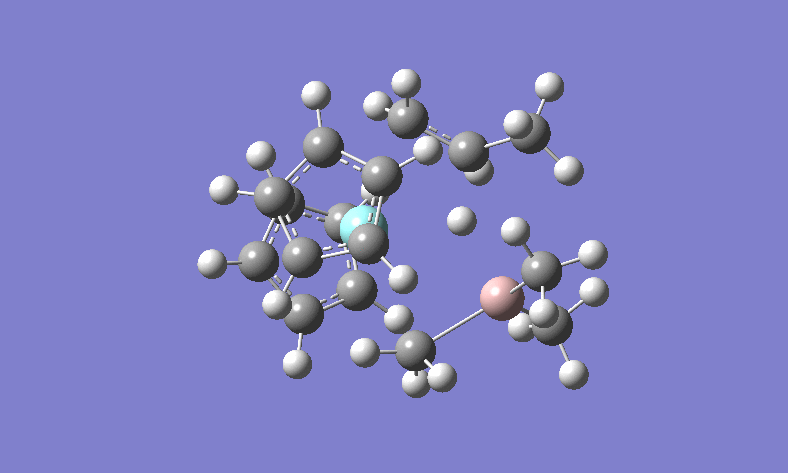

Supplement: Supplementary file 1 [file molecules-24-03565-s001.zip › TS_animation/TS-1Me.gif]

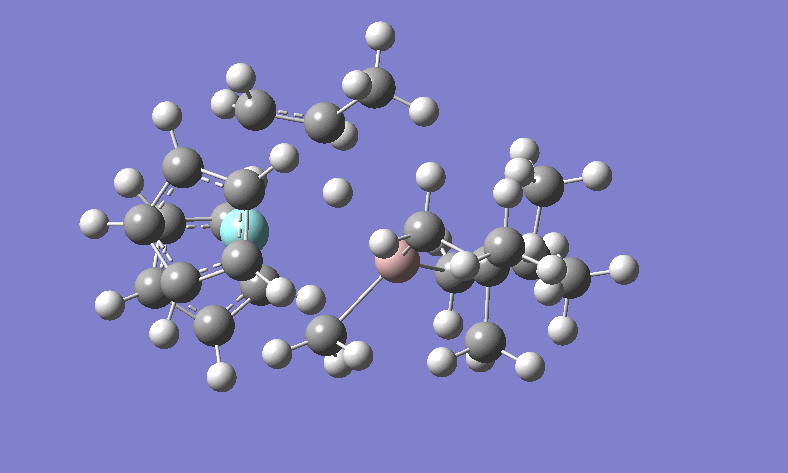

Supplement: Supplementary file 1 [file molecules-24-03565-s001.zip › TS_animation/TS-1Me_dibu.gif]

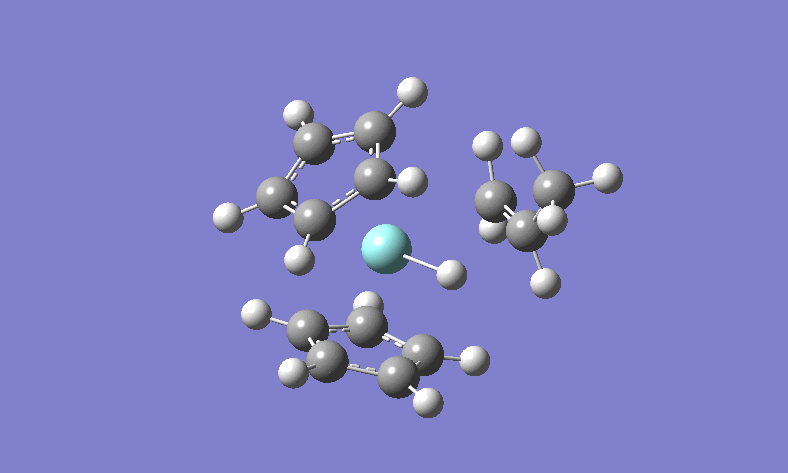

Supplement: Supplementary file 1 [file molecules-24-03565-s001.zip › TS_animation/TS-1_--.gif]

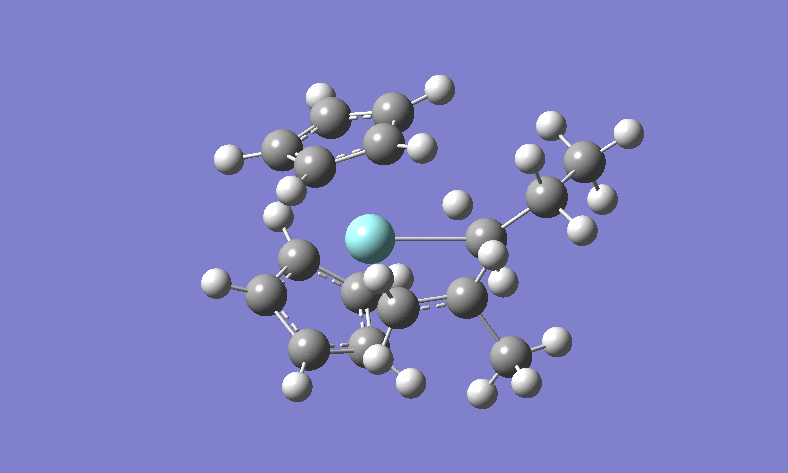

Supplement: Supplementary file 1 [file molecules-24-03565-s001.zip › TS_animation/TS-2--.gif]

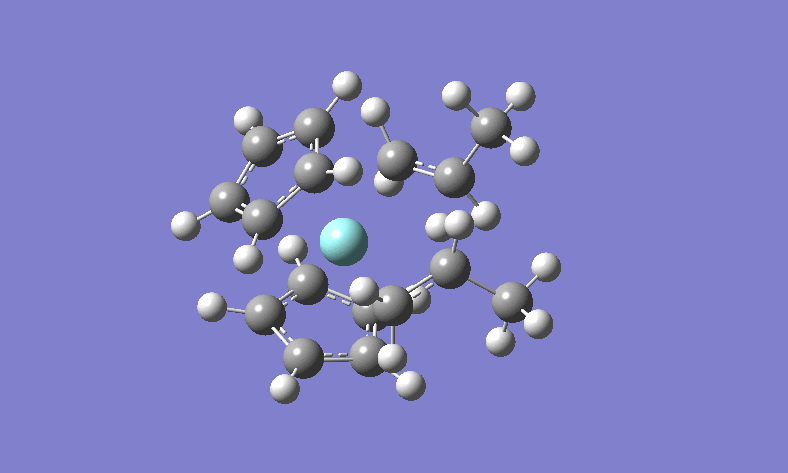

Supplement: Supplementary file 1 [file molecules-24-03565-s001.zip › TS_animation/TS-3--.gif]

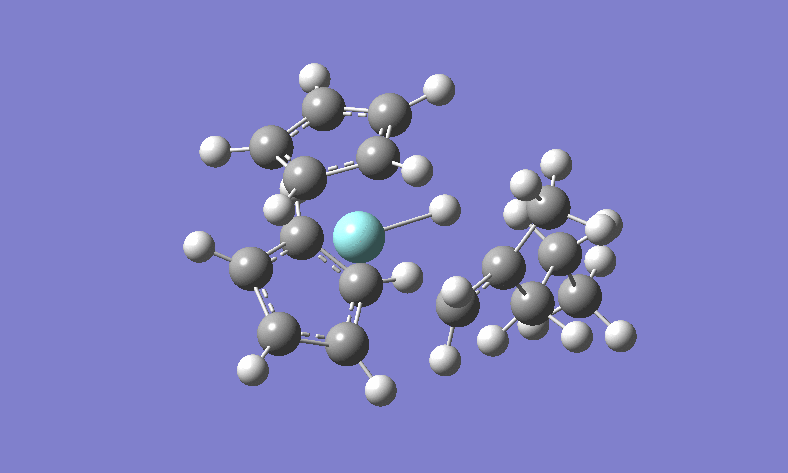

Supplement: Supplementary file 1 [file molecules-24-03565-s001.zip › TS_animation/TS-4--.gif]

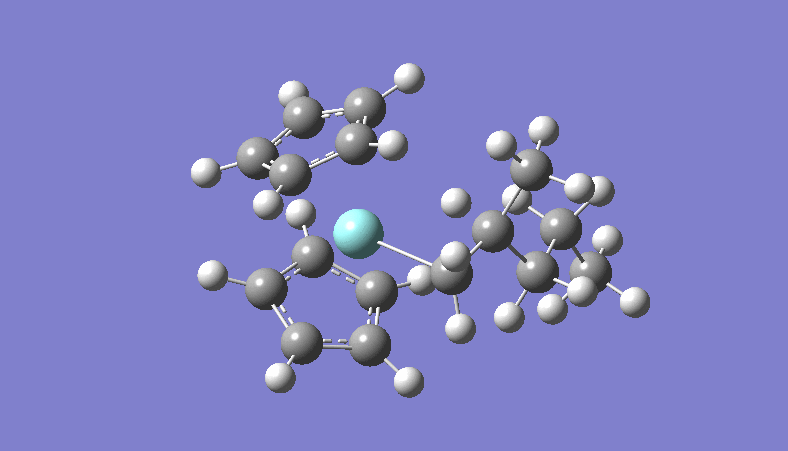

Supplement: Supplementary file 1 [file molecules-24-03565-s001.zip › TS_animation/TS-4--_IRC.gif]

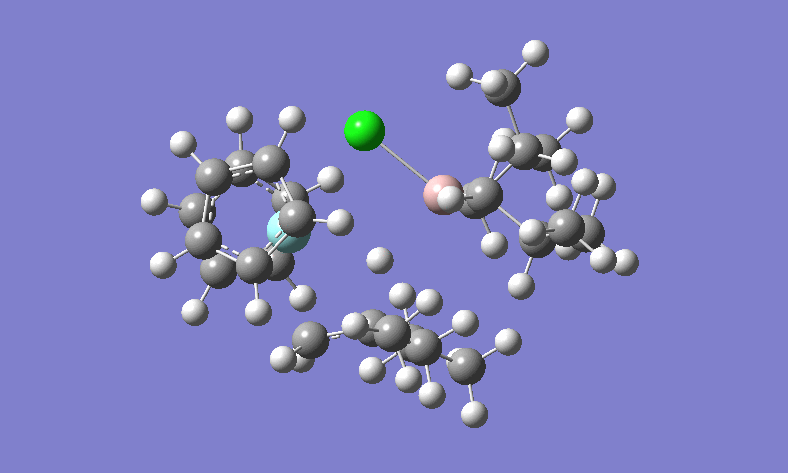

Supplement: Supplementary file 1 [file molecules-24-03565-s001.zip › TS_animation/TS-4Cl.gif]

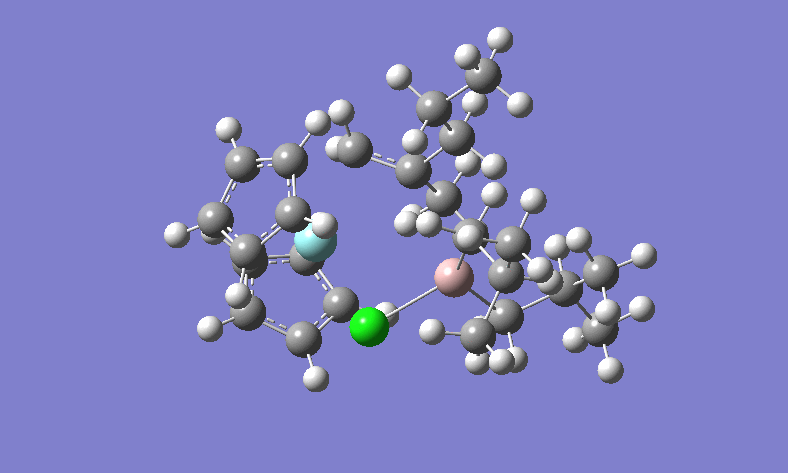

Supplement: Supplementary file 1 [file molecules-24-03565-s001.zip › TS_animation/TS-4Cl_dibu.gif]

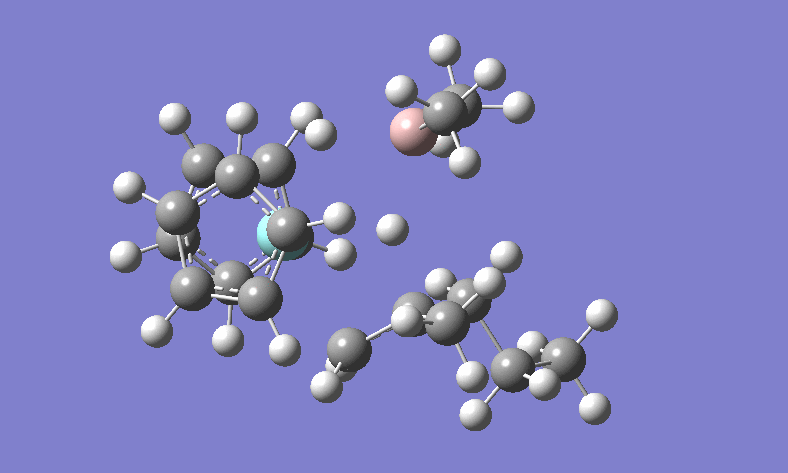

Supplement: Supplementary file 1 [file molecules-24-03565-s001.zip › TS_animation/TS-4H.gif]

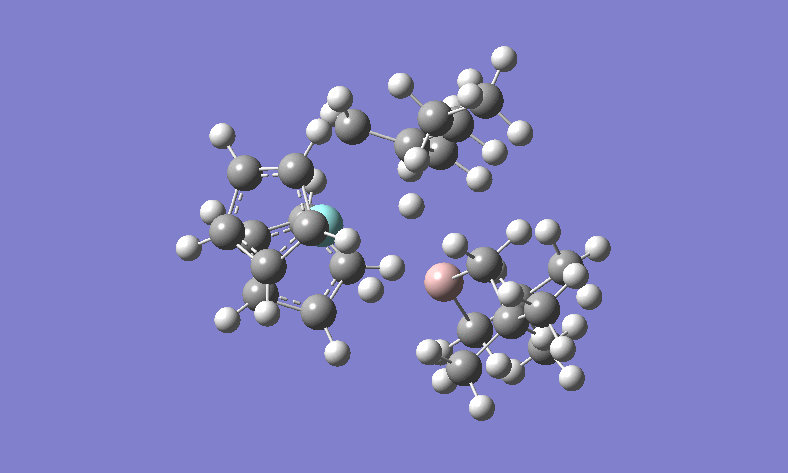

Supplement: Supplementary file 1 [file molecules-24-03565-s001.zip › TS_animation/TS-4H_dibu.gif]

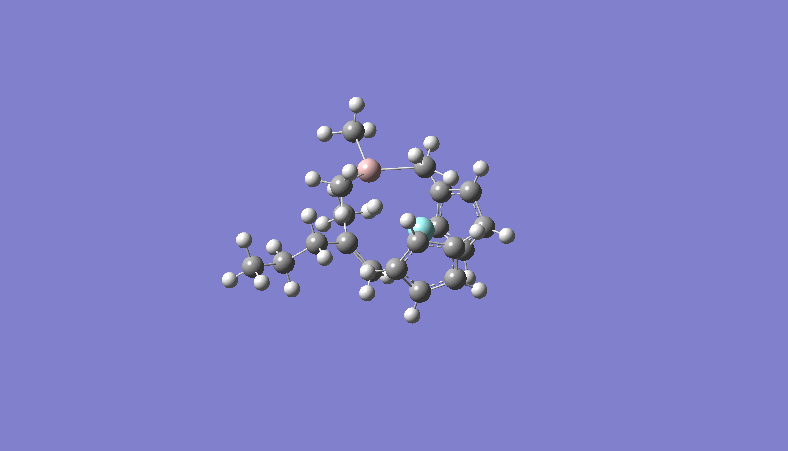

Supplement: Supplementary file 1 [file molecules-24-03565-s001.zip › TS_animation/TS-4Me.gif]

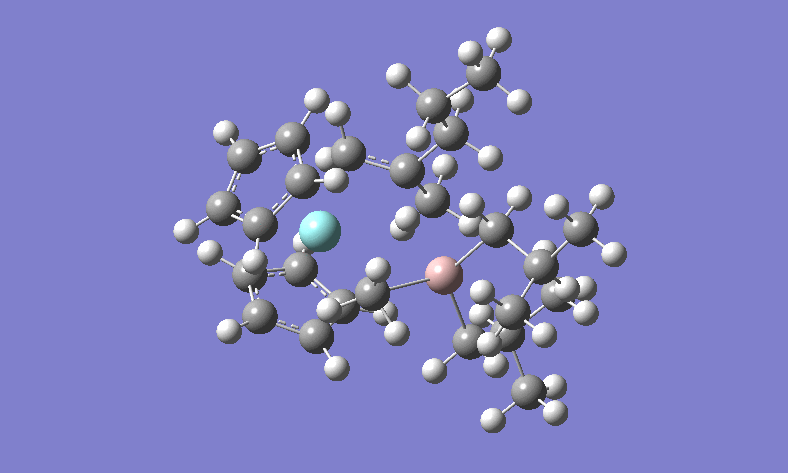

Supplement: Supplementary file 1 [file molecules-24-03565-s001.zip › TS_animation/TS-4Me_dibu.gif]

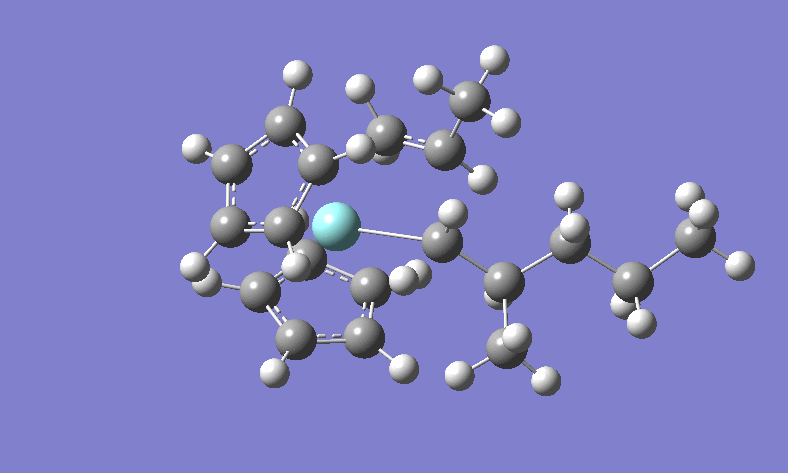

Supplement: Supplementary file 1 [file molecules-24-03565-s001.zip › TS_animation/TS-5--.gif]

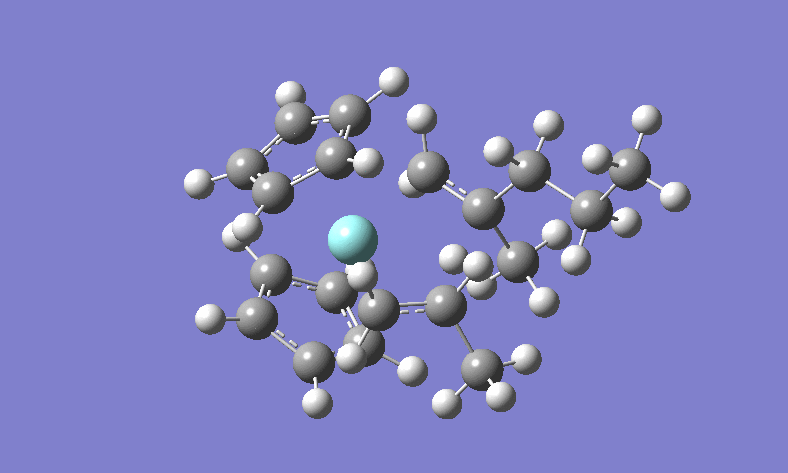

Supplement: Supplementary file 1 [file molecules-24-03565-s001.zip › TS_animation/TS-6--.gif]

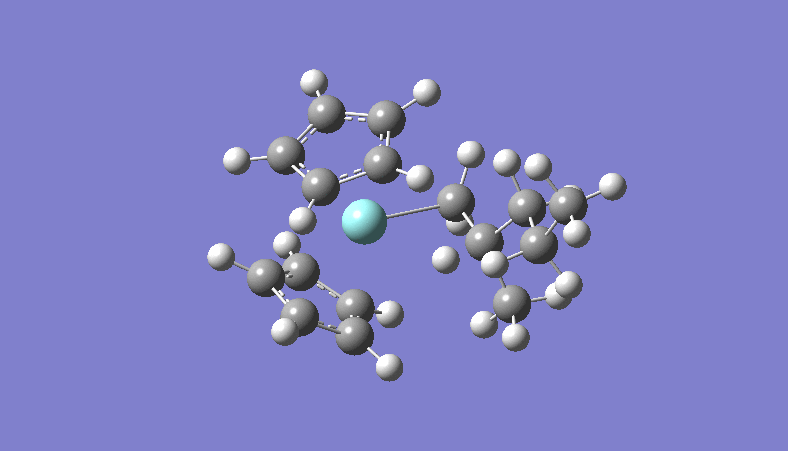

Supplement: Supplementary file 1 [file molecules-24-03565-s001.zip › TS_IRC_amination/TS-4--_IRC.gif]

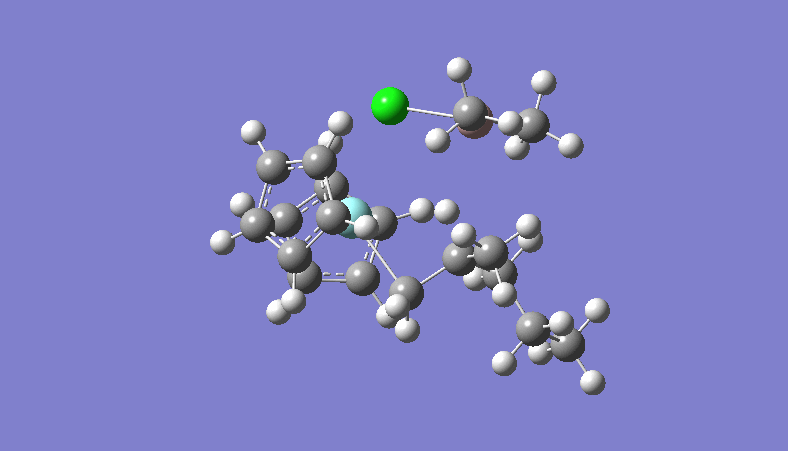

Supplement: Supplementary file 1 [file molecules-24-03565-s001.zip › TS_IRC_amination/TS-4Cl_IRC.gif]

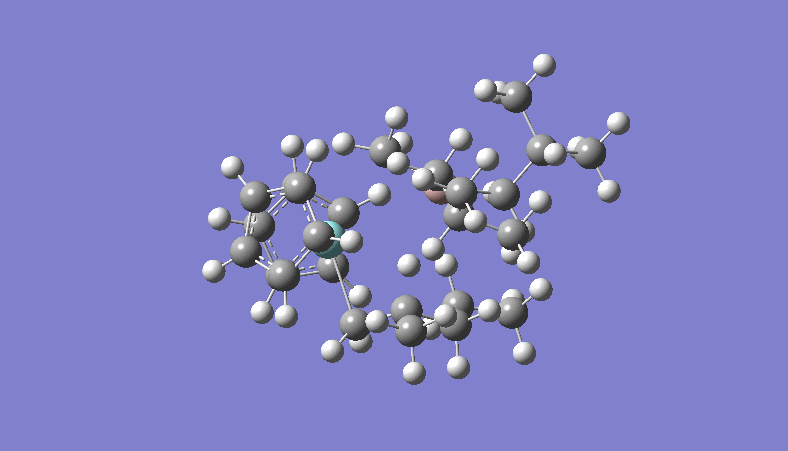

Supplement: Supplementary file 1 [file molecules-24-03565-s001.zip › TS_IRC_amination/TS-4Me_dibu_IRC.gif]
